# Roles of Cardiometabolic Factors in Mediating the Causal Effect of Type 2 Diabetes on Cardiovascular Diseases: A Two-Step, Two-Sample Multivariable Mendelian Randomization Study

**DOI:** 10.3389/fcvm.2022.813208

**Published:** 2022-02-24

**Authors:** Ken Chen, Zhenhuang Zhuang, Chunli Shao, Jilin Zheng, Qing Zhou, Erdan Dong, Tao Huang, Yi-Da Tang

**Affiliations:** ^1^Department of Cardiology, State Key Laboratory of Cardiovascular Disease, National Center for Cardiovascular Diseases, Fuwai Hospital, Chinese Academy of Medical Sciences and Peking Union Medical College, Beijing, China; ^2^Department of Cardiology, Graduate School of Peking Union Medical College, Chinese Academy of Medical Sciences and Peking Union Medical College, Beijing, China; ^3^Department of Epidemiology & Biostatistics, School of Public Health, Peking University, Beijing, China; ^4^Department of Cardiology and Institute of Vascular Medicine, Peking University Third Hospital, Beijing, China; ^5^Key Laboratory of Molecular Cardiovascular Science, Ministry of Education, Beijing, China; ^6^Department of Cardiology and Institute of Vascular Medicine, NHC Key Laboratory of Cardiovascular Molecular Biology and Regulatory Peptides, Beijing Key Laboratory of Cardiovascular Receptors Research, Peking University Third Hospital, Beijing, China; ^7^Key Laboratory of Molecular Cardiovascular Science, Ministry of Education, The Institute of Cardiovascular Sciences, Peking University, Beijing, China; ^8^Department of Global Health, School of Public Health, Peking University, Beijing, China; ^9^Key Laboratory of Molecular Cardiovascular Sciences (Peking University), Ministry of Education, Beijing, China; ^10^Center for Intelligent Public Health, Institute for Artificial Intelligence, Peking University, Beijing, China

**Keywords:** type-2 diabetes (T2DM), cardiovascular disease, blood pressure, triglycerides (TG), Mendelian randomization study

## Abstract

**Objective:**

The objective of this study is to investigate the roles of cardiometabolic factors (including blood pressure, blood lipids, thyroid function, body mass, and insulin sensitivity) in mediating the causal effect of type 2 diabetes (T2DM) on cardiovascular disease (CVD) outcomes.

**Design:**

Two-step, two-sample multivariable Mendelian randomization (MVMR) study.

**Setting:**

International genome-wide association study (GWAS) consortia data.

**Exposure:**

Type 2 diabetes, blood pressure: systolic blood pressure (SBP), diastolic blood pressure (DBP); blood lipids: low-density lipoprotein (LDL), high-density lipoprotein (HDL), total cholesterol (TC), triglycerides (TG); thyroid function: hyperthyroidism, hypothyroidism; body mass index (BMI), waist-hip-ratio (WHR), and insulin sensitivity.

**Main Outcomes:**

Cardiovascular disease includes coronary heart disease (CHD), myocardial infarction (MI), and stroke.

**Methods:**

Summary-level data for exposures and main outcomes were extracted from GWAS consortia. We used two-sample MR to illustrate the causal effect of T2DM on CVD subtypes and regression-based MVMR to quantify the possible mediation effects of cardiometabolic factors on CVD.

**Results:**

Each additional unit of log odds of T2DM increased 16% risk of CHD [odds ratio (OR): 1.16, 95% CI: 1.12–1.21], 15% risk of myocardial infarction (MI) (OR: 1.15, 95% CI: 1.10–1.20), and 10% risk of stroke (OR: 1.10, 95% CI: 1.06–1.13). In mediation analysis, SBP, DBP, and TG were found as main mediators, while the mediation effects of other cardiometabolic factors were not significant. The proportion of total effect of T2DM on CHD mediated by SBP, DBP, and TG was 16% (95% CI: 8–24%), 7% (95% CI: 1–13%) and 10% (95% CI: 2–18%), respectively. Mediation effect of SBP and DBP on MI and stroke, TG on MI was also prominent, while mediation effect of TG on stroke was not significant. The combined mediation effect of all the three mediators accounted for 29%, 26%, and 13% of the total effect of T2DM on CHD, MI, and stroke, respectively.

**Conclusion:**

Systolic blood pressure, DBP, and TG mediate a substantial proportion of the causal effect of T2DM on CVD and thus interventions on these factors might reduce the considerable excess risk of CVD among patients with T2DM.

## Introduction

Globally, cardiovascular disease (CVD) remains the leading cause of mortality which accounts for over 17 million deaths annually (1). Compelling observational studies have proved that type 2 diabetes (T2DM), which will influence up to 550 million patients by 2030 (2), has always been supposed to be associated with an increased risk of CVD (3, 4). It is estimated that patients with T2DM over 50 years old lost 6 years on average than nondiabetic population, and 58% of this difference can be attributable to CVD (5). However, controversial evidence from previous studies showed that intensive glycemic control *per se* might even increase CVD risk (6). Thus, exploring treatment strategies besides glycemic control is essential for the management of patients with T2DM. As patients with T2DM are often present with multiple cardiometabolic disorders, understanding whether these risk factors have roles in mediating the causal effect of T2DM on CVD would provide new intervention targets to reduce excess CVD risk for patients with T2DM.

An observational study has investigated the influence of T2DM on the subtype of CVD mediated by blood pressure, cholesterol, glucose, and other metabolic factors individually and concluded that decreasing systolic blood pressure (SBP) and total cholesterol (TC)/ high-density lipoprotein (HDL) ratio could reduce 10-year CVD risk (7). Besides, an open, parallel trial aiming at patients with T2DM with a mean follow-up of 7.8 years concluded that intensified interventions including decline glycosylated hemoglobin, blood pressure, TC, triglycerides (TGs), and urinary albumin excretion rate brought huge benefit in reducing CVD events by 50% (8). However, observational studies have always been criticized for their weakness in proving causal associations because of unknown or inadequately measured confounding factors. Moreover, conducting a well-designed randomized-controlled trial (RCT) is both time-consuming and costly which may take decades. Therefore, it remains largely unknown whether such mediation effects are causal.

Mendelian randomization (MR) is a genetic epidemiological method using genetic variants as instrumental variables for risk factors to explore the unbiased effect on diseases (9). Unlike traditional observational studies, MR studies are less likely to be biased by confounding factors or measurement errors and thus are becoming widely used to investigate the potential causal effect of exposures on outcomes (10). The two-sample MR method means genetic variants for exposure and outcome are extracted from the different dataset which makes it more robust in statistical power, but alternative sources of bias may be caused if the two samples used in a study overlap (11). Previous MR studies have proved the causal effect of T2DM on stroke, coronary heart disease (CHD) (12, 13), the causal effect of T2DM on blood pressure (14), and also causal effects of metabolic factors on CVD (15, 16). Results from these studies indicate that these cardiometabolic factors might partly explain the causal effect of T2DM on CVD, but none of them have quantified the mediation effect. Therefore, to understand how much of the causal effect of T2DM on CVD is mediated by cardiometabolic factors, separately and in combinations, we conducted a two-step, two-sample MR study. We quantified how much of the effects of T2DM on CVD subtypes such as CHD, myocardial infarction (MI), and stroke were mediated through cardiometabolic factors including SBP, diastolic blood pressure (DBP), low-density lipoprotein (LDL), HDL, TC, TG, etc. individually and in all possible combinations by analyzing genome-wide association study (GWAS) summary statistics from international genetic consortia.

## Methods

### Overall Study Design

The first step of our two-step MR study is to determine the causal effect of T2DM on each subtype of CVD (CHD, MI, and stroke). The second step of this study is to explore and quantify the possible mediation effects of cardiometabolic factors on the causal effect of T2DM on each subtype of CVD.

### Data Sources

#### Genetic Instrumental Variables for T2DM

We obtained genetic variants for T2DM from a meta-analysis of GWAS which consists of over 16 million genetic variants of European ancestry (17). Sources of participants include DIAGRAM (12,171 cases and 56,862 controls), GERA (6,905 cases and 46,983 controls), and UKB datasets (21,147 cases and 434,460 controls) (17).

#### Genetic Instrumental Variables for Potential Mediators

A total of 12 cardiometabolic factors including blood pressure, blood lipids, thyroid function, body mass index (BMI), and insulin sensitivity were selected as potential mediators. Genetic variants of SBP and DBP were both extracted from a genetic analysis of over one million people drawn from UK Biobank (UKB) (*N* = 458,577 Europeans) (18) and the International Consortium of Blood Pressure-Genome Wide Association Studies (ICBP) (*N* = 299,024 Europeans) (19, 20). For TG, hyperthyroidism, and hypothyroidism, we obtained data from online public GWAS of European ancestry participants provided by Neale lab and Ben Elsworth through R software (R Consortium, Boston, MA) TwoSampleMR package (http://gwas-api.mrcieu.ac.uk/). We obtained genetic variants of HDL, LDL, and TC from a GWAS of 9,961 European participants (21). Genetic variants of very low-density lipoprotein (VLDL) were identified from a GWAS including 19,273 European participants (22). For BMI and WHR, SNPs were extracted from GWAS including ~700,000 and 224,459 European participants, respectively (23, 24). We acquired genetic data sets for insulin sensitivity from a GWAS including 16,753 European participants (25).

#### Genetic Instrumental Variables for CVD

Genome-wide association study summary statistics for CHD and MI were obtained from a genome-wide association meta-analysis of 48 studies including 60,801 cases and 123,504 controls originating from mixed ancestry (77% from European, 13 and 6% from south and east Asian, others from Hispanic or African-American) (26). Genetic variants of stroke were extracted from a multiancestry meta-analysis of 29 studies which includes 67,162 cases and 454,450 controls (the number of studies with European ancestry, African ancestry, Asian ancestry, and Latin American population GWAS studies was 17, 5, 6, and 1 respectively) (27). Details of data sources for T2DM, potential mediators, and outcomes are shown in Supplementary Table 1. All the genetic variants used as instrumental variables are shown in Supplementary Tables 2–14.

### Statistical Analysis

#### Effect of T2DM on CVD Subtypes

The causal effects of T2DM on CVD were estimated using a two-sample MR method. We used the inverse-variance weighted (IVW) approach to estimate the causal effect of T2DM on CVD subtypes and each potential mediator. Results were shown using odds ratio (OR) and 95% CI. *P* < 0.05 for the IVW approach was considered suggestive for the potential association.

#### Effects of T2DM on Cardiometabolic Factors

First, we used a two-sample MR to estimate the effect of T2DM on each cardiometabolic factor. Results were shown using β coefficient and 95% CI. As some cardiometabolic factors were extracted from the same database, a Bonferroni corrected *p*-value threshold was considered significant for them (SBP and DBP: Bonferroni corrected *p* < 0.025, TG, hyperthyroidism, hypothyroidism, HDL, LDL, and TC: Bonferroni corrected *p* < 0.017) and *p* < 0.05 was considered suggestive for the potential association. Those factors with a *p* > 0.05 which indicated not statistically significant causal association with T2DM were excluded.

#### Effects of Cardiometabolic Factors on CVD Subtypes

Second, estimates of the effects of cardiometabolic factors on CVD subtypes adjusting for T2DM were obtained by regression-based multivariable MR (MVMR) (28). Results were shown using OR and 95% CI. A Bonferroni corrected *p*-value threshold was considered significant an*d p* < 0.05 was considered suggestive for the potential association. Cardiometabolic factors did not meet *p* < 0.05 standard were excluded.

#### Mediation Effects of Cardiometabolic Factors

Furthermore, an estimate of the effect of T2DM on each cardiometabolic factor was multiplied with an estimate of the effect of each cardiometabolic factor on CVD subtypes, respectively, to obtain the mediation effect of each cardiometabolic factor individually. Then, we divided the mediation effect by the total effect of T2DM on CVD subtypes to obtain the proportion mediated by each mediator. In sum, we took all the mediators into account and obtained a causal effect of T2DM on each subtype of CVD after adjusting for all possible mediators. The proportion mediated by all the mediators was obtained by subtracting the causal effect of T2DM on each subtype of CVD after adjusting for all mediators from the total effect of T2DM on CVD subtypes, then dividing the result by the total effect. All the aforementioned analyses were performed using R version 4.0.3 (The R Foundation for Statistical Computing) through the TwoSampleMR package and Mendelian Randomization package. SEs were calculated using rules derived from the Gaussian equation for normally distributed errors in order to fit different situations such as addition or subtraction and multiplication or division (Supplementary methods).

#### Sensitivity Analyses

We applied other sensitivity analyses including simple median, weighted median, MR-Egger regression, MR-PRESSO to detect potential bias from invalid variables and potential pleiotropy, single SNP analysis, and leave-one-out analysis to investigate the influence of possible outlying genetic variants. Full details of these methods were shown in the Supplementary methods.

## Results

### Selected SNPs for T2DM

After ruling out SNPs that did not meet the standard of genome-wide significance (*p* < 5 × 10^−8^) and clumping for those in linkage disequilibrium (*r*^2^ < 0.001), 143 SNPs were finally selected for T2DM in our study. Besides, the F-statistic of these SNPs were larger than 10 indicating our results were less likely to be biased by weak instruments (29).

### Total Effect of T2DM on CVD

**Figure 1:**
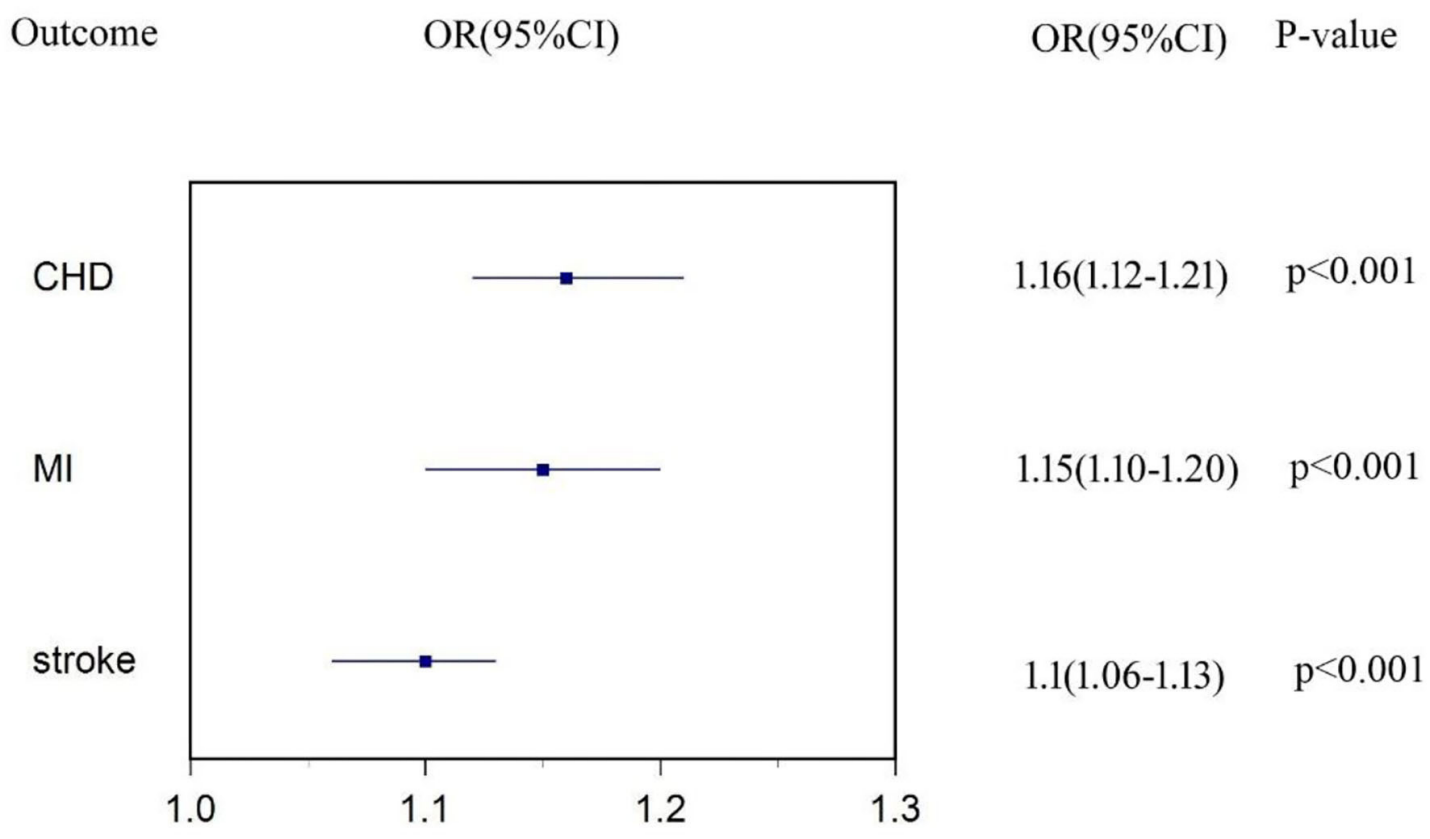
Estimates of causal effect of type 2 diabetes (T2DM) on each subtype of cardiovascular disease (CVD).

We found strong evidence supporting the causality of T2DM on subtypes of CVD. Figure 1 shows the total effect of T2DM on CHD, MI, and stroke. One-unit higher log odds of T2DM increased 16% risk of CHD (OR: 1.16, 95% CI 1.12-1.21, *p* < 0.001), 15% risk of MI (OR: 1.15, 95% CI 1.10–1.20, *p* < 0.001), and 10% risk of stroke (OR: 1.10, 95% CI 1.06–1.13, *p* < 0.001). Details of genetic associations of T2DM on CVD were shown in Supplementary Tables 15–17.

### Effect of T2DM on the Cardiometabolic Factors

**Figure 2:**
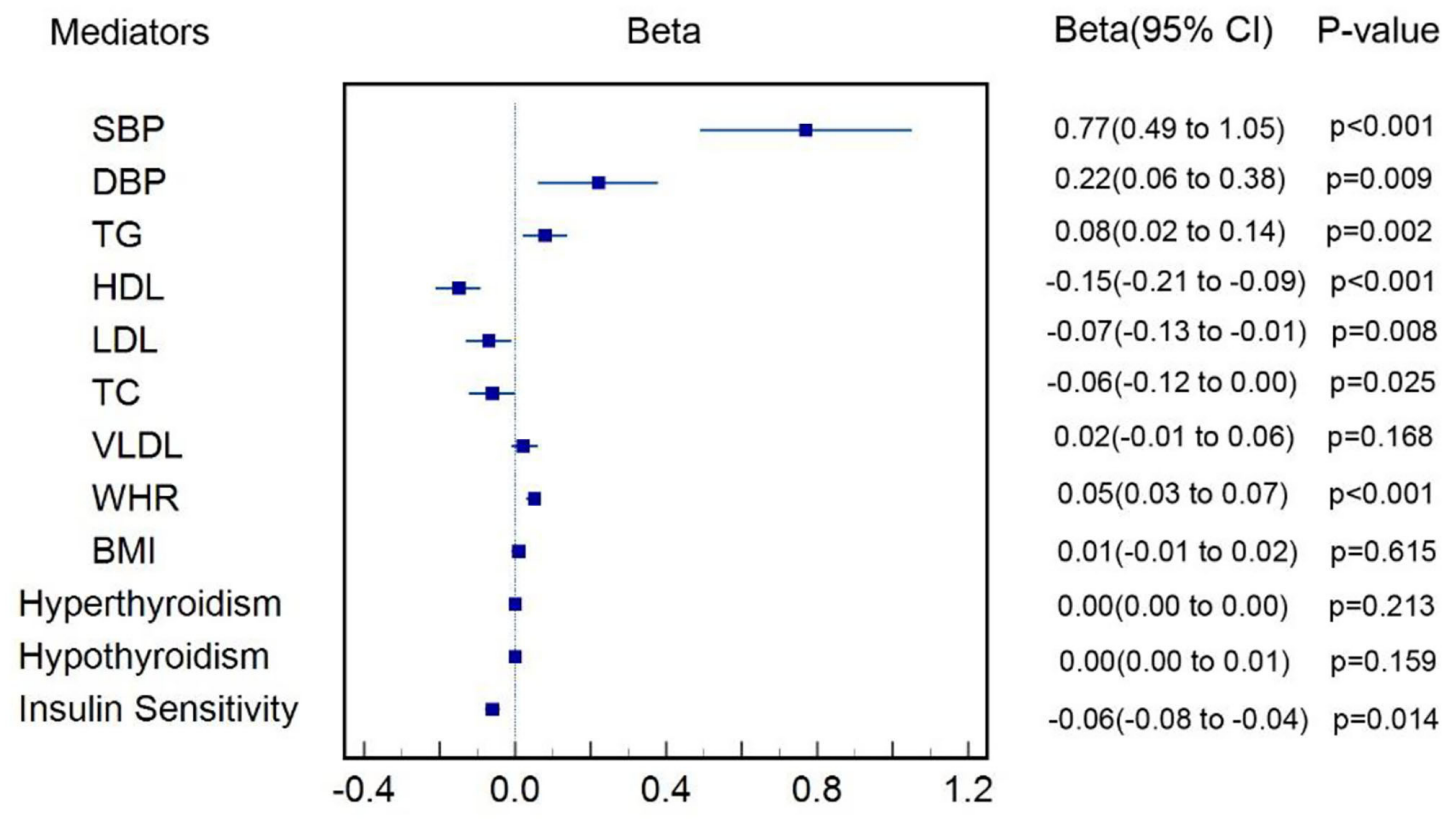
Estimate of causal effect of T2DM on cardiometabolic factors.

Figure 2 shows that one-unit higher log odds of T2DM was associated with increased SD of SBP (β = 0.77, 95% CI: 0.49–1.04, *p* < 0.001), DBP (β = 0.22, 95% CI: 0.06–0.38, *p* = 0.009), TG (β = 0.08, 95% CI: 0.02–0.14, *p* = 0.002), and WHR (β = 0.05, 95% CI: 0.03–0.07, *p* < 0.001), and was also associated with decreased SD of LDL (β = −0.07, 95% CI: −0.12−0.01, *p* = 0.008), HDL (β = −0.15, 95% CI: −0.21−0.09, *p* < 0.001), TC (β = −0.06, 95% CI: −0.12 to −0.00, *p* = 0.025), and insulin sensitivity (β = −0.31, 95% CI: −0.55−0.07, *p* = 0.012). All the aforementioned cardiometabolic factors also survived Bonferroni correction. We failed to find causal effect of T2DM on BMI (*p* = 0.615), VLDL (*p* = 0.168), hyperthyroidism (*p* = 0.213), and hypothyroidism (*p* = 0.159). Genetic associations of T2DM on each cardiometabolic factor were shown in Supplementary Tables 18–29 and those MR results aforementioned were shown in details in Supplementary Table 30.

### Effects of Cardiometabolic Factors on CVD

**Figure 3:**
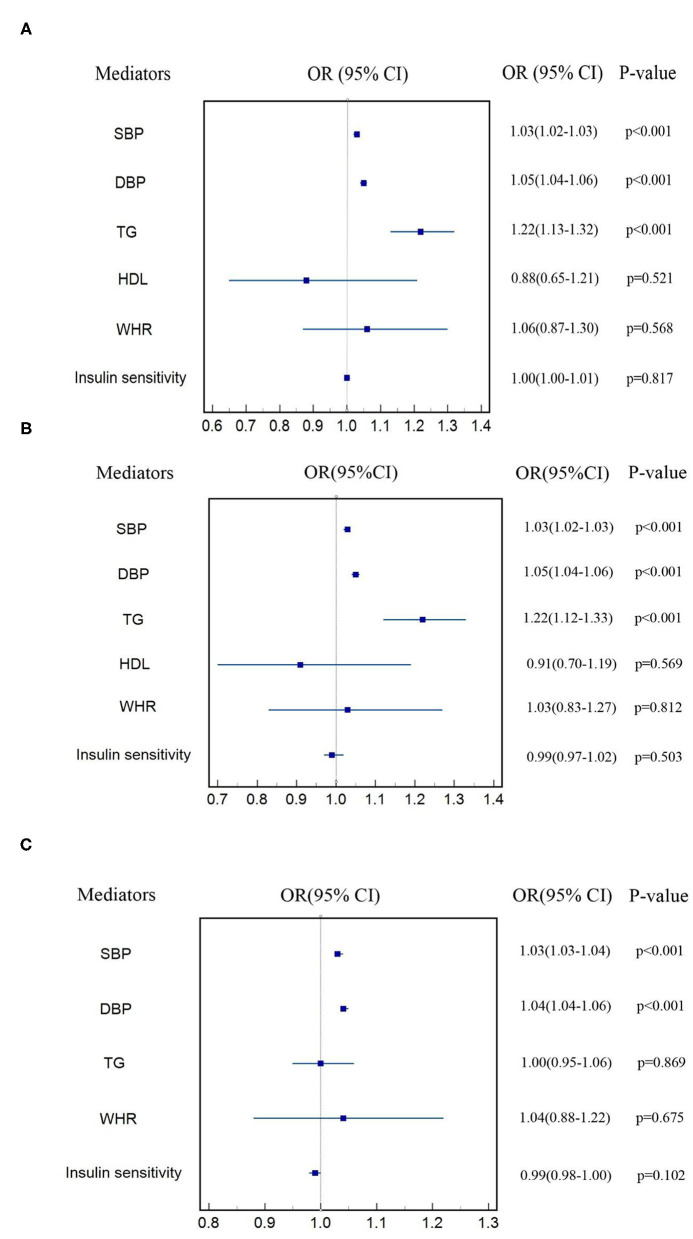
Estimate of effect of cardiometabolic factors on each subtype of CVD **(A)** CHD, **(B)** MI, **(C)** stroke.

Figure 3 shows the estimate of causal effect of one SD increase in each cardiometabolic factor on each subtype of CVD after adjusting for T2DM. Estimate of log odds of CHD for one SD increase in SBP, DBP, TG, HDL, WHR, and insulin sensitivity was 1.03 (95% CI: 1.02–1.04, *p* < 0.001), 1.05 (95% CI: 1.04–1.06, *p* < 0.001), 1.22 (95% CI: 1.13–1.32, *p* < 0.001), 0.89 (95% CI: 0.65–1.21, *p* = 0.521), 1.06 (95% CI: 0.87–1.30, *p* = 0.568), and 1.00 (95% CI: 0.98–1.01, *p* = 0.817), respectively. The estimated OR of MI for genetically determined one SD increase in SBP, DBP, TG, HDL, WHR, and insulin sensitivity was 1.03 (95% CI: 1.02–1.03, *p* < 0.001), 1.05 (95% CI: 1.04–1.06, *p* < 0.001), 1.22 (95% CI: 1.12–1.33, *p* < 0.001), 0.91 (95% CI: 0.70–1.19, *p* = 0.569), 1.03 (95% CI: 0.83–1.27, *p* = 0.812), and 0.99 (95% CI: 0.97–1.02, *p* = 0.503), respectively. Likewise, one SD increase in genetically determined SBP, DBP, TG, WHR, and insulin sensitivity was associated with 3% (OR: 1.03, 95% CI: 1.03–1.04, *p* < 0.001), 4% (OR: 1.04, 95% CI: 1.04–1.05, *p* < 0.001), 0.4% (OR: 1.00, 95% CI: 0.95–1.06, *p* = 0.869), 22% (OR: 0.22, 95% CI: 1.19–1.24, *p* = 0.038), 4% (OR: 1.04, 95% CI: 0.88–1.22, *p* = 0.675) higher risk of stroke, and 1% (OR: 0.99, 95% CI: 0.98–1.00, *p* = 0.102) lower risk of stroke, respectively. All the aforementioned positive results also survived Bonferroni correction. Regression-based MVMR failed to be performed to estimate the effect of TC and LDL on subtypes of CVD, HDL on stroke for the sake of inadequate SNPs after adjusting for T2DM. Genetic associations of each cardiometabolic factor on each subtype of CVD after adjusting for T2DM were shown in Supplementary Tables 31–47.

### Mediation Effects of Cardiometabolic Factors on CVD

**Figure 4:**
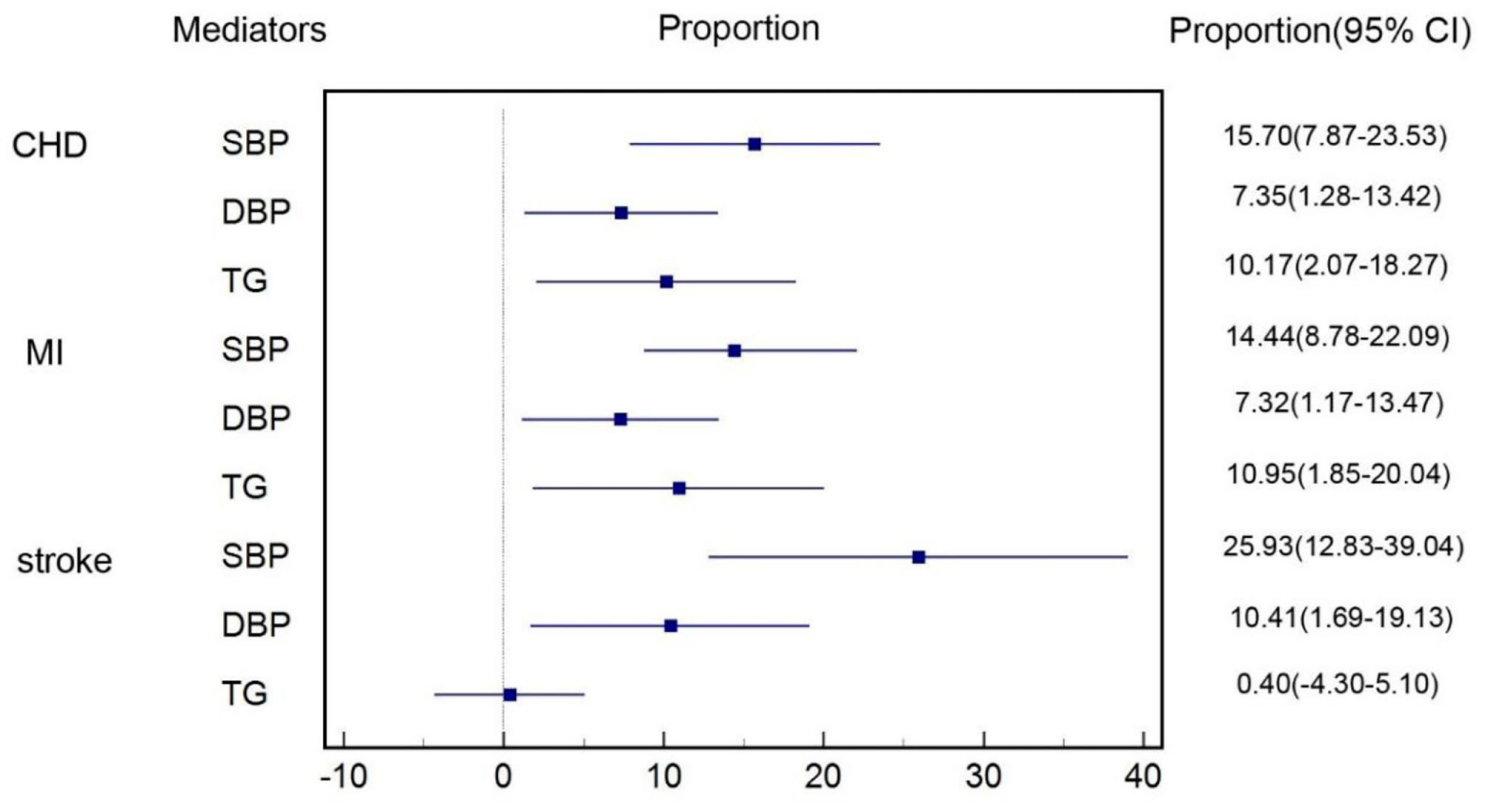
Proportion of the effect of T2DM on each subtype of CVD mediated by cardiometabolic factors.

After excluding cardiometabolic factors that were not causally influenced by T2DM and those that did not have a causal effect on CVD subtypes, we took SBP, DBP, and TG for mediation analysis. Figure 4 shows the proportion of the effect of T2DM on subtypes of CVD mediated by each cardiometabolic factor included in mediation analysis. For the causal effect of T2DM on CHD, the percentage mediated by SBP, DBP, and TG was 16% (8%–24%), 7% (1%–13%), and 10% (2%–18%), respectively. The mediation effect of SBP, DBP, and TG on MI was estimated to account for 14% (7%–22%), 7% (1%–13%), and 11% (2%–20%), respectively. The proportion of the effect of T2DM on stroke mediated by SBP, DBP, and TG was 26% (13%–39%), 10% (2%–19%), and 0.4% (−4%–5%), respectively. Thus, we identified SBP, DBP, and TG were main mediators on CHD and MI, SBP, and DBP also had significant mediation effects on stroke. The total mediation effect of the combination of SBP, DBP, and TG on CHD, MI and stroke was 29%, 26%, and 13%, respectively. Details were shown in Supplementary Tables 48–50.

### Sensitivity Analyses

Part of the results of sensitivity analyses for T2DM on CVD subtypes and three mediators (SBP, DBP, and TG) were shown in Table 1. Egger regression results of T2DM–CHD, T2DM–MI, T2DM–DBP, and T2DM–TG indicated there might be horizontal pleiotropies existing (*p* > 0.05 with nonzero Egger intercept), however, MR-Egger intercepts were close to zero with *p* > 0.05 which indicated such pleiotropies did exist, but their influences were not statistically significant. Besides, results from MR-PRESSO showed that outliers existed in T2DM-CHD, T2DM-stroke, T2DM-SBP, T2DM-DBP, and T2DM-TG. However, *p*-values of the MR-PRESSO distortion test which aimed at testing whether the difference between the causal estimate before and after removal outliers was larger than 0.05 except T2DM-TG and, thus, indicated that influence of these outliers on our results was not statistically significant (Supplementary Tables 51–56). Results of simple median and weighted median were most statistically significant (*p* < 0.05) which indicated that our results were less likely biased by invalid instruments. Other sensitivity analyses including single SNP analysis, leave-one-out analysis both provided consistent results with our main analysis and were shown in Supplementary Tables 57–62.

**Table 1:** MR sensitivity analyses.

**Analysis**	**Nsnp**	**Effect**	**Se**	***P-value***	**OR**
T2DM-CHD					
Simple median	118	0.15	0.02	<0.001	1.16
Weighted median	118	0.11	0.03	<0.001	1.11
MR Egger	118	0.06	0.05	0.267	1.06
MR Egger intercept		0.01	0.00	0.039	
Inverse variance weighted	118	0.15	0.02	<0.001	1.16
T2DM-MI					
Simple median	118	0.15	0.03	<0.001	1.16
Weighted median	118	0.09	0.03	0.001	1.09
MR Egger	118	0.03	0.05	0.499	1.03
MR Egger intercept		0.01	0.00	0.022	
Inverse variance weighted	118	0.14	0.02	<0.001	1.15
T2DM-stroke					
Simple median	118	0.09	0.02	<0.001	1.09
Weighted median	118	0.09	0.02	<0.001	1.09
MR Egger	118	0.08	0.04	0.025	1.09
MR Egger intercept		0.00	0.00	0.817	
Inverse variance weighted	118	0.09	0.02	<0.001	1.10
T2DM-SBP					
Simple median	116	0.65	0.10	<0.001	1.92
Weighted median	116	0.60	0.09	<0.001	1.82
MR Egger	116	0.70	0.32	0.031	2.00
MR Egger intercept		0.01	0.02	0.827	
Inverse variance weighted	116	0.76	0.14	<0.001	2.13
T2DM-DBP					
Simple median	116	0.09	0.06	0.122	1.09
Weighted median	116	0.04	0.05	0.511	1.04
MR Egger	116	0.07	0.19	0.726	1.07
MR Egger intercept		0.01	0.01	0.402	
Inverse variance weighted	116	0.21	0.08	0.012	1.23
T2DM-TG					
Simple median	118	0.07	0.01	<0.001	1.07
Weighted median	118	0.05	0.01	<0.001	1.05
MR Egger	118	0.01	0.06	0.871	1.01
MR Egger intercept		0.01	0.00	0.180	
Inverse variance weighted	118	0.08	0.02	0.002	1.08

*MR, mendelian randomization; Nsnp, number of SNP; OR, odds ratio; T2DM, type 2 diabetes; CHD, coronary heart disease; MI, myocardial infarction; SBP, systolic blood pressure; DBP, diastolic blood pressure; TGs, triglycerides*.

## Discussion

In this large-scale multivariable MR study, we estimated that each additional unit of log odds of T2DM was associated with 16% higher risk for CHD, 15% higher risk for MI, and 10% higher risk for stroke. More importantly, approximately one-third of excess risk for CVD among patients with T2DM was mediated by SBP, DBP, and TG. The most important mediator was elevated SBP, accounting for 16%, 14%, and 26% of the excess risk for CHD, MI, and stroke. Thus, interventions that mitigate these factors might address a substantial proportion of the excess risk of CVD among patients with T2DM.

Previous observational studies have convinced that T2DM was associated with excess CVD risk. A meta-analysis including 698,782 participants from 102 studies concluded that hazard ratios with diabetes were 2.00 (95% CI: 1.83–2.19) for CHD, 2.27 (95% CI: 1.19–2.05) for ischemic stroke (3). Although results were consistent after adjusting for many factors such as sex, smoking status, BMI, etc., observational studies might still be biased by other confounders and measurement error. Thus, diabetes participants included in the meta-analysis aforementioned might combine other confounders which led to higher CVD risk. Compared with observational studies, results from our study were more consistent with previous MR studies which showed that per unit increase in log-odds of T2DM was associated with increased risk of CHD (OR: 1.11, 95% CI: 1.05–1.17) (30), large-artery stroke (OR: 1.28, 95% CI: 1.16–1.40) (31), and coronary artery disease (OR: 1.63, 95% CI: 1.23–2.07) (13). But none of these studies analyzed the causal effect of T2DM on all the three main subtypes of CVD and neither had explored the underlying mechanism.

Our study showed that SBP, DBP, and TG were the main mediators for a causal effect of T2DM on CVD. Many previous observational studies have proved that T2DM was associated with blood pressure (32–34) and TG. Besides, results from another MR study also indicated that T2DM had a causal effect on blood pressure (35). However, few studies have investigated whether these metabolic factors had mediation roles in excess CVD risk of patients with T2DM.

Previous meta-analyses have documented that metabolic syndrome was an important risk factor for CVD (36, 37). An observational study including 1,038,704 participants in China found that among 85,684 participants with one metabolic disorder at baseline, 28.1% developed additional metabolic disorders which were responsible for higher CVD risk, while among participants without metabolic disorder at baseline only 7.9% had new-onset metabolic disorder (38). Our results further showed that the significant causal effect of T2DM on cardiometabolic factors and mediation effects of these metabolic factors on the causal effect of T2DM on CVD, thus might partly elucidate the unexplained mechanism.

We found that the combined mediation effect of three main mediators including SBP, DBP, and TG accounted for 29, 26, 13% of the total effect on CHD, MI, and stroke, implying that intervention on these factors might bring benefit to risk reduction of CVD among patients with T2DM. Although blood pressure has already been recommended as a treatment target to reduce CVD risk among patients with T2DM, opinions on TG were more controversial. Limited by study design or sample size or other factors, clinical trials including the Bezafibrate Infarction Prevention (BIP) (39), the Fenofibrate Intervention and Event Lowering in Diabetes (FIELD) (40), and the Action to Control Cardiovascular Risk in Diabetes Lipid Trial (ACCORD-LIPID) (41) failed to prove fibrate, the TG-reducing drug, could reduce CVD risk among patients with T2DM. Results from our study were in favor of new large-scale RCTs investigating whether TG could be a new treatment target of patients with T2DM to reduce CVD risk and an on-going prospective trial using Pemafibrate is inspiring.

The pathophysiological mechanism of these cardiometabolic factors remains unclear (42). Insulin resistance had an essential role in T2DM (43) that could induce hyperinsulinemia and hyperglycemia, thus led to vasoconstriction and sodium retention and contributed to blood pressure changes ultimately. Besides, its influence on blood lipid profiles progresses toward a prothrombotic and proinflammatory state (44, 45) which largely increases CVD risk.

We failed to find evidence for causal associations of T2DM with VLDL and LDL-C, TC, HDL, and other lipoprotein fractions. We found no MR studies have been conducted to explore these causal associations in the European population, while a small-sized one-sample MR study among 9,798 participants in East China showed that T2DM increased TC and LDL-C levels (46). The discrepancy may be due to ethnic differences, MR methods, and sample size. Importantly, 2016 ESC guidelines mentioned that diabetic dyslipidemia was combined with multiple plasma lipid and lipoprotein abnormalities, and these components were closely linked to each other rather than isolated (47), thus adding difficulties to estimate the causal effect of T2DM on a certain fraction of lipoprotein.

### Strength and Limitations

To the best of our knowledge, this study is the first two-sample MR study to identify the mediation effects of cardiometabolic factors of the causal effect of T2DM on CVD. Many previous clinical trials have tried to prove the mediation role of these factors (48–50), but limited by short-term follow-up, sample size, and most importantly, the influence of confounding factors, thus interpretation of results of these studies became complex. We used a two-sample MR to estimate the causal effect of T2DM on subtypes of CVD. The fact that genetic variants were determined at conception before the onset of diseases allows MR to avoid bias from reverse causation and reduce bias from confounding factors which cannot be ignored in observational studies. Besides, in MR studies, participants were lifelong exposed to alleles, which is far longer than follow-up RCTs. These unique strengths make MR an effective approach to explore the certain causal effect. Compared with one-sample MR, the main advantage of two-sample MR is the increased statistical power because of summary data extracted from different GWAS (51) and less bias caused by pleiotropy because of sensitivity analyses (28). In addition, instrumental variables of T2DM used in our study were obtained from a recently published GWAS. The number of SNPs included in this study was almost three times larger than previous MR studies and explained ~10% of the heritability of T2DM (17), while previous studies only accounted for <5% (13, 30, 31). All the SNPs included have large F-statistics which indicated that our finding was less likely influenced by weak instrument bias. We also applied different MR sensitivity analyses to minimize bias from horizontal pleiotropy or other sources and consistency across these approaches was well evaluated. Finally, we included as many cardiometabolic factors as possible, thus making our results more comprehensive, although we failed to find mediation roles for some cardiometabolic factors.

Horizontal pleiotropy has always been an important source of bias for MR studies and affected ~48% of MR studies (52). We applied sensitivity analyses to detect horizontal pleiotropy including MR-Egger, MR-Egger intercept, and MR-PRESSO. Although the results of MR-Egger and MR-PRESSO showed horizontal pleiotropy, results of MR-Egger intercept and MR-PRESSO distortion test indicated such influence of pleiotropies on our main analysis were not statistically significant. However, for T2DM-TG, the *p*-value for MR-PRESSO distortion test was <0.05, which indicated significant horizontal pleiotropy; thus, it was inconsistent with result of MR-Egger intercept. A plausible explanation of such pleiotropy is that T2DM might affect TG through other cardiometabolic factors that are not included in our study. Thus, we reckon that bias from horizontal pleiotropy was not statistically significant for most of our main analyses, but result of T2DM-TG should be interpreted with caution.

Bidirectional MR was performed for essential results and no effect of genetic predisposition of CHD and MI to T2DM was found. The number of SNP for stroke in this study is rather low; thus, estimate of causality of stroke on T2DM is less precise and previous MR study show that causality between T2DM and stroke is one-direction (T2DM to stroke) (12). Although previous MR studies suggested that blood pressure and TG had causal effect on T2DM (53, 54), genetic instruments for T2DM were rather strong and in a large number in our study, thus, it is less likely that our results are biased because of the reverse causation.

Another limitation of this study is that participants included are mainly European and, thus, generalize our results to Asian or African population should be more cautious. Besides, we used a linear-regression based MVMR method to estimate the individual mediation effect of each cardiometabolic factor. However, there may be interactions between these mediators which make it possible that one mediator may also affect other mediators; thus, resulting in less precise individual effect. Results of our study show that total mediation effect accounted for approximately one third of the total causal effect of T2DM on subtypes of CVD which suggests that there may be some unknown mediators.

## Conclusion

By using a two-step, two-sample MR method, this study provides strong evidence for the causal effect of T2DM on development of CVD, and further suggests that approximately one third of excess risk for CVD among patients with T2DM is mediated through SBP, DBP, and TG, underscoring the importance of large-scale intervention targeting on SBP, DBP, and TG which could reduce substantial proportion of CVD risk among patients with T2DM.

## Data Availability Statement

The original contributions presented in the study are included in the article/Supplementary Material, further inquiries can be directed to the corresponding author.

## Ethics Statement

Written informed consent was obtained from the individual(s) for the publication of any potentially identifiable images or data included in this article.

## Author Contributions

KC and Y-DT designed this study and also took responsibility for the integrity and accuracy of data and analysis in this study. KC wrote the manuscript and performed data analysis in this study. ZZ, CS, JZ, QZ, TH, ED, and Y-DT reviewed and revised the manuscript. All the authors had access to data in this study and contributed to statistical analysis and reviewing the manuscript. KC and Y-DT are the guarantors. All the listed authors meet authorship criteria. All authors contributed to the article and approved the submitted version.

## Funding

This study was supported by the National Natural Science Foundation of China (82070235) and the Natural Science Foundation of Beijing Municipality (7191013).

## Conflict of Interest

The authors declare that the research was conducted in the absence of any commercial or financial relationships that could be construed as a potential conflict of interest. The reviewer, ZW, declared a shared affiliation with the authors KC, CS, JZ, QZ, and Y-DT to the handling editor at time of review.

## Publisher's Note

All claims expressed in this article are solely those of the authors and do not necessarily represent those of their affiliated organizations, or those of the publisher, the editors and the reviewers. Any product that may be evaluated in this article, or claim that may be made by its manufacturer, is not guaranteed or endorsed by the publisher.
