# Supplementary material for: Roles of Cardiometabolic Factors in Mediating the Causal Effect of Type 2 Diabetes on Cardiovascular Diseases: A Two-Step, Two-Sample Multivariable Mendelian Randomization Study
Source: Front Cardiovasc Med. 2022 Feb 24;9:813208. doi: 10.3389/fcvm.2022.813208 (PMC8909643; doi:10.3389/fcvm.2022.813208)
Supplement: Supplementary file 1 [file Data_Sheet_1.doc]

**Supplementary Methods and Tables**

**Contents**

[Supplementary methods 5](#__RefHeading___Toc85138676)

[Reference 7](#__RefHeading___Toc85138677)

[Supplementary Tables 8](#__RefHeading___Toc85138678)

[Table 1: Characteristics of data sources of exposure, risk factors and outcome included in our study. 8](#__RefHeading___Toc85138679)

[Table 2: Characteristics of SNPs used as instrumental variables for T2DM 10](#__RefHeading___Toc85138680)

[Table 3: Characteristics of SNPs used as instrumental variables for SBP 15](#__RefHeading___Toc85138681)

[Table 4: Characteristics of SNPs used as instrumental variables for DBP 33](#__RefHeading___Toc85138682)

[Table 5: Characteristics of SNPs used as instrumental variables for TG 51](#__RefHeading___Toc85138683)

[Table 6: Characteristics of SNPs used as instrumental variables for LDL 60](#__RefHeading___Toc85138684)

[Table 7: Characteristics of SNPs used as instrumental variables for HDL 61](#__RefHeading___Toc85138685)

[Table 8: Characteristics of SNPs used as instrumental variables for TC 61](#__RefHeading___Toc85138686)

[Table 9: Characteristics of SNPs used as instrumental variables for WHR 62](#__RefHeading___Toc85138687)

[Table 10: Characteristics of SNPs used as instrumental variables for BMI 63](#__RefHeading___Toc85138688)

[Table 11: Characteristics of SNPs used as instrumental variables for VLDL 83](#__RefHeading___Toc85138689)

[Table 12: Characteristics of SNPs used as instrumental variables for hyperthyroidism 84](#__RefHeading___Toc85138690)

[Table 13: Characteristics of SNPs used as instrumental variables for hypothyroidism 85](#__RefHeading___Toc85138691)

[Table 14: Characteristics of SNPs used as instrumental variables for insulin sensitivity 88](#__RefHeading___Toc85138692)

[Table 15: Genetic association estimates for the effect of T2DM on CHD. ea=effect allele, gx=T2DM, gy=CHD, se=standard error 89](#__RefHeading___Toc85138693)

[Table 16: Genetic association estimates for the effect of T2DM on MI. ea=effect allele, gx=T2DM, gy=MI, se=standard error 94](#__RefHeading___Toc85138694)

[Table 17: Genetic association estimates for the effect of T2DM on stroke. ea=effect allele, gx=T2DM, gy=stroke, gz=SBP, se=standard error 99](#__RefHeading___Toc85138695)

[Table 18: Genetic association estimates for the effect of T2DM on SBP. ea=effect allele, gx=T2DM, gy=SBP, se=standard error 104](#__RefHeading___Toc85138696)

[Table 19: Genetic association estimates for the effect of T2DM on DBP. ea=effect allele, gx=T2DM, gy=DBP, se=standard error 108](#__RefHeading___Toc85138697)

[Table 20: Genetic association estimates for the effect of T2DM on TG. ea=effect allele, gx=T2DM, gy=TG, se=standard error 113](#__RefHeading___Toc85138698)

[Table 21: Genetic association estimates for the effect of T2DM on LDL. ea=effect allele, gx=T2DM, gy=LDL, se=standard error 118](#__RefHeading___Toc85138699)

[Table 22: Genetic association estimates for the effect of T2DM on HDL. ea=effect allele, gx=T2DM, gy=HDL, se=standard error 122](#__RefHeading___Toc85138700)

[Table 23: Genetic association estimates for the effect of T2DM on TC. ea=effect allele, gx=T2DM, gy=TC, se=standard error 126](#__RefHeading___Toc85138701)

[Table 24: Genetic association estimates for the effect of T2DM on WHR. ea=effect allele, gx=T2DM, gy=WHR, se=standard error 130](#__RefHeading___Toc85138702)

[Table 25: Genetic association estimates for the effect of T2DM on BMI. ea=effect allele, gx=T2DM, gy=BMI, se=standard error 135](#__RefHeading___Toc85138703)

[Table 26: Genetic association estimates for the effect of T2DM on VLDL. ea=effect allele, gx=T2DM, gy=VLDL, se=standard error 139](#__RefHeading___Toc85138704)

[Table 27: Genetic association estimates for the effect of T2DM on hyperthyroidism. ea=effect allele, gx=T2DM, gy=hyperthyroidism, se=standard error 144](#__RefHeading___Toc85138705)

[Table 28: Genetic association estimates for the effect of T2DM on hypothyroidism. ea=effect allele, gx=T2DM, gy=hypothyroidism, se=standard error 148](#__RefHeading___Toc85138706)

[Table 29: Genetic association estimates for the effect of T2DM on insulin sensitivity. ea=effect allele, gx=T2DM, gy=insulin sensitivity, se=standard error 153](#__RefHeading___Toc85138707)

[Table 30: MR estimates of T2DM on each risk factor. 158](#__RefHeading___Toc85138708)

[Table 31: Genetic association estimates for the effect of SBP on CHD, adjusted for T2DM. ea=effect allele, gx=SBP, gy=CHD, gz=T2DM, se=standard error 160](#__RefHeading___Toc85138709)

[Table 32: Genetic association estimates for the effect of DBP on CHD, adjusted for T2DM. ea=effect allele, gx=DBP, gy=CHD, gz=T2DM, se=standard error 174](#__RefHeading___Toc85138710)

[Table 33: Genetic association estimates for the effect of TG on CHD, adjusted for T2DM. ea=effect allele, gx=TG, gy=CHD, gz=T2DM, se=standard error 189](#__RefHeading___Toc85138711)

[Table 34: Genetic association estimates for the effect of HDL on CHD, adjusted for T2DM. ea=effect allele, gx=HDL, gy=CHD, gz=T2DM, se=standard error 195](#__RefHeading___Toc85138712)

[Table 35: Genetic association estimates for the effect of WHR on CHD, adjusted for T2DM. ea=effect allele, gx=WHR, gy=CHD, gz=T2DM, se=standard error 195](#__RefHeading___Toc85138713)

[Table 36: Genetic association estimates for the effect of insulin sensitivity on CHD, adjusted for T2DM. ea=effect allele, gx=insulin sensitivity, gy=CHD, gz=T2DM, se=standard error 197](#__RefHeading___Toc85138714)

[Table 37: Genetic association estimates for the effect of SBP on MI, adjusted for T2DM. ea=effect allele, gx=SBP, gy=MI, gz=T2DM, se=standard error 197](#__RefHeading___Toc85138715)

[Table 38: Genetic association estimates for the effect of DBP on MI, adjusted for T2DM. ea=effect allele, gx=DBP, gy=MI, gz=T2DM, se=standard error 212](#__RefHeading___Toc85138716)

[Table 39: Genetic association estimates for the effect of TG on MI, adjusted for T2DM. ea=effect allele, gx=TG, gy=MI, gz=T2DM, se=standard error 226](#__RefHeading___Toc85138717)

[Table 40: Genetic association estimates for the effect of HDL on MI, adjusted for T2DM. ea=effect allele, gx=HDL, gy=MI, gz=T2DM, se=standard error 232](#__RefHeading___Toc85138718)

[Table 41: Genetic association estimates for the effect of WHR on MI, adjusted for T2DM. ea=effect allele, gx=WHR, gy=MI, gz=T2DM, se=standard error 233](#__RefHeading___Toc85138719)

[Table 42: Genetic association estimates for the effect of insulin sensitivity on MI, adjusted for T2DM. ea=effect allele, gx=insulin sensitivity, gy=MI, gz=T2DM, se=standard error 234](#__RefHeading___Toc85138720)

[Table 43: Genetic association estimates for the effect of SBP on stroke, adjusted for T2DM. ea=effect allele, gx=SBP, gy=stroke, gz=T2DM, se=standard error 235](#__RefHeading___Toc85138721)

[Table 44: Genetic association estimates for the effect of DBP on stroke, adjusted for T2DM. ea=effect allele, gx=DBP, gy=stroke, gz=T2DM, se=standard error 249](#__RefHeading___Toc85138722)

[Table 45: Genetic association estimates for the effect of TG on stroke, adjusted for T2DM. ea=effect allele, gx=TG, gy=stroke, gz=T2DM, se=standard error 264](#__RefHeading___Toc85138723)

[Table 46: Genetic association estimates for the effect of WHR on stroke, adjusted for T2DM. ea=effect allele, gx=WHR, gy=stroke, gz=T2DM, se=standard error 270](#__RefHeading___Toc85138724)

[Table 47: Genetic association estimates for the effect of insulin sensitivity on stroke, adjusted for T2DM. ea=effect allele, gx=insulin sensitivity, gy=stroke, gz=T2DM, se=standard error 271](#__RefHeading___Toc85138725)

[Table 48: Genetic association estimates for the effect of T2DM on CHD, adjusted for SBP, DBP and TG. ea=effect allele, gx=T2DM, gy=CHD, ga=SBP, gb=DBP, gc=TG, se=standard error 272](#__RefHeading___Toc85138726)

[Table 49: Genetic association estimates for the effect of T2DM on MI, adjusted for SBP,DBP and TG. ea=effect allele, gx=T2DM, gy=MI, ga=SBP, gb=DBP, gc=TG, se=standard error 277](#__RefHeading___Toc85138727)

[Table 50: Genetic association estimates for the effect of T2DM on stroke, adjusted for SBP,DBP and TG. ea=effect allele, gx=T2DM, gy=stroke, ga=SBP, gb=DBP, gc=TG, se=standard error 281](#__RefHeading___Toc85138728)

[Table 51: MR-PRESSO for estimate of T2DM-CHD. 286](#__RefHeading___Toc85138729)

[Table 52: MR-PRESSO analysis for estimate of T2DM-MI. 286](#__RefHeading___Toc85138730)

[Table 53:MR-PRESSO analysis for estimate of T2DM-stroke. 287](#__RefHeading___Toc85138731)

[Table 54:MR-PRESSO analysis for estimate of T2DM-SBP. 287](#__RefHeading___Toc85138732)

[Table 55:MR-PRESSO analysis for estimate of T2DM-DBP. 287](#__RefHeading___Toc85138733)

[Table 56:MR-PRESSO analysis for estimate of T2DM-TG. 288](#__RefHeading___Toc85138734)

[Table 57: Single SNP analysis for estimate of T2DM-CHD. 288](#__RefHeading___Toc85138735)

[Table 58: Leave-one-out analysis for estimate of T2DM-CHD 293](#__RefHeading___Toc85138736)

[Table 59: Single SNP analysis for estimate of T2DM-MI. 298](#__RefHeading___Toc85138737)

[Table 60: Leave-one-out analysis for estimate of T2DM-MI. 303](#__RefHeading___Toc85138738)

[Table 61. Single SNP analysis for estimate of T2DM-stroke. 307](#__RefHeading___Toc85138739)

[Table 62: Leave-one-out analysis for estimate of T2DM-stroke. 312](#__RefHeading___Toc85138740)

# Supplementary methods

1. Details of MR analyses we used

Inverse variance weighting (IVW). The IVW method is the inverse variance weighted mean of ratio estimates of two instruments [1]. The basic assumption of IVW is that all SNPs used are valid instruments. Invalid instruments are usually caused by influencing the outcome through other pathways which is known as horizontal pleiotropy, and even one single SNP is invalid, the results may be biased because IVW method neither test nor take horizontal pleiotropy into account [2]. Thus we applied simple median, weighted median, MR-Egger regression, MR-PRESSO as sensitivity analyses in order to obtain a robust estimate to horizontal pleiotropy along with IVW method which we will discuss below. However, IVW is a statistically efficient method and more precise than other methods, we applied this method as main analysis in our study.

Simple median estimator and weighted median estimator. As we discussed above, IVW method is only efficient when all genetic variants are valid, otherwise it will be biased. Simple median enjoys a 50% breakdown level which means even up to 50% of genetic variants are invalid, it can still provide consistent estimate of the causal effect [3]. However, simple median is less efficient when each individual estimates’ precision varies largely. Weighted median is used to deal with this problem. Briefly speaking, simple median can be thought of a certain situation that weighted median estimators have equal weights [2]. Standardized weights are given to each genetic variants and thus makes the weighted median method more statistically efficient than the simple median method. Noteworthy, simple median method and the weighted median method both require no more than 50% genetic variants are invalid while the later requires no single genetic variant contributes more than 50% of the weight.

MR-Egger regression. As horizontal pleiotropy is a main source of bias for MR studies, Bowden et al. proposed an alternative method to encounter this, that is, MR-Egger regression [4], which is inspired by a method to assess small-study bias(publication bias) in meta-analysis [5]. MR-Egger regression used a weighted linear regression and if the intercept is zero, which means horizontal pleiotropy does not exist, MR-Egger regression will provide equal estimate as IVW method. In contrast, an intercept differs from zero indicates overall directional pleiotropy. Besides, MR-Egger regression can still provide consistent estimate even all genetic variants are invalid on condition that a weaker assumption called InSIDE (instrument strength independent of direct effect) assumption is satisfied. If pleiotropic effects on the outcome are not all via a certain confounder, then the InSIDE assumption is satisfied, otherwise it is violated.

Mendelian Randomization Pleiotropy RESidual Sum and Outlier (MR-PRESSO). MR-PRESSO method was designed to detect and correct for pervasive horizontal pleiotropy. MR-PRESSO method applies a unique test called ‘outlier detection’ which allows it to work within the framework of IVW, multivariable MR and MR-egger regression [6]. MR-PRESSO method is consisted of three main parts: detection of horizontal pleiotropy, correction of horizontal pleiotropy by removing relevant instrumental variables, testing the significant differences before and after outlier removal. However, there are still certain circumstances that MR-PRESSO can’t completely remove horizontal pleiotropy, such as violation of InSIDE assumption, horizontal pleiotropic variants>50%, other sources of heterogeneity other than horizontal pleiotropy etc.

1. Calculation of mediation effect

We estimated the total causal effect of T2DM on each subtype of CVD and the causal estimated of T2DM on each possible mediator using IVW method discussed above. For individual mediation effect of each mediator, we estimated the indirect effect of each mediator on the outcome after adjusting for T2DM using multivariable MR [7], and then multiplied with the corresponding causal effect of T2DM on each mediator. The proportion of each mediator was calculated through individual mediation effect divided by total effect. And the direct effect of T2DM was estimated using the same multivariable MR method adjusting for each mediator. The combination of mediation effect was calculated through total effect minus the direct effect of T2DM on each subtype of CVD which was estimated using multivariable MR adjusting for all three mediators divided by total effect.

1. Calculation of standard errors

We calculated the mediation effect using both addition or subtraction and multiplication or division, but the standard errors cannot be calculated simply in this way. There are some rules derived from the Gaussian equation for normally-distributed errors.

Addition or subtraction. Suppose Q is combination of sums, i.e. Q = a + b + · · · + c - (x + y + · · · + z), then


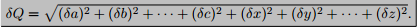


Multiplication or division. Suppose Q=$\frac{\text{ab}\ldots\text{c}}{xy\ldots\text{z}}$, then
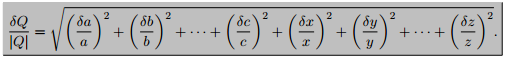


# Reference

[1]. Burgess S, Butterworth A, Thompson SG. Mendelian randomization analysis with multiple genetic variants using summarized data. Genetic epidemiology. 2013;37(7):658-65.

[2]. Bowden J, Davey Smith G, Haycock PC, Burgess S. Consistent Estimation in Mendelian Randomization with Some Invalid Instruments Using a Weighted Median Estimator. Genetic epidemiology. 2016;40(4):304-14.

[3]. Han, C. (2008). Detecting invalid instruments using L1-GMM. Econ. Lett. 101, 285–287. doi: 10.1016/J.ECONLET.2008.09.004

[4]. Bowden J, Davey Smith G, Burgess S. Mendelian randomization with invalid instruments: effect estimation and bias detection through Egger regression. International journal of epidemiology. 2015;44(2):512-25.

[5]. Egger M, Davey Smith G, Schneider M, Minder C. Bias in meta-analysis detected by a simple, graphical test. BMJ (Clinical research ed). 1997;315(7109):629-34.

[6]. Verbanck M, Chen CY, Neale B, Do R. Detection of widespread horizontal pleiotropy in causal relationships inferred from Mendelian randomization between complex traits and diseases. Nature genetics. 2018;50(5):693-8.

[7]. VanderWeele TJ. Mediation Analysis: A Practitioner's Guide. Annual review of public health. 2016;37:17-32.

# Supplementary Tables

We listed genetic variants for T2DM, each subtype of CVD and cardiometabolic disorders. Besides, genetic association estimates between T2DM and subtypes of CVD, T2DM and cardiometabolic disorders, T2DM and each subtype of CVD adjusting for each cardiometabolic disorder individually, cardiometabolic disorders and each subtype of CVD adjusting for T2DM, and T2DM and each subtype of CVD adjusting for all main mediators.

Table 1: Characteristics of data sources of exposure, risk factors and outcome included in our study.

| Trait | Population | Sample size | year |
| --- | --- | --- | --- |
| T2DM | European | 659316 | 2018 |
| CHD | 77% European* | 184305 | 2015 |
| MI | 77% European* | 171875 | 2015 |
| stroke | Multiancestry# | 446696 | 2018 |
| SBP | European | >1 million | 2018 |
| DBP | European | >1 million | 2018 |
| HDL | European | 9796 | 2017 |
| LDL | European | 9961 | 2017 |
| TC | European | 9817 | 2017 |
| VLDL | European | 24925 | 2016 |
| WHR | European | 224459 | 2015 |
| BMI | European | 700000 | 2018 |
| Hyperthyroidism | European | 462933 | 2018 |
| Hypothyroidism | European | 337159 | 2017 |
| Insulin sensitivity | European | 16753 | 2016 |

*: Data source for CHD and MI was from a meta-analysis including 48 GWAS thus resulting population mixed (77% from European, 13% and 6% from south and east Asian, others from Hispanic or African American).

#: The number of studies with European ancestry, African ancestry, Asian ancestry and Latin American population GWAS studies were 17, 5, 6 and 1 subjectively.

SNPs for TG were extracted from an online public GWAS provided by Neale lab including 13586007 SNPs from European ancestry participants in 2018. We could not find an article describing those results, but they can be obtained through <http://gwas-api.mrcieu.ac.uk/> using R 4.0.3 software TwoSampleMR package.

## Table 2: Characteristics of SNPs used as instrumental variables for T2DM

|  | SNP | effect_allele | other_allele | EAF.exposure | effect | SE | p-value | F-statistic |
| --- | --- | --- | --- | --- | --- | --- | --- | --- |
| 1 | rs1127655 | T | C | 0.53 | -0.04 | 0.01 | 2.47E-08 | 30.74 |
| 2 | rs2296173 | G | A | 0.21 | 0.07 | 0.01 | 7.66E-14 | 55.82 |
| 3 | rs12088739 | G | A | 0.09 | -0.09 | 0.01 | 9.79E-12 | 46.24 |
| 4 | rs340874 | C | T | 0.56 | 0.06 | 0.01 | 8.41E-18 | 73.54 |
| 5 | rs2820426 | G | A | 0.61 | 0.05 | 0.01 | 1.30E-12 | 50.94 |
| 6 | rs2493394 | G | A | 0.11 | 0.07 | 0.01 | 1.15E-10 | 41.73 |
| 7 | rs348330 | A | G | 0.63 | -0.05 | 0.01 | 1.86E-09 | 36.15 |
| 8 | rs2867125 | C | T | 0.83 | 0.06 | 0.01 | 4.33E-10 | 39.19 |
| 9 | rs17334919 | T | C | 0.10 | -0.14 | 0.01 | 6.69E-28 | 119.29 |
| 10 | rs13389219 | T | C | 0.39 | -0.07 | 0.01 | 2.11E-22 | 95.19 |
| 11 | rs2972144 | G | A | 0.65 | 0.09 | 0.01 | 2.55E-34 | 148.19 |
| 12 | rs840967 | A | C | 0.61 | -0.05 | 0.01 | 5.44E-10 | 38.60 |
| 13 | rs243019 | C | T | 0.46 | 0.06 | 0.01 | 2.29E-15 | 63.55 |
| 14 | rs7561798 | G | A | 0.48 | 0.04 | 0.01 | 2.79E-08 | 30.86 |
| 15 | rs780094 | C | T | 0.61 | 0.07 | 0.01 | 5.16E-21 | 87.45 |
| 16 | rs12617659 | T | C | 0.15 | -0.07 | 0.01 | 2.83E-11 | 44.23 |
| 17 | rs7572970 | G | A | 0.72 | 0.06 | 0.01 | 1.39E-11 | 45.99 |
| 18 | rs4686471 | C | T | 0.61 | 0.05 | 0.01 | 4.28E-11 | 43.46 |
| 19 | rs6795735 | T | C | 0.41 | -0.06 | 0.01 | 1.63E-14 | 58.43 |
| 20 | rs9844972 | C | G | 0.07 | 0.10 | 0.01 | 1.03E-10 | 41.72 |
| 21 | rs1899951 | T | C | 0.12 | -0.11 | 0.01 | 1.64E-24 | 105.20 |
| 22 | rs11708067 | G | A | 0.24 | -0.10 | 0.01 | 5.93E-29 | 125.91 |
| 23 | rs11926707 | C | T | 0.63 | 0.05 | 0.01 | 1.69E-08 | 31.88 |
| 24 | rs2292662 | T | C | 0.15 | -0.06 | 0.01 | 1.24E-08 | 32.11 |
| 25 | rs7619041 | A | T | 0.51 | -0.04 | 0.01 | 2.76E-08 | 30.53 |
| 26 | rs7651090 | G | A | 0.31 | 0.12 | 0.01 | 3.85E-57 | 250.97 |
| 27 | rs1496653 | G | A | 0.20 | -0.08 | 0.01 | 2.57E-18 | 76.36 |
| 28 | rs11925227 | A | G | 0.18 | -0.05 | 0.01 | 2.25E-08 | 31.60 |
| 29 | rs1801214 | T | C | 0.60 | 0.09 | 0.01 | 5.52E-34 | 148.91 |
| 30 | rs11098676 | C | T | 0.79 | 0.05 | 0.01 | 2.03E-08 | 31.64 |
| 31 | rs17086692 | T | G | 0.31 | -0.05 | 0.01 | 2.48E-08 | 30.91 |
| 32 | rs7674212 | T | G | 0.41 | -0.05 | 0.01 | 6.18E-10 | 38.44 |
| 33 | rs7685296 | T | C | 0.28 | -0.05 | 0.01 | 2.32E-10 | 39.80 |
| 34 | rs993380 | G | A | 0.67 | -0.05 | 0.01 | 4.59E-10 | 39.18 |
| 35 | rs735949 | C | T | 0.14 | -0.07 | 0.01 | 1.95E-11 | 44.99 |
| 36 | rs1061813 | A | G | 0.54 | -0.04 | 0.01 | 3.37E-09 | 34.54 |
| 37 | rs4865796 | A | G | 0.69 | 0.05 | 0.01 | 1.33E-11 | 46.17 |
| 38 | rs459193 | G | A | 0.75 | 0.07 | 0.01 | 8.81E-18 | 73.38 |
| 39 | rs6878122 | A | G | 0.68 | -0.06 | 0.01 | 1.19E-12 | 50.97 |
| 40 | rs10077431 | A | C | 0.21 | -0.05 | 0.01 | 4.75E-08 | 29.94 |
| 41 | rs7729395 | T | C | 0.05 | 0.14 | 0.02 | 1.10E-17 | 73.64 |
| 42 | rs2246618 | T | C | 0.31 | 0.05 | 0.01 | 1.20E-09 | 37.30 |
| 43 | rs72892910 | T | G | 0.17 | 0.06 | 0.01 | 6.43E-11 | 42.84 |
| 44 | rs3756784 | G | T | 0.19 | 0.05 | 0.01 | 2.59E-08 | 30.80 |
| 45 | rs1050226 | G | A | 0.41 | -0.05 | 0.01 | 3.34E-11 | 44.03 |
| 46 | rs7756992 | G | A | 0.27 | 0.13 | 0.01 | 6.00E-62 | 276.50 |
| 47 | rs9369425 | A | G | 0.71 | -0.05 | 0.01 | 1.13E-10 | 41.26 |
| 48 | rs1063355 | G | T | 0.60 | 0.07 | 0.01 | 3.72E-19 | 80.54 |
| 49 | rs853974 | C | T | 0.74 | -0.06 | 0.01 | 7.86E-12 | 46.64 |
| 50 | rs622217 | C | T | 0.48 | -0.05 | 0.01 | 3.13E-10 | 39.67 |
| 51 | rs2191348 | T | G | 0.55 | 0.07 | 0.01 | 3.44E-19 | 79.77 |
| 52 | rs17168486 | T | C | 0.17 | 0.07 | 0.01 | 2.18E-15 | 62.31 |
| 53 | rs849135 | A | G | 0.50 | -0.10 | 0.01 | 1.04E-43 | 192.52 |
| 54 | rs2908282 | A | G | 0.18 | 0.06 | 0.01 | 4.25E-09 | 34.48 |
| 55 | rs2299383 | T | C | 0.42 | 0.04 | 0.01 | 1.49E-08 | 31.85 |
| 56 | rs13234269 | A | T | 0.49 | -0.06 | 0.01 | 6.98E-14 | 55.87 |
| 57 | rs7786095 | G | A | 0.10 | -0.07 | 0.01 | 9.64E-09 | 33.17 |
| 58 | rs13239186 | T | C | 0.30 | 0.05 | 0.01 | 2.70E-10 | 40.21 |
| 59 | rs10087241 | A | G | 0.59 | -0.05 | 0.01 | 2.80E-09 | 35.25 |
| 60 | rs7845219 | C | T | 0.49 | -0.04 | 0.01 | 4.54E-09 | 34.35 |
| 61 | rs516946 | C | T | 0.76 | 0.08 | 0.01 | 3.16E-22 | 93.98 |
| 62 | rs3802177 | A | G | 0.31 | -0.12 | 0.01 | 2.32E-52 | 231.42 |
| 63 | rs10100265 | C | A | 0.61 | -0.05 | 0.01 | 6.29E-10 | 38.63 |
| 64 | rs17411031 | G | C | 0.26 | -0.05 | 0.01 | 3.04E-08 | 30.86 |
| 65 | rs2294120 | G | A | 0.46 | -0.04 | 0.01 | 1.62E-08 | 31.45 |
| 66 | rs10974438 | C | A | 0.35 | 0.06 | 0.01 | 3.01E-15 | 62.09 |
| 67 | rs10114341 | C | T | 0.44 | -0.04 | 0.01 | 1.15E-08 | 32.27 |
| 68 | rs17791483 | G | A | 0.06 | -0.10 | 0.01 | 3.42E-12 | 48.15 |
| 69 | rs1758632 | G | C | 0.62 | 0.05 | 0.01 | 1.36E-09 | 36.74 |
| 70 | rs10811661 | C | T | 0.17 | -0.16 | 0.01 | 4.13E-58 | 256.33 |
| 71 | rs1333039 | C | G | 0.60 | 0.05 | 0.01 | 5.64E-13 | 52.07 |
| 72 | rs2796441 | A | G | 0.42 | -0.07 | 0.01 | 1.96E-22 | 95.93 |
| 73 | rs10740322 | A | G | 0.69 | 0.05 | 0.01 | 2.11E-08 | 31.49 |
| 74 | rs11257655 | T | C | 0.21 | 0.07 | 0.01 | 1.97E-17 | 71.76 |
| 75 | rs753270 | C | T | 0.58 | 0.05 | 0.01 | 2.70E-11 | 44.67 |
| 76 | rs7923866 | T | C | 0.38 | -0.10 | 0.01 | 9.34E-40 | 172.53 |
| 77 | rs4506565 | T | A | 0.31 | 0.28 | 0.01 | 1.00E-200 | 1389.80 |
| 78 | rs1552224 | C | A | 0.15 | -0.10 | 0.01 | 8.64E-25 | 104.81 |
| 79 | rs67232546 | T | C | 0.21 | 0.06 | 0.01 | 4.66E-10 | 38.54 |
| 80 | rs10830963 | G | C | 0.28 | 0.09 | 0.01 | 5.85E-30 | 129.11 |
| 81 | rs2237892 | T | C | 0.06 | -0.10 | 0.02 | 8.75E-10 | 37.39 |
| 82 | rs5215 | T | C | 0.64 | -0.07 | 0.01 | 2.09E-20 | 86.26 |
| 83 | rs7929543 | C | A | 0.08 | 0.08 | 0.01 | 2.20E-09 | 36.00 |
| 84 | rs2261181 | T | C | 0.10 | 0.10 | 0.01 | 9.18E-17 | 69.68 |
| 85 | rs7138300 | T | C | 0.56 | -0.04 | 0.01 | 5.65E-10 | 37.86 |
| 86 | rs12299509 | G | A | 0.48 | 0.05 | 0.01 | 2.09E-10 | 40.92 |
| 87 | rs11107116 | T | G | 0.22 | 0.05 | 0.01 | 3.75E-08 | 30.19 |
| 88 | rs10842994 | T | C | 0.20 | -0.08 | 0.01 | 1.02E-16 | 68.84 |
| 89 | rs61953351 | T | G | 0.25 | -0.07 | 0.01 | 1.98E-14 | 59.17 |
| 90 | rs825476 | T | C | 0.58 | 0.05 | 0.01 | 6.80E-13 | 51.52 |
| 91 | rs576674 | A | G | 0.83 | -0.07 | 0.01 | 1.79E-11 | 45.46 |
| 92 | rs963740 | T | A | 0.29 | -0.05 | 0.01 | 2.23E-08 | 31.02 |
| 93 | rs1359790 | A | G | 0.29 | -0.08 | 0.01 | 2.80E-23 | 99.00 |
| 94 | rs7144011 | T | G | 0.22 | 0.05 | 0.01 | 1.64E-08 | 32.16 |
| 95 | rs7177055 | A | G | 0.72 | 0.06 | 0.01 | 2.75E-16 | 67.07 |
| 96 | rs6494307 | G | C | 0.43 | -0.04 | 0.01 | 1.67E-08 | 32.26 |
| 97 | rs982077 | G | A | 0.57 | -0.05 | 0.01 | 2.58E-10 | 39.59 |
| 98 | rs12910825 | G | A | 0.36 | 0.05 | 0.01 | 2.16E-12 | 48.81 |
| 99 | rs9940149 | A | G | 0.18 | -0.06 | 0.01 | 9.29E-10 | 37.27 |
| 100 | rs13330951 | G | A | 0.49 | -0.05 | 0.01 | 1.54E-08 | 31.69 |
| 101 | rs77258096 | A | C | 0.10 | -0.12 | 0.01 | 1.78E-18 | 76.37 |
| 102 | rs2925979 | C | T | 0.70 | -0.05 | 0.01 | 9.06E-12 | 46.87 |
| 103 | rs7185735 | G | A | 0.40 | 0.11 | 0.01 | 1.59E-47 | 209.26 |
| 104 | rs17405722 | A | G | 0.07 | 0.09 | 0.01 | 2.28E-09 | 35.51 |
| 105 | rs8068804 | A | G | 0.33 | 0.06 | 0.01 | 4.41E-14 | 56.64 |
| 106 | rs12945601 | C | T | 0.61 | -0.05 | 0.01 | 1.72E-09 | 36.00 |
| 107 | rs9894220 | G | A | 0.43 | -0.06 | 0.01 | 1.52E-13 | 54.83 |
| 108 | rs17631783 | T | C | 0.26 | -0.05 | 0.01 | 3.95E-08 | 29.94 |
| 109 | rs7240767 | C | T | 0.38 | 0.05 | 0.01 | 2.16E-08 | 31.00 |
| 110 | rs12970134 | A | G | 0.27 | 0.06 | 0.01 | 5.31E-12 | 48.13 |
| 111 | rs10401969 | C | T | 0.08 | 0.09 | 0.01 | 4.13E-12 | 47.95 |
| 112 | rs8108269 | G | T | 0.28 | 0.06 | 0.01 | 3.11E-16 | 66.45 |
| 113 | rs6515236 | C | A | 0.25 | -0.05 | 0.01 | 3.34E-08 | 30.67 |
| 114 | rs6066138 | A | G | 0.28 | -0.05 | 0.01 | 1.93E-09 | 35.71 |
| 115 | rs6059662 | G | A | 0.66 | 0.04 | 0.01 | 1.51E-08 | 31.87 |
| 116 | rs4810426 | T | C | 0.10 | 0.07 | 0.01 | 2.15E-08 | 31.19 |
| 117 | rs16988333 | G | A | 0.09 | -0.07 | 0.01 | 9.17E-09 | 32.84 |
| 118 | rs4823182 | G | A | 0.34 | 0.05 | 0.01 | 3.36E-10 | 39.18 |

EAF: Effect allele frequency; T2DM: Type-2 diabetes

## Table 3: Characteristics of SNPs used as instrumental variables for SBP

|  | SNP | effect_allele.exposure | other_allele.exposure | EAF.exposure | effect | SE | p-value | F-Statistic |
| --- | --- | --- | --- | --- | --- | --- | --- | --- |
| 1 | rs7796 | G | C | 0.49 | -0.34 | 0.03 | 5.00E-27 | 116.21 |
| 2 | rs2493296 | T | C | 0.14 | 0.42 | 0.04 | 3.14E-21 | 89.56 |
| 3 | rs488834 | T | C | 0.76 | -0.38 | 0.04 | 2.35E-25 | 108.33 |
| 4 | rs75461554 | T | C | 0.20 | -0.30 | 0.04 | 1.18E-15 | 64.00 |
| 5 | rs404100 | T | C | 0.45 | 0.19 | 0.03 | 1.68E-10 | 40.78 |
| 6 | rs1209384 | G | A | 0.61 | -0.26 | 0.03 | 2.85E-16 | 66.79 |
| 7 | rs11585169 | A | T | 0.58 | 0.18 | 0.03 | 5.34E-09 | 34.00 |
| 8 | rs12731646 | T | C | 0.41 | -0.19 | 0.03 | 7.21E-10 | 37.90 |
| 9 | rs4651224 | T | C | 0.45 | 0.20 | 0.03 | 9.00E-11 | 42.12 |
| 10 | rs12042924 | C | T | 0.47 | 0.18 | 0.03 | 2.62E-09 | 35.57 |
| 11 | rs2724377 | G | A | 0.47 | -0.19 | 0.03 | 1.29E-10 | 41.45 |
| 12 | rs7555285 | C | G | 0.80 | 0.23 | 0.04 | 1.05E-09 | 37.22 |
| 13 | rs72742507 | T | C | 0.30 | -0.21 | 0.03 | 3.80E-10 | 39.18 |
| 14 | rs708117 | A | G | 0.52 | 0.29 | 0.03 | 1.59E-21 | 90.56 |
| 15 | rs11210029 | G | A | 0.37 | 0.20 | 0.03 | 8.92E-11 | 42.06 |
| 16 | rs778124 | A | G | 0.37 | 0.30 | 0.03 | 1.45E-21 | 90.89 |
| 17 | rs61772592 | G | A | 0.13 | 0.32 | 0.05 | 2.86E-12 | 48.88 |
| 18 | rs12136922 | A | G | 0.49 | 0.20 | 0.03 | 2.69E-11 | 44.46 |
| 19 | rs76719272 | T | C | 0.13 | -0.27 | 0.05 | 2.97E-09 | 35.27 |
| 20 | rs263532 | C | T | 0.42 | -0.18 | 0.03 | 4.72E-09 | 34.30 |
| 21 | rs10779795 | G | A | 0.34 | -0.22 | 0.03 | 7.44E-12 | 46.88 |
| 22 | rs34079867 | T | C | 0.27 | 0.20 | 0.04 | 1.78E-08 | 31.66 |
| 23 | rs7514579 | C | A | 0.23 | -0.22 | 0.04 | 5.45E-10 | 38.61 |
| 24 | rs59980837 | T | G | 0.02 | 1.10 | 0.12 | 3.32E-21 | 89.41 |
| 25 | rs68085857 | T | C | 0.23 | 0.27 | 0.04 | 1.68E-14 | 58.91 |
| 26 | rs4926499 | C | G | 0.83 | 0.30 | 0.04 | 1.33E-11 | 45.82 |
| 27 | rs6699618 | G | C | 0.16 | -0.91 | 0.04 | 1.68E-109 | 494.25 |
| 28 | rs1889785 | A | G | 0.46 | 0.18 | 0.03 | 4.35E-09 | 34.36 |
| 29 | rs12063372 | A | G | 0.38 | 0.20 | 0.03 | 3.86E-10 | 39.12 |
| 30 | rs658780 | G | T | 0.26 | 0.20 | 0.03 | 5.29E-09 | 34.16 |
| 31 | rs10776752 | T | G | 0.08 | 0.82 | 0.06 | 4.61E-46 | 203.21 |
| 32 | rs10914124 | C | T | 0.38 | -0.23 | 0.03 | 6.32E-14 | 56.25 |
| 33 | rs2853736 | G | T | 0.31 | -0.24 | 0.03 | 8.34E-13 | 51.18 |
| 34 | rs1408945 | T | G | 0.42 | -0.32 | 0.03 | 8.33E-26 | 110.53 |
| 35 | rs786923 | T | C | 0.62 | -0.31 | 0.03 | 2.82E-23 | 98.84 |
| 36 | rs11120093 | T | C | 0.41 | -0.18 | 0.03 | 5.13E-09 | 34.07 |
| 37 | rs2493134 | C | T | 0.41 | 0.37 | 0.03 | 1.10E-33 | 146.18 |
| 38 | rs1565440 | A | G | 0.38 | 0.17 | 0.03 | 1.94E-08 | 31.52 |
| 39 | rs13420463 | G | A | 0.23 | -0.31 | 0.04 | 2.72E-18 | 76.22 |
| 40 | rs115262049 | T | A | 0.09 | -0.59 | 0.06 | 1.29E-26 | 113.97 |
| 41 | rs2249105 | G | A | 0.37 | -0.29 | 0.03 | 7.63E-21 | 87.45 |
| 42 | rs10188003 | T | C | 0.39 | 0.19 | 0.03 | 8.80E-10 | 37.62 |
| 43 | rs6731373 | A | G | 0.35 | 0.19 | 0.03 | 4.18E-09 | 34.43 |
| 44 | rs2580350 | A | G | 0.56 | 0.18 | 0.03 | 8.39E-09 | 33.20 |
| 45 | rs11694601 | G | A | 0.40 | 0.19 | 0.03 | 6.41E-10 | 38.17 |
| 46 | rs12694277 | C | T | 0.71 | 0.20 | 0.03 | 1.80E-09 | 36.29 |
| 47 | rs2161967 | G | T | 0.57 | -0.28 | 0.03 | 2.87E-20 | 85.34 |
| 48 | rs139354822 | C | T | 0.03 | -0.61 | 0.10 | 3.51E-10 | 39.34 |
| 49 | rs17760259 | C | T | 0.43 | 0.27 | 0.03 | 2.25E-18 | 76.22 |
| 50 | rs2384063 | T | C | 0.76 | 0.33 | 0.04 | 6.33E-20 | 83.69 |
| 51 | rs1275985 | T | C | 0.61 | -0.54 | 0.03 | 4.73E-69 | 308.64 |
| 52 | rs55944332 | G | A | 0.24 | 0.26 | 0.04 | 1.79E-13 | 54.18 |
| 53 | rs12693982 | T | C | 0.40 | 0.26 | 0.03 | 7.49E-17 | 69.44 |
| 54 | rs4952609 | G | A | 0.26 | -0.21 | 0.03 | 9.60E-10 | 37.47 |
| 55 | rs17257081 | G | A | 0.19 | -0.23 | 0.04 | 6.35E-09 | 33.65 |
| 56 | rs268263 | A | T | 0.75 | 0.59 | 0.04 | 1.77E-63 | 282.96 |
| 57 | rs13412750 | A | G | 0.27 | -0.29 | 0.03 | 2.32E-17 | 71.78 |
| 58 | rs1044822 | T | C | 0.15 | -0.25 | 0.04 | 5.16E-09 | 34.21 |
| 59 | rs12464602 | A | G | 0.62 | -0.24 | 0.03 | 1.02E-14 | 59.85 |
| 60 | rs13016772 | T | C | 0.77 | 0.25 | 0.04 | 1.23E-12 | 50.47 |
| 61 | rs6732123 | C | G | 0.42 | -0.17 | 0.03 | 1.52E-08 | 32.01 |
| 62 | rs4577304 | C | T | 0.48 | 0.18 | 0.03 | 4.99E-09 | 34.23 |
| 63 | rs10207726 | T | C | 0.30 | -0.21 | 0.03 | 8.06E-11 | 42.13 |
| 64 | rs62170470 | C | T | 0.40 | -0.20 | 0.03 | 7.68E-10 | 37.74 |
| 65 | rs3845811 | G | C | 0.43 | 0.29 | 0.03 | 1.88E-21 | 90.65 |
| 66 | rs72847885 | G | A | 0.34 | -0.24 | 0.03 | 3.08E-14 | 57.58 |
| 67 | rs6737318 | G | A | 0.22 | -0.23 | 0.04 | 1.13E-10 | 41.61 |
| 68 | rs55732192 | T | G | 0.09 | -0.34 | 0.05 | 1.15E-10 | 41.54 |
| 69 | rs34727427 | C | T | 0.32 | 0.24 | 0.03 | 4.02E-13 | 52.74 |
| 70 | rs1882212 | G | A | 0.22 | -0.28 | 0.04 | 3.34E-14 | 57.52 |
| 71 | rs3828282 | G | C | 0.57 | -0.19 | 0.03 | 5.29E-09 | 34.10 |
| 72 | rs10804330 | C | T | 0.43 | -0.24 | 0.03 | 1.62E-14 | 59.03 |
| 73 | rs2643826 | T | C | 0.45 | 0.45 | 0.03 | 1.74E-48 | 213.68 |
| 74 | rs6788984 | G | A | 0.14 | -0.30 | 0.04 | 3.81E-12 | 48.19 |
| 75 | rs3772219 | C | A | 0.32 | -0.27 | 0.03 | 3.10E-17 | 71.15 |
| 76 | rs1375564 | T | C | 0.64 | 0.26 | 0.03 | 2.84E-16 | 67.03 |
| 77 | rs12637573 | G | A | 0.53 | 0.17 | 0.03 | 9.95E-09 | 32.85 |
| 78 | rs1199330 | G | A | 0.12 | 0.27 | 0.05 | 1.65E-08 | 31.89 |
| 79 | rs4955575 | C | A | 0.25 | -0.22 | 0.03 | 5.63E-10 | 38.45 |
| 80 | rs13091418 | G | C | 0.33 | 0.22 | 0.03 | 6.15E-12 | 47.25 |
| 81 | rs9869437 | A | C | 0.35 | -0.20 | 0.03 | 3.22E-10 | 39.59 |
| 82 | rs189267552 | A | T | 0.01 | -0.87 | 0.14 | 4.55E-10 | 38.85 |
| 83 | rs743395 | T | C | 0.38 | 0.26 | 0.03 | 2.55E-16 | 67.12 |
| 84 | rs7618284 | C | G | 0.34 | -0.19 | 0.03 | 1.10E-08 | 32.64 |
| 85 | rs4499560 | T | A | 0.68 | 0.22 | 0.03 | 1.46E-11 | 45.50 |
| 86 | rs9857362 | C | A | 0.47 | -0.17 | 0.03 | 1.62E-08 | 31.85 |
| 87 | rs9876694 | T | C | 0.06 | 0.47 | 0.07 | 4.64E-13 | 52.41 |
| 88 | rs3980686 | T | G | 0.11 | -0.50 | 0.05 | 1.03E-24 | 105.33 |
| 89 | rs1290784 | T | C | 0.45 | 0.41 | 0.03 | 2.97E-42 | 185.25 |
| 90 | rs262986 | A | G | 0.47 | -0.24 | 0.03 | 7.67E-15 | 60.43 |
| 91 | rs68115553 | G | A | 0.02 | 0.64 | 0.11 | 1.74E-08 | 31.79 |
| 92 | rs6771917 | C | T | 0.75 | 0.38 | 0.04 | 1.39E-26 | 114.16 |
| 93 | rs7615099 | G | A | 0.33 | -0.19 | 0.03 | 3.90E-09 | 34.70 |
| 94 | rs9880098 | A | G | 0.39 | 0.31 | 0.03 | 1.59E-23 | 100.06 |
| 95 | rs79539362 | C | T | 0.10 | -0.40 | 0.05 | 2.09E-15 | 63.08 |
| 96 | rs6788907 | A | G | 0.27 | 0.22 | 0.03 | 7.31E-11 | 42.50 |
| 97 | rs9848170 | C | G | 0.60 | 0.32 | 0.03 | 7.01E-26 | 110.76 |
| 98 | rs4408839 | G | A | 0.26 | 0.23 | 0.03 | 2.42E-11 | 44.48 |
| 99 | rs11925504 | A | G | 0.57 | -0.29 | 0.03 | 1.78E-21 | 90.47 |
| 100 | rs1052501 | T | C | 0.83 | 0.23 | 0.04 | 4.14E-08 | 30.14 |
| 101 | rs6445583 | A | G | 0.75 | 0.28 | 0.03 | 1.90E-15 | 63.18 |
| 102 | rs6438857 | C | T | 0.42 | -0.27 | 0.03 | 3.13E-19 | 80.47 |
| 103 | rs2111557 | T | C | 0.47 | 0.18 | 0.03 | 5.22E-09 | 34.12 |
| 104 | rs60909079 | C | G | 0.25 | -0.21 | 0.04 | 1.73E-09 | 36.27 |
| 105 | rs72719160 | T | A | 0.32 | 0.22 | 0.03 | 4.34E-12 | 47.93 |
| 106 | rs2610990 | G | A | 0.74 | 0.29 | 0.03 | 2.86E-17 | 71.63 |
| 107 | rs62309747 | A | G | 0.47 | -0.22 | 0.03 | 1.59E-13 | 54.49 |
| 108 | rs1814951 | A | G | 0.88 | -0.32 | 0.05 | 3.91E-12 | 48.07 |
| 109 | rs7439567 | T | C | 0.41 | 0.25 | 0.03 | 2.31E-16 | 67.41 |
| 110 | rs2353940 | C | T | 0.25 | 0.21 | 0.04 | 6.85E-09 | 33.59 |
| 111 | rs12643599 | G | A | 0.36 | -0.31 | 0.03 | 1.23E-23 | 100.26 |
| 112 | rs2498323 | A | G | 0.10 | 0.32 | 0.05 | 8.51E-10 | 37.62 |
| 113 | rs2291434 | T | G | 0.53 | -0.26 | 0.03 | 5.10E-18 | 74.88 |
| 114 | rs12511987 | G | T | 0.18 | 0.23 | 0.04 | 5.39E-09 | 34.07 |
| 115 | rs12509595 | C | T | 0.29 | 0.84 | 0.03 | 2.55E-138 | 627.55 |
| 116 | rs17010957 | C | T | 0.15 | 0.53 | 0.04 | 1.78E-35 | 154.22 |
| 117 | rs73855810 | A | G | 0.14 | 0.27 | 0.04 | 3.04E-10 | 39.63 |
| 118 | rs7683728 | T | C | 0.53 | -0.37 | 0.03 | 2.43E-33 | 144.47 |
| 119 | rs1290933 | A | C | 0.69 | -0.28 | 0.03 | 3.17E-18 | 75.80 |
| 120 | rs55924432 | T | C | 0.40 | 0.27 | 0.03 | 5.70E-17 | 69.94 |
| 121 | rs10028284 | T | A | 0.18 | -0.29 | 0.04 | 1.69E-13 | 54.46 |
| 122 | rs13107325 | T | C | 0.07 | -0.91 | 0.06 | 4.22E-53 | 235.56 |
| 123 | rs1493132 | C | T | 0.34 | 0.18 | 0.03 | 2.73E-08 | 30.84 |
| 124 | rs4834792 | A | T | 0.48 | 0.20 | 0.03 | 7.24E-11 | 42.40 |
| 125 | rs869396 | A | C | 0.47 | -0.21 | 0.03 | 4.12E-12 | 48.09 |
| 126 | rs34535756 | T | C | 0.04 | 0.48 | 0.08 | 1.18E-09 | 36.98 |
| 127 | rs60991988 | G | T | 0.11 | -0.38 | 0.05 | 2.82E-14 | 57.89 |
| 128 | rs13107261 | A | G | 0.37 | -0.18 | 0.03 | 1.57E-08 | 32.06 |
| 129 | rs10008637 | C | T | 0.46 | -0.22 | 0.03 | 9.24E-13 | 51.01 |
| 130 | rs11097909 | C | T | 0.85 | 0.36 | 0.04 | 3.35E-17 | 71.19 |
| 131 | rs17035181 | G | T | 0.14 | -0.31 | 0.04 | 7.61E-13 | 51.34 |
| 132 | rs11241313 | T | C | 0.31 | -0.21 | 0.03 | 2.23E-10 | 40.36 |
| 133 | rs12656497 | C | T | 0.60 | 0.64 | 0.03 | 7.14E-96 | 432.15 |
| 134 | rs7722243 | A | G | 0.50 | -0.20 | 0.03 | 1.21E-11 | 45.99 |
| 135 | rs12657950 | T | C | 0.07 | 0.46 | 0.06 | 1.27E-14 | 59.47 |
| 136 | rs1422279 | T | C | 0.39 | 0.33 | 0.03 | 1.05E-26 | 114.75 |
| 137 | rs10045307 | G | C | 0.23 | 0.20 | 0.04 | 2.21E-08 | 31.26 |
| 138 | rs702395 | T | C | 0.44 | 0.23 | 0.03 | 3.24E-14 | 57.76 |
| 139 | rs1957563 | T | C | 0.27 | 0.36 | 0.03 | 2.32E-26 | 112.60 |
| 140 | rs13358657 | G | A | 0.13 | 0.39 | 0.04 | 2.95E-18 | 76.02 |
| 141 | rs3860770 | A | G | 0.29 | -0.27 | 0.03 | 1.20E-15 | 63.95 |
| 142 | rs246973 | T | C | 0.29 | 0.25 | 0.03 | 1.45E-13 | 54.76 |
| 143 | rs9327297 | G | C | 0.33 | -0.27 | 0.03 | 8.07E-18 | 74.15 |
| 144 | rs10069690 | T | C | 0.26 | 0.31 | 0.04 | 4.47E-17 | 70.49 |
| 145 | rs7725413 | T | C | 0.77 | -0.20 | 0.04 | 3.07E-08 | 30.57 |
| 146 | rs10941043 | G | T | 0.29 | 0.26 | 0.03 | 6.42E-15 | 60.62 |
| 147 | rs6870654 | C | T | 0.25 | -0.21 | 0.03 | 7.58E-10 | 37.89 |
| 148 | rs73103937 | C | T | 0.27 | -0.21 | 0.03 | 2.36E-09 | 35.55 |
| 149 | rs7703560 | G | A | 0.30 | 0.22 | 0.03 | 1.51E-11 | 45.49 |
| 150 | rs6452769 | A | G | 0.21 | -0.31 | 0.04 | 7.82E-17 | 69.50 |
| 151 | rs76443575 | C | G | 0.04 | -0.52 | 0.08 | 1.40E-10 | 41.13 |
| 152 | rs11960210 | C | T | 0.38 | -0.47 | 0.03 | 1.25E-51 | 228.08 |
| 153 | rs12153395 | A | G | 0.11 | -0.33 | 0.05 | 1.07E-11 | 46.19 |
| 154 | rs4957026 | G | A | 0.66 | -0.20 | 0.03 | 8.12E-10 | 37.65 |
| 155 | rs1664781 | A | G | 0.69 | 0.26 | 0.03 | 5.69E-16 | 65.73 |
| 156 | rs28650790 | T | C | 0.19 | 0.23 | 0.04 | 3.30E-09 | 34.92 |
| 157 | rs1871190 | T | G | 0.33 | 0.20 | 0.03 | 1.66E-09 | 36.37 |
| 158 | rs6892983 | A | C | 0.40 | 0.34 | 0.03 | 7.11E-29 | 124.61 |
| 159 | rs2913920 | T | C | 0.77 | 0.24 | 0.04 | 1.62E-11 | 45.37 |
| 160 | rs9349379 | G | A | 0.41 | -0.27 | 0.03 | 1.31E-17 | 72.91 |
| 161 | rs67600122 | T | G | 0.42 | 0.45 | 0.03 | 2.66E-47 | 208.89 |
| 162 | rs1984195 | A | G | 0.49 | 0.24 | 0.03 | 1.77E-15 | 63.21 |
| 163 | rs9486916 | T | C | 0.20 | 0.27 | 0.04 | 5.42E-12 | 47.63 |
| 164 | rs79782817 | T | G | 0.10 | 0.53 | 0.05 | 1.43E-26 | 113.83 |
| 165 | rs13204703 | C | T | 0.25 | -0.20 | 0.04 | 1.94E-08 | 31.58 |
| 166 | rs1293969 | C | T | 0.25 | 0.20 | 0.03 | 1.03E-08 | 32.82 |
| 167 | rs12661036 | C | T | 0.23 | 0.21 | 0.04 | 1.82E-08 | 31.65 |
| 168 | rs2745599 | G | A | 0.45 | -0.22 | 0.03 | 8.96E-12 | 46.60 |
| 169 | rs1630736 | T | C | 0.47 | -0.17 | 0.03 | 3.52E-08 | 30.48 |
| 170 | rs2815063 | A | C | 0.13 | 0.28 | 0.05 | 1.76E-09 | 36.18 |
| 171 | rs10782230 | A | G | 0.48 | 0.21 | 0.03 | 2.91E-12 | 48.63 |
| 172 | rs9401913 | A | G | 0.44 | 0.52 | 0.03 | 3.66E-65 | 290.90 |
| 173 | rs8180684 | T | C | 0.29 | 0.21 | 0.03 | 1.80E-10 | 40.58 |
| 174 | rs2744139 | C | T | 0.24 | -0.24 | 0.04 | 2.48E-11 | 44.53 |
| 175 | rs12528975 | A | G | 0.05 | 0.48 | 0.07 | 1.98E-10 | 40.45 |
| 176 | rs9361836 | T | C | 0.32 | 0.22 | 0.03 | 1.25E-11 | 45.94 |
| 177 | rs961764 | G | C | 0.57 | 0.19 | 0.03 | 3.74E-10 | 39.18 |
| 178 | rs9285476 | G | C | 0.29 | -0.18 | 0.03 | 3.07E-08 | 30.66 |
| 179 | rs1575290 | T | C | 0.47 | 0.20 | 0.03 | 5.59E-11 | 42.97 |
| 180 | rs9368222 | A | C | 0.27 | 0.23 | 0.03 | 1.84E-11 | 45.27 |
| 181 | rs7763558 | A | G | 0.32 | 0.34 | 0.03 | 1.17E-25 | 109.76 |
| 182 | rs78648104 | C | T | 0.09 | 0.43 | 0.05 | 2.36E-15 | 62.79 |
| 183 | rs6921291 | T | C | 0.19 | 0.36 | 0.04 | 1.58E-20 | 86.22 |
| 184 | rs7765526 | G | A | 0.54 | -0.20 | 0.03 | 5.88E-11 | 42.87 |
| 185 | rs17080102 | C | G | 0.07 | -0.81 | 0.06 | 3.52E-42 | 185.26 |
| 186 | rs509833 | G | A | 0.86 | -0.33 | 0.04 | 7.08E-14 | 55.91 |
| 187 | rs7744902 | A | G | 0.08 | -0.41 | 0.06 | 5.64E-12 | 47.52 |
| 188 | rs67617547 | G | C | 0.33 | -0.18 | 0.03 | 2.39E-08 | 31.21 |
| 189 | rs2392929 | G | T | 0.20 | 0.75 | 0.04 | 1.96E-87 | 392.33 |
| 190 | rs34072724 | A | G | 0.49 | -0.24 | 0.03 | 1.37E-15 | 63.89 |
| 191 | rs73049928 | G | A | 0.19 | 0.24 | 0.04 | 1.20E-09 | 36.92 |
| 192 | rs3807925 | G | A | 0.35 | 0.19 | 0.03 | 5.39E-09 | 33.96 |
| 193 | rs12668436 | C | T | 0.25 | 0.22 | 0.04 | 7.88E-10 | 37.77 |
| 194 | rs35680304 | T | C | 0.59 | 0.27 | 0.03 | 3.76E-18 | 75.52 |
| 195 | rs10282122 | T | C | 0.67 | -0.30 | 0.03 | 2.46E-20 | 85.29 |
| 196 | rs200700882 | G | A | 0.40 | 0.23 | 0.03 | 4.22E-14 | 57.17 |
| 197 | rs28688791 | C | T | 0.20 | 0.32 | 0.04 | 2.34E-17 | 71.89 |
| 198 | rs112509803 | C | G | 0.11 | -0.26 | 0.05 | 3.18E-08 | 30.65 |
| 199 | rs6957161 | G | A | 0.74 | -0.21 | 0.03 | 2.20E-09 | 35.79 |
| 200 | rs73727605 | A | G | 0.07 | 0.36 | 0.06 | 6.60E-09 | 33.69 |
| 201 | rs3918226 | T | C | 0.08 | 0.66 | 0.06 | 8.46E-31 | 133.35 |
| 202 | rs10224210 | C | T | 0.28 | 0.38 | 0.03 | 1.60E-29 | 126.96 |
| 203 | rs3735533 | C | T | 0.93 | 0.91 | 0.06 | 5.29E-56 | 248.73 |
| 204 | rs6961048 | G | C | 0.10 | 0.53 | 0.05 | 1.43E-26 | 113.89 |
| 205 | rs11977526 | A | G | 0.40 | -0.32 | 0.03 | 6.62E-25 | 106.05 |
| 206 | rs848445 | C | T | 0.71 | 0.20 | 0.03 | 2.28E-09 | 35.68 |
| 207 | rs42032 | A | G | 0.26 | -0.32 | 0.03 | 7.39E-21 | 87.71 |
| 208 | rs75672964 | T | C | 0.04 | 0.59 | 0.08 | 2.35E-12 | 49.20 |
| 209 | rs1870735 | G | C | 0.55 | -0.21 | 0.03 | 3.60E-11 | 43.87 |
| 210 | rs7821832 | G | T | 0.26 | -0.42 | 0.03 | 6.67E-34 | 147.19 |
| 211 | rs1906672 | A | G | 0.23 | 0.30 | 0.04 | 1.20E-16 | 68.64 |
| 212 | rs4734868 | G | A | 0.33 | 0.18 | 0.03 | 9.22E-09 | 33.00 |
| 213 | rs4440615 | A | G | 0.63 | -0.22 | 0.03 | 1.87E-12 | 49.77 |
| 214 | rs4876133 | C | G | 0.71 | 0.22 | 0.03 | 6.23E-11 | 42.87 |
| 215 | rs9918876 | A | C | 0.10 | -0.30 | 0.05 | 2.28E-09 | 35.69 |
| 216 | rs148401029 | A | C | 0.04 | -0.46 | 0.08 | 4.97E-08 | 29.72 |
| 217 | rs2470004 | T | C | 0.82 | -0.35 | 0.04 | 1.28E-18 | 77.64 |
| 218 | rs1821002 | G | C | 0.59 | -0.38 | 0.03 | 5.19E-35 | 152.73 |
| 219 | rs7844887 | A | G | 0.22 | 0.27 | 0.04 | 2.40E-13 | 53.78 |
| 220 | rs77375686 | G | A | 0.11 | 0.35 | 0.05 | 8.38E-13 | 51.10 |
| 221 | rs2354862 | C | A | 0.36 | -0.25 | 0.03 | 2.42E-15 | 62.54 |
| 222 | rs62512914 | G | A | 0.42 | -0.21 | 0.03 | 1.42E-11 | 45.58 |
| 223 | rs1786345 | C | A | 0.43 | -0.21 | 0.03 | 1.34E-11 | 45.60 |
| 224 | rs6986368 | T | A | 0.33 | 0.21 | 0.03 | 9.62E-11 | 41.99 |
| 225 | rs4260863 | G | C | 0.38 | -0.19 | 0.03 | 1.17E-09 | 37.04 |
| 226 | rs4873492 | T | C | 0.17 | 0.34 | 0.04 | 1.61E-17 | 72.48 |
| 227 | rs34917849 | C | G | 0.13 | 0.31 | 0.05 | 5.97E-12 | 47.35 |
| 228 | rs2608029 | G | C | 0.33 | -0.18 | 0.03 | 1.61E-08 | 31.82 |
| 229 | rs7463212 | A | T | 0.54 | -0.28 | 0.03 | 1.81E-19 | 81.47 |
| 230 | rs13253358 | T | C | 0.30 | 0.21 | 0.03 | 1.13E-10 | 41.54 |
| 231 | rs2126474 | T | G | 0.41 | -0.26 | 0.03 | 1.87E-17 | 72.25 |
| 232 | rs79069610 | C | T | 0.05 | 0.40 | 0.07 | 3.68E-08 | 30.35 |
| 233 | rs35783704 | A | G | 0.10 | -0.46 | 0.05 | 8.81E-20 | 83.00 |
| 234 | rs7012866 | G | T | 0.50 | 0.23 | 0.03 | 1.21E-14 | 59.66 |
| 235 | rs4961293 | T | C | 0.45 | 0.23 | 0.03 | 7.35E-14 | 56.03 |
| 236 | rs9886665 | C | T | 0.73 | -0.20 | 0.03 | 2.47E-09 | 35.65 |
| 237 | rs1410222 | T | C | 0.82 | 0.22 | 0.04 | 2.17E-08 | 31.37 |
| 238 | rs3104552 | C | T | 0.44 | -0.24 | 0.03 | 6.31E-16 | 65.33 |
| 239 | rs1332813 | C | T | 0.65 | -0.22 | 0.03 | 2.32E-12 | 49.22 |
| 240 | rs10980408 | C | T | 0.04 | 0.76 | 0.08 | 3.83E-20 | 84.59 |
| 241 | rs60191654 | G | A | 0.19 | 0.24 | 0.04 | 5.88E-10 | 38.28 |
| 242 | rs76452347 | T | C | 0.21 | -0.30 | 0.04 | 7.13E-14 | 56.12 |
| 243 | rs7026176 | T | G | 0.51 | -0.19 | 0.03 | 4.01E-10 | 39.07 |
| 244 | rs4838021 | T | C | 0.13 | -0.30 | 0.05 | 3.13E-11 | 44.12 |
| 245 | rs927315 | T | C | 0.47 | 0.17 | 0.03 | 2.44E-08 | 31.07 |
| 246 | rs4553000 | T | C | 0.51 | -0.20 | 0.03 | 1.09E-11 | 46.01 |
| 247 | rs7045409 | A | T | 0.37 | -0.19 | 0.03 | 2.55E-09 | 35.39 |
| 248 | rs34025993 | G | A | 0.59 | -0.22 | 0.03 | 4.71E-13 | 52.42 |
| 249 | rs6271 | T | C | 0.07 | -0.55 | 0.06 | 1.18E-19 | 82.42 |
| 250 | rs11145807 | G | A | 0.59 | -0.21 | 0.03 | 3.54E-11 | 43.96 |
| 251 | rs4284362 | A | C | 0.72 | -0.23 | 0.03 | 2.61E-11 | 44.55 |
| 252 | rs4245599 | G | A | 0.54 | 0.18 | 0.03 | 4.03E-09 | 34.60 |
| 253 | rs10749572 | T | G | 0.54 | -0.20 | 0.03 | 1.88E-11 | 45.18 |
| 254 | rs2689690 | T | C | 0.37 | -0.27 | 0.03 | 1.15E-17 | 73.11 |
| 255 | rs11252324 | T | G | 0.08 | -0.42 | 0.06 | 3.61E-13 | 52.81 |
| 256 | rs12258967 | G | C | 0.30 | -0.63 | 0.03 | 1.08E-78 | 352.48 |
| 257 | rs34130368 | T | G | 0.12 | -0.30 | 0.05 | 1.28E-09 | 36.83 |
| 258 | rs57946343 | C | T | 0.15 | -0.72 | 0.04 | 2.10E-63 | 282.49 |
| 259 | rs111866816 | T | C | 0.07 | 0.36 | 0.06 | 2.29E-09 | 35.74 |
| 260 | rs11191580 | C | T | 0.08 | -1.10 | 0.06 | 7.74E-89 | 399.64 |
| 261 | rs12264186 | T | C | 0.19 | 0.21 | 0.04 | 3.58E-08 | 30.44 |
| 262 | rs2177843 | T | C | 0.15 | 0.44 | 0.04 | 2.80E-24 | 103.46 |
| 263 | rs1006545 | T | G | 0.89 | 0.68 | 0.05 | 3.50E-46 | 203.42 |
| 264 | rs740746 | A | G | 0.73 | 0.46 | 0.03 | 1.42E-40 | 177.54 |
| 265 | rs7912283 | A | G | 0.65 | -0.21 | 0.03 | 2.94E-11 | 44.33 |
| 266 | rs12255372 | T | G | 0.29 | 0.24 | 0.03 | 1.94E-12 | 49.54 |
| 267 | rs11592107 | A | G | 0.31 | 0.30 | 0.03 | 1.55E-20 | 86.05 |
| 268 | rs1623474 | T | C | 0.33 | 0.38 | 0.03 | 7.66E-33 | 142.14 |
| 269 | rs3802517 | A | T | 0.46 | 0.25 | 0.03 | 4.65E-17 | 70.48 |
| 270 | rs2236295 | T | G | 0.40 | -0.30 | 0.03 | 1.04E-22 | 96.03 |
| 271 | rs57866767 | C | T | 0.43 | -0.45 | 0.03 | 1.14E-49 | 219.22 |
| 272 | rs117464403 | A | G | 0.02 | 0.86 | 0.12 | 5.80E-13 | 51.93 |
| 273 | rs60444686 | A | G | 0.04 | 0.59 | 0.08 | 4.42E-14 | 57.02 |
| 274 | rs7093894 | A | C | 0.15 | 0.24 | 0.04 | 3.16E-08 | 30.55 |
| 275 | rs1133400 | G | A | 0.21 | 0.30 | 0.04 | 2.53E-15 | 62.60 |
| 276 | rs871004 | A | G | 0.35 | 0.23 | 0.03 | 1.65E-13 | 54.30 |
| 277 | rs2904315 | G | A | 0.69 | 0.21 | 0.03 | 1.58E-10 | 41.00 |
| 278 | rs67885470 | T | C | 0.21 | -0.21 | 0.04 | 4.12E-08 | 30.16 |
| 279 | rs604723 | C | T | 0.72 | 0.66 | 0.03 | 2.55E-83 | 373.32 |
| 280 | rs7926110 | G | T | 0.33 | -0.26 | 0.03 | 5.71E-16 | 65.76 |
| 281 | rs74048190 | C | T | 0.05 | 0.44 | 0.08 | 6.07E-09 | 33.85 |
| 282 | rs1544861 | C | T | 0.66 | -0.20 | 0.03 | 5.66E-10 | 38.34 |
| 283 | rs11604357 | A | C | 0.16 | -0.28 | 0.04 | 1.60E-11 | 45.42 |
| 284 | rs7107356 | G | A | 0.50 | 0.46 | 0.03 | 1.63E-52 | 233.35 |
| 285 | rs66864335 | A | G | 0.22 | -0.40 | 0.04 | 1.79E-27 | 117.89 |
| 286 | rs7395791 | A | G | 0.44 | -0.22 | 0.03 | 2.19E-12 | 49.27 |
| 287 | rs2289124 | A | G | 0.17 | -0.31 | 0.04 | 1.14E-13 | 55.08 |
| 288 | rs10750441 | T | C | 0.66 | 0.18 | 0.03 | 3.74E-08 | 30.23 |
| 289 | rs4980379 | T | C | 0.37 | 0.58 | 0.03 | 2.47E-72 | 324.45 |
| 290 | rs2957688 | A | G | 0.47 | 0.35 | 0.03 | 2.74E-30 | 130.44 |
| 291 | rs17762 | A | G | 0.08 | 0.41 | 0.06 | 5.60E-13 | 51.99 |
| 292 | rs1382472 | A | G | 0.40 | -0.19 | 0.03 | 4.47E-10 | 38.99 |
| 293 | rs2276153 | G | C | 0.24 | -0.33 | 0.04 | 6.56E-21 | 88.18 |
| 294 | rs72931748 | G | A | 0.10 | -0.40 | 0.05 | 6.40E-14 | 56.24 |
| 295 | rs641620 | C | T | 0.15 | 0.32 | 0.04 | 3.74E-13 | 52.66 |
| 296 | rs573455 | G | A | 0.54 | -0.20 | 0.03 | 4.77E-11 | 43.31 |
| 297 | rs2014408 | T | C | 0.21 | 0.52 | 0.04 | 1.26E-43 | 192.04 |
| 298 | rs177551 | A | C | 0.13 | 0.37 | 0.04 | 3.47E-17 | 71.22 |
| 299 | rs509564 | T | C | 0.23 | 0.26 | 0.04 | 5.08E-13 | 52.17 |
| 300 | rs74538877 | C | G | 0.06 | -0.39 | 0.07 | 4.97E-08 | 29.73 |
| 301 | rs11222084 | T | A | 0.36 | 0.34 | 0.03 | 1.80E-26 | 113.26 |
| 302 | rs1340030 | C | T | 0.37 | -0.19 | 0.03 | 5.77E-10 | 38.50 |
| 303 | rs10501410 | A | G | 0.07 | 0.41 | 0.06 | 1.10E-11 | 46.11 |
| 304 | rs78998485 | G | C | 0.26 | 0.24 | 0.03 | 1.48E-12 | 50.10 |
| 305 | rs7134440 | T | C | 0.08 | 0.48 | 0.06 | 1.58E-17 | 72.58 |
| 306 | rs17249754 | A | G | 0.17 | -0.84 | 0.04 | 1.25E-97 | 439.23 |
| 307 | rs10777213 | A | G | 0.52 | -0.18 | 0.03 | 2.45E-09 | 35.68 |
| 308 | rs5742643 | C | T | 0.75 | 0.22 | 0.03 | 1.53E-10 | 40.94 |
| 309 | rs117206641 | T | C | 0.11 | 0.32 | 0.05 | 2.66E-10 | 39.95 |
| 310 | rs113695818 | T | C | 0.30 | -0.18 | 0.03 | 2.62E-08 | 30.92 |
| 311 | rs1010064 | C | A | 0.18 | -0.36 | 0.04 | 3.02E-20 | 85.14 |
| 312 | rs2129869 | T | A | 0.22 | 0.26 | 0.04 | 2.44E-13 | 53.60 |
| 313 | rs61917655 | T | C | 0.10 | 0.34 | 0.05 | 2.68E-11 | 44.45 |
| 314 | rs7134677 | T | C | 0.30 | -0.39 | 0.03 | 4.46E-31 | 134.55 |
| 315 | rs7980644 | G | A | 0.83 | -0.26 | 0.04 | 6.30E-11 | 42.73 |
| 316 | rs3819532 | C | T | 0.61 | 0.19 | 0.03 | 9.44E-10 | 37.55 |
| 317 | rs2024385 | A | T | 0.42 | -0.26 | 0.03 | 5.88E-18 | 74.55 |
| 318 | rs73075659 | G | A | 0.33 | -0.40 | 0.03 | 5.52E-35 | 152.34 |
| 319 | rs12426261 | G | A | 0.62 | -0.38 | 0.03 | 2.31E-34 | 149.25 |
| 320 | rs4143175 | C | T | 0.76 | -0.22 | 0.04 | 5.10E-10 | 38.60 |
| 321 | rs7963801 | C | T | 0.58 | 0.24 | 0.03 | 2.87E-14 | 57.68 |
| 322 | rs1169078 | G | C | 0.31 | 0.20 | 0.03 | 1.68E-09 | 36.33 |
| 323 | rs11834380 | A | C | 0.10 | -0.28 | 0.05 | 4.77E-08 | 29.82 |
| 324 | rs6490019 | G | A | 0.62 | 0.29 | 0.03 | 6.61E-21 | 87.90 |
| 325 | rs7306710 | C | T | 0.52 | 0.24 | 0.03 | 1.02E-15 | 64.26 |
| 326 | rs7310615 | G | C | 0.52 | -0.59 | 0.03 | 1.32E-81 | 365.48 |
| 327 | rs1896326 | A | G | 0.23 | -0.28 | 0.04 | 4.40E-14 | 56.84 |
| 328 | rs35444 | G | A | 0.39 | -0.44 | 0.03 | 3.47E-45 | 198.54 |
| 329 | rs17245822 | C | A | 0.37 | 0.19 | 0.03 | 1.15E-09 | 37.05 |
| 330 | rs7331680 | T | G | 0.15 | 0.41 | 0.04 | 3.35E-22 | 93.99 |
| 331 | rs75961402 | A | G | 0.15 | 0.27 | 0.04 | 1.95E-10 | 40.47 |
| 332 | rs483071 | T | C | 0.62 | 0.27 | 0.03 | 5.09E-18 | 74.91 |
| 333 | rs9507885 | T | C | 0.10 | -0.32 | 0.05 | 3.23E-09 | 35.03 |
| 334 | rs9508495 | T | C | 0.76 | -0.36 | 0.04 | 6.34E-24 | 101.54 |
| 335 | rs4274337 | G | A | 0.83 | 0.30 | 0.04 | 2.48E-13 | 53.44 |
| 336 | rs7491248 | A | G | 0.22 | 0.22 | 0.04 | 2.37E-09 | 35.70 |
| 337 | rs9526707 | A | G | 0.32 | -0.20 | 0.03 | 2.77E-10 | 39.85 |
| 338 | rs78474310 | G | A | 0.04 | 0.47 | 0.07 | 1.51E-10 | 40.98 |
| 339 | rs6562778 | G | A | 0.54 | -0.18 | 0.03 | 4.95E-09 | 34.28 |
| 340 | rs9549627 | A | G | 0.12 | 0.28 | 0.05 | 1.25E-08 | 32.40 |
| 341 | rs365990 | G | A | 0.37 | -0.23 | 0.03 | 5.95E-13 | 52.01 |
| 342 | rs57786342 | A | G | 0.21 | 0.23 | 0.04 | 5.63E-10 | 38.38 |
| 343 | rs7154723 | A | G | 0.39 | 0.25 | 0.03 | 2.72E-16 | 67.04 |
| 344 | rs8904 | A | G | 0.37 | 0.31 | 0.03 | 1.71E-22 | 95.03 |
| 345 | rs72683923 | C | T | 0.02 | -0.96 | 0.11 | 3.08E-18 | 75.82 |
| 346 | rs3815460 | G | C | 0.10 | 0.29 | 0.05 | 1.21E-08 | 32.49 |
| 347 | rs7493678 | T | A | 0.35 | 0.19 | 0.03 | 2.31E-09 | 35.77 |
| 348 | rs35413927 | G | A | 0.31 | 0.30 | 0.03 | 5.25E-20 | 83.77 |
| 349 | rs12883810 | T | C | 0.15 | -0.24 | 0.04 | 2.70E-08 | 30.97 |
| 350 | rs11159091 | A | G | 0.46 | 0.20 | 0.03 | 6.79E-11 | 42.62 |
| 351 | rs75016974 | T | C | 0.14 | -0.25 | 0.04 | 1.05E-08 | 32.77 |
| 352 | rs12885878 | G | A | 0.77 | 0.23 | 0.04 | 4.32E-10 | 38.97 |
| 353 | rs8003103 | A | G | 0.34 | -0.18 | 0.03 | 3.60E-08 | 30.27 |
| 354 | rs17562391 | T | C | 0.42 | 0.20 | 0.03 | 1.35E-10 | 41.32 |
| 355 | rs2652812 | T | C | 0.75 | -0.25 | 0.04 | 1.03E-12 | 50.80 |
| 356 | rs11636952 | C | T | 0.69 | -0.53 | 0.03 | 4.22E-59 | 262.38 |
| 357 | rs1994158 | G | A | 0.18 | -0.25 | 0.04 | 1.23E-10 | 41.31 |
| 358 | rs17807723 | A | G | 0.14 | -0.27 | 0.04 | 8.43E-10 | 37.73 |
| 359 | rs4932373 | C | A | 0.33 | 0.64 | 0.03 | 2.49E-83 | 374.80 |
| 360 | rs12906962 | C | T | 0.32 | 0.27 | 0.03 | 3.28E-16 | 66.64 |
| 361 | rs8030856 | G | C | 0.40 | 0.18 | 0.03 | 1.21E-08 | 32.38 |
| 362 | rs2589218 | C | T | 0.27 | 0.23 | 0.03 | 2.54E-11 | 44.37 |
| 363 | rs4606697 | A | G | 0.10 | -0.32 | 0.05 | 9.71E-10 | 37.34 |
| 364 | rs28866311 | G | T | 0.47 | 0.28 | 0.03 | 5.45E-20 | 83.64 |
| 365 | rs28429256 | A | G | 0.33 | 0.22 | 0.03 | 3.89E-11 | 43.76 |
| 366 | rs2627313 | T | C | 0.45 | 0.32 | 0.03 | 3.55E-26 | 112.09 |
| 367 | rs4775769 | G | T | 0.91 | 0.42 | 0.05 | 7.76E-16 | 64.81 |
| 368 | rs3098186 | T | C | 0.52 | -0.24 | 0.03 | 1.41E-15 | 63.89 |
| 369 | rs4784541 | C | T | 0.53 | 0.20 | 0.03 | 4.93E-11 | 43.08 |
| 370 | rs6540119 | T | A | 0.67 | -0.20 | 0.03 | 3.93E-10 | 39.20 |
| 371 | rs1049212 | G | A | 0.57 | 0.30 | 0.03 | 4.59E-23 | 98.02 |
| 372 | rs35098810 | C | A | 0.23 | -0.20 | 0.04 | 3.20E-08 | 30.53 |
| 373 | rs146550789 | C | T | 0.04 | 0.48 | 0.08 | 5.64E-10 | 38.45 |
| 374 | rs12596630 | T | C | 0.09 | 0.43 | 0.05 | 5.01E-15 | 61.17 |
| 375 | rs2283500 | C | A | 0.11 | -0.31 | 0.05 | 1.08E-10 | 41.70 |
| 376 | rs62047964 | T | C | 0.06 | 0.51 | 0.07 | 9.29E-14 | 55.60 |
| 377 | rs1012089 | G | C | 0.52 | 0.19 | 0.03 | 1.95E-10 | 40.42 |
| 378 | rs3950627 | A | C | 0.53 | 0.19 | 0.03 | 1.82E-09 | 36.12 |
| 379 | rs908951 | T | C | 0.44 | -0.23 | 0.03 | 7.14E-13 | 51.52 |
| 380 | rs11641374 | A | C | 0.60 | -0.19 | 0.03 | 3.26E-10 | 39.54 |
| 381 | rs7186298 | T | C | 0.43 | -0.23 | 0.03 | 1.88E-14 | 58.76 |
| 382 | rs4888408 | A | G | 0.59 | 0.37 | 0.03 | 1.42E-32 | 141.59 |
| 383 | rs12926550 | A | G | 0.32 | -0.25 | 0.03 | 3.43E-15 | 61.85 |
| 384 | rs7198817 | A | C | 0.63 | -0.18 | 0.03 | 5.99E-09 | 33.96 |
| 385 | rs77924615 | A | G | 0.20 | -0.41 | 0.04 | 1.12E-25 | 109.50 |
| 386 | rs8044992 | C | T | 0.29 | -0.21 | 0.03 | 1.07E-10 | 41.72 |
| 387 | rs34941092 | A | G | 0.15 | -0.32 | 0.04 | 3.23E-14 | 57.58 |
| 388 | rs8054587 | C | T | 0.47 | -0.17 | 0.03 | 3.41E-08 | 30.40 |
| 389 | rs79930761 | T | C | 0.09 | -0.47 | 0.06 | 4.90E-17 | 70.33 |
| 390 | rs9302885 | G | A | 0.55 | -0.22 | 0.03 | 1.03E-13 | 55.11 |
| 391 | rs8079811 | G | C | 0.65 | 0.21 | 0.03 | 1.03E-10 | 41.79 |
| 392 | rs4925159 | A | G | 0.42 | 0.22 | 0.03 | 9.66E-13 | 50.81 |
| 393 | rs9899540 | T | A | 0.60 | -0.20 | 0.03 | 1.87E-10 | 40.50 |
| 394 | rs7213273 | A | G | 0.66 | -0.40 | 0.03 | 6.24E-37 | 161.25 |
| 395 | rs17608766 | C | T | 0.14 | 0.69 | 0.04 | 2.48E-57 | 254.16 |
| 396 | rs62076622 | G | A | 0.20 | -0.24 | 0.04 | 3.79E-10 | 39.29 |
| 397 | rs4511593 | T | C | 0.65 | -0.29 | 0.03 | 1.28E-19 | 82.08 |
| 398 | rs1551355 | T | C | 0.23 | 0.21 | 0.04 | 3.89E-09 | 34.73 |
| 399 | rs9897429 | A | G | 0.52 | 0.26 | 0.03 | 1.19E-16 | 68.75 |
| 400 | rs1000423 | T | C | 0.73 | 0.41 | 0.03 | 6.50E-33 | 143.03 |
| 401 | rs56288724 | G | A | 0.42 | 0.22 | 0.03 | 2.01E-12 | 49.36 |
| 402 | rs3764400 | C | T | 0.14 | -0.37 | 0.04 | 3.69E-17 | 70.94 |
| 403 | rs11655604 | T | C | 0.36 | -0.20 | 0.03 | 1.09E-09 | 37.27 |
| 404 | rs2760748 | A | T | 0.10 | 0.36 | 0.05 | 1.05E-12 | 50.75 |
| 405 | rs113086489 | T | C | 0.55 | 0.32 | 0.03 | 3.80E-26 | 112.00 |
| 406 | rs7211535 | G | A | 0.52 | 0.18 | 0.03 | 4.61E-09 | 34.25 |
| 407 | rs6504213 | C | T | 0.58 | 0.30 | 0.03 | 1.25E-21 | 91.35 |
| 408 | rs1436138 | G | A | 0.36 | -0.31 | 0.03 | 4.73E-23 | 98.04 |
| 409 | rs34413141 | A | T | 0.18 | -0.35 | 0.04 | 2.47E-19 | 80.73 |
| 410 | rs10048404 | T | C | 0.37 | -0.26 | 0.03 | 1.91E-16 | 67.63 |
| 411 | rs56407827 | T | C | 0.27 | 0.36 | 0.03 | 2.78E-26 | 112.30 |
| 412 | rs7236548 | A | C | 0.18 | 0.34 | 0.04 | 8.51E-19 | 78.19 |
| 413 | rs665445 | A | C | 0.28 | -0.19 | 0.03 | 1.15E-08 | 32.67 |
| 414 | rs62082230 | A | T | 0.28 | -0.19 | 0.03 | 4.69E-08 | 29.82 |
| 415 | rs11874246 | T | C | 0.30 | 0.29 | 0.03 | 3.22E-18 | 75.82 |
| 416 | rs1437649 | A | G | 0.23 | -0.22 | 0.04 | 8.57E-10 | 37.60 |
| 417 | rs10460108 | G | A | 0.52 | -0.21 | 0.03 | 1.12E-12 | 50.59 |
| 418 | rs1154214 | G | T | 0.60 | 0.20 | 0.03 | 3.27E-11 | 44.05 |
| 419 | rs141958336 | A | G | 0.04 | 0.78 | 0.08 | 1.36E-23 | 100.18 |
| 420 | rs34518929 | A | G | 0.26 | -0.22 | 0.03 | 1.79E-10 | 40.66 |
| 421 | rs60138042 | G | C | 0.06 | -0.34 | 0.06 | 4.14E-08 | 30.08 |
| 422 | rs1433121 | T | C | 0.69 | -0.23 | 0.03 | 2.66E-12 | 48.91 |
| 423 | rs11672660 | T | C | 0.20 | 0.22 | 0.04 | 6.32E-09 | 33.71 |
| 424 | rs12610654 | G | A | 0.34 | -0.23 | 0.03 | 4.41E-13 | 52.34 |
| 425 | rs12978472 | G | C | 0.12 | -0.85 | 0.05 | 1.23E-66 | 297.39 |
| 426 | rs2291516 | A | G | 0.10 | 0.37 | 0.05 | 2.17E-13 | 53.91 |
| 427 | rs10420519 | T | G | 0.03 | -0.49 | 0.09 | 2.86E-08 | 30.78 |
| 428 | rs571689 | T | C | 0.52 | 0.23 | 0.03 | 6.77E-14 | 56.25 |
| 429 | rs8113613 | T | C | 0.18 | -0.23 | 0.04 | 4.59E-09 | 34.39 |
| 430 | rs1848994 | A | G | 0.28 | 0.20 | 0.03 | 1.79E-09 | 36.29 |
| 431 | rs28572357 | C | A | 0.40 | 0.27 | 0.03 | 6.34E-19 | 78.74 |
| 432 | rs7255933 | A | G | 0.26 | 0.23 | 0.03 | 2.44E-11 | 44.68 |
| 433 | rs73046792 | A | G | 0.16 | -0.36 | 0.04 | 7.23E-17 | 69.60 |
| 434 | rs6108787 | G | T | 0.47 | 0.43 | 0.03 | 5.38E-46 | 202.97 |
| 435 | rs2801008 | G | T | 0.32 | 0.19 | 0.03 | 7.37E-09 | 33.53 |
| 436 | rs6054139 | A | G | 0.61 | 0.21 | 0.03 | 8.23E-12 | 46.83 |
| 437 | rs2423514 | G | A | 0.46 | -0.30 | 0.03 | 1.77E-23 | 99.40 |
| 438 | rs6078093 | A | G | 0.43 | -0.18 | 0.03 | 1.20E-09 | 36.99 |
| 439 | rs8125763 | A | C | 0.47 | 0.18 | 0.03 | 4.84E-09 | 34.23 |
| 440 | rs6058088 | G | T | 0.16 | -0.28 | 0.04 | 1.14E-11 | 46.12 |
| 441 | rs6029756 | A | G | 0.32 | -0.27 | 0.03 | 1.88E-16 | 67.54 |
| 442 | rs2598 | G | A | 0.47 | -0.17 | 0.03 | 2.87E-08 | 30.74 |
| 443 | rs6090907 | A | G | 0.15 | -0.39 | 0.04 | 1.29E-19 | 82.23 |
| 444 | rs6026578 | G | C | 0.63 | 0.19 | 0.03 | 4.59E-09 | 34.27 |
| 445 | rs79384779 | T | C | 0.15 | 0.32 | 0.04 | 1.08E-13 | 55.17 |
| 446 | rs6026744 | T | A | 0.12 | 0.71 | 0.05 | 7.00E-54 | 239.28 |
| 447 | rs17812022 | T | C | 0.10 | -0.36 | 0.05 | 5.65E-12 | 47.36 |
| 448 | rs6031431 | G | A | 0.46 | 0.26 | 0.03 | 7.05E-18 | 74.11 |
| 449 | rs6062324 | A | G | 0.24 | -0.33 | 0.04 | 1.18E-19 | 82.34 |
| 450 | rs2833834 | A | C | 0.28 | 0.22 | 0.03 | 1.22E-10 | 41.48 |
| 451 | rs12627651 | A | G | 0.29 | 0.35 | 0.03 | 1.02E-24 | 105.23 |
| 452 | rs2776037 | C | T | 0.58 | 0.19 | 0.03 | 2.15E-09 | 35.88 |
| 453 | rs1882961 | T | C | 0.31 | 0.24 | 0.03 | 6.69E-14 | 56.16 |
| 454 | rs34487963 | A | C | 0.02 | -0.88 | 0.12 | 1.35E-12 | 50.26 |
| 455 | rs7278003 | C | T | 0.56 | 0.19 | 0.03 | 6.63E-10 | 38.08 |
| 456 | rs112854918 | G | C | 0.03 | 0.56 | 0.10 | 2.77E-08 | 30.86 |
| 457 | rs2238787 | A | G | 0.29 | 0.26 | 0.03 | 1.45E-14 | 59.09 |
| 458 | rs28578714 | C | T | 0.39 | -0.21 | 0.03 | 2.53E-10 | 39.92 |
| 459 | rs12321 | C | G | 0.43 | -0.23 | 0.03 | 3.81E-14 | 57.22 |
| 460 | rs8142376 | T | C | 0.49 | 0.17 | 0.03 | 2.19E-08 | 31.21 |
| 461 | rs148140538 | T | C | 0.08 | -0.33 | 0.06 | 7.39E-09 | 33.48 |

EAF: Effect allele frequency; SBP: Systolic blood pressure

## Table 4: Characteristics of SNPs used as instrumental variables for DBP

|  | SNP | effect_allele.exposure | other_allele.exposure | eaf.exposure | effect | SE | p-value | F-Statistic |
| --- | --- | --- | --- | --- | --- | --- | --- | --- |
| 1 | rs12728150 | G | A | 0.08 | 0.20 | 0.03 | 1.28E-10 | 41.36 |
| 2 | rs786921 | A | G | 0.60 | -0.11 | 0.02 | 8.63E-11 | 42.32 |
| 3 | rs17396055 | A | G | 0.33 | -0.12 | 0.02 | 4.13E-10 | 39.06 |
| 4 | rs72704264 | C | G | 0.22 | 0.12 | 0.02 | 3.60E-08 | 30.46 |
| 5 | rs7524019 | T | C | 0.49 | 0.10 | 0.02 | 2.60E-09 | 35.45 |
| 6 | rs1999996 | G | A | 0.44 | 0.11 | 0.02 | 1.67E-10 | 40.79 |
| 7 | rs882624 | T | C | 0.33 | -0.16 | 0.02 | 2.33E-17 | 72.11 |
| 8 | rs602521 | A | G | 0.27 | 0.14 | 0.02 | 3.97E-12 | 48.00 |
| 9 | rs1889785 | A | G | 0.46 | 0.13 | 0.02 | 5.61E-13 | 52.02 |
| 10 | rs710249 | C | G | 0.43 | 0.15 | 0.02 | 6.43E-18 | 74.42 |
| 11 | rs78256308 | G | T | 0.02 | -0.41 | 0.07 | 3.73E-10 | 39.19 |
| 12 | rs10493408 | A | C | 0.13 | 0.16 | 0.03 | 5.09E-10 | 38.59 |
| 13 | rs1819663 | G | A | 0.49 | -0.11 | 0.02 | 4.62E-11 | 43.45 |
| 14 | rs150816167 | C | T | 0.05 | 0.29 | 0.04 | 1.17E-10 | 41.50 |
| 15 | rs4926499 | C | G | 0.83 | 0.17 | 0.02 | 9.37E-12 | 46.66 |
| 16 | rs488834 | T | C | 0.76 | -0.19 | 0.02 | 1.94E-20 | 86.19 |
| 17 | rs10776752 | T | G | 0.08 | 0.46 | 0.03 | 1.25E-43 | 192.03 |
| 18 | rs12405515 | T | G | 0.57 | -0.17 | 0.02 | 1.92E-22 | 95.23 |
| 19 | rs68085857 | T | C | 0.23 | 0.19 | 0.02 | 9.82E-21 | 86.81 |
| 20 | rs12088448 | C | A | 0.36 | 0.15 | 0.02 | 2.53E-17 | 71.97 |
| 21 | rs3943093 | T | C | 0.32 | 0.25 | 0.02 | 3.94E-41 | 181.22 |
| 22 | rs34645159 | A | G | 0.50 | -0.13 | 0.02 | 2.07E-14 | 58.43 |
| 23 | rs2493296 | T | C | 0.14 | 0.25 | 0.03 | 7.45E-23 | 96.57 |
| 24 | rs2146315 | T | C | 0.23 | -0.12 | 0.02 | 5.03E-09 | 34.09 |
| 25 | rs4926901 | A | G | 0.35 | 0.10 | 0.02 | 4.82E-08 | 29.88 |
| 26 | rs11578696 | G | A | 0.13 | -0.15 | 0.03 | 1.65E-08 | 31.98 |
| 27 | rs2169137 | C | G | 0.73 | 0.16 | 0.02 | 3.17E-16 | 67.00 |
| 28 | rs1502358 | A | G | 0.68 | -0.11 | 0.02 | 1.13E-09 | 37.11 |
| 29 | rs964941 | A | G | 0.52 | 0.17 | 0.02 | 3.31E-23 | 98.40 |
| 30 | rs55857306 | A | G | 0.16 | -0.52 | 0.02 | 5.05E-109 | 494.16 |
| 31 | rs6686889 | T | C | 0.25 | 0.19 | 0.02 | 6.95E-22 | 92.89 |
| 32 | rs4926923 | C | T | 0.09 | -0.19 | 0.03 | 4.75E-10 | 38.78 |
| 33 | rs34517439 | A | C | 0.12 | -0.25 | 0.03 | 2.02E-19 | 81.19 |
| 34 | rs57748895 | T | A | 0.02 | 0.66 | 0.07 | 2.49E-23 | 99.01 |
| 35 | rs2493136 | T | C | 0.41 | 0.23 | 0.02 | 1.92E-40 | 177.53 |
| 36 | rs2384061 | A | G | 0.42 | -0.17 | 0.02 | 2.25E-23 | 98.97 |
| 37 | rs2160236 | C | G | 0.38 | -0.14 | 0.02 | 4.31E-15 | 61.64 |
| 38 | rs2586970 | G | A | 0.56 | 0.15 | 0.02 | 1.56E-17 | 72.79 |
| 39 | rs2421200 | T | G | 0.49 | -0.11 | 0.02 | 2.59E-10 | 40.21 |
| 40 | rs1876490 | A | G | 0.72 | 0.14 | 0.02 | 1.16E-12 | 50.47 |
| 41 | rs311564 | A | G | 0.35 | -0.13 | 0.02 | 4.23E-13 | 52.82 |
| 42 | rs62158170 | G | A | 0.22 | -0.16 | 0.02 | 6.63E-15 | 60.78 |
| 43 | rs6715901 | A | G | 0.50 | -0.14 | 0.02 | 2.76E-15 | 62.63 |
| 44 | rs10804330 | C | T | 0.43 | -0.13 | 0.02 | 4.60E-14 | 57.19 |
| 45 | rs112393817 | G | C | 0.22 | -0.12 | 0.02 | 3.80E-08 | 30.22 |
| 46 | rs1468816 | C | A | 0.23 | -0.12 | 0.02 | 4.65E-09 | 34.32 |
| 47 | rs4952668 | A | G | 0.62 | -0.19 | 0.02 | 1.13E-26 | 113.78 |
| 48 | rs12990959 | C | T | 0.31 | 0.13 | 0.02 | 1.11E-11 | 46.20 |
| 49 | rs2444769 | A | C | 0.79 | 0.16 | 0.02 | 4.85E-13 | 52.05 |
| 50 | rs1035673 | C | T | 0.60 | -0.16 | 0.02 | 3.00E-20 | 85.25 |
| 51 | rs1039897 | A | G | 0.65 | -0.11 | 0.02 | 3.26E-09 | 35.15 |
| 52 | rs4507125 | C | A | 0.21 | 0.12 | 0.02 | 3.60E-09 | 34.76 |
| 53 | rs1373780 | C | G | 0.18 | 0.12 | 0.02 | 2.58E-08 | 30.94 |
| 54 | rs824523 | A | C | 0.33 | 0.12 | 0.02 | 2.26E-11 | 44.88 |
| 55 | rs62155750 | G | A | 0.31 | 0.22 | 0.02 | 8.27E-29 | 123.37 |
| 56 | rs7569128 | A | C | 0.82 | 0.20 | 0.02 | 1.22E-18 | 77.52 |
| 57 | rs4675682 | C | T | 0.46 | 0.14 | 0.02 | 4.49E-16 | 66.33 |
| 58 | rs1275985 | T | C | 0.61 | -0.29 | 0.02 | 2.77E-62 | 276.46 |
| 59 | rs56809883 | T | G | 0.26 | 0.11 | 0.02 | 3.03E-08 | 30.67 |
| 60 | rs34103412 | A | G | 0.16 | 0.15 | 0.02 | 1.92E-10 | 40.55 |
| 61 | rs4954192 | T | C | 0.39 | -0.12 | 0.02 | 8.15E-12 | 46.83 |
| 62 | rs55944332 | G | A | 0.24 | 0.24 | 0.02 | 3.27E-31 | 134.40 |
| 63 | rs7572130 | G | A | 0.10 | 0.18 | 0.03 | 4.12E-10 | 39.16 |
| 64 | rs7576060 | T | C | 0.35 | -0.10 | 0.02 | 2.05E-08 | 31.57 |
| 65 | rs11692619 | T | C | 0.36 | -0.13 | 0.02 | 3.31E-12 | 48.47 |
| 66 | rs13004222 | G | C | 0.05 | -0.29 | 0.04 | 7.11E-14 | 56.12 |
| 67 | rs1044822 | T | C | 0.15 | -0.13 | 0.02 | 4.14E-08 | 30.14 |
| 68 | rs76326501 | C | A | 0.09 | -0.36 | 0.03 | 2.17E-32 | 140.71 |
| 69 | rs6546810 | C | T | 0.35 | 0.12 | 0.02 | 3.16E-11 | 43.95 |
| 70 | rs28377357 | A | G | 0.29 | -0.12 | 0.02 | 6.03E-11 | 42.80 |
| 71 | rs1446468 | C | T | 0.55 | 0.25 | 0.02 | 1.21E-47 | 211.42 |
| 72 | rs6735275 | C | T | 0.27 | -0.12 | 0.02 | 2.62E-10 | 39.87 |
| 73 | rs12693302 | A | G | 0.65 | -0.24 | 0.02 | 2.15E-39 | 172.61 |
| 74 | rs1263671 | C | T | 0.16 | 0.14 | 0.02 | 4.69E-09 | 34.31 |
| 75 | rs1687295 | C | T | 0.73 | -0.21 | 0.02 | 2.99E-26 | 112.86 |
| 76 | rs11923667 | A | T | 0.41 | 0.12 | 0.02 | 3.10E-11 | 44.07 |
| 77 | rs6777317 | A | G | 0.29 | 0.12 | 0.02 | 1.51E-10 | 41.03 |
| 78 | rs2643826 | T | C | 0.45 | 0.19 | 0.02 | 2.83E-26 | 112.60 |
| 79 | rs7427249 | A | G | 0.58 | -0.11 | 0.02 | 4.34E-10 | 38.92 |
| 80 | rs6800730 | G | A | 0.67 | 0.25 | 0.02 | 8.00E-41 | 179.13 |
| 81 | rs6795735 | T | C | 0.41 | -0.14 | 0.02 | 3.05E-16 | 66.76 |
| 82 | rs9289557 | T | C | 0.26 | -0.12 | 0.02 | 8.68E-09 | 33.05 |
| 83 | rs78151625 | C | T | 0.17 | 0.19 | 0.02 | 1.04E-15 | 64.34 |
| 84 | rs16853198 | G | A | 0.08 | -0.34 | 0.03 | 4.44E-25 | 107.22 |
| 85 | rs147501096 | C | G | 0.07 | -0.20 | 0.03 | 9.94E-09 | 32.87 |
| 86 | rs4244200 | C | G | 0.28 | -0.12 | 0.02 | 3.23E-10 | 39.63 |
| 87 | rs347585 | T | C | 0.70 | 0.15 | 0.02 | 1.57E-15 | 63.49 |
| 88 | rs62234672 | A | C | 0.18 | 0.12 | 0.02 | 4.92E-08 | 29.70 |
| 89 | rs114714860 | C | G | 0.17 | 0.33 | 0.02 | 1.42E-44 | 195.53 |
| 90 | rs9841978 | A | G | 0.33 | 0.18 | 0.02 | 1.12E-21 | 91.13 |
| 91 | rs11130602 | A | G | 0.44 | 0.15 | 0.02 | 4.27E-17 | 70.27 |
| 92 | rs3774702 | A | G | 0.18 | 0.15 | 0.02 | 1.18E-10 | 41.57 |
| 93 | rs11923343 | G | A | 0.64 | 0.11 | 0.02 | 3.10E-10 | 39.53 |
| 94 | rs28675079 | A | G | 0.19 | -0.14 | 0.02 | 8.34E-11 | 42.31 |
| 95 | rs4141663 | T | C | 0.42 | -0.15 | 0.02 | 1.41E-17 | 73.08 |
| 96 | rs4077158 | C | T | 0.53 | 0.18 | 0.02 | 3.09E-26 | 112.14 |
| 97 | rs6763931 | A | G | 0.44 | 0.14 | 0.02 | 1.48E-15 | 63.91 |
| 98 | rs1527797 | T | C | 0.74 | -0.14 | 0.02 | 8.68E-13 | 51.16 |
| 99 | rs6779368 | G | A | 0.34 | 0.18 | 0.02 | 2.28E-22 | 94.74 |
| 100 | rs7623706 | G | A | 0.43 | -0.10 | 0.02 | 2.83E-08 | 30.69 |
| 101 | rs7611674 | G | T | 0.20 | -0.16 | 0.02 | 1.67E-12 | 49.95 |
| 102 | rs3864004 | A | G | 0.47 | 0.10 | 0.02 | 6.28E-09 | 33.68 |
| 103 | rs3772219 | C | A | 0.32 | -0.18 | 0.02 | 2.94E-21 | 89.89 |
| 104 | rs12152463 | T | C | 0.43 | 0.10 | 0.02 | 8.01E-09 | 33.43 |
| 105 | rs78809139 | A | G | 0.10 | -0.23 | 0.03 | 2.58E-15 | 62.73 |
| 106 | rs1528293 | T | A | 0.51 | -0.28 | 0.02 | 1.48E-57 | 255.26 |
| 107 | rs11721984 | T | C | 0.45 | -0.14 | 0.02 | 1.89E-15 | 63.37 |
| 108 | rs12509595 | C | T | 0.29 | 0.50 | 0.02 | 1.58E-148 | 670.59 |
| 109 | rs4245930 | A | G | 0.63 | -0.12 | 0.02 | 1.14E-11 | 45.86 |
| 110 | rs72719149 | C | T | 0.32 | 0.13 | 0.02 | 6.34E-12 | 47.28 |
| 111 | rs13107325 | T | C | 0.07 | -0.67 | 0.03 | 3.72E-88 | 396.12 |
| 112 | rs13139571 | A | C | 0.24 | -0.24 | 0.02 | 2.29E-32 | 140.71 |
| 113 | rs1425486 | T | C | 0.32 | -0.13 | 0.02 | 1.11E-12 | 50.66 |
| 114 | rs16896276 | A | T | 0.26 | -0.13 | 0.02 | 3.82E-11 | 43.71 |
| 115 | rs62301873 | G | A | 0.11 | 0.17 | 0.03 | 1.06E-09 | 37.28 |
| 116 | rs11945489 | T | C | 0.29 | -0.14 | 0.02 | 3.99E-13 | 52.56 |
| 117 | rs12503341 | A | G | 0.04 | -0.30 | 0.05 | 9.43E-11 | 41.97 |
| 118 | rs990619 | G | C | 0.52 | 0.16 | 0.02 | 2.90E-20 | 84.68 |
| 119 | rs28667801 | T | A | 0.41 | 0.16 | 0.02 | 1.90E-19 | 81.20 |
| 120 | rs13152154 | T | C | 0.73 | -0.12 | 0.02 | 1.23E-09 | 36.99 |
| 121 | rs61789369 | G | A | 0.04 | 0.30 | 0.04 | 3.06E-12 | 48.58 |
| 122 | rs72976750 | C | T | 0.14 | 0.17 | 0.03 | 7.37E-12 | 46.85 |
| 123 | rs7694000 | T | A | 0.46 | 0.10 | 0.02 | 3.47E-08 | 30.41 |
| 124 | rs13118687 | A | G | 0.47 | -0.15 | 0.02 | 1.37E-17 | 73.08 |
| 125 | rs66887589 | C | T | 0.48 | 0.16 | 0.02 | 1.83E-20 | 85.62 |
| 126 | rs9286351 | G | A | 0.42 | 0.14 | 0.02 | 1.61E-15 | 63.64 |
| 127 | rs13124515 | C | T | 0.69 | 0.11 | 0.02 | 1.98E-08 | 31.65 |
| 128 | rs13355146 | T | C | 0.38 | 0.12 | 0.02 | 6.39E-12 | 47.28 |
| 129 | rs11745207 | G | C | 0.26 | -0.11 | 0.02 | 1.29E-08 | 32.36 |
| 130 | rs11960210 | C | T | 0.38 | -0.25 | 0.02 | 3.36E-43 | 188.91 |
| 131 | rs2921604 | C | T | 0.46 | 0.10 | 0.02 | 4.46E-08 | 29.75 |
| 132 | rs10941043 | G | T | 0.29 | 0.13 | 0.02 | 2.52E-11 | 44.61 |
| 133 | rs15009 | G | C | 0.35 | 0.12 | 0.02 | 1.00E-11 | 46.41 |
| 134 | rs2307111 | C | T | 0.40 | 0.17 | 0.02 | 1.62E-22 | 95.78 |
| 135 | rs3776299 | A | G | 0.46 | 0.13 | 0.02 | 5.06E-13 | 52.33 |
| 136 | rs78909293 | C | T | 0.04 | -0.32 | 0.04 | 7.31E-14 | 55.99 |
| 137 | rs13358657 | G | A | 0.13 | 0.22 | 0.03 | 1.70E-18 | 77.16 |
| 138 | rs114503346 | T | C | 0.05 | -0.27 | 0.04 | 3.10E-10 | 39.52 |
| 139 | rs12656497 | C | T | 0.60 | 0.31 | 0.02 | 1.47E-67 | 302.88 |
| 140 | rs1871190 | T | G | 0.33 | 0.11 | 0.02 | 6.63E-09 | 33.59 |
| 141 | rs9326869 | C | T | 0.75 | -0.11 | 0.02 | 3.99E-08 | 30.03 |
| 142 | rs335170 | C | A | 0.59 | -0.11 | 0.02 | 1.61E-10 | 40.76 |
| 143 | rs17677603 | G | A | 0.38 | 0.20 | 0.02 | 3.90E-29 | 126.25 |
| 144 | rs1212061 | C | G | 0.73 | 0.13 | 0.02 | 7.91E-11 | 42.28 |
| 145 | rs3117736 | T | C | 0.27 | 0.24 | 0.02 | 9.71E-34 | 146.71 |
| 146 | rs10069690 | T | C | 0.26 | 0.16 | 0.02 | 1.42E-14 | 59.14 |
| 147 | rs4645335 | G | A | 0.66 | -0.11 | 0.02 | 7.04E-10 | 38.11 |
| 148 | rs6875967 | G | A | 0.65 | -0.13 | 0.02 | 1.21E-13 | 55.14 |
| 149 | rs10054208 | T | C | 0.36 | 0.12 | 0.02 | 1.49E-10 | 41.17 |
| 150 | rs62378003 | T | C | 0.11 | -0.17 | 0.03 | 8.90E-10 | 37.47 |
| 151 | rs55770741 | T | C | 0.56 | -0.13 | 0.02 | 2.20E-13 | 53.58 |
| 152 | rs1467049 | G | T | 0.19 | -0.12 | 0.02 | 1.35E-08 | 32.27 |
| 153 | rs12515541 | T | G | 0.61 | 0.12 | 0.02 | 6.23E-11 | 42.65 |
| 154 | rs10062049 | T | C | 0.14 | 0.22 | 0.03 | 4.50E-18 | 74.98 |
| 155 | rs4704514 | T | C | 0.28 | 0.11 | 0.02 | 1.71E-08 | 31.72 |
| 156 | rs1582931 | A | G | 0.47 | 0.22 | 0.02 | 4.51E-35 | 152.49 |
| 157 | rs6556384 | A | C | 0.81 | -0.15 | 0.02 | 5.91E-12 | 47.30 |
| 158 | rs55993676 | T | G | 0.29 | -0.21 | 0.02 | 3.82E-28 | 120.54 |
| 159 | rs1114347 | G | A | 0.48 | 0.18 | 0.02 | 3.32E-25 | 107.30 |
| 160 | rs509067 | C | T | 0.59 | 0.14 | 0.02 | 2.65E-16 | 67.33 |
| 161 | rs2569882 | C | T | 0.43 | -0.12 | 0.02 | 4.28E-11 | 43.40 |
| 162 | rs440454 | G | A | 0.68 | 0.26 | 0.02 | 7.52E-42 | 183.66 |
| 163 | rs62413546 | T | C | 0.08 | -0.19 | 0.03 | 4.58E-09 | 34.41 |
| 164 | rs504691 | A | C | 0.40 | -0.12 | 0.02 | 3.14E-11 | 44.22 |
| 165 | rs11153730 | C | T | 0.49 | -0.16 | 0.02 | 2.57E-19 | 80.38 |
| 166 | rs76785130 | G | A | 0.02 | 0.43 | 0.07 | 9.36E-11 | 41.90 |
| 167 | rs13215166 | G | A | 0.44 | 0.31 | 0.02 | 1.79E-70 | 316.19 |
| 168 | rs636202 | C | T | 0.52 | -0.10 | 0.02 | 4.40E-09 | 34.57 |
| 169 | rs9365555 | G | A | 0.33 | -0.13 | 0.02 | 1.96E-11 | 44.97 |
| 170 | rs11961593 | T | C | 0.07 | -0.32 | 0.03 | 1.49E-19 | 81.88 |
| 171 | rs35261542 | A | C | 0.27 | 0.12 | 0.02 | 9.29E-10 | 37.62 |
| 172 | rs2744133 | G | A | 0.27 | -0.14 | 0.02 | 1.17E-13 | 55.28 |
| 173 | rs9467545 | T | A | 0.16 | 0.25 | 0.02 | 7.39E-27 | 115.31 |
| 174 | rs115447786 | T | C | 0.04 | 0.29 | 0.05 | 1.75E-10 | 40.74 |
| 175 | rs3798293 | G | A | 0.22 | 0.13 | 0.02 | 2.70E-10 | 39.99 |
| 176 | rs62434124 | T | C | 0.07 | -0.49 | 0.03 | 7.83E-47 | 206.15 |
| 177 | rs1322639 | A | G | 0.78 | -0.16 | 0.02 | 3.87E-14 | 57.44 |
| 178 | rs1799945 | G | C | 0.15 | 0.39 | 0.02 | 3.88E-57 | 253.91 |
| 179 | rs1265157 | G | C | 0.35 | -0.14 | 0.02 | 1.18E-14 | 59.63 |
| 180 | rs6905288 | A | G | 0.57 | 0.18 | 0.02 | 7.79E-23 | 96.57 |
| 181 | rs881858 | A | G | 0.69 | 0.16 | 0.02 | 4.65E-16 | 66.11 |
| 182 | rs1984195 | A | G | 0.49 | 0.17 | 0.02 | 1.43E-23 | 100.69 |
| 183 | rs16875357 | G | T | 0.24 | 0.12 | 0.02 | 2.70E-09 | 35.24 |
| 184 | rs72613227 | T | A | 0.13 | 0.19 | 0.03 | 3.87E-11 | 43.70 |
| 185 | rs9399137 | C | T | 0.26 | -0.11 | 0.02 | 5.83E-09 | 33.96 |
| 186 | rs9791312 | C | A | 0.35 | 0.12 | 0.02 | 2.89E-11 | 44.32 |
| 187 | rs9478282 | T | C | 0.11 | -0.20 | 0.03 | 8.70E-13 | 51.08 |
| 188 | rs9406076 | T | C | 0.33 | 0.10 | 0.02 | 4.65E-08 | 29.81 |
| 189 | rs6934891 | A | G | 0.43 | 0.13 | 0.02 | 5.21E-13 | 51.89 |
| 190 | rs2397060 | C | T | 0.14 | 0.16 | 0.03 | 1.46E-10 | 41.14 |
| 191 | rs5012479 | G | T | 0.52 | -0.10 | 0.02 | 3.66E-08 | 30.19 |
| 192 | rs11153590 | A | G | 0.38 | -0.11 | 0.02 | 1.12E-10 | 41.38 |
| 193 | rs10279432 | A | C | 0.62 | 0.12 | 0.02 | 5.73E-11 | 42.83 |
| 194 | rs17432462 | C | T | 0.38 | 0.10 | 0.02 | 7.31E-09 | 33.50 |
| 195 | rs1178979 | C | T | 0.20 | -0.15 | 0.02 | 9.96E-12 | 46.31 |
| 196 | rs3807101 | T | C | 0.12 | -0.17 | 0.03 | 4.57E-11 | 43.26 |
| 197 | rs1044608 | G | C | 0.08 | 0.20 | 0.03 | 2.76E-09 | 35.44 |
| 198 | rs6464165 | C | T | 0.28 | 0.22 | 0.02 | 7.34E-29 | 123.84 |
| 199 | rs1534338 | A | G | 0.60 | -0.11 | 0.02 | 1.24E-10 | 41.31 |
| 200 | rs73033340 | G | A | 0.04 | -0.53 | 0.05 | 5.06E-24 | 102.38 |
| 201 | rs342977 | A | G | 0.77 | -0.16 | 0.02 | 1.67E-14 | 59.18 |
| 202 | rs17454517 | G | A | 0.51 | -0.12 | 0.02 | 2.65E-12 | 48.84 |
| 203 | rs310597 | G | A | 0.37 | 0.12 | 0.02 | 2.18E-10 | 40.51 |
| 204 | rs200700882 | G | A | 0.40 | 0.13 | 0.02 | 1.02E-12 | 50.83 |
| 205 | rs7805035 | A | T | 0.41 | 0.13 | 0.02 | 2.40E-14 | 57.97 |
| 206 | rs3735533 | C | T | 0.93 | 0.49 | 0.03 | 6.32E-49 | 216.47 |
| 207 | rs6961048 | G | C | 0.10 | 0.27 | 0.03 | 1.28E-21 | 91.05 |
| 208 | rs2854746 | C | G | 0.40 | 0.11 | 0.02 | 3.26E-10 | 39.41 |
| 209 | rs1449596 | G | C | 0.65 | 0.11 | 0.02 | 1.92E-09 | 35.93 |
| 210 | rs13237249 | T | C | 0.40 | 0.14 | 0.02 | 1.02E-14 | 59.56 |
| 211 | rs75511781 | G | A | 0.04 | 0.37 | 0.05 | 2.45E-15 | 62.68 |
| 212 | rs4507656 | G | C | 0.31 | 0.15 | 0.02 | 8.69E-14 | 55.84 |
| 213 | rs11556924 | T | C | 0.38 | -0.18 | 0.02 | 1.83E-23 | 100.00 |
| 214 | rs2906152 | A | G | 0.63 | -0.19 | 0.02 | 5.55E-25 | 107.08 |
| 215 | rs7788746 | T | G | 0.67 | -0.16 | 0.02 | 3.19E-19 | 80.71 |
| 216 | rs4556017 | T | C | 0.85 | -0.16 | 0.02 | 9.66E-11 | 42.01 |
| 217 | rs2191046 | G | T | 0.26 | -0.12 | 0.02 | 1.78E-09 | 36.12 |
| 218 | rs7800558 | C | T | 0.42 | -0.10 | 0.02 | 4.46E-08 | 30.09 |
| 219 | rs3918226 | T | C | 0.08 | 0.61 | 0.03 | 5.31E-77 | 345.69 |
| 220 | rs17321041 | T | C | 0.06 | 0.23 | 0.04 | 1.78E-10 | 40.60 |
| 221 | rs11778153 | C | T | 0.36 | -0.12 | 0.02 | 5.84E-11 | 42.90 |
| 222 | rs2515424 | T | C | 0.43 | 0.13 | 0.02 | 2.18E-13 | 53.95 |
| 223 | rs73276406 | C | G | 0.15 | 0.16 | 0.02 | 1.99E-10 | 40.42 |
| 224 | rs1693560 | G | A | 0.46 | -0.15 | 0.02 | 3.41E-18 | 75.94 |
| 225 | rs142449193 | T | C | 0.05 | -0.26 | 0.04 | 1.51E-09 | 36.48 |
| 226 | rs2957468 | G | A | 0.66 | -0.14 | 0.02 | 8.43E-14 | 55.40 |
| 227 | rs722783 | A | G | 0.22 | -0.21 | 0.02 | 9.03E-24 | 101.25 |
| 228 | rs35091929 | C | T | 0.60 | -0.18 | 0.02 | 6.46E-25 | 106.66 |
| 229 | rs1906672 | A | G | 0.23 | 0.14 | 0.02 | 8.47E-12 | 46.77 |
| 230 | rs148401029 | A | C | 0.04 | -0.31 | 0.05 | 1.32E-10 | 41.27 |
| 231 | rs4739832 | C | A | 0.42 | -0.13 | 0.02 | 5.81E-14 | 56.51 |
| 232 | rs9918907 | G | A | 0.22 | 0.12 | 0.02 | 1.59E-08 | 32.00 |
| 233 | rs62503324 | T | C | 0.24 | 0.20 | 0.02 | 2.11E-23 | 99.31 |
| 234 | rs10087280 | G | A | 0.17 | -0.14 | 0.02 | 2.54E-09 | 35.43 |
| 235 | rs6983239 | T | G | 0.22 | 0.12 | 0.02 | 3.71E-08 | 30.17 |
| 236 | rs4909314 | A | T | 0.39 | 0.13 | 0.02 | 3.41E-14 | 57.23 |
| 237 | rs4074812 | A | G | 0.55 | -0.13 | 0.02 | 2.07E-14 | 58.28 |
| 238 | rs951914 | C | G | 0.71 | 0.19 | 0.02 | 5.06E-23 | 97.32 |
| 239 | rs17832905 | A | C | 0.07 | 0.19 | 0.03 | 2.80E-08 | 30.89 |
| 240 | rs4873492 | T | C | 0.17 | 0.14 | 0.02 | 1.28E-09 | 36.78 |
| 241 | rs7012891 | C | T | 0.24 | 0.14 | 0.02 | 1.20E-11 | 46.04 |
| 242 | rs3802230 | A | C | 0.54 | -0.16 | 0.02 | 2.75E-20 | 85.08 |
| 243 | rs2133386 | A | C | 0.43 | -0.13 | 0.02 | 5.21E-14 | 56.42 |
| 244 | rs4615669 | G | A | 0.44 | 0.11 | 0.02 | 6.10E-11 | 42.93 |
| 245 | rs4743021 | C | T | 0.31 | 0.11 | 0.02 | 2.41E-08 | 30.99 |
| 246 | rs10759697 | A | G | 0.49 | 0.13 | 0.02 | 3.93E-14 | 57.16 |
| 247 | rs6271 | T | C | 0.07 | -0.43 | 0.04 | 1.72E-34 | 150.13 |
| 248 | rs10491713 | T | G | 0.20 | -0.12 | 0.02 | 2.05E-08 | 31.56 |
| 249 | rs1243876 | T | C | 0.70 | -0.11 | 0.02 | 2.14E-08 | 31.30 |
| 250 | rs76452347 | T | C | 0.21 | -0.22 | 0.02 | 9.37E-23 | 96.19 |
| 251 | rs507666 | A | G | 0.19 | -0.29 | 0.02 | 2.27E-37 | 163.79 |
| 252 | rs11145807 | G | A | 0.59 | -0.16 | 0.02 | 4.10E-17 | 70.96 |
| 253 | rs12337056 | T | C | 0.18 | 0.14 | 0.02 | 2.18E-09 | 35.79 |
| 254 | rs1332812 | A | T | 0.65 | -0.11 | 0.02 | 2.71E-10 | 40.02 |
| 255 | rs11141731 | T | C | 0.23 | -0.13 | 0.02 | 1.31E-09 | 36.93 |
| 256 | rs10980408 | C | T | 0.04 | 0.37 | 0.05 | 4.17E-15 | 61.64 |
| 257 | rs2236295 | T | G | 0.40 | -0.21 | 0.02 | 1.42E-31 | 136.77 |
| 258 | rs12247028 | A | G | 0.63 | -0.14 | 0.02 | 1.18E-13 | 55.14 |
| 259 | rs2273654 | C | T | 0.44 | -0.12 | 0.02 | 2.75E-11 | 44.32 |
| 260 | rs2484294 | A | G | 0.73 | 0.32 | 0.02 | 1.17E-58 | 260.76 |
| 261 | rs11592107 | A | G | 0.31 | 0.12 | 0.02 | 1.23E-10 | 41.39 |
| 262 | rs10490923 | A | G | 0.13 | 0.15 | 0.03 | 5.02E-09 | 34.24 |
| 263 | rs1133400 | G | A | 0.21 | 0.13 | 0.02 | 8.30E-10 | 37.58 |
| 264 | rs6602177 | T | C | 0.71 | -0.12 | 0.02 | 6.52E-09 | 33.77 |
| 265 | rs12258967 | G | C | 0.30 | -0.35 | 0.02 | 3.27E-75 | 336.43 |
| 266 | rs2067831 | C | G | 0.27 | -0.13 | 0.02 | 5.08E-11 | 43.09 |
| 267 | rs1623474 | T | C | 0.33 | 0.22 | 0.02 | 6.24E-34 | 147.41 |
| 268 | rs3006583 | C | T | 0.19 | 0.13 | 0.02 | 4.66E-09 | 34.45 |
| 269 | rs4284362 | A | C | 0.72 | -0.16 | 0.02 | 3.24E-16 | 66.83 |
| 270 | rs11187838 | A | G | 0.43 | -0.28 | 0.02 | 2.55E-57 | 255.45 |
| 271 | rs11191580 | C | T | 0.08 | -0.51 | 0.03 | 6.63E-58 | 257.52 |
| 272 | rs11252324 | T | G | 0.08 | -0.23 | 0.03 | 1.03E-12 | 50.85 |
| 273 | rs3802517 | A | T | 0.46 | 0.13 | 0.02 | 9.29E-14 | 55.26 |
| 274 | rs34130368 | T | G | 0.12 | -0.20 | 0.03 | 8.77E-13 | 50.94 |
| 275 | rs9419374 | G | A | 0.65 | -0.12 | 0.02 | 3.44E-10 | 39.59 |
| 276 | rs1265842 | C | T | 0.52 | -0.11 | 0.02 | 1.70E-10 | 40.92 |
| 277 | rs2487926 | G | A | 0.43 | -0.10 | 0.02 | 3.31E-08 | 30.50 |
| 278 | rs72831343 | G | T | 0.14 | -0.49 | 0.02 | 4.77E-88 | 396.14 |
| 279 | rs35506078 | C | T | 0.34 | 0.13 | 0.02 | 1.54E-13 | 54.26 |
| 280 | rs1006545 | T | G | 0.89 | 0.36 | 0.03 | 7.96E-40 | 174.53 |
| 281 | rs72842207 | T | C | 0.21 | -0.21 | 0.02 | 1.10E-23 | 100.19 |
| 282 | rs4756779 | G | A | 0.18 | 0.15 | 0.02 | 1.48E-10 | 41.14 |
| 283 | rs10500932 | A | G | 0.07 | 0.28 | 0.03 | 5.79E-17 | 69.90 |
| 284 | rs11245631 | T | C | 0.20 | -0.15 | 0.02 | 3.99E-11 | 43.69 |
| 285 | rs7115331 | G | T | 0.29 | 0.13 | 0.02 | 3.91E-11 | 43.48 |
| 286 | rs604723 | C | T | 0.72 | 0.38 | 0.02 | 2.32E-87 | 393.43 |
| 287 | rs7938342 | A | T | 0.59 | 0.27 | 0.02 | 1.92E-49 | 217.93 |
| 288 | rs173396 | A | G | 0.58 | 0.21 | 0.02 | 1.38E-34 | 150.38 |
| 289 | rs10838702 | T | G | 0.39 | 0.24 | 0.02 | 1.27E-40 | 178.03 |
| 290 | rs11231693 | A | G | 0.06 | 0.22 | 0.04 | 4.02E-09 | 34.59 |
| 291 | rs2256187 | A | G | 0.16 | -0.14 | 0.02 | 1.39E-09 | 36.61 |
| 292 | rs11021221 | A | T | 0.17 | -0.19 | 0.02 | 6.93E-16 | 64.90 |
| 293 | rs79889784 | T | G | 0.02 | -0.39 | 0.07 | 3.86E-08 | 30.21 |
| 294 | rs145422110 | T | C | 0.02 | 0.54 | 0.07 | 6.41E-13 | 51.69 |
| 295 | rs12363520 | A | T | 0.23 | 0.17 | 0.02 | 4.24E-15 | 61.62 |
| 296 | rs962369 | C | T | 0.30 | -0.17 | 0.02 | 6.02E-19 | 79.39 |
| 297 | rs7933758 | T | C | 0.30 | -0.11 | 0.02 | 2.58E-09 | 35.50 |
| 298 | rs4930295 | G | C | 0.22 | -0.24 | 0.02 | 5.54E-31 | 133.85 |
| 299 | rs7926335 | T | C | 0.27 | 0.18 | 0.02 | 2.05E-20 | 85.59 |
| 300 | rs61909958 | G | C | 0.19 | -0.13 | 0.02 | 2.21E-08 | 31.27 |
| 301 | rs66682451 | G | A | 0.27 | -0.13 | 0.02 | 3.44E-12 | 48.28 |
| 302 | rs7106104 | C | T | 0.28 | 0.12 | 0.02 | 7.72E-10 | 37.76 |
| 303 | rs12790943 | T | C | 0.42 | -0.10 | 0.02 | 1.14E-08 | 32.78 |
| 304 | rs12574332 | T | C | 0.12 | 0.21 | 0.03 | 6.14E-15 | 60.68 |
| 305 | rs4936099 | A | C | 0.60 | 0.17 | 0.02 | 1.16E-22 | 96.11 |
| 306 | rs10832586 | C | A | 0.20 | 0.31 | 0.02 | 2.53E-46 | 203.72 |
| 307 | rs751984 | C | T | 0.12 | -0.39 | 0.03 | 1.38E-46 | 204.96 |
| 308 | rs504217 | T | C | 0.07 | 0.27 | 0.03 | 2.51E-16 | 67.14 |
| 309 | rs7134440 | T | C | 0.08 | 0.23 | 0.03 | 1.75E-12 | 49.74 |
| 310 | rs9368 | A | C | 0.39 | 0.11 | 0.02 | 3.24E-10 | 39.66 |
| 311 | rs1732664 | C | T | 0.68 | 0.11 | 0.02 | 2.58E-08 | 31.16 |
| 312 | rs7137828 | T | C | 0.52 | -0.50 | 0.02 | 4.80E-180 | 815.82 |
| 313 | rs7299936 | A | G | 0.58 | 0.18 | 0.02 | 1.11E-23 | 101.03 |
| 314 | rs61912333 | G | C | 0.50 | -0.12 | 0.02 | 1.13E-11 | 45.79 |
| 315 | rs6487076 | G | A | 0.22 | -0.17 | 0.02 | 8.69E-17 | 69.31 |
| 316 | rs11112548 | T | A | 0.04 | -0.27 | 0.04 | 5.80E-10 | 38.31 |
| 317 | rs4306343 | T | A | 0.72 | 0.32 | 0.02 | 8.22E-61 | 269.78 |
| 318 | rs6580970 | T | C | 0.30 | -0.17 | 0.02 | 4.03E-18 | 75.63 |
| 319 | rs520592 | G | T | 0.14 | 0.17 | 0.02 | 3.51E-12 | 48.49 |
| 320 | rs75507123 | T | G | 0.13 | -0.14 | 0.03 | 3.94E-08 | 30.19 |
| 321 | rs7132012 | G | A | 0.33 | -0.16 | 0.02 | 3.17E-17 | 71.38 |
| 322 | rs2681485 | A | G | 0.60 | 0.29 | 0.02 | 1.31E-62 | 279.99 |
| 323 | rs1948151 | A | G | 0.28 | -0.14 | 0.02 | 2.12E-12 | 49.29 |
| 324 | rs1669907 | G | T | 0.70 | -0.12 | 0.02 | 1.36E-09 | 36.76 |
| 325 | rs61917655 | T | C | 0.10 | 0.22 | 0.03 | 3.72E-14 | 57.19 |
| 326 | rs7967705 | C | T | 0.62 | -0.27 | 0.02 | 1.54E-51 | 229.06 |
| 327 | rs7959649 | C | T | 0.76 | -0.12 | 0.02 | 8.14E-09 | 33.32 |
| 328 | rs710698 | G | A | 0.41 | -0.11 | 0.02 | 1.88E-09 | 36.20 |
| 329 | rs11108209 | C | T | 0.09 | 0.19 | 0.03 | 2.40E-10 | 40.15 |
| 330 | rs116063464 | A | G | 0.06 | 0.20 | 0.04 | 4.68E-08 | 29.88 |
| 331 | rs35443 | C | G | 0.39 | -0.27 | 0.02 | 1.20E-50 | 223.49 |
| 332 | rs1790123 | T | C | 0.80 | 0.20 | 0.02 | 6.87E-20 | 83.41 |
| 333 | rs2271139 | A | C | 0.29 | -0.12 | 0.02 | 8.23E-11 | 42.18 |
| 334 | rs682681 | C | T | 0.67 | 0.15 | 0.02 | 4.47E-15 | 61.77 |
| 335 | rs56256111 | A | G | 0.14 | 0.19 | 0.03 | 2.60E-13 | 53.63 |
| 336 | rs7992292 | A | G | 0.82 | 0.14 | 0.02 | 3.19E-09 | 35.02 |
| 337 | rs544012 | G | T | 0.73 | -0.11 | 0.02 | 1.37E-08 | 32.34 |
| 338 | rs7321688 | A | C | 0.23 | 0.15 | 0.02 | 1.99E-13 | 54.04 |
| 339 | rs7324697 | A | C | 0.33 | 0.10 | 0.02 | 1.59E-08 | 32.03 |
| 340 | rs1215469 | C | A | 0.77 | 0.14 | 0.02 | 5.23E-11 | 42.96 |
| 341 | rs9526707 | A | G | 0.32 | -0.12 | 0.02 | 6.59E-11 | 42.81 |
| 342 | rs12866098 | A | G | 0.34 | 0.10 | 0.02 | 2.73E-08 | 30.84 |
| 343 | rs55684003 | G | A | 0.30 | -0.12 | 0.02 | 1.01E-10 | 41.67 |
| 344 | rs61948065 | C | A | 0.12 | 0.17 | 0.03 | 1.17E-10 | 41.39 |
| 345 | rs9508495 | T | C | 0.76 | -0.19 | 0.02 | 1.34E-21 | 90.81 |
| 346 | rs3861113 | A | C | 0.08 | 0.21 | 0.03 | 3.95E-11 | 43.59 |
| 347 | rs36169093 | A | G | 0.50 | 0.12 | 0.02 | 5.56E-12 | 47.29 |
| 348 | rs4424827 | T | C | 0.57 | -0.10 | 0.02 | 2.11E-08 | 31.42 |
| 349 | rs227426 | T | G | 0.56 | 0.11 | 0.02 | 1.75E-10 | 40.89 |
| 350 | rs7350752 | A | G | 0.12 | -0.15 | 0.03 | 1.97E-08 | 31.49 |
| 351 | rs1950500 | C | T | 0.71 | -0.14 | 0.02 | 2.20E-13 | 53.98 |
| 352 | rs7155504 | C | T | 0.09 | -0.23 | 0.03 | 5.16E-13 | 52.00 |
| 353 | rs35413927 | G | A | 0.30 | 0.13 | 0.02 | 1.77E-11 | 45.44 |
| 354 | rs194742 | C | T | 0.83 | -0.13 | 0.02 | 3.17E-08 | 30.49 |
| 355 | rs72683923 | C | T | 0.02 | -0.53 | 0.06 | 5.02E-17 | 70.32 |
| 356 | rs4903064 | C | T | 0.24 | -0.15 | 0.02 | 7.84E-14 | 56.10 |
| 357 | rs8014182 | T | C | 0.13 | -0.19 | 0.03 | 3.94E-14 | 57.10 |
| 358 | rs17880989 | A | G | 0.03 | 0.40 | 0.06 | 1.11E-11 | 46.13 |
| 359 | rs2239268 | A | G | 0.70 | 0.11 | 0.02 | 7.40E-09 | 33.34 |
| 360 | rs2305654 | A | C | 0.34 | 0.17 | 0.02 | 3.99E-20 | 84.23 |
| 361 | rs28429256 | A | G | 0.33 | 0.16 | 0.02 | 2.83E-18 | 75.73 |
| 362 | rs11636952 | C | T | 0.69 | -0.40 | 0.02 | 5.21E-99 | 447.24 |
| 363 | rs2627313 | T | C | 0.45 | 0.15 | 0.02 | 5.85E-18 | 74.45 |
| 364 | rs2589218 | C | T | 0.27 | 0.12 | 0.02 | 6.90E-10 | 37.92 |
| 365 | rs11070245 | G | T | 0.53 | 0.13 | 0.02 | 1.57E-13 | 54.71 |
| 366 | rs4932373 | C | A | 0.33 | 0.37 | 0.02 | 7.71E-84 | 375.83 |
| 367 | rs2925345 | C | T | 0.53 | -0.19 | 0.02 | 1.60E-27 | 117.98 |
| 368 | rs12906962 | C | T | 0.32 | 0.24 | 0.02 | 8.73E-37 | 160.00 |
| 369 | rs10873612 | T | C | 0.60 | -0.11 | 0.02 | 9.51E-10 | 37.49 |
| 370 | rs2469141 | C | T | 0.16 | -0.14 | 0.02 | 1.39E-08 | 32.22 |
| 371 | rs3743111 | A | G | 0.61 | 0.15 | 0.02 | 1.62E-17 | 72.63 |
| 372 | rs57708073 | G | A | 0.26 | -0.19 | 0.02 | 4.73E-19 | 79.41 |
| 373 | rs17807723 | A | G | 0.14 | -0.18 | 0.03 | 7.44E-12 | 46.74 |
| 374 | rs3743369 | A | G | 0.63 | 0.10 | 0.02 | 6.82E-09 | 33.76 |
| 375 | rs12596630 | T | C | 0.09 | 0.26 | 0.03 | 1.03E-16 | 68.88 |
| 376 | rs12929303 | A | G | 0.53 | 0.16 | 0.02 | 1.58E-19 | 81.62 |
| 377 | rs1049212 | G | A | 0.57 | 0.18 | 0.02 | 1.30E-24 | 104.39 |
| 378 | rs9932220 | A | G | 0.22 | -0.16 | 0.02 | 3.76E-14 | 57.40 |
| 379 | rs908951 | T | C | 0.44 | -0.20 | 0.02 | 7.73E-28 | 120.03 |
| 380 | rs9937801 | C | T | 0.43 | -0.16 | 0.02 | 4.81E-19 | 79.76 |
| 381 | rs7192407 | C | T | 0.53 | -0.10 | 0.02 | 4.53E-09 | 34.30 |
| 382 | rs17696749 | G | C | 0.59 | 0.13 | 0.02 | 9.43E-13 | 50.85 |
| 383 | rs62030049 | G | A | 0.24 | -0.13 | 0.02 | 1.55E-10 | 40.86 |
| 384 | rs12149254 | A | G | 0.17 | -0.13 | 0.02 | 1.14E-08 | 32.57 |
| 385 | rs11859505 | G | A | 0.58 | 0.10 | 0.02 | 9.76E-09 | 32.82 |
| 386 | rs79286081 | A | G | 0.10 | -0.16 | 0.03 | 4.83E-08 | 29.76 |
| 387 | rs917522 | T | C | 0.89 | 0.17 | 0.03 | 1.04E-09 | 37.20 |
| 388 | rs77924615 | A | G | 0.20 | -0.32 | 0.02 | 3.72E-45 | 199.39 |
| 389 | rs80095680 | G | A | 0.26 | 0.16 | 0.02 | 2.81E-15 | 62.55 |
| 390 | rs12919839 | T | C | 0.28 | -0.11 | 0.02 | 1.04E-08 | 32.70 |
| 391 | rs45474499 | T | C | 0.05 | 0.36 | 0.04 | 8.50E-18 | 73.67 |
| 392 | rs28544928 | G | T | 0.25 | -0.15 | 0.02 | 9.13E-15 | 60.12 |
| 393 | rs4362428 | A | C | 0.41 | -0.11 | 0.02 | 1.45E-10 | 41.00 |
| 394 | rs9900637 | A | C | 0.50 | 0.10 | 0.02 | 1.73E-08 | 31.77 |
| 395 | rs138420351 | T | C | 0.02 | 0.56 | 0.09 | 7.11E-11 | 42.51 |
| 396 | rs74439044 | C | T | 0.10 | 0.35 | 0.03 | 1.38E-32 | 141.40 |
| 397 | rs79724577 | C | A | 0.18 | -0.14 | 0.02 | 3.47E-09 | 35.07 |
| 398 | rs3916033 | T | C | 0.56 | -0.12 | 0.02 | 2.41E-11 | 44.42 |
| 399 | rs3785837 | A | G | 0.76 | 0.15 | 0.02 | 9.57E-12 | 46.53 |
| 400 | rs55868524 | A | G | 0.61 | 0.14 | 0.02 | 5.54E-16 | 65.72 |
| 401 | rs9893005 | G | C | 0.46 | 0.12 | 0.02 | 7.90E-12 | 46.88 |
| 402 | rs9889262 | A | T | 0.37 | 0.23 | 0.02 | 7.11E-37 | 160.87 |
| 403 | rs4295 | G | C | 0.62 | -0.18 | 0.02 | 1.71E-23 | 100.22 |
| 404 | rs7217916 | G | A | 0.61 | -0.11 | 0.02 | 5.63E-10 | 38.52 |
| 405 | rs76954792 | T | C | 0.23 | 0.12 | 0.02 | 5.06E-09 | 34.01 |
| 406 | rs28661492 | T | C | 0.20 | -0.14 | 0.02 | 9.56E-10 | 37.47 |
| 407 | rs11077961 | G | A | 0.37 | -0.11 | 0.02 | 8.55E-09 | 33.28 |
| 408 | rs12938803 | C | A | 0.81 | 0.16 | 0.02 | 8.76E-13 | 50.89 |
| 409 | rs2239917 | C | T | 0.57 | -0.17 | 0.02 | 9.69E-23 | 96.73 |
| 410 | rs8078510 | A | G | 0.27 | -0.13 | 0.02 | 9.84E-11 | 42.02 |
| 411 | rs1867624 | T | C | 0.61 | 0.14 | 0.02 | 2.08E-15 | 62.93 |
| 412 | rs1436138 | G | A | 0.36 | -0.20 | 0.02 | 7.33E-28 | 119.67 |
| 413 | rs58693787 | G | A | 0.25 | -0.16 | 0.02 | 3.82E-15 | 61.49 |
| 414 | rs1523871 | G | C | 0.43 | 0.12 | 0.02 | 1.46E-11 | 45.41 |
| 415 | rs11661473 | A | G | 0.27 | 0.20 | 0.02 | 1.54E-24 | 104.85 |
| 416 | rs4890499 | A | G | 0.25 | 0.11 | 0.02 | 1.33E-08 | 32.30 |
| 417 | rs11665020 | C | G | 0.32 | -0.14 | 0.02 | 2.78E-14 | 57.91 |
| 418 | rs10048404 | T | C | 0.37 | -0.11 | 0.02 | 2.00E-09 | 35.87 |
| 419 | rs7235890 | T | G | 0.90 | -0.17 | 0.03 | 4.12E-09 | 34.52 |
| 420 | rs34413141 | A | T | 0.18 | -0.18 | 0.02 | 1.49E-15 | 63.44 |
| 421 | rs1903752 | T | C | 0.54 | -0.10 | 0.02 | 3.20E-08 | 30.75 |
| 422 | rs11664194 | A | T | 0.46 | -0.11 | 0.02 | 8.69E-10 | 37.52 |
| 423 | rs4891258 | G | A | 0.32 | 0.12 | 0.02 | 5.72E-10 | 38.41 |
| 424 | rs10164193 | G | T | 0.08 | 0.22 | 0.03 | 1.87E-11 | 45.10 |
| 425 | rs12609484 | T | G | 0.32 | -0.14 | 0.02 | 1.16E-13 | 55.30 |
| 426 | rs73046792 | A | G | 0.16 | -0.15 | 0.02 | 5.87E-10 | 38.39 |
| 427 | rs73036520 | C | G | 0.25 | 0.16 | 0.02 | 1.34E-14 | 59.41 |
| 428 | rs2548459 | C | T | 0.52 | 0.13 | 0.02 | 5.95E-14 | 56.25 |
| 429 | rs7259285 | A | G | 0.45 | 0.11 | 0.02 | 2.65E-09 | 35.59 |
| 430 | rs3761077 | T | G | 0.11 | 0.17 | 0.03 | 1.04E-09 | 37.20 |
| 431 | rs7257694 | T | C | 0.40 | 0.18 | 0.02 | 6.28E-25 | 106.51 |
| 432 | rs1390754 | T | C | 0.40 | 0.13 | 0.02 | 1.19E-12 | 50.51 |
| 433 | rs12978472 | G | C | 0.12 | -0.48 | 0.03 | 8.46E-65 | 289.24 |
| 434 | rs318712 | C | T | 0.08 | 0.24 | 0.03 | 9.17E-13 | 51.16 |
| 435 | rs2291516 | A | G | 0.10 | 0.24 | 0.03 | 9.88E-17 | 68.80 |
| 436 | rs1433121 | T | C | 0.69 | -0.14 | 0.02 | 6.91E-13 | 51.72 |
| 437 | rs6108168 | A | C | 0.25 | -0.19 | 0.02 | 1.10E-21 | 91.26 |
| 438 | rs672272 | T | C | 0.60 | -0.19 | 0.02 | 2.77E-25 | 108.14 |
| 439 | rs6058261 | A | C | 0.27 | -0.12 | 0.02 | 6.83E-10 | 37.93 |
| 440 | rs34587839 | A | G | 0.15 | -0.17 | 0.02 | 8.22E-12 | 46.79 |
| 441 | rs6031431 | G | A | 0.46 | 0.12 | 0.02 | 4.94E-11 | 43.41 |
| 442 | rs4814837 | T | C | 0.34 | -0.10 | 0.02 | 4.62E-08 | 29.71 |
| 443 | rs1327235 | G | A | 0.47 | 0.30 | 0.02 | 4.76E-68 | 304.33 |
| 444 | rs6078393 | G | T | 0.41 | -0.12 | 0.02 | 7.66E-12 | 46.88 |
| 445 | rs234616 | A | G | 0.31 | -0.12 | 0.02 | 8.79E-10 | 37.55 |
| 446 | rs6026739 | T | A | 0.12 | 0.50 | 0.03 | 1.49E-79 | 357.86 |
| 447 | rs7265695 | C | T | 0.20 | -0.20 | 0.02 | 2.48E-19 | 80.67 |
| 448 | rs2598 | G | A | 0.47 | -0.14 | 0.02 | 1.94E-15 | 62.82 |
| 449 | rs79044887 | G | C | 0.15 | -0.24 | 0.02 | 4.01E-23 | 98.13 |
| 450 | rs6062477 | T | C | 0.58 | 0.12 | 0.02 | 1.81E-11 | 45.28 |
| 451 | rs35213536 | T | G | 0.25 | 0.20 | 0.02 | 2.54E-23 | 99.42 |
| 452 | rs7278003 | C | T | 0.56 | 0.13 | 0.02 | 1.78E-13 | 53.97 |
| 453 | rs12627514 | G | C | 0.29 | 0.22 | 0.02 | 1.99E-28 | 121.90 |
| 454 | rs34487963 | A | C | 0.02 | -0.57 | 0.07 | 8.18E-16 | 64.86 |
| 455 | rs2070527 | C | A | 0.75 | 0.15 | 0.02 | 3.08E-13 | 53.17 |
| 456 | rs1882961 | T | C | 0.31 | 0.13 | 0.02 | 1.40E-11 | 45.78 |
| 457 | rs12321 | C | G | 0.43 | -0.15 | 0.02 | 1.44E-17 | 72.69 |
| 458 | rs135023 | G | A | 0.58 | 0.10 | 0.02 | 3.53E-09 | 35.05 |
| 459 | rs5992929 | T | C | 0.28 | 0.17 | 0.02 | 3.07E-18 | 76.13 |
| 460 | rs926335 | T | C | 0.48 | -0.12 | 0.02 | 3.37E-12 | 48.20 |
|  |  |  |  |  |  |  |  |  |

EAF: Effect allele frequency; DBP: Diastolic blood pressure

Table 5: Characteristics of SNPs used as instrumental variables for TG

|  | SNP | effect_allele.exposure | other_allele.exposure | eaf.exposure | beta.exposure | se.exposure | pval.exposure | F-Statistics |
| --- | --- | --- | --- | --- | --- | --- | --- | --- |
| 1 | rs12119979 | G | C | 0.46 | 0.02 | 0.00 | 5.26E-19 | 79.34 |
| 2 | rs114052230 | T | C | 0.17 | -0.02 | 0.00 | 1.10E-13 | 55.18 |
| 3 | rs2114273 | C | T | 0.60 | 0.02 | 0.00 | 5.23E-13 | 52.12 |
| 4 | rs213479 | T | G | 0.47 | -0.02 | 0.00 | 1.22E-10 | 41.44 |
| 5 | rs10631642 | CTTT | C | 0.31 | -0.01 | 0.00 | 1.03E-08 | 32.79 |
| 6 | rs1044808 | C | G | 0.08 | -0.03 | 0.00 | 2.06E-11 | 44.92 |
| 7 | rs12088739 | G | A | 0.09 | -0.03 | 0.00 | 1.36E-11 | 45.73 |
| 8 | rs5005705 | A | C | 0.83 | -0.02 | 0.00 | 4.85E-11 | 43.24 |
| 9 | rs1009590 | C | G | 0.91 | 0.02 | 0.00 | 3.25E-09 | 35.03 |
| 10 | rs7529073 | C | T | 0.46 | -0.01 | 0.00 | 2.42E-08 | 31.13 |
| 11 | rs631106 | A | C | 0.35 | -0.08 | 0.00 | 1.00E-200 | 1103.93 |
| 12 | rs11122450 | G | T | 0.61 | -0.05 | 0.00 | 1.60E-88 | 398.09 |
| 13 | rs114165349 | C | G | 0.02 | 0.09 | 0.01 | 4.14E-29 | 125.43 |
| 14 | rs11206374 | A | G | 0.22 | 0.03 | 0.00 | 1.49E-19 | 81.82 |
| 15 | rs12749691 | T | A | 0.30 | -0.02 | 0.00 | 2.22E-17 | 71.95 |
| 16 | rs9425589 | A | G | 0.57 | -0.01 | 0.00 | 6.44E-09 | 33.70 |
| 17 | rs17311740 | T | C | 0.07 | -0.03 | 0.00 | 1.17E-08 | 32.54 |
| 18 | rs11688682 | C | G | 0.27 | -0.02 | 0.00 | 7.94E-10 | 37.78 |
| 19 | rs1260326 | C | T | 0.61 | -0.10 | 0.00 | 1.00E-200 | 1915.13 |
| 20 | rs870526 | T | C | 0.52 | -0.02 | 0.00 | 6.72E-15 | 60.69 |
| 21 | rs17326656 | T | G | 0.24 | 0.02 | 0.00 | 2.60E-09 | 35.47 |
| 22 | rs1420384 | T | G | 0.67 | -0.01 | 0.00 | 5.40E-09 | 34.04 |
| 23 | rs676210 | A | G | 0.20 | -0.08 | 0.00 | 3.02E-156 | 709.91 |
| 24 | rs1128249 | T | G | 0.39 | -0.04 | 0.00 | 5.70E-55 | 243.94 |
| 25 | rs10210970 | T | C | 0.13 | 0.03 | 0.00 | 5.20E-18 | 74.81 |
| 26 | rs2723067 | G | A | 0.41 | -0.02 | 0.00 | 4.57E-17 | 70.52 |
| 27 | rs71401561 | CT | C | 0.48 | 0.02 | 0.00 | 4.15E-11 | 43.54 |
| 28 | rs77342729 | G | A | 0.03 | 0.04 | 0.01 | 7.12E-09 | 33.50 |
| 29 | rs35602378 | CT | C | 0.43 | -0.02 | 0.00 | 2.42E-17 | 71.77 |
| 30 | rs73948269 | G | A | 0.02 | 0.06 | 0.01 | 2.06E-09 | 35.92 |
| 31 | rs3731696 | G | A | 0.12 | 0.02 | 0.00 | 6.96E-09 | 33.55 |
| 32 | rs35549624 | CA | C | 0.49 | -0.01 | 0.00 | 1.33E-08 | 32.29 |
| 33 | rs4675812 | A | G | 0.59 | -0.01 | 0.00 | 2.43E-08 | 31.11 |
| 34 | rs11442987 | TA | T | 0.55 | -0.01 | 0.00 | 2.81E-08 | 30.84 |
| 35 | rs2943645 | T | C | 0.65 | 0.04 | 0.00 | 5.15E-62 | 276.29 |
| 36 | rs6708784 | G | A | 0.50 | -0.01 | 0.00 | 1.73E-08 | 31.78 |
| 37 | rs377352335 | AT | A | 0.34 | -0.01 | 0.00 | 3.32E-08 | 30.51 |
| 38 | rs17052058 | G | A | 0.18 | -0.03 | 0.00 | 2.95E-26 | 112.40 |
| 39 | rs79287178 | A | G | 0.03 | 0.05 | 0.01 | 4.13E-15 | 61.64 |
| 40 | rs13066793 | G | A | 0.09 | -0.02 | 0.00 | 1.66E-08 | 31.86 |
| 41 | rs9836434 | T | C | 0.24 | 0.01 | 0.00 | 3.63E-08 | 30.34 |
| 42 | rs1279840 | C | T | 0.75 | 0.03 | 0.00 | 1.71E-25 | 108.92 |
| 43 | rs58284370 | A | G | 0.10 | 0.03 | 0.00 | 1.12E-11 | 46.10 |
| 44 | rs4647214 | AT | A | 0.42 | -0.01 | 0.00 | 3.85E-09 | 34.70 |
| 45 | rs34389637 | CT | C | 0.07 | -0.03 | 0.00 | 5.19E-10 | 38.61 |
| 46 | rs9844972 | C | G | 0.07 | 0.04 | 0.00 | 8.50E-20 | 82.94 |
| 47 | rs3105363 | G | A | 0.23 | 0.02 | 0.00 | 1.43E-16 | 68.27 |
| 48 | rs6792725 | G | A | 0.69 | -0.02 | 0.00 | 5.38E-10 | 38.54 |
| 49 | rs7631606 | G | T | 0.27 | -0.02 | 0.00 | 3.96E-09 | 34.64 |
| 50 | rs1471251 | T | A | 0.40 | 0.04 | 0.00 | 4.21E-59 | 262.91 |
| 51 | rs293435 | T | C | 0.29 | 0.02 | 0.00 | 3.58E-12 | 48.35 |
| 52 | rs12513202 | T | C | 0.60 | 0.01 | 0.00 | 1.33E-08 | 32.28 |
| 53 | rs71603401 | G | A | 0.14 | 0.03 | 0.00 | 7.25E-15 | 60.54 |
| 54 | rs11722924 | C | G | 0.54 | 0.01 | 0.00 | 5.84E-09 | 33.89 |
| 55 | rs13108218 | G | A | 0.62 | -0.03 | 0.00 | 1.10E-39 | 173.84 |
| 56 | rs35225200 | C | A | 0.08 | 0.03 | 0.00 | 4.85E-13 | 52.27 |
| 57 | rs10470884 | A | G | 0.23 | -0.02 | 0.00 | 4.71E-09 | 34.31 |
| 58 | rs6554198 | A | G | 0.59 | -0.01 | 0.00 | 1.82E-08 | 31.68 |
| 59 | rs3822072 | A | G | 0.45 | 0.02 | 0.00 | 9.86E-13 | 50.88 |
| 60 | rs2139980 | A | G | 0.37 | -0.01 | 0.00 | 7.84E-10 | 37.80 |
| 61 | rs73243877 | G | A | 0.17 | 0.03 | 0.00 | 4.22E-19 | 79.77 |
| 62 | rs6882076 | C | T | 0.64 | 0.04 | 0.00 | 7.25E-51 | 225.09 |
| 63 | rs4976033 | G | A | 0.40 | 0.02 | 0.00 | 4.01E-15 | 61.70 |
| 64 | rs72801474 | A | G | 0.09 | -0.03 | 0.00 | 4.48E-15 | 61.48 |
| 65 | rs593979 | C | T | 0.40 | -0.02 | 0.00 | 4.90E-15 | 61.30 |
| 66 | rs112424890 | T | C | 0.18 | 0.02 | 0.00 | 1.86E-09 | 36.12 |
| 67 | rs10040328 | A | C | 0.26 | 0.02 | 0.00 | 6.62E-09 | 33.65 |
| 68 | rs1045241 | T | C | 0.27 | -0.02 | 0.00 | 1.85E-17 | 72.31 |
| 69 | rs34580448 | C | T | 0.04 | -0.04 | 0.01 | 3.59E-10 | 39.32 |
| 70 | rs11429307 | GT | G | 0.19 | 0.05 | 0.00 | 2.20E-55 | 245.83 |
| 71 | rs7735249 | G | C | 0.11 | 0.03 | 0.00 | 8.82E-13 | 51.09 |
| 72 | rs154735 | A | G | 0.06 | 0.03 | 0.00 | 5.01E-09 | 34.19 |
| 73 | rs1644005 | C | T | 0.36 | -0.01 | 0.00 | 2.64E-09 | 35.43 |
| 74 | rs142047875 | T | A | 0.57 | -0.01 | 0.00 | 1.64E-08 | 31.88 |
| 75 | rs28752924 | C | T | 0.44 | 0.03 | 0.00 | 3.19E-39 | 171.71 |
| 76 | rs28359800 | C | CAA | 0.28 | 0.03 | 0.00 | 1.62E-30 | 131.87 |
| 77 | rs186696265 | T | C | 0.01 | -0.10 | 0.01 | 1.95E-26 | 113.23 |
| 78 | rs73025562 | A | G | 0.25 | 0.02 | 0.00 | 5.12E-09 | 34.15 |
| 79 | rs10872003 | A | T | 0.45 | -0.01 | 0.00 | 2.17E-08 | 31.33 |
| 80 | rs28383314 | C | T | 0.62 | 0.04 | 0.00 | 3.42E-66 | 295.47 |
| 81 | rs6916318 | T | A | 0.53 | 0.03 | 0.00 | 3.01E-29 | 126.07 |
| 82 | rs707931 | G | A | 0.06 | 0.03 | 0.00 | 4.50E-11 | 43.39 |
| 83 | rs9264277 | C | T | 0.63 | 0.01 | 0.00 | 1.84E-09 | 36.14 |
| 84 | rs199607859 | T | G | 0.59 | -0.03 | 0.00 | 2.53E-35 | 153.86 |
| 85 | rs77009508 | G | A | 0.08 | 0.05 | 0.00 | 1.13E-24 | 105.16 |
| 86 | rs1064173 | A | G | 0.29 | -0.02 | 0.00 | 2.08E-20 | 85.72 |
| 87 | rs729761 | G | T | 0.71 | 0.02 | 0.00 | 3.31E-12 | 48.50 |
| 88 | rs4710938 | G | A | 0.46 | -0.01 | 0.00 | 6.15E-10 | 38.28 |
| 89 | rs1835346 | G | A | 0.02 | -0.04 | 0.01 | 1.98E-08 | 31.52 |
| 90 | rs998584 | A | C | 0.48 | 0.04 | 0.00 | 9.14E-66 | 293.49 |
| 91 | rs11752394 | G | C | 0.76 | 0.02 | 0.00 | 2.63E-10 | 39.94 |
| 92 | rs4731701 | T | C | 0.50 | -0.03 | 0.00 | 1.43E-47 | 209.98 |
| 93 | rs4722551 | C | T | 0.16 | -0.04 | 0.00 | 1.68E-31 | 136.36 |
| 94 | rs38205 | C | A | 0.62 | -0.01 | 0.00 | 7.09E-10 | 38.00 |
| 95 | rs852392 | A | G | 0.22 | 0.02 | 0.00 | 1.08E-08 | 32.68 |
| 96 | rs13234131 | G | A | 0.13 | -0.13 | 0.00 | 1.00E-200 | 1410.49 |
| 97 | rs72555385 | G | A | 0.05 | 0.07 | 0.01 | 6.24E-35 | 152.07 |
| 98 | rs17138358 | C | G | 0.40 | 0.02 | 0.00 | 4.38E-15 | 61.53 |
| 99 | rs2699805 | A | G | 0.40 | -0.02 | 0.00 | 7.68E-15 | 60.42 |
| 100 | rs9692598 | G | A | 0.51 | -0.01 | 0.00 | 3.82E-09 | 34.71 |
| 101 | rs2971669 | T | C | 0.22 | 0.02 | 0.00 | 7.38E-09 | 33.43 |
| 102 | rs2070971 | T | G | 0.14 | 0.02 | 0.00 | 2.96E-13 | 53.24 |
| 103 | rs917195 | T | C | 0.23 | -0.02 | 0.00 | 1.69E-08 | 31.82 |
| 104 | rs4410790 | C | T | 0.63 | 0.02 | 0.00 | 2.26E-11 | 44.74 |
| 105 | rs112206063 | C | T | 0.21 | 0.02 | 0.00 | 1.38E-10 | 41.19 |
| 106 | rs41749 | A | C | 0.45 | -0.01 | 0.00 | 1.42E-10 | 41.14 |
| 107 | rs17091412 | C | G | 0.24 | -0.02 | 0.00 | 2.30E-10 | 40.20 |
| 108 | rs11249894 | T | G | 0.17 | 0.02 | 0.00 | 4.89E-08 | 29.76 |
| 109 | rs73224072 | C | G | 0.19 | 0.02 | 0.00 | 5.59E-09 | 33.97 |
| 110 | rs10957299 | G | T | 0.43 | -0.01 | 0.00 | 2.66E-08 | 30.94 |
| 111 | rs7826687 | G | C | 0.28 | 0.03 | 0.00 | 1.05E-36 | 160.20 |
| 112 | rs13280055 | A | G | 0.13 | 0.04 | 0.00 | 1.32E-29 | 127.70 |
| 113 | rs1561748 | C | G | 0.27 | -0.07 | 0.00 | 1.41E-123 | 559.43 |
| 114 | rs2081687 | C | T | 0.66 | -0.03 | 0.00 | 2.75E-26 | 112.54 |
| 115 | rs13269725 | G | A | 0.08 | 0.04 | 0.00 | 1.88E-16 | 67.73 |
| 116 | rs76308736 | A | G | 0.07 | 0.03 | 0.00 | 7.25E-14 | 56.00 |
| 117 | rs13275656 | C | T | 0.57 | 0.01 | 0.00 | 4.68E-08 | 29.85 |
| 118 | rs72657503 | C | A | 0.02 | 0.05 | 0.01 | 4.97E-08 | 29.73 |
| 119 | rs328 | G | C | 0.10 | -0.20 | 0.00 | 1.00E-200 | 2627.59 |
| 120 | rs28601761 | G | C | 0.42 | -0.10 | 0.00 | 1.00E-200 | 1703.13 |
| 121 | rs62493027 | C | T | 0.08 | 0.04 | 0.00 | 1.06E-16 | 68.85 |
| 122 | rs6996614 | A | C | 0.53 | -0.01 | 0.00 | 3.33E-09 | 34.98 |
| 123 | rs57994353 | C | T | 0.30 | 0.01 | 0.00 | 9.18E-09 | 33.01 |
| 124 | rs34081699 | A | G | 0.34 | -0.01 | 0.00 | 1.54E-08 | 32.01 |
| 125 | rs1924485 | T | G | 0.13 | -0.02 | 0.00 | 2.28E-09 | 35.72 |
| 126 | rs296884 | T | G | 0.26 | -0.02 | 0.00 | 1.07E-18 | 77.92 |
| 127 | rs10797119 | C | T | 0.54 | 0.02 | 0.00 | 1.68E-13 | 54.35 |
| 128 | rs550057 | T | C | 0.25 | -0.02 | 0.00 | 3.77E-13 | 52.77 |
| 129 | rs1853413 | G | C | 0.35 | -0.01 | 0.00 | 8.14E-09 | 33.25 |
| 130 | rs1800978 | G | C | 0.12 | -0.03 | 0.00 | 5.11E-13 | 52.17 |
| 131 | rs12686780 | T | C | 0.17 | 0.02 | 0.00 | 2.90E-08 | 30.77 |
| 132 | rs2068888 | A | G | 0.45 | -0.03 | 0.00 | 6.14E-40 | 174.99 |
| 133 | rs2773469 | G | A | 0.74 | -0.02 | 0.00 | 1.57E-14 | 59.02 |
| 134 | rs11187019 | G | A | 0.55 | -0.01 | 0.00 | 2.67E-08 | 30.94 |
| 135 | rs113344423 | A | G | 0.06 | 0.04 | 0.00 | 6.75E-18 | 74.29 |
| 136 | rs10883026 | T | C | 0.52 | -0.02 | 0.00 | 1.16E-10 | 41.54 |
| 137 | rs34931109 | TA | T | 0.24 | -0.02 | 0.00 | 2.80E-10 | 39.81 |
| 138 | rs1133400 | G | A | 0.22 | 0.02 | 0.00 | 1.68E-09 | 36.31 |
| 139 | rs7896783 | A | G | 0.47 | -0.03 | 0.00 | 1.21E-48 | 214.91 |
| 140 | rs76669111 | T | G | 0.16 | -0.02 | 0.00 | 4.75E-14 | 56.84 |
| 141 | rs74563318 | A | C | 0.04 | -0.05 | 0.01 | 1.24E-15 | 64.01 |
| 142 | rs11000468 | T | C | 0.25 | -0.02 | 0.00 | 3.76E-10 | 39.23 |
| 143 | rs17699425 | A | G | 0.06 | -0.03 | 0.00 | 1.54E-08 | 32.00 |
| 144 | rs34875072 | T | G | 0.04 | -0.04 | 0.01 | 4.60E-10 | 38.84 |
| 145 | rs6486122 | T | C | 0.69 | 0.02 | 0.00 | 2.04E-15 | 63.04 |
| 146 | rs144646355 | A | G | 0.02 | -0.06 | 0.01 | 1.09E-10 | 41.65 |
| 147 | rs4930352 | T | G | 0.50 | -0.01 | 0.00 | 5.48E-09 | 34.01 |
| 148 | rs1047964 | T | G | 0.01 | -0.24 | 0.01 | 8.47E-95 | 427.00 |
| 149 | rs10838681 | A | G | 0.27 | -0.03 | 0.00 | 8.65E-24 | 101.14 |
| 150 | rs141469619 | G | A | 0.01 | 0.34 | 0.01 | 2.00E-166 | 756.88 |
| 151 | rs12294913 | G | C | 0.05 | 0.06 | 0.01 | 2.69E-23 | 98.89 |
| 152 | rs883863 | A | G | 0.31 | 0.02 | 0.00 | 1.41E-20 | 86.49 |
| 153 | rs11030107 | G | A | 0.26 | 0.02 | 0.00 | 2.87E-10 | 39.76 |
| 154 | rs7117238 | A | G | 0.16 | -0.02 | 0.00 | 2.41E-09 | 35.61 |
| 155 | rs964184 | C | G | 0.87 | -0.26 | 0.00 | 1.00E-200 | 5899.42 |
| 156 | rs10750766 | A | C | 0.71 | 0.02 | 0.00 | 3.55E-17 | 71.02 |
| 157 | rs11600815 | A | G | 0.05 | -0.04 | 0.01 | 5.73E-12 | 47.43 |
| 158 | rs174574 | C | A | 0.65 | -0.05 | 0.00 | 1.47E-98 | 444.27 |
| 159 | rs7951019 | G | T | 0.03 | -0.04 | 0.01 | 3.87E-09 | 34.69 |
| 160 | rs12424054 | A | G | 0.23 | 0.02 | 0.00 | 3.83E-12 | 48.21 |
| 161 | rs2694913 | C | A | 0.64 | 0.01 | 0.00 | 6.43E-09 | 33.70 |
| 162 | rs11057837 | T | C | 0.10 | 0.02 | 0.00 | 1.28E-08 | 32.36 |
| 163 | rs199795230 | T | C | 0.16 | 0.03 | 0.00 | 6.77E-17 | 69.75 |
| 164 | rs4760254 | C | G | 0.24 | -0.03 | 0.00 | 8.18E-26 | 110.37 |
| 165 | rs11045171 | G | A | 0.20 | -0.03 | 0.00 | 1.54E-22 | 95.43 |
| 166 | rs4761234 | C | T | 0.49 | -0.02 | 0.00 | 2.29E-12 | 49.22 |
| 167 | rs1790099 | T | C | 0.71 | 0.02 | 0.00 | 8.40E-11 | 42.16 |
| 168 | rs7298844 | G | A | 0.21 | 0.02 | 0.00 | 3.62E-08 | 30.34 |
| 169 | rs863750 | T | C | 0.60 | 0.03 | 0.00 | 2.66E-35 | 153.76 |
| 170 | rs4930724 | C | T | 0.33 | -0.03 | 0.00 | 4.51E-27 | 116.12 |
| 171 | rs76895963 | G | T | 0.02 | -0.09 | 0.01 | 2.14E-24 | 103.91 |
| 172 | rs10772947 | G | A | 0.53 | -0.01 | 0.00 | 4.02E-09 | 34.62 |
| 173 | rs11274835 | CGAGTGTGGGAATCT | C | 0.17 | -0.03 | 0.00 | 1.03E-18 | 78.01 |
| 174 | rs1928496 | T | C | 0.74 | 0.02 | 0.00 | 3.01E-10 | 39.67 |
| 175 | rs6492721 | C | T | 0.69 | -0.01 | 0.00 | 2.92E-09 | 35.24 |
| 176 | rs7140110 | C | T | 0.30 | 0.03 | 0.00 | 9.05E-31 | 133.02 |
| 177 | rs2812208 | C | G | 0.02 | -0.05 | 0.01 | 1.74E-10 | 40.74 |
| 178 | rs1340819 | C | A | 0.35 | -0.01 | 0.00 | 3.42E-08 | 30.45 |
| 179 | rs12868517 | G | T | 0.25 | -0.01 | 0.00 | 4.79E-08 | 29.80 |
| 180 | rs61993685 | C | T | 0.08 | -0.02 | 0.00 | 3.22E-08 | 30.57 |
| 181 | rs12880341 | C | T | 0.16 | 0.02 | 0.00 | 1.62E-10 | 40.88 |
| 182 | rs139624990 | T | C | 0.01 | 0.07 | 0.01 | 4.18E-08 | 30.07 |
| 183 | rs11631625 | G | A | 0.26 | 0.01 | 0.00 | 2.64E-08 | 30.96 |
| 184 | rs8025505 | T | C | 0.26 | 0.02 | 0.00 | 1.00E-16 | 68.98 |
| 185 | rs3826043 | T | C | 0.43 | -0.01 | 0.00 | 7.52E-09 | 33.40 |
| 186 | rs2652812 | T | C | 0.77 | -0.02 | 0.00 | 3.76E-11 | 43.74 |
| 187 | rs10851698 | T | C | 0.26 | 0.02 | 0.00 | 1.06E-09 | 37.22 |
| 188 | rs139974673 | C | T | 0.02 | 0.14 | 0.01 | 8.71E-84 | 376.32 |
| 189 | rs261342 | C | G | 0.78 | -0.05 | 0.00 | 8.05E-63 | 279.97 |
| 190 | rs422137 | A | G | 0.44 | 0.03 | 0.00 | 3.21E-29 | 125.94 |
| 191 | rs7167078 | G | C | 0.31 | -0.02 | 0.00 | 8.52E-10 | 37.64 |
| 192 | rs34682685 | A | G | 0.11 | 0.03 | 0.00 | 2.97E-18 | 75.92 |
| 193 | rs8051062 | C | T | 0.58 | -0.02 | 0.00 | 1.56E-16 | 68.10 |
| 194 | rs1549293 | T | C | 0.36 | -0.02 | 0.00 | 9.22E-11 | 41.98 |
| 195 | rs12928099 | A | C | 0.30 | -0.03 | 0.00 | 1.33E-33 | 145.98 |
| 196 | rs12446515 | T | C | 0.32 | -0.04 | 0.00 | 2.88E-46 | 204.01 |
| 197 | rs2925979 | C | T | 0.70 | -0.03 | 0.00 | 1.14E-35 | 155.44 |
| 198 | rs34967613 | CTT | C | 0.47 | 0.02 | 0.00 | 1.09E-10 | 41.66 |
| 199 | rs1684608 | A | C | 0.19 | 0.02 | 0.00 | 5.90E-10 | 38.35 |
| 200 | rs4843754 | G | A | 0.50 | 0.01 | 0.00 | 1.02E-09 | 37.28 |
| 201 | rs74456742 | A | G | 0.04 | -0.05 | 0.01 | 1.52E-13 | 54.55 |
| 202 | rs115271198 | T | C | 0.07 | -0.03 | 0.00 | 1.54E-09 | 36.48 |
| 203 | rs72836561 | T | C | 0.03 | 0.14 | 0.01 | 2.37E-101 | 457.14 |
| 204 | rs56030759 | C | T | 0.06 | 0.04 | 0.00 | 5.76E-17 | 70.06 |
| 205 | rs60856912 | T | G | 0.16 | 0.03 | 0.00 | 7.96E-16 | 64.89 |
| 206 | rs591939 | G | A | 0.25 | 0.02 | 0.00 | 1.98E-14 | 58.56 |
| 207 | rs4789182 | A | G | 0.73 | 0.02 | 0.00 | 6.15E-09 | 33.79 |
| 208 | rs1801689 | C | A | 0.03 | -0.06 | 0.01 | 7.60E-21 | 87.72 |
| 209 | rs4969179 | G | T | 0.60 | -0.02 | 0.00 | 7.23E-15 | 60.54 |
| 210 | rs11078597 | C | T | 0.19 | 0.02 | 0.00 | 8.39E-12 | 46.67 |
| 211 | rs1292065 | G | C | 0.71 | -0.02 | 0.00 | 2.08E-10 | 40.39 |
| 212 | rs6506033 | T | C | 0.07 | -0.03 | 0.00 | 6.01E-11 | 42.82 |
| 213 | rs867939 | A | G | 0.58 | -0.02 | 0.00 | 4.97E-11 | 43.19 |
| 214 | rs11664106 | T | A | 0.37 | -0.01 | 0.00 | 1.70E-09 | 36.29 |
| 215 | rs2510344 | C | T | 0.49 | -0.02 | 0.00 | 2.06E-13 | 53.95 |
| 216 | rs68033110 | A | G | 0.25 | 0.02 | 0.00 | 6.63E-11 | 42.63 |
| 217 | rs58542926 | T | C | 0.08 | -0.11 | 0.00 | 1.73E-127 | 577.41 |
| 218 | rs62117489 | A | C | 0.05 | -0.05 | 0.01 | 3.88E-19 | 79.94 |
| 219 | rs5112 | G | C | 0.53 | 0.07 | 0.00 | 3.29E-166 | 755.81 |
| 220 | rs62112763 | G | C | 0.44 | 0.02 | 0.00 | 1.55E-18 | 77.20 |
| 221 | rs1688043 | T | C | 0.93 | 0.03 | 0.00 | 8.38E-10 | 37.67 |
| 222 | rs34690548 | CAAA | C | 0.11 | 0.02 | 0.00 | 8.18E-09 | 33.23 |
| 223 | rs188247550 | T | C | 0.01 | -0.14 | 0.01 | 4.02E-38 | 166.68 |
| 224 | rs739320 | C | T | 0.60 | -0.02 | 0.00 | 1.75E-18 | 76.97 |
| 225 | rs12151142 | C | T | 0.44 | 0.02 | 0.00 | 3.00E-16 | 66.81 |
| 226 | rs62118471 | C | T | 0.03 | 0.05 | 0.01 | 1.41E-10 | 41.16 |
| 227 | rs116843064 | A | G | 0.02 | -0.23 | 0.01 | 2.67E-163 | 742.42 |
| 228 | rs10405944 | C | T | 0.48 | -0.01 | 0.00 | 3.32E-08 | 30.51 |
| 229 | rs483082 | T | G | 0.24 | 0.09 | 0.00 | 1.00E-200 | 1074.80 |
| 230 | rs62102718 | T | A | 0.29 | 0.02 | 0.00 | 5.50E-16 | 65.61 |
| 231 | rs6073958 | C | T | 0.20 | 0.06 | 0.00 | 1.00E-81 | 366.83 |
| 232 | rs8126001 | T | C | 0.49 | -0.02 | 0.00 | 8.43E-15 | 60.23 |
| 233 | rs149142833 | T | C | 0.16 | 0.02 | 0.00 | 1.99E-09 | 35.99 |
| 234 | rs6093446 | A | G | 0.29 | 0.01 | 0.00 | 2.91E-08 | 30.77 |
| 235 | rs6066138 | A | G | 0.28 | -0.02 | 0.00 | 1.69E-14 | 58.87 |
| 236 | rs7274718 | A | G | 0.60 | 0.02 | 0.00 | 2.43E-11 | 44.59 |
| 237 | rs12480662 | T | C | 0.25 | -0.02 | 0.00 | 8.49E-09 | 33.16 |
| 238 | rs2071887 | A | T | 0.34 | 0.02 | 0.00 | 5.70E-12 | 47.44 |
| 239 | rs11705483 | A | C | 0.11 | 0.02 | 0.00 | 8.69E-11 | 42.10 |
| 240 | rs2267373 | T | C | 0.58 | 0.02 | 0.00 | 1.29E-21 | 91.22 |
| 241 | rs5755799 | G | C | 0.45 | 0.01 | 0.00 | 1.92E-08 | 31.58 |
| 242 | rs5965373 | G | T | 0.86 | -0.02 | 0.00 | 1.22E-10 | 41.44 |

EAF: Effect allele frequency; TG: Triglycerides

Table 6: Characteristics of SNPs used as instrumental variables for LDL

|  | SNP | effect_allele.exposure | other_allele.exposure | eaf.exposure | beta.exposure | se.exposure | pval.exposure | F-Statistics |
| --- | --- | --- | --- | --- | --- | --- | --- | --- |
| 1 | rs7528419 | G | A | NA | -0.14 | 0.02 | 7.73E-16 | 49 |
| 2 | rs11591147 | T | G | NA | -0.33 | 0.06 | 2.03E-08 | 30.25 |
| 3 | rs548145 | C | T | NA | 0.11 | 0.02 | 8.43E-09 | 30.25 |
| 4 | rs55730499 | T | C | NA | 0.16 | 0.03 | 8.94E-10 | 28.44 |
| 5 | rs7412 | T | C | NA | -0.48 | 0.03 | 1.15E-77 | 256 |
| 6 | rs112552009 | G | T | NA | -0.16 | 0.02 | 2.24E-13 | 64 |

EAF: Effect allele frequency; LDL: Low-density lipoprotein

Table 7: Characteristics of SNPs used as instrumental variables for HDL

|  | SNP | effect_allele.exposure | other_allele.exposure | eaf.exposure | beta.exposure | se.exposure | pval.exposure | F-Statistics |
| --- | --- | --- | --- | --- | --- | --- | --- | --- |
| 1 | rs117199990 | T | C | NA | 0.19 | 0.02 | 6.22E-14 | 90.25 |
| 2 | rs964184 | C | G | NA | 0.14 | 0.02 | 2.00E-10 | 49 |
| 3 | rs1532085 | G | A | NA | -0.1 | 0.02 | 1.64E-11 | 25 |
| 4 | rs183130 | T | C | NA | 0.24 | 0.02 | 3.22E-54 | 144 |
| 5 | rs75911530 | A | G | NA | -0.25 | 0.04 | 1.65E-08 | 39.06 |

EAF: Effect allele frequency; HDL: High-density lipoprotein

Table 8: Characteristics of SNPs used as instrumental variables for TC

|  | SNP | effect_allele.exposure | other_allele.exposure | eaf.exposure | beta.exposure | se.exposure | pval.exposure | F-Statistics |
| --- | --- | --- | --- | --- | --- | --- | --- | --- |
| 1 | rs12740374 | T | G | NA | -0.11 | 0.02 | 5.28E-10 | 30.25 |
| 2 | rs10468017 | T | C | NA | 0.09 | 0.02 | 2.19E-08 | 20.25 |
| 3 | rs112552009 | G | T | NA | -0.14 | 0.02 | 2.33E-10 | 49 |
| 4 | rs7412 | T | C | NA | -0.3 | 0.03 | 8.50E-32 | 100 |

EAF: Effect allele frequency; TC: Total cholesterol

Table 9: Characteristics of SNPs used as instrumental variables for WHR

|  | SNP | effect_allele.exposure | other_allele.exposure | eaf.exposure | beta.exposure | se.exposure | pval.exposure | F-Statistics |
| --- | --- | --- | --- | --- | --- | --- | --- | --- |
| 1 | rs714515 | A | G | 0.54 | -0.03 | 0.00 | 4.40E-15 | 63.06 |
| 2 | rs2820443 | C | T | 0.30 | -0.04 | 0.00 | 5.30E-21 | 89.48 |
| 3 | rs12143789 | C | G | 0.16 | 0.02 | 0.00 | 7.60E-09 | 32.65 |
| 4 | rs2645294 | T | C | 0.53 | 0.03 | 0.00 | 1.70E-19 | 78.45 |
| 5 | rs905938 | C | T | 0.33 | -0.03 | 0.00 | 7.30E-10 | 39.06 |
| 6 | rs1385167 | G | A | 0.14 | 0.03 | 0.00 | 1.80E-09 | 35.03 |
| 7 | rs1569135 | G | A | 0.47 | -0.02 | 0.00 | 5.60E-10 | 38.15 |
| 8 | rs1128249 | T | G | 0.44 | -0.03 | 0.00 | 2.00E-15 | 64.00 |
| 9 | rs10804591 | A | C | 0.85 | 0.02 | 0.00 | 6.60E-09 | 32.65 |
| 10 | rs17819328 | G | T | 0.45 | 0.02 | 0.00 | 2.40E-09 | 36.00 |
| 11 | rs2276824 | G | C | 0.52 | -0.02 | 0.00 | 3.20E-11 | 44.44 |
| 12 | rs6772129 | G | A | 0.24 | -0.04 | 0.00 | 3.40E-21 | 89.48 |
| 13 | rs17451107 | C | T | 0.38 | -0.03 | 0.00 | 1.10E-12 | 52.16 |
| 14 | rs9991328 | T | C | 0.48 | 0.02 | 0.00 | 4.50E-08 | 28.03 |
| 15 | rs303084 | A | G | 0.78 | 0.02 | 0.00 | 3.90E-08 | 29.99 |
| 16 | rs459193 | G | A | 0.78 | -0.03 | 0.00 | 1.60E-11 | 46.81 |
| 17 | rs7705502 | A | G | 0.29 | 0.03 | 0.00 | 4.70E-14 | 56.25 |
| 18 | rs1936805 | T | C | 0.55 | 0.04 | 0.00 | 3.60E-35 | 152.60 |
| 19 | rs7759742 | A | T | 0.53 | 0.02 | 0.00 | 4.40E-11 | 45.76 |
| 20 | rs998584 | A | C | 0.48 | 0.04 | 0.00 | 1.10E-29 | 128.05 |
| 21 | rs1294410 | C | T | 0.63 | 0.03 | 0.00 | 2.00E-18 | 78.45 |
| 22 | rs10245353 | A | C | 0.18 | 0.04 | 0.00 | 8.40E-16 | 66.25 |
| 23 | rs12679556 | G | T | 0.21 | 0.03 | 0.00 | 2.10E-11 | 45.56 |
| 24 | rs10991437 | A | C | 0.10 | 0.03 | 0.01 | 1.00E-08 | 32.96 |
| 25 | rs11231693 | A | G | 0.04 | 0.04 | 0.01 | 4.50E-08 | 29.88 |
| 26 | rs2071449 | A | C | 0.33 | 0.03 | 0.00 | 3.00E-14 | 60.49 |
| 27 | rs4765219 | A | C | 0.38 | -0.03 | 0.00 | 1.60E-15 | 60.49 |
| 28 | rs10842707 | T | C | 0.17 | 0.03 | 0.00 | 4.40E-16 | 64.00 |
| 29 | rs8030605 | A | G | 0.16 | 0.03 | 0.01 | 8.80E-09 | 32.04 |
| 30 | rs8042543 | T | C | 0.15 | -0.03 | 0.00 | 1.20E-09 | 36.56 |
| 31 | rs1440372 | C | T | 0.74 | 0.02 | 0.00 | 1.10E-10 | 39.89 |
| 32 | rs4646404 | A | G | 0.38 | -0.03 | 0.00 | 1.40E-11 | 47.93 |
| 33 | rs12608504 | G | A | 0.66 | -0.02 | 0.00 | 8.80E-10 | 37.35 |
| 34 | rs4081724 | A | G | 0.15 | -0.04 | 0.01 | 7.40E-12 | 47.10 |
| 35 | rs6090583 | G | A | 0.57 | -0.02 | 0.00 | 6.20E-11 | 41.87 |
| 36 | rs979012 | C | T | 0.64 | -0.03 | 0.00 | 3.30E-14 | 56.25 |
| 37 | rs878639 | G | A | 0.36 | -0.02 | 0.00 | 5.10E-09 | 36.00 |
| 38 | rs2294239 | G | A | 0.45 | -0.03 | 0.00 | 7.20E-13 | 51.02 |

EAF: Effect allele frequency; WHR: Waist-hip-ratio

Table 10: Characteristics of SNPs used as instrumental variables for BMI

|  | SNP | effect_allele.exposure | other_allele.exposure | eaf.exposure | beta.exposure | se.exposure | pval.exposure | F-Statistics |
| --- | --- | --- | --- | --- | --- | --- | --- | --- |
| 1 | rs12044597 | G | A | 0.50 | 0.01 | 0.00 | 1.70E-18 | 79.88 |
| 2 | rs2284746 | G | C | 0.52 | -0.01 | 0.00 | 1.40E-09 | 37.43 |
| 3 | rs6692586 | G | A | 0.83 | -0.02 | 0.00 | 1.10E-16 | 69.69 |
| 4 | rs705217 | G | T | 0.37 | -0.01 | 0.00 | 9.30E-09 | 32.11 |
| 5 | rs4660443 | T | C | 0.22 | 0.02 | 0.00 | 6.80E-15 | 60.99 |
| 6 | rs17014375 | G | T | 0.13 | 0.02 | 0.00 | 1.10E-11 | 47.33 |
| 7 | rs7535528 | A | G | 0.37 | -0.02 | 0.00 | 1.40E-16 | 71.31 |
| 8 | rs2235564 | T | C | 0.35 | 0.01 | 0.00 | 3.70E-13 | 52.97 |
| 9 | rs10914462 | G | A | 0.43 | -0.01 | 0.00 | 1.50E-10 | 43.40 |
| 10 | rs12033257 | G | A | 0.38 | -0.01 | 0.00 | 2.40E-15 | 65.79 |
| 11 | rs6587552 | G | A | 0.76 | -0.02 | 0.00 | 1.60E-17 | 74.82 |
| 12 | rs6673081 | C | T | 0.55 | -0.01 | 0.00 | 1.80E-08 | 30.86 |
| 13 | rs543874 | G | A | 0.20 | 0.05 | 0.00 | 1.20E-122 | 564.06 |
| 14 | rs11118308 | G | A | 0.47 | -0.01 | 0.00 | 4.80E-10 | 39.85 |
| 15 | rs17425707 | C | T | 0.10 | 0.02 | 0.00 | 4.40E-09 | 35.57 |
| 16 | rs6593688 | G | A | 0.37 | 0.01 | 0.00 | 8.60E-15 | 57.93 |
| 17 | rs946824 | C | T | 0.86 | -0.02 | 0.00 | 1.10E-15 | 62.78 |
| 18 | rs1891216 | G | T | 0.38 | 0.01 | 0.00 | 2.40E-09 | 35.34 |
| 19 | rs2791653 | G | A | 0.76 | -0.01 | 0.00 | 1.30E-13 | 55.07 |
| 20 | rs657452 | G | A | 0.62 | -0.02 | 0.00 | 7.20E-29 | 122.30 |
| 21 | rs2481665 | C | T | 0.44 | -0.02 | 0.00 | 7.20E-23 | 101.25 |
| 22 | rs7519259 | A | G | 0.54 | 0.01 | 0.00 | 3.80E-13 | 54.07 |
| 23 | rs7551507 | T | C | 0.56 | -0.02 | 0.00 | 9.30E-30 | 132.25 |
| 24 | rs12049202 | T | C | 0.20 | 0.02 | 0.00 | 1.00E-28 | 119.01 |
| 25 | rs818524 | C | T | 0.69 | 0.01 | 0.00 | 3.40E-08 | 31.12 |
| 26 | rs2007231 | T | C | 0.64 | -0.01 | 0.00 | 5.20E-09 | 33.38 |
| 27 | rs10733051 | G | A | 0.48 | -0.01 | 0.00 | 2.90E-09 | 36.75 |
| 28 | rs12564992 | G | A | 0.11 | 0.02 | 0.00 | 5.30E-14 | 56.83 |
| 29 | rs10915840 | A | G | 0.28 | -0.01 | 0.00 | 1.30E-09 | 38.57 |
| 30 | rs4653017 | T | C | 0.68 | 0.01 | 0.00 | 4.50E-11 | 45.94 |
| 31 | rs977747 | G | T | 0.59 | -0.02 | 0.00 | 1.30E-24 | 98.83 |
| 32 | rs1993709 | G | A | 0.82 | 0.03 | 0.00 | 1.90E-57 | 248.44 |
| 33 | rs11165643 | T | C | 0.58 | 0.02 | 0.00 | 1.40E-35 | 146.84 |
| 34 | rs10747488 | A | C | 0.76 | -0.01 | 0.00 | 1.20E-09 | 37.82 |
| 35 | rs11185111 | A | G | 0.30 | -0.01 | 0.00 | 7.70E-12 | 46.10 |
| 36 | rs7550711 | T | C | 0.03 | 0.06 | 0.01 | 3.20E-38 | 168.48 |
| 37 | rs4414033 | A | G | 0.63 | 0.01 | 0.00 | 1.40E-12 | 51.36 |
| 38 | rs10920678 | G | A | 0.57 | -0.02 | 0.00 | 1.50E-21 | 93.85 |
| 39 | rs12041258 | C | T | 0.23 | -0.01 | 0.00 | 9.50E-13 | 53.29 |
| 40 | rs2820311 | G | A | 0.34 | 0.02 | 0.00 | 4.10E-38 | 170.45 |
| 41 | rs16849710 | G | A | 0.52 | -0.01 | 0.00 | 6.00E-11 | 41.53 |
| 42 | rs2693826 | A | G | 0.44 | -0.01 | 0.00 | 2.00E-15 | 64.94 |
| 43 | rs4358081 | C | A | 0.46 | 0.01 | 0.00 | 1.50E-08 | 32.56 |
| 44 | rs6545714 | A | G | 0.61 | -0.02 | 0.00 | 9.10E-31 | 126.23 |
| 45 | rs1371108 | A | C | 0.32 | 0.01 | 0.00 | 9.00E-11 | 43.71 |
| 46 | rs4851029 | G | T | 0.52 | 0.01 | 0.00 | 1.70E-12 | 50.66 |
| 47 | rs10192119 | G | T | 0.17 | 0.02 | 0.00 | 3.00E-14 | 56.93 |
| 48 | rs1521527 | C | G | 0.53 | -0.01 | 0.00 | 3.10E-12 | 50.66 |
| 49 | rs17499593 | G | C | 0.19 | 0.01 | 0.00 | 1.10E-08 | 32.28 |
| 50 | rs1064213 | A | G | 0.49 | 0.01 | 0.00 | 2.40E-12 | 49.83 |
| 51 | rs4639527 | G | A | 0.30 | 0.02 | 0.00 | 3.30E-20 | 81.95 |
| 52 | rs13021737 | G | A | 0.83 | 0.06 | 0.00 | 7.50E-157 | 747.11 |
| 53 | rs10169594 | C | T | 0.36 | 0.01 | 0.00 | 2.00E-11 | 45.19 |
| 54 | rs4556997 | A | C | 0.13 | 0.02 | 0.00 | 6.90E-17 | 67.38 |
| 55 | rs429343 | G | A | 0.58 | -0.02 | 0.00 | 6.80E-18 | 77.85 |
| 56 | rs3764835 | A | G | 0.15 | -0.01 | 0.00 | 3.10E-09 | 34.52 |
| 57 | rs3754963 | T | A | 0.26 | -0.01 | 0.00 | 3.30E-10 | 37.82 |
| 58 | rs12328930 | C | T | 0.42 | 0.01 | 0.00 | 1.80E-08 | 33.23 |
| 59 | rs1528435 | T | C | 0.63 | 0.02 | 0.00 | 9.10E-23 | 93.07 |
| 60 | rs3731695 | C | T | 0.56 | 0.01 | 0.00 | 7.90E-13 | 52.56 |
| 61 | rs7599312 | A | G | 0.27 | -0.02 | 0.00 | 6.90E-24 | 95.83 |
| 62 | rs1260326 | C | T | 0.60 | 0.01 | 0.00 | 3.90E-10 | 38.15 |
| 63 | rs17399237 | C | T | 0.55 | -0.01 | 0.00 | 6.70E-14 | 57.58 |
| 64 | rs930295 | C | A | 0.84 | -0.02 | 0.00 | 1.00E-19 | 84.16 |
| 65 | rs4671328 | G | T | 0.55 | -0.02 | 0.00 | 2.20E-36 | 165.96 |
| 66 | rs7557796 | C | T | 0.65 | -0.02 | 0.00 | 2.30E-19 | 79.01 |
| 67 | rs10197031 | C | T | 0.28 | 0.02 | 0.00 | 1.90E-18 | 76.33 |
| 68 | rs1445652 | A | G | 0.19 | 0.01 | 0.00 | 4.30E-08 | 31.26 |
| 69 | rs4482463 | A | C | 0.92 | -0.03 | 0.00 | 2.80E-23 | 100.61 |
| 70 | rs3732084 | C | T | 0.61 | 0.01 | 0.00 | 1.10E-09 | 35.34 |
| 71 | rs2162524 | C | T | 0.33 | 0.02 | 0.00 | 4.10E-17 | 74.15 |
| 72 | rs10182181 | G | A | 0.48 | 0.03 | 0.00 | 6.70E-90 | 412.60 |
| 73 | rs902695 | A | G | 0.48 | -0.01 | 0.00 | 2.20E-09 | 36.71 |
| 74 | rs4954638 | C | A | 0.25 | -0.01 | 0.00 | 2.90E-09 | 34.81 |
| 75 | rs4589691 | G | C | 0.16 | 0.01 | 0.00 | 4.70E-09 | 34.52 |
| 76 | rs11889536 | G | A | 0.15 | -0.02 | 0.00 | 6.40E-15 | 62.02 |
| 77 | rs934224 | T | C | 0.74 | 0.01 | 0.00 | 4.70E-08 | 28.62 |
| 78 | rs4952843 | G | A | 0.38 | -0.01 | 0.00 | 6.80E-14 | 52.97 |
| 79 | rs3806572 | A | G | 0.28 | -0.01 | 0.00 | 1.60E-14 | 58.24 |
| 80 | rs2861683 | C | A | 0.41 | -0.01 | 0.00 | 1.30E-16 | 71.75 |
| 81 | rs17551974 | A | C | 0.18 | -0.01 | 0.00 | 1.90E-10 | 41.08 |
| 82 | rs17203016 | G | A | 0.20 | 0.02 | 0.00 | 2.10E-13 | 56.25 |
| 83 | rs199783965 | T | C | 0.35 | 0.02 | 0.00 | 6.50E-18 | 75.11 |
| 84 | rs2317299 | C | T | 0.56 | -0.01 | 0.00 | 1.30E-09 | 38.88 |
| 85 | rs17033117 | T | C | 0.19 | 0.01 | 0.00 | 8.90E-10 | 38.78 |
| 86 | rs11713193 | A | G | 0.51 | 0.02 | 0.00 | 2.40E-44 | 197.65 |
| 87 | rs1452075 | T | C | 0.73 | 0.01 | 0.00 | 1.30E-14 | 61.36 |
| 88 | rs1006896 | C | A | 0.11 | -0.02 | 0.00 | 5.50E-18 | 75.11 |
| 89 | rs645040 | T | G | 0.78 | 0.02 | 0.00 | 2.50E-18 | 73.10 |
| 90 | rs6443750 | C | T | 0.81 | 0.01 | 0.00 | 3.20E-12 | 49.67 |
| 91 | rs9816226 | T | A | 0.82 | 0.03 | 0.00 | 1.60E-52 | 236.57 |
| 92 | rs1552893 | G | A | 0.28 | -0.01 | 0.00 | 8.10E-11 | 43.98 |
| 93 | rs6764533 | A | G | 0.36 | 0.01 | 0.00 | 1.40E-10 | 41.53 |
| 94 | rs17535749 | A | G | 0.10 | 0.02 | 0.00 | 2.50E-08 | 30.86 |
| 95 | rs754635 | G | C | 0.89 | 0.02 | 0.00 | 2.20E-13 | 53.78 |
| 96 | rs7626079 | T | C | 0.34 | 0.01 | 0.00 | 1.60E-09 | 37.35 |
| 97 | rs1656377 | C | T | 0.59 | 0.01 | 0.00 | 1.60E-08 | 33.91 |
| 98 | rs13069244 | A | G | 0.08 | 0.02 | 0.00 | 3.00E-09 | 34.15 |
| 99 | rs2600226 | T | C | 0.67 | -0.01 | 0.00 | 3.70E-10 | 37.27 |
| 100 | rs9845966 | G | T | 0.55 | -0.01 | 0.00 | 2.50E-10 | 38.15 |
| 101 | rs4858193 | C | T | 0.28 | -0.01 | 0.00 | 1.60E-11 | 46.10 |
| 102 | rs33500 | T | C | 0.81 | -0.02 | 0.00 | 4.30E-14 | 57.62 |
| 103 | rs538579 | C | G | 0.32 | 0.01 | 0.00 | 1.30E-13 | 51.99 |
| 104 | rs3772882 | A | C | 0.37 | 0.01 | 0.00 | 6.60E-13 | 49.78 |
| 105 | rs7640424 | T | C | 0.30 | -0.01 | 0.00 | 2.30E-14 | 57.09 |
| 106 | rs2868975 | A | G | 0.18 | -0.01 | 0.00 | 2.20E-10 | 38.66 |
| 107 | rs8192675 | C | T | 0.29 | 0.02 | 0.00 | 1.40E-17 | 71.31 |
| 108 | rs10510419 | T | G | 0.14 | -0.02 | 0.00 | 2.20E-14 | 59.22 |
| 109 | rs2365389 | T | C | 0.41 | -0.02 | 0.00 | 1.30E-25 | 104.76 |
| 110 | rs6785245 | C | T | 0.40 | 0.01 | 0.00 | 4.00E-14 | 60.29 |
| 111 | rs12629015 | G | A | 0.19 | -0.01 | 0.00 | 2.10E-09 | 34.45 |
| 112 | rs1320903 | A | G | 0.32 | 0.02 | 0.00 | 9.20E-32 | 144.00 |
| 113 | rs16851483 | T | G | 0.07 | 0.04 | 0.00 | 3.20E-26 | 111.15 |
| 114 | rs7615297 | G | C | 0.15 | -0.01 | 0.00 | 5.70E-10 | 38.54 |
| 115 | rs6804842 | G | A | 0.57 | 0.02 | 0.00 | 3.60E-21 | 84.21 |
| 116 | rs7637852 | G | A | 0.70 | -0.01 | 0.00 | 1.70E-13 | 53.52 |
| 117 | rs2325036 | C | A | 0.38 | -0.02 | 0.00 | 3.60E-27 | 113.36 |
| 118 | rs1454687 | G | C | 0.52 | -0.02 | 0.00 | 5.20E-32 | 141.19 |
| 119 | rs1436344 | C | G | 0.59 | 0.01 | 0.00 | 4.10E-16 | 68.79 |
| 120 | rs2124499 | C | G | 0.37 | -0.01 | 0.00 | 3.40E-13 | 52.35 |
| 121 | rs355777 | C | G | 0.41 | 0.02 | 0.00 | 1.40E-18 | 81.00 |
| 122 | rs1476322 | A | G | 0.57 | 0.01 | 0.00 | 5.00E-09 | 35.30 |
| 123 | rs6772756 | G | A | 0.34 | -0.01 | 0.00 | 4.00E-08 | 29.96 |
| 124 | rs865809 | G | A | 0.77 | -0.01 | 0.00 | 5.40E-10 | 40.32 |
| 125 | rs10938397 | G | A | 0.43 | 0.03 | 0.00 | 3.40E-86 | 410.06 |
| 126 | rs326896 | T | C | 0.39 | -0.01 | 0.00 | 2.80E-13 | 50.57 |
| 127 | rs4864201 | C | T | 0.65 | -0.01 | 0.00 | 1.50E-16 | 68.79 |
| 128 | rs331966 | C | A | 0.38 | 0.01 | 0.00 | 3.20E-10 | 38.72 |
| 129 | rs11736228 | T | A | 0.26 | -0.01 | 0.00 | 4.10E-12 | 48.30 |
| 130 | rs1492767 | T | C | 0.50 | 0.01 | 0.00 | 1.00E-08 | 34.52 |
| 131 | rs7685048 | T | C | 0.47 | -0.01 | 0.00 | 4.10E-09 | 35.30 |
| 132 | rs13107325 | T | C | 0.07 | 0.05 | 0.00 | 1.10E-47 | 215.72 |
| 133 | rs2051559 | C | T | 0.13 | 0.02 | 0.00 | 5.00E-12 | 45.82 |
| 134 | rs13132853 | G | A | 0.35 | -0.01 | 0.00 | 4.70E-15 | 62.23 |
| 135 | rs10009336 | T | C | 0.16 | -0.01 | 0.00 | 2.20E-10 | 40.50 |
| 136 | rs6815910 | A | T | 0.54 | -0.01 | 0.00 | 1.40E-13 | 56.69 |
| 137 | rs17001561 | A | G | 0.16 | 0.02 | 0.00 | 3.80E-11 | 43.10 |
| 138 | rs13147390 | C | T | 0.36 | 0.01 | 0.00 | 1.00E-08 | 32.74 |
| 139 | rs4148155 | G | A | 0.11 | -0.02 | 0.00 | 5.00E-13 | 52.28 |
| 140 | rs7694732 | G | A | 0.44 | -0.01 | 0.00 | 8.70E-09 | 33.91 |
| 141 | rs1296328 | C | A | 0.57 | -0.02 | 0.00 | 4.90E-24 | 98.89 |
| 142 | rs13110266 | A | G | 0.41 | -0.01 | 0.00 | 1.90E-12 | 47.37 |
| 143 | rs1522569 | G | T | 0.18 | -0.02 | 0.00 | 2.90E-13 | 55.57 |
| 144 | rs2643452 | A | T | 0.54 | 0.01 | 0.00 | 4.70E-15 | 64.00 |
| 145 | rs6448587 | C | A | 0.19 | -0.02 | 0.00 | 2.30E-13 | 52.72 |
| 146 | rs11945861 | A | G | 0.24 | -0.01 | 0.00 | 5.00E-13 | 54.76 |
| 147 | rs1804528 | A | G | 0.35 | 0.01 | 0.00 | 3.00E-08 | 29.70 |
| 148 | rs6841761 | T | G | 0.53 | -0.01 | 0.00 | 6.40E-16 | 67.04 |
| 149 | rs1863652 | A | G | 0.34 | -0.01 | 0.00 | 1.40E-10 | 40.82 |
| 150 | rs7683836 | A | G | 0.54 | -0.01 | 0.00 | 6.30E-11 | 44.97 |
| 151 | rs7730004 | T | C | 0.67 | 0.01 | 0.00 | 9.10E-16 | 67.60 |
| 152 | rs17424296 | A | G | 0.37 | -0.01 | 0.00 | 2.40E-09 | 36.00 |
| 153 | rs2931434 | T | C | 0.32 | -0.01 | 0.00 | 1.40E-08 | 33.38 |
| 154 | rs13174863 | G | A | 0.15 | 0.02 | 0.00 | 2.90E-16 | 69.69 |
| 155 | rs3844598 | G | A | 0.52 | 0.01 | 0.00 | 3.80E-08 | 31.23 |
| 156 | rs2367112 | G | T | 0.49 | -0.01 | 0.00 | 2.30E-13 | 55.32 |
| 157 | rs16903285 | C | T | 0.14 | 0.03 | 0.00 | 7.60E-38 | 162.07 |
| 158 | rs11739877 | T | C | 0.61 | 0.01 | 0.00 | 6.60E-11 | 42.25 |
| 159 | rs6595205 | G | C | 0.53 | -0.01 | 0.00 | 2.00E-12 | 50.77 |
| 160 | rs7715256 | T | G | 0.58 | -0.02 | 0.00 | 2.20E-24 | 107.64 |
| 161 | rs2307111 | C | T | 0.40 | -0.03 | 0.00 | 1.60E-58 | 274.32 |
| 162 | rs6235 | G | C | 0.27 | 0.02 | 0.00 | 1.50E-19 | 84.83 |
| 163 | rs11951673 | T | C | 0.39 | -0.01 | 0.00 | 1.10E-13 | 52.35 |
| 164 | rs10478110 | C | A | 0.43 | 0.01 | 0.00 | 9.60E-09 | 34.60 |
| 165 | rs13184896 | T | G | 0.43 | -0.01 | 0.00 | 3.30E-16 | 69.10 |
| 166 | rs7724675 | A | G | 0.22 | -0.01 | 0.00 | 9.50E-09 | 32.11 |
| 167 | rs7703576 | C | T | 0.29 | 0.01 | 0.00 | 4.80E-08 | 29.39 |
| 168 | rs16871902 | A | G | 0.49 | 0.01 | 0.00 | 4.60E-13 | 54.07 |
| 169 | rs7704281 | A | G | 0.05 | 0.03 | 0.00 | 6.50E-11 | 43.69 |
| 170 | rs1503526 | C | T | 0.48 | 0.01 | 0.00 | 5.50E-17 | 67.82 |
| 171 | rs2009416 | T | C | 0.36 | -0.01 | 0.00 | 1.10E-11 | 45.19 |
| 172 | rs40067 | A | G | 0.17 | -0.03 | 0.00 | 7.10E-30 | 133.75 |
| 173 | rs294704 | T | G | 0.72 | -0.01 | 0.00 | 4.00E-09 | 35.37 |
| 174 | rs4518345 | A | G | 0.28 | -0.01 | 0.00 | 1.00E-09 | 37.92 |
| 175 | rs876605 | G | A | 0.74 | -0.01 | 0.00 | 3.40E-08 | 29.16 |
| 176 | rs10942267 | G | A | 0.31 | -0.02 | 0.00 | 3.90E-17 | 67.41 |
| 177 | rs11738695 | A | C | 0.59 | 0.01 | 0.00 | 2.00E-08 | 32.56 |
| 178 | rs17056301 | C | T | 0.26 | 0.01 | 0.00 | 2.40E-09 | 34.81 |
| 179 | rs189843 | C | G | 0.56 | -0.01 | 0.00 | 1.70E-08 | 33.23 |
| 180 | rs17663412 | A | C | 0.11 | 0.02 | 0.00 | 6.10E-09 | 33.81 |
| 181 | rs7730898 | A | G | 0.73 | 0.02 | 0.00 | 4.50E-20 | 87.11 |
| 182 | rs806600 | G | A | 0.48 | -0.01 | 0.00 | 3.30E-08 | 31.23 |
| 183 | rs6556301 | T | G | 0.36 | -0.01 | 0.00 | 4.10E-10 | 38.03 |
| 184 | rs2228213 | A | G | 0.35 | -0.01 | 0.00 | 4.60E-16 | 66.85 |
| 185 | rs9367368 | C | T | 0.30 | -0.01 | 0.00 | 1.00E-11 | 45.19 |
| 186 | rs9379827 | A | C | 0.24 | -0.01 | 0.00 | 6.90E-12 | 48.27 |
| 187 | rs9370261 | T | C | 0.05 | 0.02 | 0.00 | 3.40E-08 | 30.25 |
| 188 | rs947612 | A | G | 0.75 | -0.01 | 0.00 | 5.60E-09 | 33.64 |
| 189 | rs2246012 | C | T | 0.16 | 0.02 | 0.00 | 3.10E-13 | 51.58 |
| 190 | rs1885728 | A | G | 0.68 | 0.01 | 0.00 | 1.00E-08 | 32.31 |
| 191 | rs3828783 | A | G | 0.18 | -0.02 | 0.00 | 5.60E-15 | 61.73 |
| 192 | rs2744974 | T | C | 0.34 | 0.02 | 0.00 | 1.40E-45 | 191.36 |
| 193 | rs765875 | T | C | 0.48 | -0.01 | 0.00 | 3.00E-12 | 50.66 |
| 194 | rs3806114 | A | G | 0.68 | -0.01 | 0.00 | 3.40E-10 | 39.41 |
| 195 | rs987237 | G | A | 0.18 | 0.04 | 0.00 | 9.30E-84 | 379.32 |
| 196 | rs1327259 | G | A | 0.39 | -0.02 | 0.00 | 1.70E-18 | 74.15 |
| 197 | rs9688431 | C | T | 0.06 | -0.02 | 0.00 | 2.40E-11 | 43.56 |
| 198 | rs9294260 | A | G | 0.47 | 0.01 | 0.00 | 1.80E-19 | 84.41 |
| 199 | rs2357760 | A | G | 0.68 | 0.01 | 0.00 | 6.80E-17 | 72.75 |
| 200 | rs2875762 | C | G | 0.25 | 0.01 | 0.00 | 1.20E-11 | 48.30 |
| 201 | rs1268065 | A | G | 0.48 | -0.01 | 0.00 | 1.00E-09 | 36.00 |
| 202 | rs262130 | T | C | 0.20 | 0.01 | 0.00 | 1.80E-08 | 30.49 |
| 203 | rs4495304 | C | T | 0.07 | -0.02 | 0.00 | 5.00E-09 | 34.56 |
| 204 | rs2033529 | G | A | 0.29 | 0.02 | 0.00 | 1.90E-30 | 129.71 |
| 205 | rs3749897 | T | C | 0.42 | 0.01 | 0.00 | 8.40E-12 | 45.94 |
| 206 | rs1266874 | G | A | 0.36 | 0.01 | 0.00 | 9.80E-15 | 60.49 |
| 207 | rs7761673 | A | T | 0.21 | -0.01 | 0.00 | 1.90E-09 | 36.00 |
| 208 | rs200810 | C | T | 0.37 | -0.01 | 0.00 | 5.50E-16 | 64.00 |
| 209 | rs9375702 | T | C | 0.71 | -0.01 | 0.00 | 7.90E-10 | 36.63 |
| 210 | rs9362662 | G | A | 0.52 | -0.01 | 0.00 | 1.20E-10 | 43.40 |
| 211 | rs901630 | T | C | 0.40 | -0.01 | 0.00 | 1.90E-18 | 73.76 |
| 212 | rs17789218 | C | T | 0.24 | 0.01 | 0.00 | 7.40E-12 | 46.81 |
| 213 | rs156201 | C | G | 0.76 | 0.01 | 0.00 | 5.80E-10 | 37.82 |
| 214 | rs3800229 | T | G | 0.71 | 0.02 | 0.00 | 1.40E-22 | 94.52 |
| 215 | rs1538247 | C | T | 0.32 | 0.01 | 0.00 | 1.00E-08 | 32.31 |
| 216 | rs9478671 | G | A | 0.21 | 0.01 | 0.00 | 1.70E-08 | 32.65 |
| 217 | rs486359 | C | G | 0.49 | 0.01 | 0.00 | 1.60E-11 | 43.40 |
| 218 | rs13191362 | G | A | 0.12 | -0.02 | 0.00 | 5.90E-21 | 89.11 |
| 219 | rs17207196 | T | C | 0.41 | -0.02 | 0.00 | 2.10E-35 | 150.74 |
| 220 | rs3807645 | A | G | 0.22 | -0.02 | 0.00 | 2.40E-15 | 62.49 |
| 221 | rs11496125 | T | C | 0.42 | 0.02 | 0.00 | 3.00E-22 | 98.83 |
| 222 | rs10247983 | A | G | 0.92 | 0.02 | 0.00 | 1.70E-09 | 37.10 |
| 223 | rs3800637 | C | T | 0.34 | 0.01 | 0.00 | 5.10E-10 | 40.82 |
| 224 | rs2907948 | A | G | 0.24 | -0.01 | 0.00 | 1.30E-13 | 55.07 |
| 225 | rs1830074 | C | T | 0.29 | 0.01 | 0.00 | 1.40E-09 | 36.63 |
| 226 | rs4307239 | G | A | 0.46 | 0.01 | 0.00 | 3.90E-11 | 45.76 |
| 227 | rs12718572 | T | C | 0.40 | -0.01 | 0.00 | 3.00E-11 | 42.25 |
| 228 | rs38314 | A | G | 0.49 | -0.01 | 0.00 | 4.70E-12 | 49.83 |
| 229 | rs13240600 | G | A | 0.16 | -0.02 | 0.00 | 3.50E-17 | 72.25 |
| 230 | rs7788008 | A | G | 0.44 | -0.02 | 0.00 | 1.10E-19 | 85.29 |
| 231 | rs6461115 | G | A | 0.23 | -0.01 | 0.00 | 1.20E-13 | 57.44 |
| 232 | rs4722398 | T | C | 0.13 | 0.02 | 0.00 | 3.60E-10 | 39.94 |
| 233 | rs10248136 | T | C | 0.51 | -0.01 | 0.00 | 2.00E-08 | 32.56 |
| 234 | rs202240867 | T | C | 0.34 | -0.01 | 0.00 | 3.30E-11 | 45.19 |
| 235 | rs11505821 | T | A | 0.06 | 0.03 | 0.00 | 2.70E-19 | 78.96 |
| 236 | rs7811342 | C | T | 0.11 | -0.02 | 0.00 | 1.10E-11 | 46.15 |
| 237 | rs10243319 | C | T | 0.39 | -0.01 | 0.00 | 1.20E-09 | 35.34 |
| 238 | rs774246 | G | A | 0.14 | 0.02 | 0.00 | 5.40E-10 | 37.45 |
| 239 | rs215634 | G | A | 0.62 | -0.02 | 0.00 | 2.60E-17 | 71.31 |
| 240 | rs10269783 | A | G | 0.39 | 0.01 | 0.00 | 1.40E-15 | 61.21 |
| 241 | rs7780752 | C | T | 0.36 | 0.01 | 0.00 | 1.00E-14 | 59.63 |
| 242 | rs10953740 | G | A | 0.55 | -0.02 | 0.00 | 1.00E-18 | 81.00 |
| 243 | rs2283093 | T | C | 0.21 | 0.01 | 0.00 | 3.10E-09 | 36.57 |
| 244 | rs2543132 | C | G | 0.81 | 0.01 | 0.00 | 5.00E-11 | 44.04 |
| 245 | rs1421334 | C | A | 0.54 | -0.01 | 0.00 | 1.00E-12 | 48.23 |
| 246 | rs7826312 | C | T | 0.59 | 0.01 | 0.00 | 4.90E-10 | 37.43 |
| 247 | rs12546578 | A | T | 0.72 | 0.01 | 0.00 | 1.00E-13 | 53.29 |
| 248 | rs12680842 | G | A | 0.32 | -0.01 | 0.00 | 4.40E-14 | 54.60 |
| 249 | rs12675063 | T | A | 0.11 | 0.02 | 0.00 | 1.30E-09 | 36.00 |
| 250 | rs4072917 | A | G | 0.47 | 0.01 | 0.00 | 6.90E-11 | 40.82 |
| 251 | rs7844647 | C | T | 0.27 | -0.01 | 0.00 | 2.80E-11 | 46.69 |
| 252 | rs13250058 | T | G | 0.68 | 0.01 | 0.00 | 2.90E-10 | 38.72 |
| 253 | rs6985109 | A | G | 0.53 | -0.02 | 0.00 | 1.50E-26 | 108.40 |
| 254 | rs17119937 | C | T | 0.07 | 0.02 | 0.00 | 5.60E-09 | 34.68 |
| 255 | rs6471941 | A | G | 0.17 | 0.02 | 0.00 | 3.10E-13 | 55.18 |
| 256 | rs12334877 | A | G | 0.20 | -0.01 | 0.00 | 7.70E-11 | 42.84 |
| 257 | rs1431659 | G | A | 0.73 | -0.02 | 0.00 | 6.00E-24 | 106.42 |
| 258 | rs7819514 | A | G | 0.32 | -0.01 | 0.00 | 5.70E-09 | 35.34 |
| 259 | rs2694047 | G | A | 0.75 | 0.02 | 0.00 | 3.90E-21 | 88.36 |
| 260 | rs11781699 | C | T | 0.19 | 0.01 | 0.00 | 3.10E-10 | 39.51 |
| 261 | rs13263601 | C | A | 0.35 | 0.02 | 0.00 | 2.20E-17 | 73.20 |
| 262 | rs1982441 | T | G | 0.14 | 0.02 | 0.00 | 7.00E-12 | 45.30 |
| 263 | rs17405819 | C | T | 0.30 | -0.02 | 0.00 | 4.30E-33 | 142.67 |
| 264 | rs7037266 | A | C | 0.37 | -0.01 | 0.00 | 3.50E-10 | 38.72 |
| 265 | rs1948080 | G | T | 0.37 | -0.01 | 0.00 | 1.10E-14 | 57.93 |
| 266 | rs10962550 | C | G | 0.18 | 0.02 | 0.00 | 6.20E-16 | 68.44 |
| 267 | rs10971709 | T | C | 0.21 | 0.01 | 0.00 | 6.20E-10 | 39.51 |
| 268 | rs3829849 | T | C | 0.36 | 0.01 | 0.00 | 5.90E-09 | 33.23 |
| 269 | rs10858334 | G | C | 0.14 | 0.01 | 0.00 | 2.70E-08 | 30.25 |
| 270 | rs1412235 | C | G | 0.32 | 0.02 | 0.00 | 6.00E-45 | 209.40 |
| 271 | rs1472169 | T | C | 0.38 | -0.01 | 0.00 | 2.80E-15 | 59.63 |
| 272 | rs7024334 | G | T | 0.77 | -0.01 | 0.00 | 3.10E-12 | 47.61 |
| 273 | rs10984756 | G | C | 0.10 | 0.02 | 0.00 | 1.10E-09 | 36.00 |
| 274 | rs10968114 | C | A | 0.47 | -0.01 | 0.00 | 6.10E-11 | 44.18 |
| 275 | rs7025938 | G | C | 0.32 | 0.02 | 0.00 | 3.70E-19 | 76.33 |
| 276 | rs9408882 | A | G | 0.46 | -0.01 | 0.00 | 1.30E-08 | 33.79 |
| 277 | rs2174307 | C | G | 0.41 | 0.01 | 0.00 | 4.90E-12 | 50.66 |
| 278 | rs10867256 | T | C | 0.55 | -0.01 | 0.00 | 8.70E-12 | 48.18 |
| 279 | rs13287131 | C | T | 0.25 | 0.01 | 0.00 | 6.80E-10 | 37.82 |
| 280 | rs7869771 | C | A | 0.26 | -0.01 | 0.00 | 4.90E-13 | 54.29 |
| 281 | rs9650755 | G | A | 0.27 | 0.02 | 0.00 | 2.80E-15 | 59.29 |
| 282 | rs380857 | A | C | 0.89 | -0.02 | 0.00 | 3.60E-08 | 31.28 |
| 283 | rs1535660 | C | T | 0.86 | -0.01 | 0.00 | 5.20E-09 | 34.57 |
| 284 | rs4740619 | C | T | 0.45 | -0.02 | 0.00 | 2.30E-30 | 135.14 |
| 285 | rs10811871 | G | A | 0.38 | -0.01 | 0.00 | 1.60E-09 | 36.00 |
| 286 | rs1187352 | C | T | 0.65 | 0.01 | 0.00 | 6.00E-11 | 43.71 |
| 287 | rs1928295 | C | T | 0.45 | -0.01 | 0.00 | 5.40E-18 | 77.66 |
| 288 | rs7871866 | C | G | 0.15 | 0.02 | 0.00 | 2.30E-14 | 60.71 |
| 289 | rs12098284 | T | C | 0.12 | 0.02 | 0.00 | 1.80E-11 | 46.87 |
| 290 | rs11251352 | G | A | 0.60 | 0.01 | 0.00 | 7.00E-10 | 36.67 |
| 291 | rs12779328 | T | C | 0.28 | 0.01 | 0.00 | 4.50E-08 | 30.54 |
| 292 | rs10795422 | G | A | 0.69 | 0.01 | 0.00 | 9.30E-14 | 53.52 |
| 293 | rs1624134 | C | G | 0.41 | 0.01 | 0.00 | 1.10E-08 | 31.48 |
| 294 | rs2163188 | C | G | 0.47 | 0.01 | 0.00 | 2.00E-14 | 59.38 |
| 295 | rs7899106 | G | A | 0.05 | 0.03 | 0.00 | 1.00E-18 | 80.03 |
| 296 | rs7903146 | T | C | 0.29 | -0.02 | 0.00 | 1.30E-23 | 101.11 |
| 297 | rs7084454 | A | G | 0.34 | 0.02 | 0.00 | 4.00E-25 | 103.18 |
| 298 | rs12762034 | C | T | 0.08 | 0.02 | 0.00 | 7.30E-14 | 56.25 |
| 299 | rs3977755 | T | C | 0.28 | -0.01 | 0.00 | 5.90E-13 | 50.48 |
| 300 | rs3904244 | A | T | 0.14 | 0.02 | 0.00 | 4.30E-10 | 38.44 |
| 301 | rs1937683 | T | C | 0.67 | 0.01 | 0.00 | 3.20E-09 | 36.67 |
| 302 | rs17113297 | T | C | 0.21 | 0.02 | 0.00 | 2.10E-15 | 62.49 |
| 303 | rs17636031 | C | T | 0.27 | 0.02 | 0.00 | 1.20E-17 | 70.91 |
| 304 | rs999889 | A | G | 0.28 | -0.01 | 0.00 | 1.40E-08 | 32.31 |
| 305 | rs10887578 | C | G | 0.49 | 0.01 | 0.00 | 1.60E-13 | 56.69 |
| 306 | rs577525 | C | T | 0.57 | 0.02 | 0.00 | 9.70E-22 | 95.35 |
| 307 | rs1681740 | C | A | 0.39 | -0.01 | 0.00 | 1.10E-10 | 40.82 |
| 308 | rs845084 | A | G | 0.27 | 0.01 | 0.00 | 1.30E-12 | 49.00 |
| 309 | rs4880341 | T | C | 0.56 | -0.01 | 0.00 | 1.10E-11 | 48.18 |
| 310 | rs4757144 | A | G | 0.59 | 0.02 | 0.00 | 5.60E-22 | 88.15 |
| 311 | rs6265 | T | C | 0.20 | -0.04 | 0.00 | 1.00E-86 | 384.91 |
| 312 | rs7102454 | C | T | 0.34 | 0.02 | 0.00 | 2.40E-18 | 77.05 |
| 313 | rs592483 | T | C | 0.57 | -0.01 | 0.00 | 2.00E-18 | 74.77 |
| 314 | rs349088 | A | C | 0.50 | -0.01 | 0.00 | 1.80E-13 | 56.69 |
| 315 | rs7358465 | T | C | 0.68 | 0.01 | 0.00 | 3.00E-08 | 29.39 |
| 316 | rs2605603 | A | G | 0.49 | -0.01 | 0.00 | 2.50E-10 | 41.44 |
| 317 | rs10750215 | T | G | 0.39 | 0.01 | 0.00 | 1.30E-10 | 40.36 |
| 318 | rs4936175 | C | T | 0.44 | 0.01 | 0.00 | 1.40E-12 | 51.50 |
| 319 | rs12416812 | A | G | 0.51 | 0.01 | 0.00 | 6.10E-12 | 48.13 |
| 320 | rs10832778 | G | C | 0.62 | 0.01 | 0.00 | 1.30E-13 | 54.07 |
| 321 | rs2065418 | G | T | 0.36 | -0.02 | 0.00 | 3.60E-20 | 85.05 |
| 322 | rs10768994 | C | T | 0.43 | -0.01 | 0.00 | 6.40E-12 | 44.97 |
| 323 | rs6591407 | A | C | 0.19 | -0.01 | 0.00 | 1.90E-08 | 31.57 |
| 324 | rs4937870 | G | A | 0.32 | -0.01 | 0.00 | 8.80E-09 | 32.91 |
| 325 | rs7925214 | T | C | 0.51 | 0.01 | 0.00 | 4.40E-17 | 66.69 |
| 326 | rs491711 | C | A | 0.32 | -0.01 | 0.00 | 1.10E-09 | 36.63 |
| 327 | rs10742752 | C | T | 0.62 | 0.01 | 0.00 | 1.10E-13 | 53.20 |
| 328 | rs7117238 | A | G | 0.17 | -0.01 | 0.00 | 2.50E-09 | 35.46 |
| 329 | rs4310573 | T | C | 0.78 | 0.01 | 0.00 | 3.50E-08 | 30.51 |
| 330 | rs1048932 | A | C | 0.42 | -0.02 | 0.00 | 3.80E-22 | 88.58 |
| 331 | rs4929923 | C | T | 0.64 | 0.02 | 0.00 | 7.20E-27 | 113.36 |
| 332 | rs4237643 | G | T | 0.69 | -0.02 | 0.00 | 4.30E-33 | 137.75 |
| 333 | rs685870 | C | T | 0.70 | 0.01 | 0.00 | 2.40E-10 | 39.89 |
| 334 | rs11030618 | T | C | 0.57 | 0.01 | 0.00 | 2.40E-10 | 41.87 |
| 335 | rs7124681 | A | C | 0.41 | 0.03 | 0.00 | 3.20E-58 | 270.19 |
| 336 | rs1465900 | C | A | 0.22 | -0.01 | 0.00 | 4.80E-10 | 39.06 |
| 337 | rs1784460 | A | T | 0.40 | 0.01 | 0.00 | 9.00E-14 | 53.78 |
| 338 | rs12364470 | G | T | 0.16 | 0.02 | 0.00 | 1.10E-15 | 65.46 |
| 339 | rs2429150 | C | A | 0.42 | 0.01 | 0.00 | 2.70E-10 | 38.03 |
| 340 | rs7970953 | A | G | 0.29 | 0.01 | 0.00 | 9.80E-14 | 56.25 |
| 341 | rs10878946 | T | C | 0.71 | -0.01 | 0.00 | 3.60E-13 | 55.07 |
| 342 | rs11066188 | A | G | 0.42 | -0.01 | 0.00 | 8.10E-13 | 49.83 |
| 343 | rs11615578 | T | C | 0.25 | 0.01 | 0.00 | 8.10E-11 | 42.25 |
| 344 | rs11170468 | C | A | 0.23 | -0.01 | 0.00 | 1.90E-10 | 41.91 |
| 345 | rs7138803 | A | G | 0.38 | 0.03 | 0.00 | 2.30E-71 | 311.42 |
| 346 | rs705704 | A | G | 0.33 | -0.01 | 0.00 | 1.90E-13 | 52.97 |
| 347 | rs11105839 | A | T | 0.38 | -0.01 | 0.00 | 1.10E-10 | 41.11 |
| 348 | rs7488867 | T | C | 0.26 | -0.02 | 0.00 | 8.40E-24 | 104.04 |
| 349 | rs10492229 | T | C | 0.23 | 0.01 | 0.00 | 7.70E-14 | 55.86 |
| 350 | rs4148866 | T | C | 0.41 | 0.01 | 0.00 | 4.00E-08 | 29.64 |
| 351 | rs2306537 | G | A | 0.31 | 0.01 | 0.00 | 8.70E-13 | 49.00 |
| 352 | rs12422552 | C | G | 0.27 | -0.01 | 0.00 | 1.60E-11 | 44.89 |
| 353 | rs2608703 | A | C | 0.45 | 0.01 | 0.00 | 1.90E-16 | 69.77 |
| 354 | rs12299814 | A | C | 0.25 | -0.02 | 0.00 | 5.20E-15 | 61.62 |
| 355 | rs2943465 | C | T | 0.94 | 0.02 | 0.00 | 2.00E-10 | 40.44 |
| 356 | rs11173522 | A | C | 0.21 | 0.01 | 0.00 | 1.10E-09 | 37.15 |
| 357 | rs11115176 | C | T | 0.24 | -0.01 | 0.00 | 2.00E-10 | 40.56 |
| 358 | rs4842491 | T | C | 0.71 | 0.01 | 0.00 | 4.00E-08 | 29.64 |
| 359 | rs11609659 | C | T | 0.24 | -0.02 | 0.00 | 2.20E-14 | 59.29 |
| 360 | rs12369179 | T | C | 0.09 | -0.04 | 0.00 | 2.50E-31 | 134.11 |
| 361 | rs11611246 | T | G | 0.21 | 0.02 | 0.00 | 5.00E-32 | 144.00 |
| 362 | rs1843328 | A | C | 0.51 | -0.01 | 0.00 | 7.90E-09 | 33.91 |
| 363 | rs7318817 | T | C | 0.61 | -0.02 | 0.00 | 2.70E-18 | 74.15 |
| 364 | rs1927790 | C | T | 0.41 | 0.01 | 0.00 | 1.80E-19 | 85.56 |
| 365 | rs9522285 | A | G | 0.41 | 0.01 | 0.00 | 2.50E-13 | 55.81 |
| 366 | rs6561943 | T | C | 0.26 | 0.01 | 0.00 | 4.20E-10 | 39.23 |
| 367 | rs9300422 | G | A | 0.69 | -0.01 | 0.00 | 4.00E-09 | 32.74 |
| 368 | rs7334078 | C | T | 0.29 | -0.01 | 0.00 | 2.20E-10 | 40.56 |
| 369 | rs1218822 | A | G | 0.67 | 0.02 | 0.00 | 1.90E-22 | 97.66 |
| 370 | rs7983065 | T | C | 0.45 | -0.01 | 0.00 | 8.90E-18 | 75.79 |
| 371 | rs12429545 | A | G | 0.12 | 0.03 | 0.00 | 9.60E-38 | 159.77 |
| 372 | rs8181823 | C | A | 0.76 | 0.01 | 0.00 | 4.10E-10 | 40.32 |
| 373 | rs9547153 | G | A | 0.38 | 0.01 | 0.00 | 8.70E-09 | 33.23 |
| 374 | rs1112613 | A | G | 0.18 | -0.01 | 0.00 | 3.40E-09 | 33.44 |
| 375 | rs9538162 | C | T | 0.41 | -0.02 | 0.00 | 4.80E-19 | 75.11 |
| 376 | rs1144387 | C | G | 0.57 | 0.01 | 0.00 | 1.60E-08 | 33.23 |
| 377 | rs7998796 | G | A | 0.34 | 0.01 | 0.00 | 1.10E-08 | 34.03 |
| 378 | rs2479958 | G | A | 0.51 | -0.02 | 0.00 | 1.50E-17 | 73.20 |
| 379 | rs17446257 | A | G | 0.13 | 0.02 | 0.00 | 2.90E-09 | 34.63 |
| 380 | rs9571687 | A | C | 0.33 | -0.01 | 0.00 | 2.80E-12 | 51.36 |
| 381 | rs1330052 | G | C | 0.35 | 0.01 | 0.00 | 1.50E-13 | 53.78 |
| 382 | rs3007105 | T | C | 0.47 | 0.01 | 0.00 | 1.10E-17 | 69.77 |
| 383 | rs4981693 | A | G | 0.77 | 0.02 | 0.00 | 6.90E-24 | 106.09 |
| 384 | rs226000 | T | C | 0.82 | 0.01 | 0.00 | 3.60E-08 | 29.26 |
| 385 | rs3902951 | G | T | 0.25 | 0.01 | 0.00 | 7.00E-12 | 44.89 |
| 386 | rs1285997 | G | C | 0.72 | 0.01 | 0.00 | 1.20E-13 | 55.86 |
| 387 | rs9989141 | T | C | 0.64 | 0.02 | 0.00 | 3.60E-21 | 90.81 |
| 388 | rs709400 | G | A | 0.38 | -0.02 | 0.00 | 4.60E-19 | 77.85 |
| 389 | rs7148846 | G | T | 0.19 | 0.01 | 0.00 | 2.20E-08 | 31.77 |
| 390 | rs4430672 | C | T | 0.80 | -0.01 | 0.00 | 3.90E-09 | 33.32 |
| 391 | rs217671 | G | A | 0.27 | 0.01 | 0.00 | 1.30E-13 | 57.44 |
| 392 | rs768840 | A | G | 0.42 | 0.01 | 0.00 | 2.00E-10 | 40.11 |
| 393 | rs7144011 | T | G | 0.21 | 0.03 | 0.00 | 5.20E-47 | 198.81 |
| 394 | rs12888545 | G | A | 0.25 | 0.01 | 0.00 | 9.10E-12 | 46.24 |
| 395 | rs10132280 | A | C | 0.30 | -0.02 | 0.00 | 5.60E-35 | 153.48 |
| 396 | rs872281 | T | C | 0.17 | -0.02 | 0.00 | 4.70E-11 | 43.10 |
| 397 | rs12888955 | A | G | 0.65 | -0.02 | 0.00 | 1.40E-22 | 97.79 |
| 398 | rs1836303 | G | A | 0.39 | 0.01 | 0.00 | 5.30E-11 | 41.53 |
| 399 | rs3736485 | G | A | 0.54 | -0.01 | 0.00 | 2.50E-16 | 70.14 |
| 400 | rs339991 | G | A | 0.56 | 0.01 | 0.00 | 1.20E-12 | 47.46 |
| 401 | rs12593036 | G | A | 0.30 | -0.02 | 0.00 | 3.80E-16 | 65.70 |
| 402 | rs4906908 | G | T | 0.53 | 0.01 | 0.00 | 2.50E-09 | 36.71 |
| 403 | rs8036040 | A | C | 0.49 | 0.01 | 0.00 | 2.70E-10 | 41.11 |
| 404 | rs10518694 | A | C | 0.14 | 0.01 | 0.00 | 3.30E-09 | 34.11 |
| 405 | rs17238110 | G | A | 0.16 | -0.04 | 0.01 | 2.00E-12 | 49.84 |
| 406 | rs16953563 | A | G | 0.25 | -0.01 | 0.00 | 1.50E-11 | 44.89 |
| 407 | rs11855853 | T | C | 0.26 | -0.01 | 0.00 | 2.40E-13 | 52.56 |
| 408 | rs7172627 | G | A | 0.47 | 0.01 | 0.00 | 1.10E-11 | 47.37 |
| 409 | rs17311369 | T | C | 0.33 | -0.01 | 0.00 | 3.10E-08 | 29.96 |
| 410 | rs13329567 | T | C | 0.23 | -0.03 | 0.00 | 1.00E-50 | 214.62 |
| 411 | rs7181498 | C | T | 0.63 | -0.02 | 0.00 | 1.00E-19 | 82.00 |
| 412 | rs12905439 | G | C | 0.34 | -0.01 | 0.00 | 1.40E-10 | 42.98 |
| 413 | rs9806742 | A | G | 0.88 | 0.02 | 0.00 | 1.40E-15 | 64.00 |
| 414 | rs12914489 | A | G | 0.11 | 0.02 | 0.00 | 3.80E-10 | 40.27 |
| 415 | rs8027205 | G | C | 0.40 | -0.01 | 0.00 | 1.40E-09 | 36.00 |
| 416 | rs12448257 | A | G | 0.22 | 0.02 | 0.00 | 8.10E-20 | 84.64 |
| 417 | rs879620 | T | C | 0.62 | 0.02 | 0.00 | 5.30E-38 | 164.69 |
| 418 | rs9926784 | C | T | 0.18 | -0.03 | 0.00 | 9.90E-35 | 150.94 |
| 419 | rs9927848 | A | C | 0.73 | -0.01 | 0.00 | 6.40E-10 | 37.21 |
| 420 | rs1477199 | G | A | 0.15 | 0.02 | 0.00 | 9.40E-22 | 90.25 |
| 421 | rs825688 | T | C | 0.46 | -0.01 | 0.00 | 4.70E-08 | 31.23 |
| 422 | rs2361988 | C | T | 0.25 | -0.02 | 0.00 | 5.20E-15 | 60.06 |
| 423 | rs1896767 | A | G | 0.54 | -0.01 | 0.00 | 2.40E-10 | 41.11 |
| 424 | rs4786903 | G | A | 0.74 | 0.01 | 0.00 | 3.50E-10 | 39.06 |
| 425 | rs7196720 | C | T | 0.51 | -0.01 | 0.00 | 7.30E-14 | 57.58 |
| 426 | rs3814883 | T | C | 0.48 | 0.02 | 0.00 | 1.10E-40 | 186.24 |
| 427 | rs8047395 | A | G | 0.51 | 0.06 | 0.00 | 1.00E-200 | 1426.17 |
| 428 | rs12922346 | C | G | 0.27 | 0.01 | 0.00 | 1.00E-11 | 46.24 |
| 429 | rs4783830 | A | G | 0.31 | -0.01 | 0.00 | 2.40E-08 | 30.54 |
| 430 | rs889398 | T | C | 0.42 | -0.02 | 0.00 | 1.30E-32 | 150.06 |
| 431 | rs12933482 | G | A | 0.10 | 0.02 | 0.00 | 4.90E-11 | 44.13 |
| 432 | rs7206608 | G | C | 0.31 | 0.01 | 0.00 | 1.30E-12 | 48.27 |
| 433 | rs7498665 | G | A | 0.40 | 0.03 | 0.00 | 5.60E-60 | 254.12 |
| 434 | rs6500208 | A | G | 0.20 | 0.01 | 0.00 | 4.10E-12 | 49.00 |
| 435 | rs756717 | A | G | 0.40 | -0.01 | 0.00 | 5.40E-18 | 75.79 |
| 436 | rs4516268 | A | C | 0.19 | -0.02 | 0.00 | 5.20E-25 | 106.78 |
| 437 | rs4986044 | T | C | 0.47 | -0.02 | 0.00 | 3.30E-23 | 105.06 |
| 438 | rs208015 | C | T | 0.92 | -0.04 | 0.00 | 1.40E-25 | 109.63 |
| 439 | rs4968656 | G | A | 0.32 | 0.01 | 0.00 | 8.20E-10 | 37.27 |
| 440 | rs12939549 | G | A | 0.43 | -0.02 | 0.00 | 2.70E-28 | 126.56 |
| 441 | rs12936083 | G | A | 0.33 | 0.01 | 0.00 | 4.10E-13 | 53.52 |
| 442 | rs12150665 | C | T | 0.41 | -0.02 | 0.00 | 1.60E-22 | 90.81 |
| 443 | rs2411182 | A | G | 0.69 | 0.01 | 0.00 | 7.70E-11 | 41.91 |
| 444 | rs962273 | C | T | 0.71 | 0.01 | 0.00 | 2.60E-13 | 51.99 |
| 445 | rs3935648 | G | C | 0.23 | -0.01 | 0.00 | 6.80E-09 | 32.28 |
| 446 | rs1075901 | C | T | 0.56 | 0.01 | 0.00 | 1.20E-13 | 57.19 |
| 447 | rs7222349 | A | G | 0.34 | 0.01 | 0.00 | 3.30E-10 | 40.82 |
| 448 | rs391300 | C | T | 0.63 | -0.01 | 0.00 | 3.10E-12 | 49.00 |
| 449 | rs11656076 | A | G | 0.23 | -0.01 | 0.00 | 5.60E-12 | 45.72 |
| 450 | rs8071182 | A | G | 0.17 | 0.01 | 0.00 | 2.10E-09 | 36.55 |
| 451 | rs12602912 | T | C | 0.20 | 0.02 | 0.00 | 9.90E-18 | 70.24 |
| 452 | rs8097672 | T | A | 0.15 | 0.02 | 0.00 | 8.40E-16 | 64.00 |
| 453 | rs1430387 | C | T | 0.43 | -0.01 | 0.00 | 5.80E-11 | 44.97 |
| 454 | rs9783858 | T | C | 0.52 | 0.01 | 0.00 | 3.30E-08 | 28.65 |
| 455 | rs8097783 | A | G | 0.08 | -0.04 | 0.00 | 7.20E-36 | 157.46 |
| 456 | rs17710386 | C | T | 0.33 | 0.01 | 0.00 | 1.00E-12 | 49.00 |
| 457 | rs11150911 | C | A | 0.72 | -0.01 | 0.00 | 4.70E-13 | 54.60 |
| 458 | rs4800191 | C | G | 0.64 | 0.01 | 0.00 | 2.50E-09 | 36.71 |
| 459 | rs1365466 | T | C | 0.74 | -0.01 | 0.00 | 3.30E-13 | 51.99 |
| 460 | rs559231 | T | G | 0.40 | 0.01 | 0.00 | 2.40E-14 | 56.25 |
| 461 | rs663129 | A | G | 0.23 | 0.05 | 0.00 | 1.60E-178 | 822.78 |
| 462 | rs1241986 | A | G | 0.85 | -0.01 | 0.00 | 1.10E-08 | 33.54 |
| 463 | rs7239575 | C | T | 0.48 | -0.02 | 0.00 | 7.40E-32 | 141.19 |
| 464 | rs1158805 | A | C | 0.38 | -0.01 | 0.00 | 1.20E-14 | 57.93 |
| 465 | rs8090983 | G | A | 0.33 | 0.01 | 0.00 | 2.00E-10 | 42.98 |
| 466 | rs9951619 | G | T | 0.76 | 0.02 | 0.00 | 1.40E-15 | 60.84 |
| 467 | rs287104 | A | G | 0.66 | 0.01 | 0.00 | 4.40E-11 | 45.76 |
| 468 | rs895330 | G | C | 0.19 | -0.02 | 0.00 | 5.50E-19 | 76.37 |
| 469 | rs273504 | G | A | 0.43 | 0.02 | 0.00 | 4.40E-18 | 72.25 |
| 470 | rs17724992 | G | A | 0.26 | -0.02 | 0.00 | 1.00E-22 | 92.77 |
| 471 | rs11880870 | G | A | 0.48 | -0.02 | 0.00 | 1.00E-28 | 123.60 |
| 472 | rs1982725 | T | C | 0.48 | 0.01 | 0.00 | 3.30E-08 | 32.56 |
| 473 | rs11084553 | G | A | 0.15 | -0.02 | 0.00 | 1.80E-18 | 76.56 |
| 474 | rs11672660 | T | C | 0.20 | -0.03 | 0.00 | 1.70E-60 | 262.13 |
| 475 | rs9304665 | A | T | 0.76 | 0.02 | 0.00 | 2.90E-29 | 131.10 |
| 476 | rs10408324 | T | C | 0.27 | -0.01 | 0.00 | 9.50E-11 | 42.59 |
| 477 | rs12981256 | A | G | 0.53 | 0.01 | 0.00 | 1.10E-15 | 62.23 |
| 478 | rs998732 | G | A | 0.16 | -0.02 | 0.00 | 2.00E-14 | 60.42 |
| 479 | rs17513613 | C | T | 0.32 | 0.02 | 0.00 | 3.60E-26 | 106.78 |
| 480 | rs769449 | A | G | 0.12 | -0.03 | 0.00 | 2.30E-20 | 88.50 |
| 481 | rs6050446 | G | A | 0.97 | 0.03 | 0.00 | 4.40E-13 | 53.26 |
| 482 | rs11908637 | A | G | 0.24 | -0.01 | 0.00 | 4.90E-09 | 32.65 |
| 483 | rs6011457 | A | T | 0.50 | -0.01 | 0.00 | 2.70E-11 | 46.56 |
| 484 | rs4813619 | T | G | 0.51 | -0.01 | 0.00 | 2.30E-09 | 36.00 |
| 485 | rs1321432 | C | A | 0.63 | 0.02 | 0.00 | 3.50E-29 | 124.69 |
| 486 | rs4012234 | G | T | 0.59 | 0.01 | 0.00 | 9.90E-16 | 61.36 |
| 487 | rs2143253 | A | G | 0.12 | -0.02 | 0.00 | 1.10E-12 | 52.28 |
| 488 | rs2425840 | C | A | 0.41 | 0.01 | 0.00 | 1.60E-11 | 43.71 |
| 489 | rs8123881 | G | A | 0.13 | 0.02 | 0.00 | 4.40E-16 | 66.69 |
| 490 | rs852056 | C | T | 0.76 | -0.01 | 0.00 | 1.80E-10 | 40.96 |
| 491 | rs1884389 | T | C | 0.43 | -0.01 | 0.00 | 4.00E-09 | 36.71 |
| 492 | rs2423668 | C | T | 0.55 | -0.01 | 0.00 | 2.80E-08 | 30.54 |
| 493 | rs1409818 | T | C | 0.12 | 0.02 | 0.00 | 2.50E-12 | 48.04 |
| 494 | rs17806379 | T | C | 0.18 | -0.03 | 0.00 | 1.50E-30 | 137.53 |
| 495 | rs6512302 | C | G | 0.75 | 0.01 | 0.00 | 2.10E-12 | 50.41 |
| 496 | rs427943 | C | A | 0.57 | 0.02 | 0.00 | 7.30E-23 | 100.00 |
| 497 | rs2832283 | A | G | 0.22 | 0.01 | 0.00 | 5.80E-09 | 33.06 |
| 498 | rs13047416 | G | C | 0.38 | -0.02 | 0.00 | 2.20E-17 | 73.20 |
| 499 | rs2836964 | C | T | 0.36 | -0.01 | 0.00 | 1.30E-09 | 37.35 |
| 500 | rs4818225 | G | A | 0.66 | 0.01 | 0.00 | 2.30E-10 | 42.25 |
| 501 | rs175165 | G | T | 0.39 | -0.01 | 0.00 | 5.20E-09 | 32.74 |
| 502 | rs9615905 | T | C | 0.45 | 0.01 | 0.00 | 2.70E-10 | 41.87 |
| 503 | rs11538 | G | A | 0.18 | 0.01 | 0.00 | 3.30E-09 | 34.45 |
| 504 | rs6712 | C | G | 0.14 | 0.01 | 0.00 | 4.40E-08 | 30.47 |
| 505 | rs138289 | T | A | 0.48 | -0.01 | 0.00 | 3.30E-09 | 36.71 |
| 506 | rs2285178 | C | T | 0.31 | 0.01 | 0.00 | 9.40E-09 | 34.75 |
| 507 | rs4820408 | G | T | 0.59 | -0.02 | 0.00 | 2.10E-19 | 78.90 |

EAF: Effect allele frequency; BMI: Body mass index

Table 11: Characteristics of SNPs used as instrumental variables for VLDL

|  | SNP | effect_allele.exposure | other_allele.exposure | eaf.exposure | beta.exposure | se.exposure | pval.exposure | F-Statistics |
| --- | --- | --- | --- | --- | --- | --- | --- | --- |
| 1 | rs3005923 | A | G | 0.03 | -0.33 | 0.04 | 9.25E-20 | 85.60 |
| 2 | rs190934192 | A | G | 0.02 | -0.40 | 0.04 | 1.40E-22 | 98.88 |
| 3 | rs553741 | C | G | 0.62 | 0.08 | 0.01 | 2.56E-12 | 50.69 |
| 4 | rs185415345 | A | G | 0.05 | -0.17 | 0.03 | 4.24E-10 | 40.34 |
| 5 | rs629301 | T | G | 0.78 | 0.13 | 0.01 | 1.05E-26 | 118.34 |
| 6 | rs11591147 | T | G | 0.03 | -0.52 | 0.03 | 8.60E-49 | 222.82 |
| 7 | rs10449300 | G | A | 0.34 | -0.06 | 0.01 | 9.40E-09 | 34.10 |
| 8 | rs193084249 | G | A | 0.03 | 0.18 | 0.03 | 8.68E-09 | 34.26 |
| 9 | rs934197 | A | G | 0.28 | 0.11 | 0.01 | 6.60E-23 | 100.43 |
| 10 | rs62120792 | C | T | 0.14 | -0.09 | 0.01 | 1.28E-09 | 38.11 |
| 11 | rs6756629 | A | G | 0.08 | -0.13 | 0.02 | 2.78E-12 | 50.52 |
| 12 | rs144064722 | G | A | 0.03 | 0.23 | 0.03 | 2.53E-11 | 46.04 |
| 13 | rs182695896 | C | A | 0.02 | 0.29 | 0.04 | 1.91E-12 | 51.28 |
| 14 | rs79225634 | T | C | 0.35 | 0.09 | 0.01 | 1.33E-17 | 75.46 |
| 15 | rs73066442 | G | A | 0.25 | 0.07 | 0.01 | 1.24E-08 | 33.54 |
| 16 | rs2980860 | G | A | 0.48 | -0.07 | 0.01 | 4.88E-10 | 40.06 |
| 17 | rs635634 | T | C | 0.20 | 0.07 | 0.01 | 1.24E-08 | 33.54 |
| 18 | rs3741298 | T | C | 0.77 | -0.07 | 0.01 | 2.78E-09 | 36.55 |
| 19 | rs34042070 | G | C | 0.20 | 0.07 | 0.01 | 1.54E-08 | 33.10 |
| 20 | rs2965146 | C | T | 0.40 | -0.06 | 0.01 | 4.56E-08 | 30.93 |
| 21 | rs137992968 | T | C | 0.02 | -0.22 | 0.03 | 8.32E-10 | 38.98 |
| 22 | rs8106814 | C | T | 0.22 | -0.10 | 0.01 | 9.68E-12 | 47.99 |
| 23 | rs142130958 | A | G | 0.10 | -0.24 | 0.02 | 1.19E-41 | 188.99 |
| 24 | rs7412 | T | C | 0.06 | -0.57 | 0.03 | 1.54E-105 | 492.00 |
| 25 | rs2207132 | A | G | 0.06 | 0.14 | 0.02 | 9.50E-09 | 34.08 |

EAF: Effect allele frequency; VLDL: Very low-density lipoprotein

Table 12: Characteristics of SNPs used as instrumental variables for hyperthyroidism

|  | SNP | effect_allele.exposure | other_allele.exposure | eaf.exposure | beta.exposure | se.exposure | pval.exposure | F-Statistics |
| --- | --- | --- | --- | --- | --- | --- | --- | --- |
| 1 | rs6679677 | A | C | 0.10 | 0.00 | 0.00 | 1.40E-16 | 68.24 |
| 2 | rs12741781 | G | T | 0.33 | 0.00 | 0.00 | 4.30E-09 | 34.50 |
| 3 | rs3087243 | A | G | 0.45 | 0.00 | 0.00 | 7.50E-24 | 101.40 |
| 4 | rs1559810 | A | C | 0.41 | 0.00 | 0.00 | 2.90E-08 | 30.76 |
| 5 | rs409602 | A | T | 0.14 | 0.00 | 0.00 | 4.60E-08 | 29.87 |
| 6 | rs1794279 | T | G | 0.13 | 0.01 | 0.00 | 6.50E-134 | 606.49 |
| 7 | rs71542456 | G | A | 0.20 | 0.00 | 0.00 | 2.90E-51 | 226.82 |
| 8 | rs185774696 | T | C | 0.30 | 0.00 | 0.00 | 3.20E-40 | 176.25 |
| 9 | rs9368644 | T | C | 0.15 | 0.00 | 0.00 | 2.80E-14 | 57.84 |
| 10 | rs10087240 | T | C | 0.46 | 0.00 | 0.00 | 3.30E-09 | 34.99 |
| 11 | rs7090530 | A | C | 0.60 | 0.00 | 0.00 | 4.50E-09 | 34.38 |
| 12 | rs4409785 | C | T | 0.17 | 0.00 | 0.00 | 9.20E-10 | 37.48 |
| 13 | rs2160215 | C | T | 0.38 | 0.00 | 0.00 | 1.70E-45 | 200.42 |

EAF: Effect allele frequency

Table 13: Characteristics of SNPs used as instrumental variables for hypothyroidism

|  | SNP | effect_allele.exposure | other_allele.exposure | eaf.exposure | beta.exposure | se.exposure | pval.exposure | F-Statistics |
| --- | --- | --- | --- | --- | --- | --- | --- | --- |
| 1 | rs17020110 | C | T | 0.27 | 0.00 | 0.00 | 2.07E-12 | 49.42 |
| 2 | rs6679677 | A | C | 0.10 | 0.02 | 0.00 | 1.07E-122 | 555.39 |
| 3 | rs12117927 | A | C | 0.49 | 0.00 | 0.00 | 8.18E-10 | 37.72 |
| 4 | rs4081335 | C | T | 0.05 | -0.01 | 0.00 | 3.55E-08 | 30.38 |
| 5 | rs6426808 | A | G | 0.51 | 0.00 | 0.00 | 3.13E-10 | 39.60 |
| 6 | rs926103 | C | T | 0.65 | 0.00 | 0.00 | 4.92E-08 | 29.75 |
| 7 | rs11675342 | T | C | 0.42 | 0.00 | 0.00 | 1.67E-18 | 77.05 |
| 8 | rs7582694 | G | C | 0.77 | -0.01 | 0.00 | 2.61E-29 | 126.35 |
| 9 | rs11571297 | C | T | 0.49 | -0.01 | 0.00 | 5.34E-61 | 271.61 |
| 10 | rs2111485 | G | A | 0.61 | 0.00 | 0.00 | 7.84E-13 | 51.32 |
| 11 | rs13399762 | G | A | 0.05 | 0.01 | 0.00 | 4.25E-09 | 34.51 |
| 12 | rs1534430 | T | C | 0.39 | 0.00 | 0.00 | 3.99E-14 | 57.18 |
| 13 | rs113229608 | A | C | 0.06 | 0.01 | 0.00 | 1.17E-09 | 37.02 |
| 14 | rs11706511 | G | A | 0.13 | 0.00 | 0.00 | 2.47E-09 | 35.57 |
| 15 | rs9815073 | A | C | 0.35 | -0.01 | 0.00 | 1.94E-35 | 154.39 |
| 16 | rs7649344 | C | T | 0.46 | 0.00 | 0.00 | 1.23E-08 | 32.43 |
| 17 | rs13090803 | T | G | 0.21 | 0.01 | 0.00 | 2.09E-15 | 62.98 |
| 18 | rs4276275 | T | C | 0.47 | 0.00 | 0.00 | 5.84E-11 | 42.88 |
| 19 | rs6833591 | G | A | 0.35 | 0.00 | 0.00 | 6.10E-09 | 33.80 |
| 20 | rs3775291 | T | C | 0.30 | 0.00 | 0.00 | 1.05E-12 | 50.74 |
| 21 | rs4444866 | T | C | 0.28 | 0.00 | 0.00 | 2.85E-08 | 30.81 |
| 22 | rs7441808 | G | A | 0.30 | 0.00 | 0.00 | 3.53E-10 | 39.36 |
| 23 | rs13145888 | C | T | 0.21 | -0.01 | 0.00 | 2.45E-21 | 89.95 |
| 24 | rs10036386 | T | C | 0.38 | 0.00 | 0.00 | 1.75E-09 | 36.23 |
| 25 | rs244672 | T | C | 0.88 | 0.00 | 0.00 | 4.84E-09 | 34.25 |
| 26 | rs28157 | T | G | 0.32 | 0.00 | 0.00 | 4.16E-10 | 39.04 |
| 27 | rs2736191 | G | C | 0.03 | -0.01 | 0.00 | 1.59E-08 | 31.94 |
| 28 | rs9497965 | T | C | 0.41 | 0.00 | 0.00 | 5.19E-12 | 47.62 |
| 29 | rs4263621 | A | G | 0.54 | 0.00 | 0.00 | 1.48E-08 | 32.09 |
| 30 | rs9272426 | G | A | 0.46 | 0.01 | 0.00 | 7.96E-121 | 546.76 |
| 31 | rs76518703 | G | A | 0.05 | -0.01 | 0.00 | 1.42E-16 | 68.28 |
| 32 | rs28418426 | C | T | 0.53 | 0.01 | 0.00 | 3.06E-58 | 258.95 |
| 33 | rs933243 | A | C | 0.33 | -0.01 | 0.00 | 3.90E-24 | 102.72 |
| 34 | rs7768019 | G | C | 0.25 | -0.01 | 0.00 | 1.31E-41 | 182.66 |
| 35 | rs654537 | A | G | 0.61 | 0.01 | 0.00 | 2.13E-29 | 126.75 |
| 36 | rs2523483 | G | T | 0.09 | -0.01 | 0.00 | 4.37E-13 | 52.47 |
| 37 | rs9277569 | T | C | 0.11 | 0.01 | 0.00 | 1.36E-23 | 100.24 |
| 38 | rs761357 | T | A | 0.38 | 0.00 | 0.00 | 6.81E-10 | 38.08 |
| 39 | rs221786 | C | T | 0.89 | 0.00 | 0.00 | 1.53E-09 | 36.50 |
| 40 | rs60600003 | G | T | 0.10 | 0.01 | 0.00 | 7.19E-09 | 33.48 |
| 41 | rs6992869 | C | T | 0.37 | 0.00 | 0.00 | 2.56E-08 | 31.01 |
| 42 | rs2921053 | C | G | 0.45 | 0.00 | 0.00 | 3.34E-19 | 80.23 |
| 43 | rs1032129 | C | A | 0.35 | 0.00 | 0.00 | 2.67E-08 | 30.93 |
| 44 | rs16903097 | G | T | 0.13 | 0.00 | 0.00 | 3.97E-09 | 34.64 |
| 45 | rs853303 | G | A | 0.62 | 0.00 | 0.00 | 2.30E-12 | 49.21 |
| 46 | rs10956412 | C | A | 0.16 | -0.01 | 0.00 | 5.84E-13 | 51.90 |
| 47 | rs11783023 | T | C | 0.72 | 0.00 | 0.00 | 1.06E-08 | 32.73 |
| 48 | rs911760 | A | C | 0.21 | 0.00 | 0.00 | 5.10E-10 | 38.64 |
| 49 | rs2123340 | A | G | 0.65 | 0.00 | 0.00 | 7.76E-12 | 46.83 |
| 50 | rs925489 | T | C | 0.67 | 0.01 | 0.00 | 1.52E-71 | 320.06 |
| 51 | rs11258303 | A | C | 0.75 | 0.00 | 0.00 | 5.59E-10 | 38.46 |
| 52 | rs71508903 | T | C | 0.19 | 0.01 | 0.00 | 3.21E-21 | 89.42 |
| 53 | rs3850765 | C | T | 0.59 | 0.00 | 0.00 | 2.76E-09 | 35.35 |
| 54 | rs7905731 | C | T | 0.59 | 0.00 | 0.00 | 2.66E-09 | 35.42 |
| 55 | rs683763 | T | G | 0.32 | 0.00 | 0.00 | 2.57E-08 | 31.01 |
| 56 | rs7090530 | A | C | 0.60 | 0.00 | 0.00 | 2.94E-15 | 62.31 |
| 57 | rs6584277 | G | A | 0.53 | 0.00 | 0.00 | 8.79E-10 | 37.58 |
| 58 | rs736374 | A | G | 0.36 | 0.00 | 0.00 | 2.48E-14 | 58.11 |
| 59 | rs4409785 | C | T | 0.17 | 0.01 | 0.00 | 4.31E-21 | 88.84 |
| 60 | rs3184504 | C | T | 0.52 | -0.01 | 0.00 | 1.10E-81 | 366.66 |
| 61 | rs11052877 | G | A | 0.37 | -0.01 | 0.00 | 9.31E-27 | 114.69 |
| 62 | rs12582330 | T | G | 0.73 | 0.00 | 0.00 | 1.10E-12 | 50.66 |
| 63 | rs705702 | G | A | 0.34 | 0.00 | 0.00 | 1.96E-11 | 45.01 |
| 64 | rs66749983 | T | A | 0.31 | 0.00 | 0.00 | 1.39E-10 | 41.18 |
| 65 | rs111618453 | A | G | 0.27 | 0.01 | 0.00 | 4.33E-23 | 97.95 |
| 66 | rs76428106 | C | T | 0.01 | 0.03 | 0.00 | 4.26E-30 | 129.95 |
| 67 | rs1045853 | C | A | 0.66 | 0.00 | 0.00 | 1.10E-09 | 37.15 |
| 68 | rs8008961 | T | C | 0.28 | 0.00 | 0.00 | 5.54E-09 | 33.99 |
| 69 | rs8043085 | T | G | 0.23 | 0.00 | 0.00 | 4.47E-12 | 47.91 |
| 70 | rs142997491 | G | A | 0.01 | 0.02 | 0.00 | 2.81E-10 | 39.80 |
| 71 | rs13333582 | C | T | 0.04 | 0.01 | 0.00 | 1.80E-08 | 31.70 |
| 72 | rs8054578 | G | A | 0.78 | 0.00 | 0.00 | 8.95E-09 | 33.06 |
| 73 | rs1088898 | T | G | 0.77 | 0.00 | 0.00 | 4.22E-08 | 30.04 |
| 74 | rs61759532 | T | C | 0.25 | 0.00 | 0.00 | 1.26E-11 | 45.88 |
| 75 | rs62076510 | G | T | 0.16 | 0.01 | 0.00 | 3.46E-13 | 52.93 |
| 76 | rs1790604 | G | A | 0.54 | 0.00 | 0.00 | 5.49E-10 | 38.50 |
| 77 | rs1549142 | T | C | 0.23 | 0.00 | 0.00 | 3.90E-11 | 43.67 |
| 78 | rs10424978 | A | C | 0.60 | 0.00 | 0.00 | 7.21E-15 | 60.55 |
| 79 | rs12980063 | G | A | 0.39 | 0.00 | 0.00 | 1.74E-10 | 40.75 |
| 80 | rs2745803 | G | A | 0.21 | 0.00 | 0.00 | 9.31E-09 | 32.98 |
| 81 | rs2823272 | A | T | 0.32 | 0.00 | 0.00 | 2.03E-10 | 40.44 |
| 82 | rs229540 | G | T | 0.42 | 0.01 | 0.00 | 8.83E-22 | 91.98 |
| 83 | rs2412974 | T | C | 0.36 | 0.00 | 0.00 | 8.01E-09 | 33.28 |

EAF: Effect allele frequency

Table 14: Characteristics of SNPs used as instrumental variables for insulin sensitivity

|  | SNP | effect_allele.exposure | other_allele.exposure | eaf.exposure | beta.exposure | se.exposure | pval.exposure | F-Statistics |
| --- | --- | --- | --- | --- | --- | --- | --- | --- |
| 1 | rs1857095 | T | C | NA | 0.88 | 0.25 | 5.40E-16 | 12.39 |
| 2 | rs2972146 | T | G | NA | 0.14 | 0.07 | 4.72E-10 | 3.58 |
| 3 | rs10495667 | A | G | NA | -0.90 | 0.18 | 3.83E-09 | 25.00 |
| 4 | rs3900087 | C | T | NA | -2.30 | 0.38 | 9.49E-10 | 36.63 |
| 5 | rs12522198 | A | G | NA | 1.80 | 0.28 | 1.18E-10 | 41.33 |
| 6 | rs16924527 | A | C | NA | -0.79 | 0.23 | 7.60E-10 | 11.80 |
| 7 | rs11790816 | T | C | NA | -1.40 | 0.26 | 3.56E-09 | 28.99 |
| 8 | rs11594101 | G | A | NA | -1.60 | 0.25 | 8.38E-10 | 40.96 |
| 9 | rs10506418 | A | G | NA | -0.80 | 0.25 | 2.09E-08 | 10.24 |
| 10 | rs12583553 | T | A | NA | -1.00 | 0.19 | 1.41E-10 | 27.70 |
| 11 | rs4078023 | T | G | NA | 1.30 | 0.23 | 3.41E-10 | 31.95 |
| 12 | rs6027072 | A | G | NA | -0.48 | 0.18 | 5.70E-09 | 7.11 |
| 13 | rs2828537 | T | A | NA | -1.10 | 0.17 | 1.45E-10 | 41.87 |
| 14 | rs10483182 | A | G | NA | -2.00 | 0.26 | 7.32E-15 | 59.17 |

EAF: Effect allele frequency

Table 15: Genetic association estimates for the effect of T2DM on CHD. ea=effect allele, gx=T2DM, gy=CHD, se=standard error

|  | SNP | ea | gx | gx_se | gy | gy_se |
| --- | --- | --- | --- | --- | --- | --- |
| 1 | rs10077431 | A | -0.05 | 0.01 | 0.01 | 0.01 |
| 2 | rs10087241 | A | -0.05 | 0.01 | -0.01 | 0.01 |
| 3 | rs10100265 | C | -0.05 | 0.01 | -0.02 | 0.01 |
| 4 | rs10114341 | C | -0.04 | 0.01 | -0.01 | 0.01 |
| 5 | rs10401969 | C | 0.09 | 0.01 | -0.05 | 0.02 |
| 6 | rs1050226 | G | -0.05 | 0.01 | -0.01 | 0.01 |
| 7 | rs1061813 | A | -0.04 | 0.01 | -0.01 | 0.01 |
| 8 | rs1063355 | G | 0.07 | 0.01 | 0.01 | 0.01 |
| 9 | rs10740322 | A | 0.05 | 0.01 | 0.00 | 0.01 |
| 10 | rs10811661 | C | -0.16 | 0.01 | 0.00 | 0.01 |
| 11 | rs10830963 | G | 0.09 | 0.01 | 0.02 | 0.01 |
| 12 | rs10842994 | T | -0.08 | 0.01 | 0.00 | 0.01 |
| 13 | rs10974438 | C | 0.06 | 0.01 | 0.00 | 0.01 |
| 14 | rs11098676 | C | 0.05 | 0.01 | -0.01 | 0.01 |
| 15 | rs11107116 | T | 0.05 | 0.01 | 0.00 | 0.01 |
| 16 | rs11257655 | T | 0.07 | 0.01 | 0.03 | 0.01 |
| 17 | rs1127655 | T | -0.04 | 0.01 | 0.00 | 0.01 |
| 18 | rs11708067 | G | -0.10 | 0.01 | 0.00 | 0.01 |
| 19 | rs11925227 | A | -0.05 | 0.01 | -0.01 | 0.01 |
| 20 | rs11926707 | C | 0.05 | 0.01 | -0.01 | 0.01 |
| 21 | rs12088739 | G | -0.09 | 0.01 | -0.05 | 0.02 |
| 22 | rs12299509 | G | 0.05 | 0.01 | 0.00 | 0.01 |
| 23 | rs12617659 | T | -0.07 | 0.01 | 0.00 | 0.01 |
| 24 | rs12910825 | G | 0.05 | 0.01 | 0.01 | 0.01 |
| 25 | rs12945601 | C | -0.05 | 0.01 | -0.03 | 0.01 |
| 26 | rs12970134 | A | 0.06 | 0.01 | 0.06 | 0.01 |
| 27 | rs13234269 | A | -0.06 | 0.01 | -0.02 | 0.01 |
| 28 | rs13239186 | T | 0.05 | 0.01 | 0.02 | 0.01 |
| 29 | rs1333039 | C | 0.05 | 0.01 | 0.12 | 0.02 |
| 30 | rs13330951 | G | -0.05 | 0.01 | -0.02 | 0.01 |
| 31 | rs13389219 | T | -0.07 | 0.01 | -0.02 | 0.01 |
| 32 | rs1359790 | A | -0.08 | 0.01 | -0.01 | 0.01 |
| 33 | rs1496653 | G | -0.08 | 0.01 | 0.01 | 0.01 |
| 34 | rs1552224 | C | -0.10 | 0.01 | 0.00 | 0.01 |
| 35 | rs16988333 | G | -0.07 | 0.01 | -0.03 | 0.02 |
| 36 | rs17086692 | T | -0.05 | 0.01 | 0.01 | 0.01 |
| 37 | rs17168486 | T | 0.07 | 0.01 | 0.02 | 0.01 |
| 38 | rs17334919 | T | -0.14 | 0.01 | 0.04 | 0.02 |
| 39 | rs17405722 | A | 0.09 | 0.01 | 0.04 | 0.02 |
| 40 | rs17411031 | G | -0.05 | 0.01 | -0.05 | 0.01 |
| 41 | rs1758632 | G | 0.05 | 0.01 | 0.00 | 0.01 |
| 42 | rs17631783 | T | -0.05 | 0.01 | -0.01 | 0.01 |
| 43 | rs17791483 | G | -0.10 | 0.01 | -0.01 | 0.02 |
| 44 | rs1801214 | T | 0.09 | 0.01 | 0.01 | 0.01 |
| 45 | rs1899951 | T | -0.11 | 0.01 | 0.00 | 0.01 |
| 46 | rs2191348 | T | 0.07 | 0.01 | 0.00 | 0.01 |
| 47 | rs2237892 | T | -0.10 | 0.02 | -0.01 | 0.02 |
| 48 | rs2246618 | T | 0.05 | 0.01 | 0.00 | 0.01 |
| 49 | rs2261181 | T | 0.10 | 0.01 | 0.02 | 0.01 |
| 50 | rs2292662 | T | -0.06 | 0.01 | -0.02 | 0.01 |
| 51 | rs2294120 | G | -0.04 | 0.01 | 0.01 | 0.01 |
| 52 | rs2296173 | G | 0.07 | 0.01 | 0.02 | 0.01 |
| 53 | rs2299383 | T | 0.04 | 0.01 | 0.01 | 0.01 |
| 54 | rs243019 | C | 0.06 | 0.01 | 0.02 | 0.01 |
| 55 | rs2493394 | G | 0.07 | 0.01 | -0.02 | 0.01 |
| 56 | rs2796441 | A | -0.07 | 0.01 | -0.01 | 0.01 |
| 57 | rs2820426 | G | 0.05 | 0.01 | 0.01 | 0.01 |
| 58 | rs2867125 | C | 0.06 | 0.01 | 0.04 | 0.01 |
| 59 | rs2908282 | A | 0.06 | 0.01 | 0.01 | 0.01 |
| 60 | rs2925979 | C | -0.05 | 0.01 | -0.02 | 0.01 |
| 61 | rs2972144 | G | 0.09 | 0.01 | 0.04 | 0.01 |
| 62 | rs340874 | C | 0.06 | 0.01 | -0.01 | 0.01 |
| 63 | rs348330 | A | -0.05 | 0.01 | -0.01 | 0.01 |
| 64 | rs3756784 | G | 0.05 | 0.01 | 0.02 | 0.01 |
| 65 | rs3802177 | A | -0.12 | 0.01 | -0.01 | 0.01 |
| 66 | rs4506565 | T | 0.28 | 0.01 | 0.03 | 0.01 |
| 67 | rs459193 | G | 0.07 | 0.01 | 0.03 | 0.01 |
| 68 | rs4686471 | C | 0.05 | 0.01 | 0.00 | 0.01 |
| 69 | rs4810426 | T | 0.07 | 0.01 | 0.01 | 0.01 |
| 70 | rs4823182 | G | 0.05 | 0.01 | 0.00 | 0.01 |
| 71 | rs4865796 | A | 0.05 | 0.01 | 0.01 | 0.01 |
| 72 | rs516946 | C | 0.08 | 0.01 | 0.00 | 0.01 |
| 73 | rs5215 | T | -0.07 | 0.01 | -0.01 | 0.01 |
| 74 | rs576674 | A | -0.07 | 0.01 | 0.00 | 0.01 |
| 75 | rs6059662 | G | 0.04 | 0.01 | 0.00 | 0.01 |
| 76 | rs6066138 | A | -0.05 | 0.01 | -0.01 | 0.01 |
| 77 | rs61953351 | T | -0.07 | 0.01 | -0.02 | 0.01 |
| 78 | rs622217 | C | -0.05 | 0.01 | -0.04 | 0.01 |
| 79 | rs6494307 | G | -0.04 | 0.01 | 0.00 | 0.01 |
| 80 | rs6515236 | C | -0.05 | 0.01 | 0.00 | 0.01 |
| 81 | rs67232546 | T | 0.06 | 0.01 | -0.01 | 0.01 |
| 82 | rs6795735 | T | -0.06 | 0.01 | 0.01 | 0.01 |
| 83 | rs6878122 | A | -0.06 | 0.01 | -0.02 | 0.01 |
| 84 | rs7138300 | T | -0.04 | 0.01 | 0.00 | 0.01 |
| 85 | rs7144011 | T | 0.05 | 0.01 | 0.02 | 0.01 |
| 86 | rs7177055 | A | 0.06 | 0.01 | 0.01 | 0.01 |
| 87 | rs7185735 | G | 0.11 | 0.01 | 0.03 | 0.01 |
| 88 | rs7240767 | C | 0.05 | 0.01 | 0.02 | 0.01 |
| 89 | rs72892910 | T | 0.06 | 0.01 | 0.02 | 0.01 |
| 90 | rs735949 | C | -0.07 | 0.01 | 0.00 | 0.01 |
| 91 | rs753270 | C | 0.05 | 0.01 | 0.01 | 0.01 |
| 92 | rs7561798 | G | 0.04 | 0.01 | 0.03 | 0.01 |
| 93 | rs7572970 | G | 0.06 | 0.01 | 0.04 | 0.01 |
| 94 | rs7619041 | A | -0.04 | 0.01 | -0.01 | 0.01 |
| 95 | rs7651090 | G | 0.12 | 0.01 | 0.01 | 0.01 |
| 96 | rs7674212 | T | -0.05 | 0.01 | 0.01 | 0.01 |
| 97 | rs7685296 | T | -0.05 | 0.01 | -0.02 | 0.01 |
| 98 | rs77258096 | A | -0.12 | 0.01 | -0.06 | 0.02 |
| 99 | rs7729395 | T | 0.14 | 0.02 | -0.03 | 0.03 |
| 100 | rs7756992 | G | 0.13 | 0.01 | 0.02 | 0.01 |
| 101 | rs7786095 | G | -0.07 | 0.01 | -0.01 | 0.02 |
| 102 | rs780094 | C | 0.07 | 0.01 | 0.00 | 0.01 |
| 103 | rs7845219 | C | -0.04 | 0.01 | -0.02 | 0.01 |
| 104 | rs7923866 | T | -0.10 | 0.01 | -0.02 | 0.01 |
| 105 | rs7929543 | C | 0.08 | 0.01 | 0.01 | 0.01 |
| 106 | rs8068804 | A | 0.06 | 0.01 | 0.01 | 0.01 |
| 107 | rs8108269 | G | 0.06 | 0.01 | 0.00 | 0.01 |
| 108 | rs825476 | T | 0.05 | 0.01 | 0.02 | 0.01 |
| 109 | rs840967 | A | -0.05 | 0.01 | -0.01 | 0.01 |
| 110 | rs849135 | A | -0.10 | 0.01 | -0.01 | 0.01 |
| 111 | rs853974 | C | -0.06 | 0.01 | 0.02 | 0.01 |
| 112 | rs9369425 | A | -0.05 | 0.01 | 0.01 | 0.01 |
| 113 | rs963740 | T | -0.05 | 0.01 | -0.01 | 0.01 |
| 114 | rs982077 | G | -0.05 | 0.01 | -0.01 | 0.01 |
| 115 | rs9844972 | C | 0.10 | 0.01 | 0.04 | 0.02 |
| 116 | rs9894220 | G | -0.06 | 0.01 | -0.04 | 0.01 |
| 117 | rs993380 | G | -0.05 | 0.01 | -0.01 | 0.01 |
| 118 | rs9940149 | A | -0.06 | 0.01 | 0.00 | 0.01 |

T2DM: Type-2 diabetes; CHD: Coronary heart disease

Table 16: Genetic association estimates for the effect of T2DM on MI. ea=effect allele, gx=T2DM, gy=MI, se=standard error

|  | SNP | ea | gx | gx_se | gy | gy_se |
| --- | --- | --- | --- | --- | --- | --- |
| 1 | rs10077431 | A | -0.05 | 0.01 | 0.02 | 0.01 |
| 2 | rs10087241 | A | -0.05 | 0.01 | -0.01 | 0.01 |
| 3 | rs10100265 | C | -0.05 | 0.01 | -0.01 | 0.01 |
| 4 | rs10114341 | C | -0.04 | 0.01 | -0.01 | 0.01 |
| 5 | rs10401969 | C | 0.09 | 0.01 | -0.04 | 0.02 |
| 6 | rs1050226 | G | -0.05 | 0.01 | 0.00 | 0.01 |
| 7 | rs1061813 | A | -0.04 | 0.01 | 0.00 | 0.01 |
| 8 | rs1063355 | G | 0.07 | 0.01 | 0.01 | 0.01 |
| 9 | rs10740322 | A | 0.05 | 0.01 | 0.01 | 0.01 |
| 10 | rs10811661 | C | -0.16 | 0.01 | 0.00 | 0.01 |
| 11 | rs10830963 | G | 0.09 | 0.01 | 0.02 | 0.01 |
| 12 | rs10842994 | T | -0.08 | 0.01 | 0.01 | 0.01 |
| 13 | rs10974438 | C | 0.06 | 0.01 | 0.00 | 0.01 |
| 14 | rs11098676 | C | 0.05 | 0.01 | -0.01 | 0.01 |
| 15 | rs11107116 | T | 0.05 | 0.01 | 0.01 | 0.01 |
| 16 | rs11257655 | T | 0.07 | 0.01 | 0.02 | 0.01 |
| 17 | rs1127655 | T | -0.04 | 0.01 | 0.00 | 0.01 |
| 18 | rs11708067 | G | -0.10 | 0.01 | -0.01 | 0.01 |
| 19 | rs11925227 | A | -0.05 | 0.01 | -0.01 | 0.01 |
| 20 | rs11926707 | C | 0.05 | 0.01 | -0.01 | 0.01 |
| 21 | rs12088739 | G | -0.09 | 0.01 | -0.05 | 0.02 |
| 22 | rs12299509 | G | 0.05 | 0.01 | 0.01 | 0.01 |
| 23 | rs12617659 | T | -0.07 | 0.01 | -0.01 | 0.01 |
| 24 | rs12910825 | G | 0.05 | 0.01 | -0.01 | 0.01 |
| 25 | rs12945601 | C | -0.05 | 0.01 | -0.04 | 0.01 |
| 26 | rs12970134 | A | 0.06 | 0.01 | 0.05 | 0.01 |
| 27 | rs13234269 | A | -0.06 | 0.01 | -0.02 | 0.01 |
| 28 | rs13239186 | T | 0.05 | 0.01 | 0.03 | 0.01 |
| 29 | rs1333039 | C | 0.05 | 0.01 | 0.11 | 0.02 |
| 30 | rs13330951 | G | -0.05 | 0.01 | -0.02 | 0.01 |
| 31 | rs13389219 | T | -0.07 | 0.01 | -0.01 | 0.01 |
| 32 | rs1359790 | A | -0.08 | 0.01 | -0.01 | 0.01 |
| 33 | rs1496653 | G | -0.08 | 0.01 | 0.01 | 0.01 |
| 34 | rs1552224 | C | -0.10 | 0.01 | -0.02 | 0.01 |
| 35 | rs16988333 | G | -0.07 | 0.01 | -0.05 | 0.02 |
| 36 | rs17086692 | T | -0.05 | 0.01 | -0.01 | 0.01 |
| 37 | rs17168486 | T | 0.07 | 0.01 | 0.01 | 0.01 |
| 38 | rs17334919 | T | -0.14 | 0.01 | 0.03 | 0.02 |
| 39 | rs17405722 | A | 0.09 | 0.01 | 0.03 | 0.02 |
| 40 | rs17411031 | G | -0.05 | 0.01 | -0.04 | 0.01 |
| 41 | rs1758632 | G | 0.05 | 0.01 | 0.01 | 0.01 |
| 42 | rs17631783 | T | -0.05 | 0.01 | -0.01 | 0.01 |
| 43 | rs17791483 | G | -0.10 | 0.01 | -0.01 | 0.02 |
| 44 | rs1801214 | T | 0.09 | 0.01 | 0.00 | 0.01 |
| 45 | rs1899951 | T | -0.11 | 0.01 | 0.02 | 0.02 |
| 46 | rs2191348 | T | 0.07 | 0.01 | 0.01 | 0.01 |
| 47 | rs2237892 | T | -0.10 | 0.02 | -0.02 | 0.02 |
| 48 | rs2246618 | T | 0.05 | 0.01 | -0.01 | 0.01 |
| 49 | rs2261181 | T | 0.10 | 0.01 | 0.01 | 0.02 |
| 50 | rs2292662 | T | -0.06 | 0.01 | -0.02 | 0.01 |
| 51 | rs2294120 | G | -0.04 | 0.01 | 0.02 | 0.01 |
| 52 | rs2296173 | G | 0.07 | 0.01 | 0.02 | 0.01 |
| 53 | rs2299383 | T | 0.04 | 0.01 | -0.01 | 0.01 |
| 54 | rs243019 | C | 0.06 | 0.01 | 0.00 | 0.01 |
| 55 | rs2493394 | G | 0.07 | 0.01 | -0.01 | 0.02 |
| 56 | rs2796441 | A | -0.07 | 0.01 | -0.01 | 0.01 |
| 57 | rs2820426 | G | 0.05 | 0.01 | 0.00 | 0.01 |
| 58 | rs2867125 | C | 0.06 | 0.01 | 0.01 | 0.01 |
| 59 | rs2908282 | A | 0.06 | 0.01 | 0.01 | 0.01 |
| 60 | rs2925979 | C | -0.05 | 0.01 | -0.01 | 0.01 |
| 61 | rs2972144 | G | 0.09 | 0.01 | 0.04 | 0.01 |
| 62 | rs340874 | C | 0.06 | 0.01 | 0.00 | 0.01 |
| 63 | rs348330 | A | -0.05 | 0.01 | 0.00 | 0.01 |
| 64 | rs3756784 | G | 0.05 | 0.01 | 0.03 | 0.01 |
| 65 | rs3802177 | A | -0.12 | 0.01 | 0.00 | 0.01 |
| 66 | rs4506565 | T | 0.28 | 0.01 | 0.02 | 0.01 |
| 67 | rs459193 | G | 0.07 | 0.01 | 0.03 | 0.01 |
| 68 | rs4686471 | C | 0.05 | 0.01 | 0.00 | 0.01 |
| 69 | rs4810426 | T | 0.07 | 0.01 | 0.01 | 0.01 |
| 70 | rs4823182 | G | 0.05 | 0.01 | -0.01 | 0.01 |
| 71 | rs4865796 | A | 0.05 | 0.01 | 0.01 | 0.01 |
| 72 | rs516946 | C | 0.08 | 0.01 | 0.02 | 0.01 |
| 73 | rs5215 | T | -0.07 | 0.01 | -0.02 | 0.01 |
| 74 | rs576674 | A | -0.07 | 0.01 | 0.00 | 0.01 |
| 75 | rs6059662 | G | 0.04 | 0.01 | 0.00 | 0.01 |
| 76 | rs6066138 | A | -0.05 | 0.01 | -0.01 | 0.01 |
| 77 | rs61953351 | T | -0.07 | 0.01 | -0.03 | 0.01 |
| 78 | rs622217 | C | -0.05 | 0.01 | -0.03 | 0.01 |
| 79 | rs6494307 | G | -0.04 | 0.01 | 0.01 | 0.01 |
| 80 | rs6515236 | C | -0.05 | 0.01 | 0.00 | 0.01 |
| 81 | rs67232546 | T | 0.06 | 0.01 | -0.01 | 0.01 |
| 82 | rs6795735 | T | -0.06 | 0.01 | 0.01 | 0.01 |
| 83 | rs6878122 | A | -0.06 | 0.01 | -0.02 | 0.01 |
| 84 | rs7138300 | T | -0.04 | 0.01 | -0.01 | 0.01 |
| 85 | rs7144011 | T | 0.05 | 0.01 | 0.02 | 0.01 |
| 86 | rs7177055 | A | 0.06 | 0.01 | 0.02 | 0.01 |
| 87 | rs7185735 | G | 0.11 | 0.01 | 0.02 | 0.01 |
| 88 | rs7240767 | C | 0.05 | 0.01 | 0.02 | 0.01 |
| 89 | rs72892910 | T | 0.06 | 0.01 | 0.01 | 0.01 |
| 90 | rs735949 | C | -0.07 | 0.01 | 0.00 | 0.02 |
| 91 | rs753270 | C | 0.05 | 0.01 | 0.01 | 0.01 |
| 92 | rs7561798 | G | 0.04 | 0.01 | 0.04 | 0.01 |
| 93 | rs7572970 | G | 0.06 | 0.01 | 0.04 | 0.01 |
| 94 | rs7619041 | A | -0.04 | 0.01 | -0.01 | 0.01 |
| 95 | rs7651090 | G | 0.12 | 0.01 | 0.02 | 0.01 |
| 96 | rs7674212 | T | -0.05 | 0.01 | 0.00 | 0.01 |
| 97 | rs7685296 | T | -0.05 | 0.01 | -0.02 | 0.01 |
| 98 | rs77258096 | A | -0.12 | 0.01 | -0.05 | 0.02 |
| 99 | rs7729395 | T | 0.14 | 0.02 | -0.05 | 0.03 |
| 100 | rs7756992 | G | 0.13 | 0.01 | 0.01 | 0.01 |
| 101 | rs7786095 | G | -0.07 | 0.01 | -0.01 | 0.02 |
| 102 | rs780094 | C | 0.07 | 0.01 | 0.00 | 0.01 |
| 103 | rs7845219 | C | -0.04 | 0.01 | -0.02 | 0.01 |
| 104 | rs7923866 | T | -0.10 | 0.01 | -0.02 | 0.01 |
| 105 | rs7929543 | C | 0.08 | 0.01 | 0.03 | 0.02 |
| 106 | rs8068804 | A | 0.06 | 0.01 | 0.01 | 0.01 |
| 107 | rs8108269 | G | 0.06 | 0.01 | 0.01 | 0.01 |
| 108 | rs825476 | T | 0.05 | 0.01 | 0.03 | 0.01 |
| 109 | rs840967 | A | -0.05 | 0.01 | -0.01 | 0.01 |
| 110 | rs849135 | A | -0.10 | 0.01 | -0.01 | 0.01 |
| 111 | rs853974 | C | -0.06 | 0.01 | 0.02 | 0.01 |
| 112 | rs9369425 | A | -0.05 | 0.01 | 0.01 | 0.01 |
| 113 | rs963740 | T | -0.05 | 0.01 | -0.01 | 0.01 |
| 114 | rs982077 | G | -0.05 | 0.01 | 0.00 | 0.01 |
| 115 | rs9844972 | C | 0.10 | 0.01 | 0.04 | 0.02 |
| 116 | rs9894220 | G | -0.06 | 0.01 | -0.04 | 0.01 |
| 117 | rs993380 | G | -0.05 | 0.01 | -0.01 | 0.01 |
| 118 | rs9940149 | A | -0.06 | 0.01 | 0.01 | 0.01 |

T2DM: Type-2 diabetes; MI: Myocardial infarction

Table 17: Genetic association estimates for the effect of T2DM on stroke. ea=effect allele, gx=T2DM, gy=stroke, gz=SBP, se=standard error

|  | SNP | ea | gx | gx_se | gy | gy_se |
| --- | --- | --- | --- | --- | --- | --- |
| 1 | rs10077431 | A | -0.05 | 0.01 | 0.00 | 0.01 |
| 2 | rs10087241 | A | -0.05 | 0.01 | 0.01 | 0.01 |
| 3 | rs10100265 | C | -0.05 | 0.01 | -0.02 | 0.01 |
| 4 | rs10114341 | C | -0.04 | 0.01 | 0.00 | 0.01 |
| 5 | rs10401969 | C | 0.09 | 0.01 | 0.01 | 0.01 |
| 6 | rs1050226 | G | -0.05 | 0.01 | 0.00 | 0.01 |
| 7 | rs1061813 | A | -0.04 | 0.01 | -0.01 | 0.01 |
| 8 | rs1063355 | G | 0.07 | 0.01 | -0.01 | 0.01 |
| 9 | rs10740322 | A | 0.05 | 0.01 | -0.01 | 0.01 |
| 10 | rs10811661 | C | -0.16 | 0.01 | -0.01 | 0.01 |
| 11 | rs10830963 | G | 0.09 | 0.01 | 0.00 | 0.01 |
| 12 | rs10842994 | T | -0.08 | 0.01 | -0.01 | 0.01 |
| 13 | rs10974438 | C | 0.06 | 0.01 | 0.01 | 0.01 |
| 14 | rs11098676 | C | 0.05 | 0.01 | 0.00 | 0.01 |
| 15 | rs11107116 | T | 0.05 | 0.01 | 0.01 | 0.01 |
| 16 | rs11257655 | T | 0.07 | 0.01 | 0.01 | 0.01 |
| 17 | rs1127655 | T | -0.04 | 0.01 | 0.00 | 0.01 |
| 18 | rs11708067 | G | -0.10 | 0.01 | -0.03 | 0.01 |
| 19 | rs11925227 | A | -0.05 | 0.01 | 0.01 | 0.01 |
| 20 | rs11926707 | C | 0.05 | 0.01 | -0.02 | 0.01 |
| 21 | rs12088739 | G | -0.09 | 0.01 | 0.00 | 0.01 |
| 22 | rs12299509 | G | 0.05 | 0.01 | 0.00 | 0.01 |
| 23 | rs12617659 | T | -0.07 | 0.01 | 0.02 | 0.01 |
| 24 | rs12910825 | G | 0.05 | 0.01 | 0.00 | 0.01 |
| 25 | rs12945601 | C | -0.05 | 0.01 | -0.02 | 0.01 |
| 26 | rs12970134 | A | 0.06 | 0.01 | 0.02 | 0.01 |
| 27 | rs13234269 | A | -0.06 | 0.01 | -0.02 | 0.01 |
| 28 | rs13239186 | T | 0.05 | 0.01 | 0.03 | 0.01 |
| 29 | rs1333039 | C | 0.05 | 0.01 | 0.03 | 0.01 |
| 30 | rs13330951 | G | -0.05 | 0.01 | -0.01 | 0.01 |
| 31 | rs13389219 | T | -0.07 | 0.01 | -0.02 | 0.01 |
| 32 | rs1359790 | A | -0.08 | 0.01 | 0.01 | 0.01 |
| 33 | rs1496653 | G | -0.08 | 0.01 | 0.00 | 0.01 |
| 34 | rs1552224 | C | -0.10 | 0.01 | 0.01 | 0.01 |
| 35 | rs16988333 | G | -0.07 | 0.01 | -0.02 | 0.02 |
| 36 | rs17086692 | T | -0.05 | 0.01 | 0.00 | 0.01 |
| 37 | rs17168486 | T | 0.07 | 0.01 | 0.00 | 0.01 |
| 38 | rs17334919 | T | -0.14 | 0.01 | -0.03 | 0.02 |
| 39 | rs17405722 | A | 0.09 | 0.01 | 0.02 | 0.02 |
| 40 | rs17411031 | G | -0.05 | 0.01 | -0.02 | 0.01 |
| 41 | rs1758632 | G | 0.05 | 0.01 | 0.00 | 0.01 |
| 42 | rs17631783 | T | -0.05 | 0.01 | -0.01 | 0.01 |
| 43 | rs17791483 | G | -0.10 | 0.01 | -0.01 | 0.02 |
| 44 | rs1801214 | T | 0.09 | 0.01 | 0.01 | 0.01 |
| 45 | rs1899951 | T | -0.11 | 0.01 | 0.00 | 0.01 |
| 46 | rs2191348 | T | 0.07 | 0.01 | 0.01 | 0.01 |
| 47 | rs2237892 | T | -0.10 | 0.02 | -0.02 | 0.01 |
| 48 | rs2246618 | T | 0.05 | 0.01 | -0.01 | 0.01 |
| 49 | rs2261181 | T | 0.10 | 0.01 | -0.01 | 0.01 |
| 50 | rs2292662 | T | -0.06 | 0.01 | -0.01 | 0.01 |
| 51 | rs2294120 | G | -0.04 | 0.01 | 0.00 | 0.01 |
| 52 | rs2296173 | G | 0.07 | 0.01 | 0.00 | 0.01 |
| 53 | rs2299383 | T | 0.04 | 0.01 | 0.00 | 0.01 |
| 54 | rs243019 | C | 0.06 | 0.01 | 0.00 | 0.01 |
| 55 | rs2493394 | G | 0.07 | 0.01 | 0.03 | 0.01 |
| 56 | rs2796441 | A | -0.07 | 0.01 | 0.01 | 0.01 |
| 57 | rs2820426 | G | 0.05 | 0.01 | 0.00 | 0.01 |
| 58 | rs2867125 | C | 0.06 | 0.01 | 0.00 | 0.01 |
| 59 | rs2908282 | A | 0.06 | 0.01 | 0.01 | 0.01 |
| 60 | rs2925979 | C | -0.05 | 0.01 | -0.02 | 0.01 |
| 61 | rs2972144 | G | 0.09 | 0.01 | 0.00 | 0.01 |
| 62 | rs340874 | C | 0.06 | 0.01 | 0.01 | 0.01 |
| 63 | rs348330 | A | -0.05 | 0.01 | 0.00 | 0.01 |
| 64 | rs3756784 | G | 0.05 | 0.01 | 0.01 | 0.01 |
| 65 | rs3802177 | A | -0.12 | 0.01 | -0.02 | 0.01 |
| 66 | rs4506565 | T | 0.28 | 0.01 | 0.02 | 0.01 |
| 67 | rs459193 | G | 0.07 | 0.01 | 0.01 | 0.01 |
| 68 | rs4686471 | C | 0.05 | 0.01 | 0.01 | 0.01 |
| 69 | rs4810426 | T | 0.07 | 0.01 | -0.02 | 0.01 |
| 70 | rs4823182 | G | 0.05 | 0.01 | 0.00 | 0.01 |
| 71 | rs4865796 | A | 0.05 | 0.01 | 0.01 | 0.01 |
| 72 | rs516946 | C | 0.08 | 0.01 | 0.00 | 0.01 |
| 73 | rs5215 | T | -0.07 | 0.01 | -0.04 | 0.01 |
| 74 | rs576674 | A | -0.07 | 0.01 | -0.02 | 0.01 |
| 75 | rs6059662 | G | 0.04 | 0.01 | 0.00 | 0.01 |
| 76 | rs6066138 | A | -0.05 | 0.01 | -0.02 | 0.01 |
| 77 | rs61953351 | T | -0.07 | 0.01 | 0.00 | 0.01 |
| 78 | rs622217 | C | -0.05 | 0.01 | 0.01 | 0.01 |
| 79 | rs6494307 | G | -0.04 | 0.01 | -0.01 | 0.01 |
| 80 | rs6515236 | C | -0.05 | 0.01 | 0.00 | 0.01 |
| 81 | rs67232546 | T | 0.06 | 0.01 | 0.00 | 0.01 |
| 82 | rs6795735 | T | -0.06 | 0.01 | -0.03 | 0.01 |
| 83 | rs6878122 | A | -0.06 | 0.01 | -0.02 | 0.01 |
| 84 | rs7138300 | T | -0.04 | 0.01 | -0.01 | 0.01 |
| 85 | rs7144011 | T | 0.05 | 0.01 | 0.02 | 0.01 |
| 86 | rs7177055 | A | 0.06 | 0.01 | 0.01 | 0.01 |
| 87 | rs7185735 | G | 0.11 | 0.01 | 0.01 | 0.01 |
| 88 | rs7240767 | C | 0.05 | 0.01 | 0.00 | 0.01 |
| 89 | rs72892910 | T | 0.06 | 0.01 | 0.02 | 0.01 |
| 90 | rs735949 | C | -0.07 | 0.01 | -0.01 | 0.01 |
| 91 | rs753270 | C | 0.05 | 0.01 | 0.01 | 0.01 |
| 92 | rs7561798 | G | 0.04 | 0.01 | 0.01 | 0.01 |
| 93 | rs7572970 | G | 0.06 | 0.01 | 0.02 | 0.01 |
| 94 | rs7619041 | A | -0.04 | 0.01 | 0.01 | 0.01 |
| 95 | rs7651090 | G | 0.12 | 0.01 | 0.02 | 0.01 |
| 96 | rs7674212 | T | -0.05 | 0.01 | 0.01 | 0.01 |
| 97 | rs7685296 | T | -0.05 | 0.01 | 0.00 | 0.01 |
| 98 | rs77258096 | A | -0.12 | 0.01 | 0.00 | 0.01 |
| 99 | rs7729395 | T | 0.14 | 0.02 | 0.03 | 0.02 |
| 100 | rs7756992 | G | 0.13 | 0.01 | 0.00 | 0.01 |
| 101 | rs7786095 | G | -0.07 | 0.01 | -0.01 | 0.01 |
| 102 | rs780094 | C | 0.07 | 0.01 | -0.01 | 0.01 |
| 103 | rs7845219 | C | -0.04 | 0.01 | 0.00 | 0.01 |
| 104 | rs7923866 | T | -0.10 | 0.01 | 0.01 | 0.01 |
| 105 | rs7929543 | C | 0.08 | 0.01 | 0.01 | 0.01 |
| 106 | rs8068804 | A | 0.06 | 0.01 | 0.02 | 0.01 |
| 107 | rs8108269 | G | 0.06 | 0.01 | 0.02 | 0.01 |
| 108 | rs825476 | T | 0.05 | 0.01 | 0.02 | 0.01 |
| 109 | rs840967 | A | -0.05 | 0.01 | 0.01 | 0.01 |
| 110 | rs849135 | A | -0.10 | 0.01 | 0.00 | 0.01 |
| 111 | rs853974 | C | -0.06 | 0.01 | 0.00 | 0.01 |
| 112 | rs9369425 | A | -0.05 | 0.01 | 0.02 | 0.01 |
| 113 | rs963740 | T | -0.05 | 0.01 | -0.01 | 0.01 |
| 114 | rs982077 | G | -0.05 | 0.01 | -0.02 | 0.01 |
| 115 | rs9844972 | C | 0.10 | 0.01 | 0.00 | 0.02 |
| 116 | rs9894220 | G | -0.06 | 0.01 | 0.00 | 0.01 |
| 117 | rs993380 | G | -0.05 | 0.01 | -0.01 | 0.01 |
| 118 | rs9940149 | A | -0.06 | 0.01 | -0.01 | 0.01 |

T2DM: Type-2 diabetes

Table 18: Genetic association estimates for the effect of T2DM on SBP. ea=effect allele, gx=T2DM, gy=SBP, se=standard error

|  | SNP | ea | gx | gx_se | gy | gy_se |
| --- | --- | --- | --- | --- | --- | --- |
| 1 | rs10077431 | A | -0.05 | 0.01 | -0.07 | 0.04 |
| 2 | rs10100265 | C | -0.05 | 0.01 | -0.36 | 0.03 |
| 3 | rs10114341 | C | -0.04 | 0.01 | 0.01 | 0.03 |
| 4 | rs10401969 | C | 0.09 | 0.01 | 0.24 | 0.06 |
| 5 | rs1050226 | G | -0.05 | 0.01 | -0.06 | 0.03 |
| 6 | rs1061813 | A | -0.04 | 0.01 | 0.04 | 0.03 |
| 7 | rs1063355 | G | 0.07 | 0.01 | 0.20 | 0.03 |
| 8 | rs10740322 | A | 0.05 | 0.01 | 0.01 | 0.03 |
| 9 | rs10811661 | C | -0.16 | 0.01 | -0.06 | 0.04 |
| 10 | rs10830963 | G | 0.09 | 0.01 | 0.13 | 0.03 |
| 11 | rs10842994 | T | -0.08 | 0.01 | -0.08 | 0.04 |
| 12 | rs10974438 | C | 0.06 | 0.01 | -0.04 | 0.03 |
| 13 | rs11098676 | C | 0.05 | 0.01 | 0.12 | 0.04 |
| 14 | rs11107116 | T | 0.05 | 0.01 | -0.02 | 0.04 |
| 15 | rs11257655 | T | 0.07 | 0.01 | 0.16 | 0.04 |
| 16 | rs1127655 | T | -0.04 | 0.01 | -0.02 | 0.03 |
| 17 | rs11708067 | G | -0.10 | 0.01 | 0.15 | 0.04 |
| 18 | rs11925227 | A | -0.05 | 0.01 | 0.02 | 0.04 |
| 19 | rs11926707 | C | 0.05 | 0.01 | 0.09 | 0.03 |
| 20 | rs12088739 | G | -0.09 | 0.01 | -0.08 | 0.05 |
| 21 | rs12299509 | G | 0.05 | 0.01 | -0.06 | 0.03 |
| 22 | rs12617659 | T | -0.07 | 0.01 | -0.13 | 0.04 |
| 23 | rs12910825 | G | 0.05 | 0.01 | -0.08 | 0.03 |
| 24 | rs12945601 | C | -0.05 | 0.01 | -0.02 | 0.03 |
| 25 | rs12970134 | A | 0.06 | 0.01 | -0.17 | 0.03 |
| 26 | rs13234269 | A | -0.06 | 0.01 | -0.24 | 0.03 |
| 27 | rs13239186 | T | 0.05 | 0.01 | 0.12 | 0.03 |
| 28 | rs1333039 | C | 0.05 | 0.01 | 0.05 | 0.03 |
| 29 | rs13330951 | G | -0.05 | 0.01 | 0.02 | 0.03 |
| 30 | rs13389219 | T | -0.07 | 0.01 | -0.23 | 0.03 |
| 31 | rs1359790 | A | -0.08 | 0.01 | 0.15 | 0.03 |
| 32 | rs1496653 | G | -0.08 | 0.01 | 0.01 | 0.04 |
| 33 | rs1552224 | C | -0.10 | 0.01 | 0.08 | 0.04 |
| 34 | rs16988333 | G | -0.07 | 0.01 | -0.08 | 0.05 |
| 35 | rs17086692 | T | -0.05 | 0.01 | -0.12 | 0.03 |
| 36 | rs17168486 | T | 0.07 | 0.01 | 0.12 | 0.04 |
| 37 | rs17334919 | T | -0.14 | 0.01 | 0.03 | 0.05 |
| 38 | rs17405722 | A | 0.09 | 0.01 | -0.04 | 0.06 |
| 39 | rs17411031 | G | -0.05 | 0.01 | -0.07 | 0.03 |
| 40 | rs1758632 | G | 0.05 | 0.01 | -0.08 | 0.03 |
| 41 | rs17631783 | T | -0.05 | 0.01 | 0.02 | 0.03 |
| 42 | rs17791483 | G | -0.10 | 0.01 | -0.15 | 0.06 |
| 43 | rs1801214 | T | 0.09 | 0.01 | -0.08 | 0.03 |
| 44 | rs1899951 | T | -0.11 | 0.01 | -0.15 | 0.05 |
| 45 | rs2191348 | T | 0.07 | 0.01 | 0.02 | 0.03 |
| 46 | rs2237892 | T | -0.10 | 0.02 | -0.20 | 0.06 |
| 47 | rs2246618 | T | 0.05 | 0.01 | -0.06 | 0.03 |
| 48 | rs2261181 | T | 0.10 | 0.01 | 0.21 | 0.05 |
| 49 | rs2292662 | T | -0.06 | 0.01 | -0.04 | 0.04 |
| 50 | rs2294120 | G | -0.04 | 0.01 | -0.10 | 0.03 |
| 51 | rs2296173 | G | 0.07 | 0.01 | 0.06 | 0.04 |
| 52 | rs2299383 | T | 0.04 | 0.01 | 0.08 | 0.03 |
| 53 | rs243019 | C | 0.06 | 0.01 | -0.14 | 0.03 |
| 54 | rs2493394 | G | 0.07 | 0.01 | 0.12 | 0.05 |
| 55 | rs2796441 | A | -0.07 | 0.01 | -0.03 | 0.03 |
| 56 | rs2820426 | G | 0.05 | 0.01 | 0.10 | 0.03 |
| 57 | rs2867125 | C | 0.06 | 0.01 | 0.05 | 0.04 |
| 58 | rs2908282 | A | 0.06 | 0.01 | 0.15 | 0.04 |
| 59 | rs2925979 | C | -0.05 | 0.01 | -0.15 | 0.03 |
| 60 | rs2972144 | G | 0.09 | 0.01 | 0.25 | 0.03 |
| 61 | rs340874 | C | 0.06 | 0.01 | 0.06 | 0.03 |
| 62 | rs348330 | A | -0.05 | 0.01 | -0.08 | 0.03 |
| 63 | rs3756784 | G | 0.05 | 0.01 | -0.04 | 0.04 |
| 64 | rs3802177 | A | -0.12 | 0.01 | -0.04 | 0.03 |
| 65 | rs4506565 | T | 0.28 | 0.01 | 0.22 | 0.03 |
| 66 | rs459193 | G | 0.07 | 0.01 | 0.21 | 0.03 |
| 67 | rs4686471 | C | 0.05 | 0.01 | 0.04 | 0.03 |
| 68 | rs4810426 | T | 0.07 | 0.01 | -0.10 | 0.05 |
| 69 | rs4823182 | G | 0.05 | 0.01 | -0.04 | 0.03 |
| 70 | rs4865796 | A | 0.05 | 0.01 | 0.26 | 0.03 |
| 71 | rs516946 | C | 0.08 | 0.01 | -0.02 | 0.04 |
| 72 | rs5215 | T | -0.07 | 0.01 | -0.35 | 0.03 |
| 73 | rs576674 | A | -0.07 | 0.01 | 0.04 | 0.04 |
| 74 | rs6059662 | G | 0.04 | 0.01 | 0.01 | 0.03 |
| 75 | rs6066138 | A | -0.05 | 0.01 | -0.07 | 0.03 |
| 76 | rs61953351 | T | -0.07 | 0.01 | 0.03 | 0.03 |
| 77 | rs622217 | C | -0.05 | 0.01 | -0.15 | 0.03 |
| 78 | rs6494307 | G | -0.04 | 0.01 | 0.05 | 0.03 |
| 79 | rs6515236 | C | -0.05 | 0.01 | -0.03 | 0.03 |
| 80 | rs67232546 | T | 0.06 | 0.01 | -0.05 | 0.04 |
| 81 | rs6795735 | T | -0.06 | 0.01 | -0.07 | 0.03 |
| 82 | rs7138300 | T | -0.04 | 0.01 | 0.02 | 0.03 |
| 83 | rs7144011 | T | 0.05 | 0.01 | 0.00 | 0.04 |
| 84 | rs7177055 | A | 0.06 | 0.01 | 0.04 | 0.03 |
| 85 | rs7185735 | G | 0.11 | 0.01 | 0.01 | 0.03 |
| 86 | rs7240767 | C | 0.05 | 0.01 | -0.02 | 0.03 |
| 87 | rs72892910 | T | 0.06 | 0.01 | -0.01 | 0.04 |
| 88 | rs735949 | C | -0.07 | 0.01 | -0.01 | 0.04 |
| 89 | rs753270 | C | 0.05 | 0.01 | -0.03 | 0.03 |
| 90 | rs7561798 | G | 0.04 | 0.01 | 0.03 | 0.03 |
| 91 | rs7572970 | G | 0.06 | 0.01 | 0.03 | 0.03 |
| 92 | rs7619041 | A | -0.04 | 0.01 | -0.08 | 0.03 |
| 93 | rs7651090 | G | 0.12 | 0.01 | 0.05 | 0.03 |
| 94 | rs7674212 | T | -0.05 | 0.01 | -0.02 | 0.03 |
| 95 | rs7685296 | T | -0.05 | 0.01 | -0.15 | 0.03 |
| 96 | rs77258096 | A | -0.12 | 0.01 | -0.06 | 0.05 |
| 97 | rs7729395 | T | 0.14 | 0.02 | 0.23 | 0.07 |
| 98 | rs7756992 | G | 0.13 | 0.01 | 0.21 | 0.03 |
| 99 | rs7786095 | G | -0.07 | 0.01 | -0.07 | 0.05 |
| 100 | rs780094 | C | 0.07 | 0.01 | -0.15 | 0.03 |
| 101 | rs7845219 | C | -0.04 | 0.01 | -0.17 | 0.03 |
| 102 | rs7923866 | T | -0.10 | 0.01 | 0.01 | 0.03 |
| 103 | rs7929543 | C | 0.08 | 0.01 | 0.30 | 0.05 |
| 104 | rs8068804 | A | 0.06 | 0.01 | 0.05 | 0.03 |
| 105 | rs8108269 | G | 0.06 | 0.01 | 0.02 | 0.03 |
| 106 | rs825476 | T | 0.05 | 0.01 | 0.08 | 0.03 |
| 107 | rs840967 | A | -0.05 | 0.01 | -0.08 | 0.03 |
| 108 | rs849135 | A | -0.10 | 0.01 | -0.17 | 0.03 |
| 109 | rs853974 | C | -0.06 | 0.01 | 0.24 | 0.03 |
| 110 | rs9369425 | A | -0.05 | 0.01 | 0.04 | 0.03 |
| 111 | rs963740 | T | -0.05 | 0.01 | -0.01 | 0.03 |
| 112 | rs982077 | G | -0.05 | 0.01 | -0.05 | 0.03 |
| 113 | rs9844972 | C | 0.10 | 0.01 | 0.47 | 0.06 |
| 114 | rs9894220 | G | -0.06 | 0.01 | -0.17 | 0.03 |
| 115 | rs993380 | G | -0.05 | 0.01 | 0.01 | 0.03 |
| 116 | rs9940149 | A | -0.06 | 0.01 | -0.03 | 0.04 |

T2DM: Type-2 diabetes; SBP: Systolic blood pressure

Table 19: Genetic association estimates for the effect of T2DM on DBP. ea=effect allele, gx=T2DM, gy=DBP, se=standard error

|  | SNP | ea | gx | gx_se | gy | gy_se |
| --- | --- | --- | --- | --- | --- | --- |
| 1 | rs10077431 | A | -0.05 | 0.02 | -0.05 | 0.01 |
| 2 | rs10100265 | C | -0.05 | 0.02 | -0.16 | 0.01 |
| 3 | rs10114341 | C | -0.04 | 0.02 | 0.02 | 0.01 |
| 4 | rs10401969 | C | 0.09 | 0.03 | 0.24 | 0.01 |
| 5 | rs1050226 | G | -0.05 | 0.02 | -0.04 | 0.01 |
| 6 | rs1061813 | A | -0.04 | 0.02 | 0.03 | 0.01 |
| 7 | rs1063355 | G | 0.07 | 0.02 | 0.02 | 0.01 |
| 8 | rs10740322 | A | 0.05 | 0.02 | 0 | 0.01 |
| 9 | rs10811661 | C | -0.16 | 0.02 | 0 | 0.01 |
| 10 | rs10830963 | G | 0.09 | 0.02 | -0.01 | 0.01 |
| 11 | rs10842994 | T | -0.08 | 0.02 | 0.09 | 0.01 |
| 12 | rs10974438 | C | 0.06 | 0.02 | -0.02 | 0.01 |
| 13 | rs11098676 | C | 0.05 | 0.02 | 0.04 | 0.01 |
| 14 | rs11107116 | T | 0.05 | 0.02 | -0.02 | 0.01 |
| 15 | rs1111875 | T | -0.09 | 0.02 | 0.05 | 0.01 |
| 16 | rs11257655 | T | 0.07 | 0.02 | 0.02 | 0.01 |
| 17 | rs1127655 | T | -0.04 | 0.02 | -0.05 | 0.01 |
| 18 | rs11708067 | G | -0.1 | 0.02 | 0.07 | 0.01 |
| 19 | rs11925227 | A | -0.05 | 0.02 | 0.02 | 0.01 |
| 20 | rs11926707 | C | 0.05 | 0.02 | 0.03 | 0.01 |
| 21 | rs12088739 | G | -0.09 | 0.03 | 0.14 | 0.01 |
| 22 | rs12299509 | G | 0.05 | 0.02 | -0.06 | 0.01 |
| 23 | rs12617659 | T | -0.07 | 0.02 | -0.1 | 0.01 |
| 24 | rs12910825 | G | 0.05 | 0.02 | -0.04 | 0.01 |
| 25 | rs12945601 | C | -0.05 | 0.02 | -0.03 | 0.01 |
| 26 | rs12970134 | A | 0.06 | 0.02 | -0.06 | 0.01 |
| 27 | rs13234269 | A | -0.06 | 0.02 | -0.13 | 0.01 |
| 28 | rs13239186 | T | 0.05 | 0.02 | 0.06 | 0.01 |
| 29 | rs13330951 | G | -0.05 | 0.02 | 0.06 | 0.01 |
| 30 | rs13389219 | T | -0.07 | 0.02 | -0.13 | 0.01 |
| 31 | rs1359790 | A | -0.08 | 0.02 | 0.1 | 0.01 |
| 32 | rs1496653 | G | -0.08 | 0.02 | 0.01 | 0.01 |
| 33 | rs1552224 | C | -0.1 | 0.02 | 0.04 | 0.01 |
| 34 | rs16988333 | G | -0.07 | 0.03 | -0.08 | 0.01 |
| 35 | rs17086692 | T | -0.05 | 0.02 | -0.03 | 0.01 |
| 36 | rs17168486 | T | 0.07 | 0.02 | 0.04 | 0.01 |
| 37 | rs17405722 | A | 0.09 | 0.03 | 0 | 0.01 |
| 38 | rs17411031 | G | -0.05 | 0.02 | -0.03 | 0.01 |
| 39 | rs1758632 | G | 0.05 | 0.02 | -0.03 | 0.01 |
| 40 | rs17631783 | T | -0.05 | 0.02 | 0.05 | 0.01 |
| 41 | rs17791513 | G | -0.1 | 0.04 | -0.03 | 0.01 |
| 42 | rs1801214 | T | 0.09 | 0.02 | -0.02 | 0.01 |
| 43 | rs1899951 | T | -0.11 | 0.03 | 0.01 | 0.01 |
| 44 | rs2058913 | T | -0.05 | 0.02 | -0.03 | 0.01 |
| 45 | rs2237892 | T | -0.1 | 0.04 | -0.09 | 0.02 |
| 46 | rs2246618 | T | 0.05 | 0.02 | -0.11 | 0.01 |
| 47 | rs2261181 | T | 0.1 | 0.03 | 0.1 | 0.01 |
| 48 | rs2294120 | G | -0.04 | 0.02 | -0.04 | 0.01 |
| 49 | rs2296173 | G | 0.07 | 0.02 | 0.07 | 0.01 |
| 50 | rs2299383 | T | 0.04 | 0.02 | 0.01 | 0.01 |
| 51 | rs243019 | C | 0.06 | 0.02 | -0.02 | 0.01 |
| 52 | rs2493394 | G | 0.07 | 0.03 | 0.01 | 0.01 |
| 53 | rs2796441 | A | -0.07 | 0.02 | -0.02 | 0.01 |
| 54 | rs2820426 | G | 0.05 | 0.02 | -0.02 | 0.01 |
| 55 | rs2867125 | C | 0.06 | 0.02 | -0.01 | 0.01 |
| 56 | rs2908282 | A | 0.06 | 0.02 | -0.02 | 0.01 |
| 57 | rs2925979 | C | -0.05 | 0.02 | -0.1 | 0.01 |
| 58 | rs2943656 | G | 0.09 | 0.02 | 0.13 | 0.01 |
| 59 | rs3217992 | T | 0.05 | 0.02 | -0.1 | 0.01 |
| 60 | rs340874 | C | 0.06 | 0.02 | 0 | 0.01 |
| 61 | rs348330 | A | -0.05 | 0.02 | -0.06 | 0.01 |
| 62 | rs3756784 | G | 0.05 | 0.02 | -0.07 | 0.01 |
| 63 | rs3802177 | A | -0.12 | 0.02 | 0 | 0.01 |
| 64 | rs459193 | G | 0.07 | 0.02 | 0.07 | 0.01 |
| 65 | rs4622883 | G | -0.04 | 0.02 | -0.02 | 0.01 |
| 66 | rs4686471 | C | 0.05 | 0.02 | -0.02 | 0.01 |
| 67 | rs4812829 | A | 0.05 | 0.02 | -0.02 | 0.01 |
| 68 | rs4823182 | G | 0.05 | 0.02 | 0.02 | 0.01 |
| 69 | rs4865796 | A | 0.05 | 0.02 | 0.13 | 0.01 |
| 70 | rs516946 | C | 0.08 | 0.02 | 0 | 0.01 |
| 71 | rs5215 | T | -0.07 | 0.02 | -0.13 | 0.01 |
| 72 | rs55966194 | G | -0.05 | 0.02 | -0.05 | 0.01 |
| 73 | rs576674 | A | -0.07 | 0.02 | 0.05 | 0.01 |
| 74 | rs6059662 | G | 0.04 | 0.02 | -0.03 | 0.01 |
| 75 | rs61953351 | T | -0.07 | 0.02 | 0.01 | 0.01 |
| 76 | rs622217 | C | -0.05 | 0.02 | -0.02 | 0.01 |
| 77 | rs6494307 | G | -0.04 | 0.02 | 0 | 0.01 |
| 78 | rs6515236 | C | -0.05 | 0.02 | 0.01 | 0.01 |
| 79 | rs67232546 | T | 0.06 | 0.02 | 0 | 0.01 |
| 80 | rs6767484 | G | 0.12 | 0.02 | 0.01 | 0.01 |
| 81 | rs6785040 | C | -0.06 | 0.03 | 0.04 | 0.01 |
| 82 | rs6795735 | T | -0.06 | 0.02 | -0.14 | 0.01 |
| 83 | rs6960043 | C | 0.06 | 0.02 | 0 | 0.01 |
| 84 | rs7144011 | T | 0.05 | 0.02 | -0.01 | 0.01 |
| 85 | rs7177055 | A | 0.06 | 0.02 | 0.06 | 0.01 |
| 86 | rs7240767 | C | 0.05 | 0.02 | -0.01 | 0.01 |
| 87 | rs72802358 | C | -0.12 | 0.03 | -0.03 | 0.01 |
| 88 | rs72892910 | T | 0.06 | 0.02 | -0.06 | 0.01 |
| 89 | rs735949 | C | -0.07 | 0.03 | -0.01 | 0.01 |
| 90 | rs753270 | C | 0.05 | 0.02 | -0.01 | 0.01 |
| 91 | rs7561798 | G | 0.04 | 0.02 | 0.01 | 0.01 |
| 92 | rs7572970 | G | 0.06 | 0.02 | 0.01 | 0.01 |
| 93 | rs7607777 | T | -0.14 | 0.03 | -0.04 | 0.01 |
| 94 | rs7674212 | T | -0.05 | 0.02 | -0.13 | 0.01 |
| 95 | rs7685296 | T | -0.05 | 0.02 | -0.03 | 0.01 |
| 96 | rs7729395 | T | 0.14 | 0.04 | 0.16 | 0.02 |
| 97 | rs7756992 | G | 0.13 | 0.02 | 0.11 | 0.01 |
| 98 | rs7786095 | G | -0.07 | 0.03 | 0 | 0.01 |
| 99 | rs780094 | C | 0.07 | 0.02 | -0.07 | 0.01 |
| 100 | rs7845219 | C | -0.04 | 0.02 | -0.09 | 0.01 |
| 101 | rs7903146 | T | 0.31 | 0.02 | 0.01 | 0.01 |
| 102 | rs7929543 | C | 0.08 | 0.03 | 0.12 | 0.01 |
| 103 | rs7955901 | T | -0.04 | 0.02 | 0.04 | 0.01 |
| 104 | rs8068804 | A | 0.06 | 0.02 | 0.11 | 0.01 |
| 105 | rs8108269 | G | 0.06 | 0.02 | -0.01 | 0.01 |
| 106 | rs825476 | T | 0.05 | 0.02 | 0.05 | 0.01 |
| 107 | rs840967 | A | -0.05 | 0.02 | -0.06 | 0.01 |
| 108 | rs849135 | A | -0.1 | 0.02 | -0.08 | 0.01 |
| 109 | rs853974 | C | -0.06 | 0.02 | 0.1 | 0.01 |
| 110 | rs9369425 | A | -0.05 | 0.02 | 0.14 | 0.01 |
| 111 | rs963740 | T | -0.05 | 0.02 | -0.03 | 0.01 |
| 112 | rs9844972 | C | 0.1 | 0.03 | 0.23 | 0.01 |
| 113 | rs9894220 | G | -0.06 | 0.02 | -0.1 | 0.01 |
| 114 | rs9928094 | G | 0.1 | 0.02 | -0.06 | 0.01 |
| 115 | rs993380 | G | -0.05 | 0.02 | 0 | 0.01 |
| 116 | rs9940149 | A | -0.06 | 0.02 | -0.05 | 0.01 |

T2DM: Type-2 diabetes; DBP: Diastolic blood pressure

Table 20: Genetic association estimates for the effect of T2DM on TG. ea=effect allele, gx=T2DM, gy=TG, se=standard error

|  | SNP | ea | gx | gx_se | gy | gy_se |
| --- | --- | --- | --- | --- | --- | --- |
| 1 | rs10077431 | A | -0.05 | 0.01 | -0.01 | 0.00 |
| 2 | rs10087241 | A | -0.05 | 0.01 | -0.01 | 0.00 |
| 3 | rs10100265 | C | -0.05 | 0.01 | 0.02 | 0.00 |
| 4 | rs10114341 | C | -0.04 | 0.01 | -0.01 | 0.00 |
| 5 | rs10401969 | C | 0.09 | 0.01 | -0.10 | 0.00 |
| 6 | rs1050226 | G | -0.05 | 0.01 | 0.00 | 0.00 |
| 7 | rs1061813 | A | -0.04 | 0.01 | 0.00 | 0.00 |
| 8 | rs1063355 | G | 0.07 | 0.01 | -0.01 | 0.00 |
| 9 | rs10740322 | A | 0.05 | 0.01 | 0.00 | 0.00 |
| 10 | rs10811661 | C | -0.16 | 0.01 | -0.01 | 0.00 |
| 11 | rs10830963 | G | 0.09 | 0.01 | 0.01 | 0.00 |
| 12 | rs10842994 | T | -0.08 | 0.01 | 0.00 | 0.00 |
| 13 | rs10974438 | C | 0.06 | 0.01 | -0.01 | 0.00 |
| 14 | rs11098676 | C | 0.05 | 0.01 | 0.00 | 0.00 |
| 15 | rs11107116 | T | 0.05 | 0.01 | 0.01 | 0.00 |
| 16 | rs11257655 | T | 0.07 | 0.01 | 0.01 | 0.00 |
| 17 | rs1127655 | T | -0.04 | 0.01 | 0.00 | 0.00 |
| 18 | rs11708067 | G | -0.10 | 0.01 | 0.00 | 0.00 |
| 19 | rs11925227 | A | -0.05 | 0.01 | 0.00 | 0.00 |
| 20 | rs11926707 | C | 0.05 | 0.01 | 0.01 | 0.00 |
| 21 | rs12088739 | G | -0.09 | 0.01 | -0.03 | 0.00 |
| 22 | rs12299509 | G | 0.05 | 0.01 | 0.00 | 0.00 |
| 23 | rs12617659 | T | -0.07 | 0.01 | -0.01 | 0.00 |
| 24 | rs12910825 | G | 0.05 | 0.01 | 0.00 | 0.00 |
| 25 | rs12945601 | C | -0.05 | 0.01 | -0.01 | 0.00 |
| 26 | rs12970134 | A | 0.06 | 0.01 | 0.02 | 0.00 |
| 27 | rs13234269 | A | -0.06 | 0.01 | -0.03 | 0.00 |
| 28 | rs13239186 | T | 0.05 | 0.01 | 0.00 | 0.00 |
| 29 | rs1333039 | C | 0.05 | 0.01 | 0.00 | 0.00 |
| 30 | rs13330951 | G | -0.05 | 0.01 | -0.02 | 0.00 |
| 31 | rs13389219 | T | -0.07 | 0.01 | -0.04 | 0.00 |
| 32 | rs1359790 | A | -0.08 | 0.01 | 0.00 | 0.00 |
| 33 | rs1496653 | G | -0.08 | 0.01 | 0.00 | 0.00 |
| 34 | rs1552224 | C | -0.10 | 0.01 | 0.00 | 0.00 |
| 35 | rs16988333 | G | -0.07 | 0.01 | 0.00 | 0.00 |
| 36 | rs17086692 | T | -0.05 | 0.01 | 0.00 | 0.00 |
| 37 | rs17168486 | T | 0.07 | 0.01 | 0.00 | 0.00 |
| 38 | rs17334919 | T | -0.14 | 0.01 | -0.01 | 0.00 |
| 39 | rs17405722 | A | 0.09 | 0.01 | 0.00 | 0.00 |
| 40 | rs17411031 | G | -0.05 | 0.01 | -0.12 | 0.00 |
| 41 | rs1758632 | G | 0.05 | 0.01 | 0.01 | 0.00 |
| 42 | rs17631783 | T | -0.05 | 0.01 | 0.00 | 0.00 |
| 43 | rs17791483 | G | -0.10 | 0.01 | 0.00 | 0.00 |
| 44 | rs1801214 | T | 0.09 | 0.01 | 0.00 | 0.00 |
| 45 | rs1899951 | T | -0.11 | 0.01 | -0.03 | 0.00 |
| 46 | rs2191348 | T | 0.07 | 0.01 | 0.00 | 0.00 |
| 47 | rs2237892 | T | -0.10 | 0.02 | 0.00 | 0.00 |
| 48 | rs2246618 | T | 0.05 | 0.01 | -0.01 | 0.00 |
| 49 | rs2261181 | T | 0.10 | 0.01 | 0.01 | 0.00 |
| 50 | rs2292662 | T | -0.06 | 0.01 | -0.01 | 0.00 |
| 51 | rs2294120 | G | -0.04 | 0.01 | -0.01 | 0.00 |
| 52 | rs2296173 | G | 0.07 | 0.01 | 0.02 | 0.00 |
| 53 | rs2299383 | T | 0.04 | 0.01 | 0.00 | 0.00 |
| 54 | rs243019 | C | 0.06 | 0.01 | 0.00 | 0.00 |
| 55 | rs2493394 | G | 0.07 | 0.01 | 0.00 | 0.00 |
| 56 | rs2796441 | A | -0.07 | 0.01 | 0.01 | 0.00 |
| 57 | rs2820426 | G | 0.05 | 0.01 | 0.02 | 0.00 |
| 58 | rs2867125 | C | 0.06 | 0.01 | 0.00 | 0.00 |
| 59 | rs2908282 | A | 0.06 | 0.01 | 0.01 | 0.00 |
| 60 | rs2925979 | C | -0.05 | 0.01 | -0.03 | 0.00 |
| 61 | rs2972144 | G | 0.09 | 0.01 | 0.04 | 0.00 |
| 62 | rs340874 | C | 0.06 | 0.01 | 0.01 | 0.00 |
| 63 | rs348330 | A | -0.05 | 0.01 | 0.00 | 0.00 |
| 64 | rs3756784 | G | 0.05 | 0.01 | 0.01 | 0.00 |
| 65 | rs3802177 | A | -0.12 | 0.01 | -0.02 | 0.00 |
| 66 | rs4506565 | T | 0.28 | 0.01 | 0.00 | 0.00 |
| 67 | rs459193 | G | 0.07 | 0.01 | 0.04 | 0.00 |
| 68 | rs4686471 | C | 0.05 | 0.01 | 0.00 | 0.00 |
| 69 | rs4810426 | T | 0.07 | 0.01 | 0.00 | 0.00 |
| 70 | rs4823182 | G | 0.05 | 0.01 | 0.00 | 0.00 |
| 71 | rs4865796 | A | 0.05 | 0.01 | 0.01 | 0.00 |
| 72 | rs516946 | C | 0.08 | 0.01 | 0.00 | 0.00 |
| 73 | rs5215 | T | -0.07 | 0.01 | 0.00 | 0.00 |
| 74 | rs576674 | A | -0.07 | 0.01 | 0.00 | 0.00 |
| 75 | rs6059662 | G | 0.04 | 0.01 | 0.01 | 0.00 |
| 76 | rs6066138 | A | -0.05 | 0.01 | -0.02 | 0.00 |
| 77 | rs61953351 | T | -0.07 | 0.01 | 0.00 | 0.00 |
| 78 | rs622217 | C | -0.05 | 0.01 | -0.01 | 0.00 |
| 79 | rs6494307 | G | -0.04 | 0.01 | 0.00 | 0.00 |
| 80 | rs6515236 | C | -0.05 | 0.01 | 0.00 | 0.00 |
| 81 | rs67232546 | T | 0.06 | 0.01 | 0.00 | 0.00 |
| 82 | rs6795735 | T | -0.06 | 0.01 | 0.00 | 0.00 |
| 83 | rs6878122 | A | -0.06 | 0.01 | 0.00 | 0.00 |
| 84 | rs7138300 | T | -0.04 | 0.01 | -0.01 | 0.00 |
| 85 | rs7144011 | T | 0.05 | 0.01 | 0.01 | 0.00 |
| 86 | rs7177055 | A | 0.06 | 0.01 | 0.01 | 0.00 |
| 87 | rs7185735 | G | 0.11 | 0.01 | 0.00 | 0.00 |
| 88 | rs7240767 | C | 0.05 | 0.01 | 0.00 | 0.00 |
| 89 | rs72892910 | T | 0.06 | 0.01 | 0.00 | 0.00 |
| 90 | rs735949 | C | -0.07 | 0.01 | 0.00 | 0.00 |
| 91 | rs753270 | C | 0.05 | 0.01 | 0.01 | 0.00 |
| 92 | rs7561798 | G | 0.04 | 0.01 | 0.01 | 0.00 |
| 93 | rs7572970 | G | 0.06 | 0.01 | 0.01 | 0.00 |
| 94 | rs7619041 | A | -0.04 | 0.01 | 0.00 | 0.00 |
| 95 | rs7651090 | G | 0.12 | 0.01 | 0.01 | 0.00 |
| 96 | rs7674212 | T | -0.05 | 0.01 | -0.01 | 0.00 |
| 97 | rs7685296 | T | -0.05 | 0.01 | 0.00 | 0.00 |
| 98 | rs77258096 | A | -0.12 | 0.01 | 0.00 | 0.00 |
| 99 | rs7729395 | T | 0.14 | 0.02 | 0.01 | 0.01 |
| 100 | rs7756992 | G | 0.13 | 0.01 | 0.01 | 0.00 |
| 101 | rs7786095 | G | -0.07 | 0.01 | -0.01 | 0.00 |
| 102 | rs780094 | C | 0.07 | 0.01 | -0.10 | 0.00 |
| 103 | rs7845219 | C | -0.04 | 0.01 | -0.01 | 0.00 |
| 104 | rs7923866 | T | -0.10 | 0.01 | -0.01 | 0.00 |
| 105 | rs7929543 | C | 0.08 | 0.01 | 0.02 | 0.00 |
| 106 | rs8068804 | A | 0.06 | 0.01 | 0.00 | 0.00 |
| 107 | rs8108269 | G | 0.06 | 0.01 | 0.01 | 0.00 |
| 108 | rs825476 | T | 0.05 | 0.01 | 0.02 | 0.00 |
| 109 | rs840967 | A | -0.05 | 0.01 | -0.01 | 0.00 |
| 110 | rs849135 | A | -0.10 | 0.01 | -0.01 | 0.00 |
| 111 | rs853974 | C | -0.06 | 0.01 | 0.00 | 0.00 |
| 112 | rs9369425 | A | -0.05 | 0.01 | 0.01 | 0.00 |
| 113 | rs963740 | T | -0.05 | 0.01 | 0.01 | 0.00 |
| 114 | rs982077 | G | -0.05 | 0.01 | -0.01 | 0.00 |
| 115 | rs9844972 | C | 0.10 | 0.01 | 0.04 | 0.00 |
| 116 | rs9894220 | G | -0.06 | 0.01 | -0.01 | 0.00 |
| 117 | rs993380 | G | -0.05 | 0.01 | 0.00 | 0.00 |
| 118 | rs9940149 | A | -0.06 | 0.01 | 0.00 | 0.00 |

T2DM: Type-2 diabetes; TG: Triglycerides

Table 21: Genetic association estimates for the effect of T2DM on LDL. ea=effect allele, gx=T2DM, gy=LDL, se=standard error

|  | SNP | ea | gx | gx_se | gy | gy_se |
| --- | --- | --- | --- | --- | --- | --- |
| 1 | rs10077431 | A | -0.05 | 0.01 | -0.01 | 0.02 |
| 2 | rs10100265 | C | -0.05 | 0.01 | -0.03 | 0.02 |
| 3 | rs10114341 | C | -0.04 | 0.01 | -0.01 | 0.01 |
| 4 | rs10401969 | C | 0.09 | 0.01 | -0.12 | 0.03 |
| 5 | rs1050226 | G | -0.05 | 0.01 | -0.01 | 0.01 |
| 6 | rs1061813 | A | -0.04 | 0.01 | 0.02 | 0.01 |
| 7 | rs1063355 | G | 0.07 | 0.01 | -0.01 | 0.02 |
| 8 | rs10740322 | A | 0.05 | 0.01 | -0.04 | 0.02 |
| 9 | rs10811661 | C | -0.16 | 0.01 | 0.05 | 0.02 |
| 10 | rs10830963 | G | 0.09 | 0.01 | -0.01 | 0.02 |
| 11 | rs10842994 | T | -0.08 | 0.01 | 0.01 | 0.02 |
| 12 | rs10974438 | C | 0.06 | 0.01 | -0.01 | 0.02 |
| 13 | rs11107116 | T | 0.05 | 0.01 | 0.03 | 0.02 |
| 14 | rs1111875 | T | -0.09 | 0.01 | -0.01 | 0.01 |
| 15 | rs11257655 | T | 0.07 | 0.01 | 0.01 | 0.02 |
| 16 | rs1127655 | T | -0.04 | 0.01 | 0.02 | 0.01 |
| 17 | rs11708067 | G | -0.10 | 0.01 | -0.02 | 0.02 |
| 18 | rs12088739 | G | -0.09 | 0.01 | 0.01 | 0.03 |
| 19 | rs12617659 | T | -0.07 | 0.01 | 0.01 | 0.02 |
| 20 | rs12910825 | G | 0.05 | 0.01 | 0.03 | 0.02 |
| 21 | rs12945601 | C | -0.05 | 0.01 | -0.01 | 0.02 |
| 22 | rs12970134 | A | 0.06 | 0.01 | 0.02 | 0.02 |
| 23 | rs13234269 | A | -0.06 | 0.01 | 0.03 | 0.01 |
| 24 | rs13239186 | T | 0.05 | 0.01 | -0.02 | 0.02 |
| 25 | rs13389219 | T | -0.07 | 0.01 | 0.02 | 0.02 |
| 26 | rs1359790 | A | -0.08 | 0.01 | 0.02 | 0.02 |
| 27 | rs1496653 | G | -0.08 | 0.01 | -0.01 | 0.02 |
| 28 | rs1552224 | C | -0.10 | 0.01 | -0.02 | 0.02 |
| 29 | rs16988333 | G | -0.07 | 0.01 | 0.01 | 0.02 |
| 30 | rs17086692 | T | -0.05 | 0.01 | 0.02 | 0.02 |
| 31 | rs17168486 | T | 0.07 | 0.01 | -0.01 | 0.02 |
| 32 | rs17405722 | A | 0.09 | 0.01 | 0.02 | 0.03 |
| 33 | rs17411031 | G | -0.05 | 0.01 | -0.01 | 0.02 |
| 34 | rs1758632 | G | 0.05 | 0.01 | -0.02 | 0.01 |
| 35 | rs17631783 | T | -0.05 | 0.01 | 0.01 | 0.02 |
| 36 | rs17791513 | G | -0.10 | 0.01 | -0.05 | 0.03 |
| 37 | rs1801214 | T | 0.09 | 0.01 | -0.01 | 0.01 |
| 38 | rs1899951 | T | -0.11 | 0.01 | -0.01 | 0.02 |
| 39 | rs2058913 | T | -0.05 | 0.01 | -0.02 | 0.01 |
| 40 | rs2237892 | T | -0.10 | 0.02 | 0.04 | 0.03 |
| 41 | rs2246618 | T | 0.05 | 0.01 | -0.01 | 0.02 |
| 42 | rs2261181 | T | 0.10 | 0.01 | -0.02 | 0.02 |
| 43 | rs2294120 | G | -0.04 | 0.01 | -0.01 | 0.01 |
| 44 | rs2296173 | G | 0.07 | 0.01 | -0.04 | 0.02 |
| 45 | rs2299383 | T | 0.04 | 0.01 | 0.01 | 0.01 |
| 46 | rs243019 | C | 0.06 | 0.01 | -0.01 | 0.01 |
| 47 | rs2493394 | G | 0.07 | 0.01 | 0.01 | 0.02 |
| 48 | rs2796441 | A | -0.07 | 0.01 | 0.02 | 0.01 |
| 49 | rs2820426 | G | 0.05 | 0.01 | 0.01 | 0.02 |
| 50 | rs2867125 | C | 0.06 | 0.01 | 0.01 | 0.02 |
| 51 | rs2908282 | A | 0.06 | 0.01 | -0.01 | 0.02 |
| 52 | rs2925979 | C | -0.05 | 0.01 | -0.01 | 0.02 |
| 53 | rs2943656 | G | 0.09 | 0.01 | -0.02 | 0.02 |
| 54 | rs3217992 | T | 0.05 | 0.01 | 0.01 | 0.02 |
| 55 | rs340874 | C | 0.06 | 0.01 | -0.01 | 0.01 |
| 56 | rs3756784 | G | 0.05 | 0.01 | -0.01 | 0.02 |
| 57 | rs3802177 | A | -0.12 | 0.01 | 0.01 | 0.02 |
| 58 | rs459193 | G | 0.07 | 0.01 | 0.02 | 0.02 |
| 59 | rs4622883 | G | -0.04 | 0.01 | -0.01 | 0.01 |
| 60 | rs4686471 | C | 0.05 | 0.01 | -0.01 | 0.01 |
| 61 | rs4812829 | A | 0.05 | 0.01 | 0.01 | 0.02 |
| 62 | rs4865796 | A | 0.05 | 0.01 | 0.01 | 0.02 |
| 63 | rs516946 | C | 0.08 | 0.01 | -0.01 | 0.02 |
| 64 | rs5215 | T | -0.07 | 0.01 | -0.01 | 0.02 |
| 65 | rs55966194 | G | -0.05 | 0.01 | 0.01 | 0.02 |
| 66 | rs576674 | A | -0.07 | 0.01 | -0.01 | 0.02 |
| 67 | rs6059662 | G | 0.04 | 0.01 | -0.02 | 0.02 |
| 68 | rs61953351 | T | -0.07 | 0.01 | -0.02 | 0.02 |
| 69 | rs6494307 | G | -0.04 | 0.01 | 0.02 | 0.01 |
| 70 | rs67232546 | T | 0.06 | 0.01 | 0.02 | 0.02 |
| 71 | rs6767484 | G | 0.12 | 0.01 | 0.02 | 0.02 |
| 72 | rs6785040 | C | -0.06 | 0.01 | 0.01 | 0.02 |
| 73 | rs6795735 | T | -0.06 | 0.01 | 0.01 | 0.01 |
| 74 | rs6960043 | C | 0.06 | 0.01 | -0.01 | 0.01 |
| 75 | rs7144011 | T | 0.05 | 0.01 | -0.01 | 0.02 |
| 76 | rs7177055 | A | 0.06 | 0.01 | 0.02 | 0.02 |
| 77 | rs7240767 | C | 0.05 | 0.01 | -0.01 | 0.01 |
| 78 | rs72892910 | T | 0.06 | 0.01 | 0.04 | 0.02 |
| 79 | rs735949 | C | -0.07 | 0.01 | 0.03 | 0.02 |
| 80 | rs7572970 | G | 0.06 | 0.01 | 0.01 | 0.02 |
| 81 | rs7607777 | T | -0.14 | 0.01 | 0.01 | 0.02 |
| 82 | rs7674212 | T | -0.05 | 0.01 | -0.01 | 0.02 |
| 83 | rs7685296 | T | -0.05 | 0.01 | 0.03 | 0.02 |
| 84 | rs7729395 | T | 0.14 | 0.02 | 0.01 | 0.03 |
| 85 | rs7756992 | G | 0.13 | 0.01 | 0.01 | 0.02 |
| 86 | rs7786095 | G | -0.07 | 0.01 | 0.07 | 0.02 |
| 87 | rs780094 | C | 0.07 | 0.01 | -0.01 | 0.01 |
| 88 | rs7845219 | C | -0.04 | 0.01 | -0.01 | 0.01 |
| 89 | rs7903146 | T | 0.31 | 0.01 | -0.03 | 0.02 |
| 90 | rs7929543 | C | 0.08 | 0.01 | -0.05 | 0.03 |
| 91 | rs7955901 | T | -0.04 | 0.01 | 0.01 | 0.01 |
| 92 | rs8068804 | A | 0.06 | 0.01 | 0.01 | 0.02 |
| 93 | rs8108269 | G | 0.06 | 0.01 | 0.02 | 0.02 |
| 94 | rs825476 | T | 0.05 | 0.01 | -0.01 | 0.01 |
| 95 | rs840967 | A | -0.05 | 0.01 | -0.01 | 0.01 |
| 96 | rs849135 | A | -0.10 | 0.01 | -0.01 | 0.01 |
| 97 | rs853974 | C | -0.06 | 0.01 | 0.02 | 0.02 |
| 98 | rs9369425 | A | -0.05 | 0.01 | -0.01 | 0.02 |
| 99 | rs963740 | T | -0.05 | 0.01 | 0.01 | 0.02 |
| 100 | rs9844972 | C | 0.10 | 0.01 | -0.01 | 0.03 |
| 101 | rs9894220 | G | -0.06 | 0.01 | 0.01 | 0.01 |
| 102 | rs9928094 | G | 0.10 | 0.01 | -0.01 | 0.02 |
| 103 | rs9940149 | A | -0.06 | 0.01 | -0.02 | 0.02 |

T2DM: Type-2 diabetes; LDL: Low-density lipoprotein

Table 22: Genetic association estimates for the effect of T2DM on HDL. ea=effect allele, gx=T2DM, gy=HDL, se=standard error

|  | SNP | ea | gx | gx_se | gy | gy_se |
| --- | --- | --- | --- | --- | --- | --- |
| 1 | rs10077431 | A | -0.05 | 0.01 | 0.01 | 0.02 |
| 2 | rs10100265 | C | -0.05 | 0.01 | 0.01 | 0.02 |
| 3 | rs10114341 | C | -0.04 | 0.01 | 0.02 | 0.01 |
| 4 | rs10401969 | C | 0.09 | 0.01 | 0.04 | 0.03 |
| 5 | rs1050226 | G | -0.05 | 0.01 | 0.04 | 0.02 |
| 6 | rs1061813 | A | -0.04 | 0.01 | 0.02 | 0.01 |
| 7 | rs1063355 | G | 0.07 | 0.01 | 0.01 | 0.02 |
| 8 | rs10740322 | A | 0.05 | 0.01 | 0.01 | 0.02 |
| 9 | rs10811661 | C | -0.16 | 0.01 | 0.02 | 0.02 |
| 10 | rs10974438 | C | 0.06 | 0.01 | -0.01 | 0.02 |
| 11 | rs11098676 | C | 0.05 | 0.01 | -0.03 | 0.02 |
| 12 | rs11107116 | T | 0.05 | 0.01 | -0.02 | 0.02 |
| 13 | rs11257655 | T | 0.07 | 0.01 | 0.01 | 0.02 |
| 14 | rs1127655 | T | -0.04 | 0.01 | 0.02 | 0.02 |
| 15 | rs11708067 | G | -0.10 | 0.01 | -0.01 | 0.02 |
| 16 | rs11925227 | A | -0.05 | 0.01 | 0.01 | 0.02 |
| 17 | rs12088739 | G | -0.09 | 0.01 | 0.08 | 0.03 |
| 18 | rs12299509 | G | 0.05 | 0.01 | 0.03 | 0.02 |
| 19 | rs12617659 | T | -0.07 | 0.01 | 0.01 | 0.02 |
| 20 | rs12910825 | G | 0.05 | 0.01 | 0.01 | 0.02 |
| 21 | rs12945601 | C | -0.05 | 0.01 | 0.02 | 0.02 |
| 22 | rs12970134 | A | 0.06 | 0.01 | -0.02 | 0.02 |
| 23 | rs13234269 | A | -0.06 | 0.01 | 0.03 | 0.01 |
| 24 | rs13330951 | G | -0.05 | 0.01 | 0.01 | 0.02 |
| 25 | rs13389219 | T | -0.07 | 0.01 | 0.02 | 0.02 |
| 26 | rs1496653 | G | -0.08 | 0.01 | 0.02 | 0.02 |
| 27 | rs1552224 | C | -0.10 | 0.01 | 0.01 | 0.02 |
| 28 | rs16988333 | G | -0.07 | 0.01 | 0.01 | 0.03 |
| 29 | rs17086692 | T | -0.05 | 0.01 | 0.11 | 0.06 |
| 30 | rs17168486 | T | 0.07 | 0.01 | -0.03 | 0.02 |
| 31 | rs17405722 | A | 0.09 | 0.01 | -0.03 | 0.03 |
| 32 | rs17411031 | G | -0.05 | 0.01 | 0.10 | 0.02 |
| 33 | rs1758632 | G | 0.05 | 0.01 | -0.04 | 0.02 |
| 34 | rs17791513 | G | -0.10 | 0.01 | 0.01 | 0.03 |
| 35 | rs1801214 | T | 0.09 | 0.01 | -0.01 | 0.02 |
| 36 | rs1899951 | T | -0.11 | 0.01 | 0.03 | 0.02 |
| 37 | rs2058913 | T | -0.05 | 0.01 | 0.01 | 0.02 |
| 38 | rs2237892 | T | -0.10 | 0.02 | 0.05 | 0.03 |
| 39 | rs2246618 | T | 0.05 | 0.01 | -0.01 | 0.02 |
| 40 | rs2261181 | T | 0.10 | 0.01 | 0.01 | 0.02 |
| 41 | rs2294120 | G | -0.04 | 0.01 | -0.01 | 0.02 |
| 42 | rs2296173 | G | 0.07 | 0.01 | -0.03 | 0.02 |
| 43 | rs2299383 | T | 0.04 | 0.01 | -0.02 | 0.01 |
| 44 | rs243019 | C | 0.06 | 0.01 | 0.01 | 0.02 |
| 45 | rs2493394 | G | 0.07 | 0.01 | -0.03 | 0.02 |
| 46 | rs2820426 | G | 0.05 | 0.01 | -0.03 | 0.02 |
| 47 | rs2867125 | C | 0.06 | 0.01 | -0.02 | 0.02 |
| 48 | rs2908282 | A | 0.06 | 0.01 | 0.04 | 0.02 |
| 49 | rs2925979 | C | -0.05 | 0.01 | 0.04 | 0.02 |
| 50 | rs2943656 | G | 0.09 | 0.01 | -0.03 | 0.02 |
| 51 | rs3217992 | T | 0.05 | 0.01 | -0.01 | 0.02 |
| 52 | rs3756784 | G | 0.05 | 0.01 | -0.03 | 0.02 |
| 53 | rs3802177 | A | -0.12 | 0.01 | 0.01 | 0.02 |
| 54 | rs459193 | G | 0.07 | 0.01 | -0.02 | 0.02 |
| 55 | rs4622883 | G | -0.04 | 0.01 | 0.02 | 0.01 |
| 56 | rs4686471 | C | 0.05 | 0.01 | -0.01 | 0.02 |
| 57 | rs4812829 | A | 0.05 | 0.01 | 0.01 | 0.02 |
| 58 | rs4823182 | G | 0.05 | 0.01 | -0.03 | 0.02 |
| 59 | rs4865796 | A | 0.05 | 0.01 | -0.01 | 0.02 |
| 60 | rs516946 | C | 0.08 | 0.01 | -0.02 | 0.02 |
| 61 | rs5215 | T | -0.07 | 0.01 | -0.01 | 0.02 |
| 62 | rs55966194 | G | -0.05 | 0.01 | -0.01 | 0.02 |
| 63 | rs576674 | A | -0.07 | 0.01 | -0.02 | 0.02 |
| 64 | rs6059662 | G | 0.04 | 0.01 | 0.01 | 0.02 |
| 65 | rs61953351 | T | -0.07 | 0.01 | -0.01 | 0.02 |
| 66 | rs622217 | C | -0.05 | 0.01 | 0.01 | 0.02 |
| 67 | rs6494307 | G | -0.04 | 0.01 | -0.01 | 0.02 |
| 68 | rs6515236 | C | -0.05 | 0.01 | -0.01 | 0.02 |
| 69 | rs67232546 | T | 0.06 | 0.01 | 0.02 | 0.02 |
| 70 | rs6767484 | G | 0.12 | 0.01 | -0.04 | 0.02 |
| 71 | rs6785040 | C | -0.06 | 0.01 | 0.01 | 0.02 |
| 72 | rs6878122 | A | -0.06 | 0.01 | -0.01 | 0.02 |
| 73 | rs6960043 | C | 0.06 | 0.01 | -0.01 | 0.02 |
| 74 | rs7240767 | C | 0.05 | 0.01 | -0.01 | 0.01 |
| 75 | rs72802358 | C | -0.12 | 0.01 | 0.01 | 0.02 |
| 76 | rs72892910 | T | 0.06 | 0.01 | 0.02 | 0.02 |
| 77 | rs735949 | C | -0.07 | 0.01 | -0.01 | 0.02 |
| 78 | rs753270 | C | 0.05 | 0.01 | -0.01 | 0.02 |
| 79 | rs7561798 | G | 0.04 | 0.01 | 0.02 | 0.01 |
| 80 | rs7572970 | G | 0.06 | 0.01 | -0.01 | 0.02 |
| 81 | rs7607777 | T | -0.14 | 0.01 | -0.03 | 0.02 |
| 82 | rs7674212 | T | -0.05 | 0.01 | 0.01 | 0.02 |
| 83 | rs7685296 | T | -0.05 | 0.01 | 0.03 | 0.02 |
| 84 | rs7729395 | T | 0.14 | 0.02 | -0.06 | 0.03 |
| 85 | rs7756992 | G | 0.13 | 0.01 | -0.01 | 0.02 |
| 86 | rs7786095 | G | -0.07 | 0.01 | -0.01 | 0.02 |
| 87 | rs780094 | C | 0.07 | 0.01 | 0.01 | 0.02 |
| 88 | rs7845219 | C | -0.04 | 0.01 | 0.01 | 0.02 |
| 89 | rs7903146 | T | 0.31 | 0.01 | -0.01 | 0.02 |
| 90 | rs7929543 | C | 0.08 | 0.01 | 0.01 | 0.03 |
| 91 | rs7955901 | T | -0.04 | 0.01 | 0.03 | 0.02 |
| 92 | rs8068804 | A | 0.06 | 0.01 | -0.01 | 0.02 |
| 93 | rs8108269 | G | 0.06 | 0.01 | -0.02 | 0.02 |
| 94 | rs825476 | T | 0.05 | 0.01 | -0.01 | 0.01 |
| 95 | rs853974 | C | -0.06 | 0.01 | 0.01 | 0.02 |
| 96 | rs9369425 | A | -0.05 | 0.01 | 0.02 | 0.02 |
| 97 | rs9844972 | C | 0.10 | 0.01 | -0.03 | 0.03 |
| 98 | rs9894220 | G | -0.06 | 0.01 | 0.01 | 0.02 |
| 99 | rs9928094 | G | 0.10 | 0.01 | -0.02 | 0.02 |
| 100 | rs993380 | G | -0.05 | 0.01 | 0.01 | 0.02 |
| 101 | rs9940149 | A | -0.06 | 0.01 | 0.02 | 0.02 |

T2DM: Type-2 diabetes; HDL: High-density lipoprotein

Table 23: Genetic association estimates for the effect of T2DM on TC. ea=effect allele, gx=T2DM, gy=TC, se=standard error

|  | SNP | ea | gx | gx_se | gy | gy_se |
| --- | --- | --- | --- | --- | --- | --- |
| 1 | rs10100265 | C | -0.05 | 0.01 | -0.02 | 0.02 |
| 2 | rs10114341 | C | -0.04 | 0.01 | -0.01 | 0.01 |
| 3 | rs10401969 | C | 0.09 | 0.01 | -0.14 | 0.03 |
| 4 | rs1050226 | G | -0.05 | 0.01 | -0.01 | 0.01 |
| 5 | rs1061813 | A | -0.04 | 0.01 | 0.01 | 0.01 |
| 6 | rs1063355 | G | 0.07 | 0.01 | -0.01 | 0.02 |
| 7 | rs10740322 | A | 0.05 | 0.01 | -0.03 | 0.02 |
| 8 | rs10811661 | C | -0.16 | 0.01 | 0.06 | 0.02 |
| 9 | rs10830963 | G | 0.09 | 0.01 | -0.01 | 0.02 |
| 10 | rs10842994 | T | -0.08 | 0.01 | 0.01 | 0.02 |
| 11 | rs10974438 | C | 0.06 | 0.01 | -0.01 | 0.02 |
| 12 | rs11098676 | C | 0.05 | 0.01 | -0.01 | 0.02 |
| 13 | rs11107116 | T | 0.05 | 0.01 | 0.02 | 0.02 |
| 14 | rs1127655 | T | -0.04 | 0.01 | 0.02 | 0.01 |
| 15 | rs11708067 | G | -0.10 | 0.01 | -0.02 | 0.02 |
| 16 | rs12299509 | G | 0.05 | 0.01 | 0.01 | 0.02 |
| 17 | rs12617659 | T | -0.07 | 0.01 | 0.01 | 0.02 |
| 18 | rs12910825 | G | 0.05 | 0.01 | 0.05 | 0.02 |
| 19 | rs12945601 | C | -0.05 | 0.01 | 0.01 | 0.02 |
| 20 | rs12970134 | A | 0.06 | 0.01 | 0.01 | 0.02 |
| 21 | rs13234269 | A | -0.06 | 0.01 | 0.02 | 0.01 |
| 22 | rs13239186 | T | 0.05 | 0.01 | -0.01 | 0.02 |
| 23 | rs13330951 | G | -0.05 | 0.01 | 0.01 | 0.01 |
| 24 | rs13389219 | T | -0.07 | 0.01 | 0.02 | 0.02 |
| 25 | rs1359790 | A | -0.08 | 0.01 | 0.02 | 0.02 |
| 26 | rs1496653 | G | -0.08 | 0.01 | -0.01 | 0.02 |
| 27 | rs1552224 | C | -0.10 | 0.01 | -0.02 | 0.02 |
| 28 | rs16988333 | G | -0.07 | 0.01 | 0.02 | 0.02 |
| 29 | rs17086692 | T | -0.05 | 0.01 | 0.03 | 0.02 |
| 30 | rs17168486 | T | 0.07 | 0.01 | 0.01 | 0.02 |
| 31 | rs17405722 | A | 0.09 | 0.01 | 0.01 | 0.03 |
| 32 | rs17411031 | G | -0.05 | 0.01 | -0.01 | 0.02 |
| 33 | rs1758632 | G | 0.05 | 0.01 | -0.02 | 0.01 |
| 34 | rs17631783 | T | -0.05 | 0.01 | 0.01 | 0.02 |
| 35 | rs17791513 | G | -0.10 | 0.01 | -0.06 | 0.03 |
| 36 | rs1801214 | T | 0.09 | 0.01 | 0.01 | 0.01 |
| 37 | rs1899951 | T | -0.11 | 0.01 | 0.01 | 0.02 |
| 38 | rs2058913 | T | -0.05 | 0.01 | -0.01 | 0.01 |
| 39 | rs2237892 | T | -0.10 | 0.02 | 0.04 | 0.03 |
| 40 | rs2246618 | T | 0.05 | 0.01 | -0.02 | 0.02 |
| 41 | rs2261181 | T | 0.10 | 0.01 | -0.01 | 0.02 |
| 42 | rs2294120 | G | -0.04 | 0.01 | -0.01 | 0.01 |
| 43 | rs2296173 | G | 0.07 | 0.01 | -0.04 | 0.02 |
| 44 | rs2299383 | T | 0.04 | 0.01 | 0.01 | 0.01 |
| 45 | rs243019 | C | 0.06 | 0.01 | -0.01 | 0.01 |
| 46 | rs2493394 | G | 0.07 | 0.01 | -0.01 | 0.02 |
| 47 | rs2796441 | A | -0.07 | 0.01 | 0.02 | 0.01 |
| 48 | rs2820426 | G | 0.05 | 0.01 | 0.01 | 0.02 |
| 49 | rs2867125 | C | 0.06 | 0.01 | -0.01 | 0.02 |
| 50 | rs2925979 | C | -0.05 | 0.01 | -0.01 | 0.02 |
| 51 | rs2943656 | G | 0.09 | 0.01 | -0.01 | 0.02 |
| 52 | rs3217992 | T | 0.05 | 0.01 | 0.01 | 0.02 |
| 53 | rs3756784 | G | 0.05 | 0.01 | -0.02 | 0.02 |
| 54 | rs459193 | G | 0.07 | 0.01 | 0.01 | 0.02 |
| 55 | rs4622883 | G | -0.04 | 0.01 | -0.01 | 0.01 |
| 56 | rs4823182 | G | 0.05 | 0.01 | -0.01 | 0.02 |
| 57 | rs516946 | C | 0.08 | 0.01 | -0.01 | 0.02 |
| 58 | rs5215 | T | -0.07 | 0.01 | -0.03 | 0.02 |
| 59 | rs576674 | A | -0.07 | 0.01 | -0.01 | 0.02 |
| 60 | rs6059662 | G | 0.04 | 0.01 | -0.02 | 0.02 |
| 61 | rs61953351 | T | -0.07 | 0.01 | -0.02 | 0.02 |
| 62 | rs6494307 | G | -0.04 | 0.01 | 0.02 | 0.01 |
| 63 | rs67232546 | T | 0.06 | 0.01 | 0.01 | 0.02 |
| 64 | rs6767484 | G | 0.12 | 0.01 | 0.01 | 0.02 |
| 65 | rs6785040 | C | -0.06 | 0.01 | -0.01 | 0.02 |
| 66 | rs6795735 | T | -0.06 | 0.01 | 0.02 | 0.01 |
| 67 | rs6960043 | C | 0.06 | 0.01 | -0.01 | 0.01 |
| 68 | rs7144011 | T | 0.05 | 0.01 | -0.02 | 0.02 |
| 69 | rs7177055 | A | 0.06 | 0.01 | 0.01 | 0.02 |
| 70 | rs7240767 | C | 0.05 | 0.01 | -0.01 | 0.01 |
| 71 | rs72802358 | C | -0.12 | 0.01 | 0.01 | 0.02 |
| 72 | rs72892910 | T | 0.06 | 0.01 | 0.03 | 0.02 |
| 73 | rs735949 | C | -0.07 | 0.01 | 0.03 | 0.02 |
| 74 | rs753270 | C | 0.05 | 0.01 | 0.01 | 0.01 |
| 75 | rs7561798 | G | 0.04 | 0.01 | 0.01 | 0.01 |
| 76 | rs7572970 | G | 0.06 | 0.01 | 0.01 | 0.02 |
| 77 | rs7607777 | T | -0.14 | 0.01 | -0.01 | 0.02 |
| 78 | rs7674212 | T | -0.05 | 0.01 | -0.01 | 0.02 |
| 79 | rs7685296 | T | -0.05 | 0.01 | 0.04 | 0.02 |
| 80 | rs7729395 | T | 0.14 | 0.02 | 0.02 | 0.03 |
| 81 | rs7756992 | G | 0.13 | 0.01 | -0.01 | 0.02 |
| 82 | rs7786095 | G | -0.07 | 0.01 | 0.05 | 0.02 |
| 83 | rs780094 | C | 0.07 | 0.01 | -0.03 | 0.02 |
| 84 | rs7845219 | C | -0.04 | 0.01 | -0.01 | 0.01 |
| 85 | rs7903146 | T | 0.31 | 0.01 | -0.03 | 0.02 |
| 86 | rs7929543 | C | 0.08 | 0.01 | -0.05 | 0.03 |
| 87 | rs7955901 | T | -0.04 | 0.01 | 0.02 | 0.02 |
| 88 | rs8068804 | A | 0.06 | 0.01 | -0.01 | 0.02 |
| 89 | rs8108269 | G | 0.06 | 0.01 | 0.02 | 0.02 |
| 90 | rs825476 | T | 0.05 | 0.01 | -0.01 | 0.01 |
| 91 | rs840967 | A | -0.05 | 0.01 | -0.01 | 0.01 |
| 92 | rs849135 | A | -0.10 | 0.01 | -0.02 | 0.01 |
| 93 | rs853974 | C | -0.06 | 0.01 | 0.02 | 0.02 |
| 94 | rs9369425 | A | -0.05 | 0.01 | -0.01 | 0.02 |
| 95 | rs9894220 | G | -0.06 | 0.01 | 0.01 | 0.01 |
| 96 | rs9928094 | G | 0.10 | 0.01 | -0.01 | 0.02 |
| 97 | rs993380 | G | -0.05 | 0.01 | -0.01 | 0.02 |
| 98 | rs9940149 | A | -0.06 | 0.01 | -0.01 | 0.02 |

T2DM: Type-2 diabetes; TC: Total cholesterol

Table 24: Genetic association estimates for the effect of T2DM on WHR. ea=effect allele, gx=T2DM, gy=WHR, se=standard error

|  | SNP | ea | gx | gx_se | gy | gy_se |
| --- | --- | --- | --- | --- | --- | --- |
| 1 | rs10077431 | A | -0.05 | 0.01 | -0.01 | 0.00 |
| 2 | rs10100265 | C | -0.05 | 0.01 | 0.01 | 0.00 |
| 3 | rs10114341 | C | -0.04 | 0.01 | -0.01 | 0.00 |
| 4 | rs10401969 | C | 0.09 | 0.01 | 0.02 | 0.01 |
| 5 | rs1050226 | G | -0.05 | 0.01 | 0.00 | 0.00 |
| 6 | rs1061813 | A | -0.04 | 0.01 | 0.00 | 0.00 |
| 7 | rs1063355 | G | 0.07 | 0.01 | 0.00 | 0.01 |
| 8 | rs10740322 | A | 0.05 | 0.01 | 0.00 | 0.00 |
| 9 | rs10811661 | C | -0.16 | 0.01 | -0.02 | 0.00 |
| 10 | rs10830963 | G | 0.09 | 0.01 | 0.00 | 0.00 |
| 11 | rs10842994 | T | -0.08 | 0.01 | 0.00 | 0.00 |
| 12 | rs10974438 | C | 0.06 | 0.01 | 0.00 | 0.00 |
| 13 | rs11098676 | C | 0.05 | 0.01 | 0.02 | 0.01 |
| 14 | rs11107116 | T | 0.05 | 0.01 | 0.00 | 0.00 |
| 15 | rs11257655 | T | 0.07 | 0.01 | 0.00 | 0.00 |
| 16 | rs1127655 | T | -0.04 | 0.01 | 0.00 | 0.00 |
| 17 | rs11708067 | G | -0.10 | 0.01 | 0.00 | 0.00 |
| 18 | rs11925227 | A | -0.05 | 0.01 | 0.00 | 0.00 |
| 19 | rs11926707 | C | 0.05 | 0.01 | 0.02 | 0.00 |
| 20 | rs12088739 | G | -0.09 | 0.01 | -0.02 | 0.01 |
| 21 | rs12299509 | G | 0.05 | 0.01 | 0.00 | 0.00 |
| 22 | rs12617659 | T | -0.07 | 0.01 | 0.00 | 0.00 |
| 23 | rs12910825 | G | 0.05 | 0.01 | 0.00 | 0.00 |
| 24 | rs12945601 | C | -0.05 | 0.01 | -0.02 | 0.00 |
| 25 | rs12970134 | A | 0.06 | 0.01 | 0.00 | 0.00 |
| 26 | rs13234269 | A | -0.06 | 0.01 | -0.01 | 0.00 |
| 27 | rs13239186 | T | 0.05 | 0.01 | 0.01 | 0.00 |
| 28 | rs1333039 | C | 0.05 | 0.01 | 0.00 | 0.00 |
| 29 | rs13330951 | G | -0.05 | 0.01 | -0.01 | 0.00 |
| 30 | rs13389219 | T | -0.07 | 0.01 | -0.03 | 0.00 |
| 31 | rs1359790 | A | -0.08 | 0.01 | 0.00 | 0.00 |
| 32 | rs1496653 | G | -0.08 | 0.01 | 0.00 | 0.00 |
| 33 | rs1552224 | C | -0.10 | 0.01 | 0.00 | 0.00 |
| 34 | rs16988333 | G | -0.07 | 0.01 | -0.02 | 0.01 |
| 35 | rs17086692 | T | -0.05 | 0.01 | 0.00 | 0.00 |
| 36 | rs17168486 | T | 0.07 | 0.01 | 0.00 | 0.00 |
| 37 | rs17334919 | T | -0.14 | 0.01 | 0.02 | 0.01 |
| 38 | rs17405722 | A | 0.09 | 0.01 | 0.00 | 0.01 |
| 39 | rs17411031 | G | -0.05 | 0.01 | 0.00 | 0.00 |
| 40 | rs1758632 | G | 0.05 | 0.01 | 0.01 | 0.00 |
| 41 | rs17631783 | T | -0.05 | 0.01 | -0.01 | 0.00 |
| 42 | rs17791483 | G | -0.10 | 0.01 | 0.01 | 0.01 |
| 43 | rs1801214 | T | 0.09 | 0.01 | 0.00 | 0.00 |
| 44 | rs1899951 | T | -0.11 | 0.01 | 0.01 | 0.01 |
| 45 | rs2191348 | T | 0.07 | 0.01 | 0.00 | 0.00 |
| 46 | rs2237892 | T | -0.10 | 0.02 | 0.01 | 0.01 |
| 47 | rs2246618 | T | 0.05 | 0.01 | -0.01 | 0.00 |
| 48 | rs2261181 | T | 0.10 | 0.01 | 0.00 | 0.01 |
| 49 | rs2292662 | T | -0.06 | 0.01 | 0.00 | 0.01 |
| 50 | rs2294120 | G | -0.04 | 0.01 | 0.00 | 0.00 |
| 51 | rs2296173 | G | 0.07 | 0.01 | 0.00 | 0.00 |
| 52 | rs2299383 | T | 0.04 | 0.01 | -0.01 | 0.00 |
| 53 | rs243019 | C | 0.06 | 0.01 | -0.01 | 0.00 |
| 54 | rs2493394 | G | 0.07 | 0.01 | 0.00 | 0.01 |
| 55 | rs2796441 | A | -0.07 | 0.01 | 0.00 | 0.00 |
| 56 | rs2820426 | G | 0.05 | 0.01 | 0.03 | 0.00 |
| 57 | rs2867125 | C | 0.06 | 0.01 | -0.01 | 0.00 |
| 58 | rs2908282 | A | 0.06 | 0.01 | 0.00 | 0.00 |
| 59 | rs2925979 | C | -0.05 | 0.01 | -0.02 | 0.00 |
| 60 | rs2972144 | G | 0.09 | 0.01 | 0.00 | 0.00 |
| 61 | rs340874 | C | 0.06 | 0.01 | 0.00 | 0.00 |
| 62 | rs3756784 | G | 0.05 | 0.01 | 0.00 | 0.00 |
| 63 | rs3802177 | A | -0.12 | 0.01 | -0.01 | 0.00 |
| 64 | rs4506565 | T | 0.28 | 0.01 | 0.01 | 0.00 |
| 65 | rs459193 | G | 0.07 | 0.01 | -0.03 | 0.00 |
| 66 | rs4823182 | G | 0.05 | 0.01 | 0.01 | 0.00 |
| 67 | rs4865796 | A | 0.05 | 0.01 | 0.00 | 0.00 |
| 68 | rs516946 | C | 0.08 | 0.01 | -0.01 | 0.00 |
| 69 | rs5215 | T | -0.07 | 0.01 | -0.01 | 0.00 |
| 70 | rs576674 | A | -0.07 | 0.01 | -0.01 | 0.00 |
| 71 | rs6059662 | G | 0.04 | 0.01 | 0.00 | 0.00 |
| 72 | rs6066138 | A | -0.05 | 0.01 | -0.01 | 0.00 |
| 73 | rs622217 | C | -0.05 | 0.01 | -0.01 | 0.01 |
| 74 | rs6494307 | G | -0.04 | 0.01 | 0.00 | 0.00 |
| 75 | rs6515236 | C | -0.05 | 0.01 | 0.00 | 0.00 |
| 76 | rs6795735 | T | -0.06 | 0.01 | -0.03 | 0.00 |
| 77 | rs6878122 | A | -0.06 | 0.01 | -0.02 | 0.00 |
| 78 | rs7138300 | T | -0.04 | 0.01 | 0.00 | 0.00 |
| 79 | rs7144011 | T | 0.05 | 0.01 | 0.01 | 0.00 |
| 80 | rs7177055 | A | 0.06 | 0.01 | 0.00 | 0.00 |
| 81 | rs7185735 | G | 0.11 | 0.01 | 0.00 | 0.00 |
| 82 | rs7240767 | C | 0.05 | 0.01 | 0.00 | 0.00 |
| 83 | rs72892910 | T | 0.06 | 0.01 | 0.00 | 0.00 |
| 84 | rs735949 | C | -0.07 | 0.01 | 0.00 | 0.00 |
| 85 | rs753270 | C | 0.05 | 0.01 | 0.01 | 0.00 |
| 86 | rs7561798 | G | 0.04 | 0.01 | 0.00 | 0.00 |
| 87 | rs7572970 | G | 0.06 | 0.01 | 0.01 | 0.00 |
| 88 | rs7619041 | A | -0.04 | 0.01 | 0.01 | 0.00 |
| 89 | rs7651090 | G | 0.12 | 0.01 | 0.02 | 0.00 |
| 90 | rs7674212 | T | -0.05 | 0.01 | 0.00 | 0.00 |
| 91 | rs7685296 | T | -0.05 | 0.01 | 0.00 | 0.00 |
| 92 | rs77258096 | A | -0.12 | 0.01 | -0.01 | 0.01 |
| 93 | rs7729395 | T | 0.14 | 0.02 | -0.02 | 0.01 |
| 94 | rs7756992 | G | 0.13 | 0.01 | 0.01 | 0.00 |
| 95 | rs7786095 | G | -0.07 | 0.01 | -0.01 | 0.01 |
| 96 | rs780094 | C | 0.07 | 0.01 | -0.01 | 0.00 |
| 97 | rs7845219 | C | -0.04 | 0.01 | 0.00 | 0.00 |
| 98 | rs7923866 | T | -0.10 | 0.01 | -0.01 | 0.00 |
| 99 | rs7929543 | C | 0.08 | 0.01 | 0.00 | 0.01 |
| 100 | rs8068804 | A | 0.06 | 0.01 | 0.01 | 0.00 |
| 101 | rs8108269 | G | 0.06 | 0.01 | 0.00 | 0.00 |
| 102 | rs825476 | T | 0.05 | 0.01 | 0.02 | 0.00 |
| 103 | rs840967 | A | -0.05 | 0.01 | 0.00 | 0.00 |
| 104 | rs849135 | A | -0.10 | 0.01 | -0.01 | 0.00 |
| 105 | rs9369425 | A | -0.05 | 0.01 | 0.01 | 0.00 |
| 106 | rs963740 | T | -0.05 | 0.01 | 0.00 | 0.00 |
| 107 | rs982077 | G | -0.05 | 0.01 | -0.01 | 0.00 |
| 108 | rs9844972 | C | 0.10 | 0.01 | 0.03 | 0.01 |
| 109 | rs9894220 | G | -0.06 | 0.01 | -0.01 | 0.00 |
| 110 | rs993380 | G | -0.05 | 0.01 | 0.00 | 0.00 |
| 111 | rs9940149 | A | -0.06 | 0.01 | 0.00 | 0.00 |

T2DM: Type-2 diabetes; WHR: Waist-hip-ratio

Table 25: Genetic association estimates for the effect of T2DM on BMI. ea=effect allele, gx=T2DM, gy=BMI, se=standard error

|  | SNP | ea | gx | gx_se | gy | gy_se |
| --- | --- | --- | --- | --- | --- | --- |
| 1 | rs10077431 | A | -0.05 | 0.01 | -0.01 | 0.01 |
| 2 | rs10087241 | A | -0.05 | 0.01 | 0.00 | 0.01 |
| 3 | rs10100265 | C | -0.05 | 0.01 | 0.00 | 0.01 |
| 4 | rs10114341 | C | -0.04 | 0.01 | -0.01 | 0.01 |
| 5 | rs10401969 | C | 0.09 | 0.01 | 0.04 | 0.02 |
| 6 | rs1050226 | G | -0.05 | 0.01 | -0.01 | 0.01 |
| 7 | rs1061813 | A | -0.04 | 0.01 | -0.01 | 0.01 |
| 8 | rs1063355 | G | 0.07 | 0.01 | 0.00 | 0.01 |
| 9 | rs10740322 | A | 0.05 | 0.01 | 0.01 | 0.01 |
| 10 | rs10811661 | C | -0.16 | 0.01 | -0.01 | 0.01 |
| 11 | rs10830963 | G | 0.09 | 0.01 | 0.01 | 0.01 |
| 12 | rs10842994 | T | -0.08 | 0.01 | 0.02 | 0.01 |
| 13 | rs10974438 | C | 0.06 | 0.01 | 0.02 | 0.01 |
| 14 | rs11098676 | C | 0.05 | 0.01 | 0.00 | 0.01 |
| 15 | rs11107116 | T | 0.05 | 0.01 | 0.00 | 0.01 |
| 16 | rs11257655 | T | 0.07 | 0.01 | 0.01 | 0.01 |
| 17 | rs1127655 | T | -0.04 | 0.01 | -0.01 | 0.01 |
| 18 | rs11708067 | G | -0.10 | 0.01 | 0.00 | 0.01 |
| 19 | rs11925227 | A | -0.05 | 0.01 | 0.01 | 0.01 |
| 20 | rs11926707 | C | 0.05 | 0.01 | 0.01 | 0.01 |
| 21 | rs12088739 | G | -0.09 | 0.01 | 0.01 | 0.02 |
| 22 | rs12299509 | G | 0.05 | 0.01 | 0.00 | 0.01 |
| 23 | rs12617659 | T | -0.07 | 0.01 | 0.00 | 0.01 |
| 24 | rs12910825 | G | 0.05 | 0.01 | 0.00 | 0.01 |
| 25 | rs12945601 | C | -0.05 | 0.01 | 0.01 | 0.01 |
| 26 | rs12970134 | A | 0.06 | 0.01 | 0.00 | 0.01 |
| 27 | rs13234269 | A | -0.06 | 0.01 | 0.00 | 0.01 |
| 28 | rs13239186 | T | 0.05 | 0.01 | 0.00 | 0.01 |
| 29 | rs1333039 | C | 0.05 | 0.01 | 0.00 | 0.01 |
| 30 | rs13330951 | G | -0.05 | 0.01 | -0.01 | 0.01 |
| 31 | rs13389219 | T | -0.07 | 0.01 | -0.01 | 0.01 |
| 32 | rs1359790 | A | -0.08 | 0.01 | -0.01 | 0.01 |
| 33 | rs1496653 | G | -0.08 | 0.01 | 0.00 | 0.01 |
| 34 | rs1552224 | C | -0.10 | 0.01 | 0.00 | 0.01 |
| 35 | rs16988333 | G | -0.07 | 0.01 | -0.01 | 0.02 |
| 36 | rs17086692 | T | -0.05 | 0.01 | 0.01 | 0.01 |
| 37 | rs17168486 | T | 0.07 | 0.01 | 0.00 | 0.01 |
| 38 | rs17334919 | T | -0.14 | 0.01 | 0.01 | 0.02 |
| 39 | rs17405722 | A | 0.09 | 0.01 | -0.02 | 0.02 |
| 40 | rs17411031 | G | -0.05 | 0.01 | -0.01 | 0.01 |
| 41 | rs17631783 | T | -0.05 | 0.01 | -0.01 | 0.01 |
| 42 | rs17791483 | G | -0.10 | 0.01 | -0.02 | 0.02 |
| 43 | rs1801214 | T | 0.09 | 0.01 | 0.00 | 0.01 |
| 44 | rs1899951 | T | -0.11 | 0.01 | 0.00 | 0.01 |
| 45 | rs2191348 | T | 0.07 | 0.01 | 0.00 | 0.01 |
| 46 | rs2237892 | T | -0.10 | 0.02 | 0.03 | 0.02 |
| 47 | rs2246618 | T | 0.05 | 0.01 | 0.00 | 0.01 |
| 48 | rs2261181 | T | 0.10 | 0.01 | -0.02 | 0.02 |
| 49 | rs2292662 | T | -0.06 | 0.01 | 0.00 | 0.01 |
| 50 | rs2294120 | G | -0.04 | 0.01 | -0.01 | 0.01 |
| 51 | rs2296173 | G | 0.07 | 0.01 | 0.01 | 0.01 |
| 52 | rs2299383 | T | 0.04 | 0.01 | 0.01 | 0.01 |
| 53 | rs243019 | C | 0.06 | 0.01 | -0.01 | 0.01 |
| 54 | rs2493394 | G | 0.07 | 0.01 | -0.01 | 0.02 |
| 55 | rs2796441 | A | -0.07 | 0.01 | 0.00 | 0.01 |
| 56 | rs2820426 | G | 0.05 | 0.01 | 0.00 | 0.01 |
| 57 | rs2867125 | C | 0.06 | 0.01 | -0.01 | 0.01 |
| 58 | rs2908282 | A | 0.06 | 0.01 | -0.01 | 0.01 |
| 59 | rs2925979 | C | -0.05 | 0.01 | 0.00 | 0.01 |
| 60 | rs2972144 | G | 0.09 | 0.01 | 0.00 | 0.01 |
| 61 | rs340874 | C | 0.06 | 0.01 | 0.00 | 0.01 |
| 62 | rs348330 | A | -0.05 | 0.01 | -0.01 | 0.01 |
| 63 | rs3756784 | G | 0.05 | 0.01 | -0.01 | 0.01 |
| 64 | rs3802177 | A | -0.12 | 0.01 | 0.00 | 0.01 |
| 65 | rs4506565 | T | 0.28 | 0.01 | 0.00 | 0.01 |
| 66 | rs459193 | G | 0.07 | 0.01 | 0.01 | 0.01 |
| 67 | rs4686471 | C | 0.05 | 0.01 | 0.00 | 0.01 |
| 68 | rs4810426 | T | 0.07 | 0.01 | 0.00 | 0.02 |
| 69 | rs4823182 | G | 0.05 | 0.01 | 0.00 | 0.01 |
| 70 | rs4865796 | A | 0.05 | 0.01 | 0.01 | 0.01 |
| 71 | rs516946 | C | 0.08 | 0.01 | -0.01 | 0.01 |
| 72 | rs5215 | T | -0.07 | 0.01 | 0.00 | 0.01 |
| 73 | rs576674 | A | -0.07 | 0.01 | -0.01 | 0.01 |
| 74 | rs6059662 | G | 0.04 | 0.01 | 0.00 | 0.01 |
| 75 | rs6066138 | A | -0.05 | 0.01 | 0.01 | 0.01 |
| 76 | rs61953351 | T | -0.07 | 0.01 | 0.01 | 0.01 |
| 77 | rs622217 | C | -0.05 | 0.01 | 0.00 | 0.01 |
| 78 | rs6494307 | G | -0.04 | 0.01 | 0.00 | 0.01 |
| 79 | rs6515236 | C | -0.05 | 0.01 | 0.00 | 0.01 |
| 80 | rs67232546 | T | 0.06 | 0.01 | 0.01 | 0.01 |
| 81 | rs6795735 | T | -0.06 | 0.01 | 0.00 | 0.01 |
| 82 | rs6878122 | A | -0.06 | 0.01 | 0.00 | 0.01 |
| 83 | rs7138300 | T | -0.04 | 0.01 | -0.01 | 0.01 |
| 84 | rs7144011 | T | 0.05 | 0.01 | -0.01 | 0.01 |
| 85 | rs7177055 | A | 0.06 | 0.01 | 0.00 | 0.01 |
| 86 | rs7185735 | G | 0.11 | 0.01 | -0.02 | 0.01 |
| 87 | rs7240767 | C | 0.05 | 0.01 | 0.00 | 0.01 |
| 88 | rs72892910 | T | 0.06 | 0.01 | 0.00 | 0.01 |
| 89 | rs735949 | C | -0.07 | 0.01 | 0.00 | 0.01 |
| 90 | rs753270 | C | 0.05 | 0.01 | 0.01 | 0.01 |
| 91 | rs7561798 | G | 0.04 | 0.01 | 0.00 | 0.01 |
| 92 | rs7572970 | G | 0.06 | 0.01 | 0.00 | 0.01 |
| 93 | rs7619041 | A | -0.04 | 0.01 | 0.00 | 0.01 |
| 94 | rs7651090 | G | 0.12 | 0.01 | 0.01 | 0.01 |
| 95 | rs7674212 | T | -0.05 | 0.01 | -0.01 | 0.01 |
| 96 | rs7685296 | T | -0.05 | 0.01 | 0.00 | 0.01 |
| 97 | rs77258096 | A | -0.12 | 0.01 | -0.03 | 0.02 |
| 98 | rs7729395 | T | 0.14 | 0.02 | 0.00 | 0.03 |
| 99 | rs7756992 | G | 0.13 | 0.01 | -0.01 | 0.01 |
| 100 | rs7786095 | G | -0.07 | 0.01 | 0.00 | 0.02 |
| 101 | rs780094 | C | 0.07 | 0.01 | 0.00 | 0.01 |
| 102 | rs7845219 | C | -0.04 | 0.01 | 0.01 | 0.01 |
| 103 | rs7923866 | T | -0.10 | 0.01 | 0.01 | 0.01 |
| 104 | rs7929543 | C | 0.08 | 0.01 | -0.04 | 0.02 |
| 105 | rs8068804 | A | 0.06 | 0.01 | 0.01 | 0.01 |
| 106 | rs8108269 | G | 0.06 | 0.01 | 0.01 | 0.01 |
| 107 | rs825476 | T | 0.05 | 0.01 | 0.00 | 0.01 |
| 108 | rs840967 | A | -0.05 | 0.01 | -0.01 | 0.01 |
| 109 | rs849135 | A | -0.10 | 0.01 | 0.01 | 0.01 |
| 110 | rs853974 | C | -0.06 | 0.01 | -0.01 | 0.01 |
| 111 | rs9369425 | A | -0.05 | 0.01 | 0.00 | 0.01 |
| 112 | rs963740 | T | -0.05 | 0.01 | 0.00 | 0.01 |
| 113 | rs982077 | G | -0.05 | 0.01 | 0.00 | 0.01 |
| 114 | rs9844972 | C | 0.10 | 0.01 | -0.01 | 0.03 |
| 115 | rs9894220 | G | -0.06 | 0.01 | 0.00 | 0.01 |
| 116 | rs993380 | G | -0.05 | 0.01 | -0.01 | 0.01 |
| 117 | rs9940149 | A | -0.06 | 0.01 | 0.01 | 0.01 |

T2DM: Type-2 diabetes; BMI: Body mass index

Table 26: Genetic association estimates for the effect of T2DM on VLDL. ea=effect allele, gx=T2DM, gy=VLDL, se=standard error

|  | SNP | ea | gx | gx_se | gy | gy_se |
| --- | --- | --- | --- | --- | --- | --- |
| 1 | rs10077431 | A | -0.05 | 0.01 | -0.02 | 0.01 |
| 2 | rs10087241 | A | -0.05 | 0.01 | -0.01 | 0.01 |
| 3 | rs10100265 | C | -0.05 | 0.01 | 0.01 | 0.01 |
| 4 | rs10114341 | C | -0.04 | 0.01 | -0.01 | 0.01 |
| 5 | rs10401969 | C | 0.09 | 0.01 | -0.10 | 0.02 |
| 6 | rs1050226 | G | -0.05 | 0.01 | 0.00 | 0.01 |
| 7 | rs1061813 | A | -0.04 | 0.01 | -0.01 | 0.01 |
| 8 | rs1063355 | G | 0.07 | 0.01 | -0.02 | 0.01 |
| 9 | rs10740322 | A | 0.05 | 0.01 | 0.01 | 0.01 |
| 10 | rs10811661 | C | -0.16 | 0.01 | 0.00 | 0.01 |
| 11 | rs10830963 | G | 0.09 | 0.01 | 0.00 | 0.01 |
| 12 | rs10842994 | T | -0.08 | 0.01 | -0.01 | 0.01 |
| 13 | rs10974438 | C | 0.06 | 0.01 | 0.01 | 0.01 |
| 14 | rs11098676 | C | 0.05 | 0.01 | 0.00 | 0.01 |
| 15 | rs11107116 | T | 0.05 | 0.01 | 0.01 | 0.01 |
| 16 | rs11257655 | T | 0.07 | 0.01 | 0.00 | 0.01 |
| 17 | rs1127655 | T | -0.04 | 0.01 | -0.01 | 0.01 |
| 18 | rs11708067 | G | -0.10 | 0.01 | -0.01 | 0.01 |
| 19 | rs11925227 | A | -0.05 | 0.01 | 0.01 | 0.01 |
| 20 | rs11926707 | C | 0.05 | 0.01 | 0.00 | 0.01 |
| 21 | rs12088739 | G | -0.09 | 0.01 | 0.02 | 0.02 |
| 22 | rs12299509 | G | 0.05 | 0.01 | 0.01 | 0.01 |
| 23 | rs12617659 | T | -0.07 | 0.01 | 0.01 | 0.01 |
| 24 | rs12910825 | G | 0.05 | 0.01 | -0.01 | 0.01 |
| 25 | rs12945601 | C | -0.05 | 0.01 | 0.01 | 0.01 |
| 26 | rs12970134 | A | 0.06 | 0.01 | 0.02 | 0.01 |
| 27 | rs13234269 | A | -0.06 | 0.01 | -0.01 | 0.01 |
| 28 | rs13239186 | T | 0.05 | 0.01 | -0.01 | 0.01 |
| 29 | rs1333039 | C | 0.05 | 0.01 | -0.02 | 0.01 |
| 30 | rs13330951 | G | -0.05 | 0.01 | 0.01 | 0.01 |
| 31 | rs13389219 | T | -0.07 | 0.01 | -0.01 | 0.01 |
| 32 | rs1359790 | A | -0.08 | 0.01 | 0.02 | 0.01 |
| 33 | rs1496653 | G | -0.08 | 0.01 | 0.01 | 0.01 |
| 34 | rs1552224 | C | -0.10 | 0.01 | -0.01 | 0.01 |
| 35 | rs16988333 | G | -0.07 | 0.01 | 0.01 | 0.02 |
| 36 | rs17086692 | T | -0.05 | 0.01 | 0.01 | 0.01 |
| 37 | rs17168486 | T | 0.07 | 0.01 | 0.01 | 0.01 |
| 38 | rs17334919 | T | -0.14 | 0.01 | -0.04 | 0.02 |
| 39 | rs17405722 | A | 0.09 | 0.01 | 0.01 | 0.02 |
| 40 | rs17411031 | G | -0.05 | 0.01 | -0.02 | 0.01 |
| 41 | rs1758632 | G | 0.05 | 0.01 | -0.01 | 0.01 |
| 42 | rs17631783 | T | -0.05 | 0.01 | -0.01 | 0.01 |
| 43 | rs17791483 | G | -0.10 | 0.01 | 0.00 | 0.02 |
| 44 | rs1801214 | T | 0.09 | 0.01 | 0.02 | 0.01 |
| 45 | rs1899951 | T | -0.11 | 0.01 | 0.00 | 0.01 |
| 46 | rs2191348 | T | 0.07 | 0.01 | -0.01 | 0.01 |
| 47 | rs2237892 | T | -0.10 | 0.02 | -0.01 | 0.02 |
| 48 | rs2246618 | T | 0.05 | 0.01 | 0.01 | 0.01 |
| 49 | rs2261181 | T | 0.10 | 0.01 | 0.00 | 0.02 |
| 50 | rs2292662 | T | -0.06 | 0.01 | -0.01 | 0.01 |
| 51 | rs2294120 | G | -0.04 | 0.01 | 0.00 | 0.01 |
| 52 | rs2296173 | G | 0.07 | 0.01 | 0.00 | 0.01 |
| 53 | rs2299383 | T | 0.04 | 0.01 | 0.01 | 0.01 |
| 54 | rs243019 | C | 0.06 | 0.01 | -0.01 | 0.01 |
| 55 | rs2493394 | G | 0.07 | 0.01 | 0.03 | 0.02 |
| 56 | rs2796441 | A | -0.07 | 0.01 | 0.01 | 0.01 |
| 57 | rs2820426 | G | 0.05 | 0.01 | 0.02 | 0.01 |
| 58 | rs2867125 | C | 0.06 | 0.01 | 0.01 | 0.01 |
| 59 | rs2908282 | A | 0.06 | 0.01 | -0.02 | 0.02 |
| 60 | rs2925979 | C | -0.05 | 0.01 | 0.00 | 0.01 |
| 61 | rs2972144 | G | 0.09 | 0.01 | 0.01 | 0.01 |
| 62 | rs340874 | C | 0.06 | 0.01 | 0.01 | 0.01 |
| 63 | rs348330 | A | -0.05 | 0.01 | 0.00 | 0.01 |
| 64 | rs3756784 | G | 0.05 | 0.01 | -0.01 | 0.01 |
| 65 | rs3802177 | A | -0.12 | 0.01 | -0.01 | 0.01 |
| 66 | rs4506565 | T | 0.28 | 0.01 | 0.01 | 0.01 |
| 67 | rs459193 | G | 0.07 | 0.01 | -0.01 | 0.01 |
| 68 | rs4686471 | C | 0.05 | 0.01 | -0.01 | 0.01 |
| 69 | rs4810426 | T | 0.07 | 0.01 | -0.01 | 0.01 |
| 70 | rs4823182 | G | 0.05 | 0.01 | 0.01 | 0.01 |
| 71 | rs4865796 | A | 0.05 | 0.01 | 0.01 | 0.01 |
| 72 | rs516946 | C | 0.08 | 0.01 | 0.00 | 0.01 |
| 73 | rs5215 | T | -0.07 | 0.01 | -0.01 | 0.01 |
| 74 | rs576674 | A | -0.07 | 0.01 | 0.01 | 0.02 |
| 75 | rs6059662 | G | 0.04 | 0.01 | 0.00 | 0.01 |
| 76 | rs6066138 | A | -0.05 | 0.01 | -0.01 | 0.01 |
| 77 | rs61953351 | T | -0.07 | 0.01 | -0.03 | 0.01 |
| 78 | rs622217 | C | -0.05 | 0.01 | -0.02 | 0.01 |
| 79 | rs6494307 | G | -0.04 | 0.01 | 0.01 | 0.01 |
| 80 | rs6515236 | C | -0.05 | 0.01 | 0.00 | 0.01 |
| 81 | rs67232546 | T | 0.06 | 0.01 | 0.02 | 0.01 |
| 82 | rs6795735 | T | -0.06 | 0.01 | 0.01 | 0.01 |
| 83 | rs6878122 | A | -0.06 | 0.01 | -0.02 | 0.01 |
| 84 | rs7138300 | T | -0.04 | 0.01 | 0.01 | 0.01 |
| 85 | rs7144011 | T | 0.05 | 0.01 | 0.00 | 0.01 |
| 86 | rs7177055 | A | 0.06 | 0.01 | 0.00 | 0.01 |
| 87 | rs7185735 | G | 0.11 | 0.01 | 0.02 | 0.01 |
| 88 | rs7240767 | C | 0.05 | 0.01 | 0.01 | 0.01 |
| 89 | rs72892910 | T | 0.06 | 0.01 | 0.01 | 0.01 |
| 90 | rs735949 | C | -0.07 | 0.01 | 0.00 | 0.02 |
| 91 | rs753270 | C | 0.05 | 0.01 | 0.02 | 0.01 |
| 92 | rs7561798 | G | 0.04 | 0.01 | 0.00 | 0.01 |
| 93 | rs7572970 | G | 0.06 | 0.01 | 0.00 | 0.01 |
| 94 | rs7619041 | A | -0.04 | 0.01 | 0.00 | 0.01 |
| 95 | rs7651090 | G | 0.12 | 0.01 | 0.00 | 0.01 |
| 96 | rs7674212 | T | -0.05 | 0.01 | -0.01 | 0.01 |
| 97 | rs7685296 | T | -0.05 | 0.01 | 0.00 | 0.01 |
| 98 | rs77258096 | A | -0.12 | 0.01 | 0.01 | 0.02 |
| 99 | rs7729395 | T | 0.14 | 0.02 | -0.03 | 0.02 |
| 100 | rs7756992 | G | 0.13 | 0.01 | -0.02 | 0.01 |
| 101 | rs7786095 | G | -0.07 | 0.01 | -0.02 | 0.02 |
| 102 | rs780094 | C | 0.07 | 0.01 | -0.04 | 0.01 |
| 103 | rs7845219 | C | -0.04 | 0.01 | 0.01 | 0.01 |
| 104 | rs7923866 | T | -0.10 | 0.01 | -0.02 | 0.01 |
| 105 | rs7929543 | C | 0.08 | 0.01 | 0.00 | 0.02 |
| 106 | rs8068804 | A | 0.06 | 0.01 | -0.01 | 0.01 |
| 107 | rs8108269 | G | 0.06 | 0.01 | 0.02 | 0.01 |
| 108 | rs825476 | T | 0.05 | 0.01 | 0.02 | 0.01 |
| 109 | rs840967 | A | -0.05 | 0.01 | -0.01 | 0.01 |
| 110 | rs849135 | A | -0.10 | 0.01 | 0.01 | 0.01 |
| 111 | rs853974 | C | -0.06 | 0.01 | 0.02 | 0.01 |
| 112 | rs9369425 | A | -0.05 | 0.01 | 0.01 | 0.01 |
| 113 | rs963740 | T | -0.05 | 0.01 | -0.01 | 0.01 |
| 114 | rs982077 | G | -0.05 | 0.01 | 0.00 | 0.01 |
| 115 | rs9844972 | C | 0.10 | 0.01 | 0.00 | 0.02 |
| 116 | rs9894220 | G | -0.06 | 0.01 | 0.00 | 0.01 |
| 117 | rs993380 | G | -0.05 | 0.01 | 0.00 | 0.01 |
| 118 | rs9940149 | A | -0.06 | 0.01 | 0.02 | 0.02 |

T2DM: Type-2 diabetes; VLDL: Very low-density lipoprotein

Table 27: Genetic association estimates for the effect of T2DM on hyperthyroidism. ea=effect allele, gx=T2DM, gy=hyperthyroidism, se=standard error

|  | SNP | ea | gx | gx_se | gy | gy_se |
| --- | --- | --- | --- | --- | --- | --- |
| 1 | rs10077431 | A | -0.05 | 0.01 | 0.00 | 0.00 |
| 2 | rs10087241 | A | -0.05 | 0.01 | 0.00 | 0.00 |
| 3 | rs10100265 | C | -0.05 | 0.01 | 0.00 | 0.00 |
| 4 | rs10114341 | C | -0.04 | 0.01 | 0.00 | 0.00 |
| 5 | rs1050226 | G | -0.05 | 0.01 | 0.00 | 0.00 |
| 6 | rs1061813 | A | -0.04 | 0.01 | 0.00 | 0.00 |
| 7 | rs1063355 | G | 0.07 | 0.01 | 0.00 | 0.00 |
| 8 | rs10740322 | A | 0.05 | 0.01 | 0.00 | 0.00 |
| 9 | rs10811661 | C | -0.16 | 0.01 | 0.00 | 0.00 |
| 10 | rs10830963 | G | 0.09 | 0.01 | 0.00 | 0.00 |
| 11 | rs10842994 | T | -0.08 | 0.01 | 0.00 | 0.00 |
| 12 | rs10974438 | C | 0.06 | 0.01 | 0.00 | 0.00 |
| 13 | rs11098676 | C | 0.05 | 0.01 | 0.00 | 0.00 |
| 14 | rs11107116 | T | 0.05 | 0.01 | 0.00 | 0.00 |
| 15 | rs11257655 | T | 0.07 | 0.01 | 0.00 | 0.00 |
| 16 | rs1127655 | T | -0.04 | 0.01 | 0.00 | 0.00 |
| 17 | rs11708067 | G | -0.10 | 0.01 | 0.00 | 0.00 |
| 18 | rs11925227 | A | -0.05 | 0.01 | 0.00 | 0.00 |
| 19 | rs11926707 | C | 0.05 | 0.01 | 0.00 | 0.00 |
| 20 | rs12299509 | G | 0.05 | 0.01 | 0.00 | 0.00 |
| 21 | rs12617659 | T | -0.07 | 0.01 | 0.00 | 0.00 |
| 22 | rs12910825 | G | 0.05 | 0.01 | 0.00 | 0.00 |
| 23 | rs12945601 | C | -0.05 | 0.01 | 0.00 | 0.00 |
| 24 | rs12970134 | A | 0.06 | 0.01 | 0.00 | 0.00 |
| 25 | rs13234269 | A | -0.06 | 0.01 | 0.00 | 0.00 |
| 26 | rs13239186 | T | 0.05 | 0.01 | 0.00 | 0.00 |
| 27 | rs1333039 | C | 0.05 | 0.01 | 0.00 | 0.00 |
| 28 | rs13330951 | G | -0.05 | 0.01 | 0.00 | 0.00 |
| 29 | rs13389219 | T | -0.07 | 0.01 | 0.00 | 0.00 |
| 30 | rs1359790 | A | -0.08 | 0.01 | 0.00 | 0.00 |
| 31 | rs1496653 | G | -0.08 | 0.01 | 0.00 | 0.00 |
| 32 | rs1552224 | C | -0.10 | 0.01 | 0.00 | 0.00 |
| 33 | rs17086692 | T | -0.05 | 0.01 | 0.00 | 0.00 |
| 34 | rs17168486 | T | 0.07 | 0.01 | 0.00 | 0.00 |
| 35 | rs17334919 | T | -0.14 | 0.01 | 0.00 | 0.00 |
| 36 | rs17411031 | G | -0.05 | 0.01 | 0.00 | 0.00 |
| 37 | rs1758632 | G | 0.05 | 0.01 | 0.00 | 0.00 |
| 38 | rs17631783 | T | -0.05 | 0.01 | 0.00 | 0.00 |
| 39 | rs1801214 | T | 0.09 | 0.01 | 0.00 | 0.00 |
| 40 | rs1899951 | T | -0.11 | 0.01 | 0.00 | 0.00 |
| 41 | rs2191348 | T | 0.07 | 0.01 | 0.00 | 0.00 |
| 42 | rs2246618 | T | 0.05 | 0.01 | 0.00 | 0.00 |
| 43 | rs2261181 | T | 0.10 | 0.01 | 0.00 | 0.00 |
| 44 | rs2292662 | T | -0.06 | 0.01 | 0.00 | 0.00 |
| 45 | rs2294120 | G | -0.04 | 0.01 | 0.00 | 0.00 |
| 46 | rs2296173 | G | 0.07 | 0.01 | 0.00 | 0.00 |
| 47 | rs2299383 | T | 0.04 | 0.01 | 0.00 | 0.00 |
| 48 | rs243019 | C | 0.06 | 0.01 | 0.00 | 0.00 |
| 49 | rs2493394 | G | 0.07 | 0.01 | 0.00 | 0.00 |
| 50 | rs2796441 | A | -0.07 | 0.01 | 0.00 | 0.00 |
| 51 | rs2820426 | G | 0.05 | 0.01 | 0.00 | 0.00 |
| 52 | rs2867125 | C | 0.06 | 0.01 | 0.00 | 0.00 |
| 53 | rs2908282 | A | 0.06 | 0.01 | 0.00 | 0.00 |
| 54 | rs2925979 | C | -0.05 | 0.01 | 0.00 | 0.00 |
| 55 | rs2972144 | G | 0.09 | 0.01 | 0.00 | 0.00 |
| 56 | rs340874 | C | 0.06 | 0.01 | 0.00 | 0.00 |
| 57 | rs348330 | A | -0.05 | 0.01 | 0.00 | 0.00 |
| 58 | rs3756784 | G | 0.05 | 0.01 | 0.00 | 0.00 |
| 59 | rs3802177 | A | -0.12 | 0.01 | 0.00 | 0.00 |
| 60 | rs4506565 | T | 0.28 | 0.01 | 0.00 | 0.00 |
| 61 | rs459193 | G | 0.07 | 0.01 | 0.00 | 0.00 |
| 62 | rs4686471 | C | 0.05 | 0.01 | 0.00 | 0.00 |
| 63 | rs4823182 | G | 0.05 | 0.01 | 0.00 | 0.00 |
| 64 | rs4865796 | A | 0.05 | 0.01 | 0.00 | 0.00 |
| 65 | rs516946 | C | 0.08 | 0.01 | 0.00 | 0.00 |
| 66 | rs5215 | T | -0.07 | 0.01 | 0.00 | 0.00 |
| 67 | rs576674 | A | -0.07 | 0.01 | 0.00 | 0.00 |
| 68 | rs6059662 | G | 0.04 | 0.01 | 0.00 | 0.00 |
| 69 | rs6066138 | A | -0.05 | 0.01 | 0.00 | 0.00 |
| 70 | rs61953351 | T | -0.07 | 0.01 | 0.00 | 0.00 |
| 71 | rs622217 | C | -0.05 | 0.01 | 0.00 | 0.00 |
| 72 | rs6494307 | G | -0.04 | 0.01 | 0.00 | 0.00 |
| 73 | rs6515236 | C | -0.05 | 0.01 | 0.00 | 0.00 |
| 74 | rs67232546 | T | 0.06 | 0.01 | 0.00 | 0.00 |
| 75 | rs6795735 | T | -0.06 | 0.01 | 0.00 | 0.00 |
| 76 | rs6878122 | A | -0.06 | 0.01 | 0.00 | 0.00 |
| 77 | rs7138300 | T | -0.04 | 0.01 | 0.00 | 0.00 |
| 78 | rs7144011 | T | 0.05 | 0.01 | 0.00 | 0.00 |
| 79 | rs7177055 | A | 0.06 | 0.01 | 0.00 | 0.00 |
| 80 | rs7185735 | G | 0.11 | 0.01 | 0.00 | 0.00 |
| 81 | rs7240767 | C | 0.05 | 0.01 | 0.00 | 0.00 |
| 82 | rs72892910 | T | 0.06 | 0.01 | 0.00 | 0.00 |
| 83 | rs735949 | C | -0.07 | 0.01 | 0.00 | 0.00 |
| 84 | rs753270 | C | 0.05 | 0.01 | 0.00 | 0.00 |
| 85 | rs7561798 | G | 0.04 | 0.01 | 0.00 | 0.00 |
| 86 | rs7572970 | G | 0.06 | 0.01 | 0.00 | 0.00 |
| 87 | rs7619041 | A | -0.04 | 0.01 | 0.00 | 0.00 |
| 88 | rs7651090 | G | 0.12 | 0.01 | 0.00 | 0.00 |
| 89 | rs7674212 | T | -0.05 | 0.01 | 0.00 | 0.00 |
| 90 | rs7685296 | T | -0.05 | 0.01 | 0.00 | 0.00 |
| 91 | rs77258096 | A | -0.12 | 0.01 | 0.00 | 0.00 |
| 92 | rs7756992 | G | 0.13 | 0.01 | 0.00 | 0.00 |
| 93 | rs7786095 | G | -0.07 | 0.01 | 0.00 | 0.00 |
| 94 | rs780094 | C | 0.07 | 0.01 | 0.00 | 0.00 |
| 95 | rs7845219 | C | -0.04 | 0.01 | 0.00 | 0.00 |
| 96 | rs7923866 | T | -0.10 | 0.01 | 0.00 | 0.00 |
| 97 | rs8068804 | A | 0.06 | 0.01 | 0.00 | 0.00 |
| 98 | rs8108269 | G | 0.06 | 0.01 | 0.00 | 0.00 |
| 99 | rs825476 | T | 0.05 | 0.01 | 0.00 | 0.00 |
| 100 | rs840967 | A | -0.05 | 0.01 | 0.00 | 0.00 |
| 101 | rs849135 | A | -0.10 | 0.01 | 0.00 | 0.00 |
| 102 | rs853974 | C | -0.06 | 0.01 | 0.00 | 0.00 |
| 103 | rs9369425 | A | -0.05 | 0.01 | 0.00 | 0.00 |
| 104 | rs963740 | T | -0.05 | 0.01 | 0.00 | 0.00 |
| 105 | rs982077 | G | -0.05 | 0.01 | 0.00 | 0.00 |
| 106 | rs9894220 | G | -0.06 | 0.01 | 0.00 | 0.00 |
| 107 | rs993380 | G | -0.05 | 0.01 | 0.00 | 0.00 |
| 108 | rs9940149 | A | -0.06 | 0.01 | 0.00 | 0.00 |

T2DM: Type-2 diabetes

Table 28: Genetic association estimates for the effect of T2DM on hypothyroidism. ea=effect allele, gx=T2DM, gy=hypothyroidism, se=standard error

|  | SNP | ea | gx | gx_se | gy | gy_se |
| --- | --- | --- | --- | --- | --- | --- |
| 1 | rs10077431 | A | -0.05 | 0.01 | 0.00 | 0.00 |
| 2 | rs10087241 | A | -0.05 | 0.01 | 0.00 | 0.00 |
| 3 | rs10100265 | C | -0.05 | 0.01 | 0.00 | 0.00 |
| 4 | rs10114341 | C | -0.04 | 0.01 | 0.00 | 0.00 |
| 5 | rs10401969 | C | 0.09 | 0.01 | 0.00 | 0.00 |
| 6 | rs1050226 | G | -0.05 | 0.01 | 0.00 | 0.00 |
| 7 | rs1061813 | A | -0.04 | 0.01 | 0.00 | 0.00 |
| 8 | rs1063355 | G | 0.07 | 0.01 | 0.01 | 0.00 |
| 9 | rs10740322 | A | 0.05 | 0.01 | 0.00 | 0.00 |
| 10 | rs10811661 | C | -0.16 | 0.01 | 0.00 | 0.00 |
| 11 | rs10830963 | G | 0.09 | 0.01 | 0.00 | 0.00 |
| 12 | rs10842994 | T | -0.08 | 0.01 | 0.00 | 0.00 |
| 13 | rs10974438 | C | 0.06 | 0.01 | 0.00 | 0.00 |
| 14 | rs11098676 | C | 0.05 | 0.01 | 0.00 | 0.00 |
| 15 | rs11107116 | T | 0.05 | 0.01 | 0.00 | 0.00 |
| 16 | rs11257655 | T | 0.07 | 0.01 | 0.00 | 0.00 |
| 17 | rs1127655 | T | -0.04 | 0.01 | 0.00 | 0.00 |
| 18 | rs11708067 | G | -0.10 | 0.01 | 0.00 | 0.00 |
| 19 | rs11925227 | A | -0.05 | 0.01 | 0.00 | 0.00 |
| 20 | rs11926707 | C | 0.05 | 0.01 | 0.00 | 0.00 |
| 21 | rs12088739 | G | -0.09 | 0.01 | 0.00 | 0.00 |
| 22 | rs12299509 | G | 0.05 | 0.01 | 0.00 | 0.00 |
| 23 | rs12617659 | T | -0.07 | 0.01 | 0.00 | 0.00 |
| 24 | rs12910825 | G | 0.05 | 0.01 | 0.00 | 0.00 |
| 25 | rs12945601 | C | -0.05 | 0.01 | 0.00 | 0.00 |
| 26 | rs12970134 | A | 0.06 | 0.01 | 0.00 | 0.00 |
| 27 | rs13234269 | A | -0.06 | 0.01 | 0.00 | 0.00 |
| 28 | rs13239186 | T | 0.05 | 0.01 | 0.00 | 0.00 |
| 29 | rs1333039 | C | 0.05 | 0.01 | 0.00 | 0.00 |
| 30 | rs13330951 | G | -0.05 | 0.01 | 0.00 | 0.00 |
| 31 | rs13389219 | T | -0.07 | 0.01 | 0.00 | 0.00 |
| 32 | rs1359790 | A | -0.08 | 0.01 | 0.00 | 0.00 |
| 33 | rs1496653 | G | -0.08 | 0.01 | 0.00 | 0.00 |
| 34 | rs1552224 | C | -0.10 | 0.01 | 0.00 | 0.00 |
| 35 | rs16988333 | G | -0.07 | 0.01 | 0.00 | 0.00 |
| 36 | rs17086692 | T | -0.05 | 0.01 | 0.00 | 0.00 |
| 37 | rs17168486 | T | 0.07 | 0.01 | 0.00 | 0.00 |
| 38 | rs17334919 | T | -0.14 | 0.01 | 0.00 | 0.00 |
| 39 | rs17405722 | A | 0.09 | 0.01 | 0.00 | 0.00 |
| 40 | rs17411031 | G | -0.05 | 0.01 | 0.00 | 0.00 |
| 41 | rs1758632 | G | 0.05 | 0.01 | 0.00 | 0.00 |
| 42 | rs17631783 | T | -0.05 | 0.01 | 0.00 | 0.00 |
| 43 | rs17791483 | G | -0.10 | 0.01 | 0.00 | 0.00 |
| 44 | rs1801214 | T | 0.09 | 0.01 | 0.00 | 0.00 |
| 45 | rs1899951 | T | -0.11 | 0.01 | 0.00 | 0.00 |
| 46 | rs2191348 | T | 0.07 | 0.01 | 0.00 | 0.00 |
| 47 | rs2237892 | T | -0.10 | 0.02 | 0.00 | 0.00 |
| 48 | rs2246618 | T | 0.05 | 0.01 | 0.00 | 0.00 |
| 49 | rs2261181 | T | 0.10 | 0.01 | 0.00 | 0.00 |
| 50 | rs2292662 | T | -0.06 | 0.01 | 0.00 | 0.00 |
| 51 | rs2294120 | G | -0.04 | 0.01 | 0.00 | 0.00 |
| 52 | rs2296173 | G | 0.07 | 0.01 | 0.00 | 0.00 |
| 53 | rs2299383 | T | 0.04 | 0.01 | 0.00 | 0.00 |
| 54 | rs243019 | C | 0.06 | 0.01 | 0.00 | 0.00 |
| 55 | rs2493394 | G | 0.07 | 0.01 | 0.00 | 0.00 |
| 56 | rs2796441 | A | -0.07 | 0.01 | 0.00 | 0.00 |
| 57 | rs2820426 | G | 0.05 | 0.01 | 0.00 | 0.00 |
| 58 | rs2867125 | C | 0.06 | 0.01 | 0.00 | 0.00 |
| 59 | rs2908282 | A | 0.06 | 0.01 | 0.00 | 0.00 |
| 60 | rs2925979 | C | -0.05 | 0.01 | 0.00 | 0.00 |
| 61 | rs2972144 | G | 0.09 | 0.01 | 0.00 | 0.00 |
| 62 | rs340874 | C | 0.06 | 0.01 | 0.00 | 0.00 |
| 63 | rs348330 | A | -0.05 | 0.01 | 0.00 | 0.00 |
| 64 | rs3756784 | G | 0.05 | 0.01 | 0.00 | 0.00 |
| 65 | rs3802177 | A | -0.12 | 0.01 | 0.00 | 0.00 |
| 66 | rs4506565 | T | 0.28 | 0.01 | 0.00 | 0.00 |
| 67 | rs459193 | G | 0.07 | 0.01 | 0.00 | 0.00 |
| 68 | rs4686471 | C | 0.05 | 0.01 | 0.00 | 0.00 |
| 69 | rs4810426 | T | 0.07 | 0.01 | 0.00 | 0.00 |
| 70 | rs4823182 | G | 0.05 | 0.01 | 0.00 | 0.00 |
| 71 | rs4865796 | A | 0.05 | 0.01 | 0.00 | 0.00 |
| 72 | rs516946 | C | 0.08 | 0.01 | 0.00 | 0.00 |
| 73 | rs5215 | T | -0.07 | 0.01 | 0.00 | 0.00 |
| 74 | rs576674 | A | -0.07 | 0.01 | 0.00 | 0.00 |
| 75 | rs6059662 | G | 0.04 | 0.01 | 0.00 | 0.00 |
| 76 | rs6066138 | A | -0.05 | 0.01 | 0.00 | 0.00 |
| 77 | rs61953351 | T | -0.07 | 0.01 | 0.00 | 0.00 |
| 78 | rs622217 | C | -0.05 | 0.01 | 0.00 | 0.00 |
| 79 | rs6494307 | G | -0.04 | 0.01 | 0.00 | 0.00 |
| 80 | rs6515236 | C | -0.05 | 0.01 | 0.00 | 0.00 |
| 81 | rs67232546 | T | 0.06 | 0.01 | 0.00 | 0.00 |
| 82 | rs6795735 | T | -0.06 | 0.01 | 0.00 | 0.00 |
| 83 | rs6878122 | A | -0.06 | 0.01 | 0.00 | 0.00 |
| 84 | rs7138300 | T | -0.04 | 0.01 | 0.00 | 0.00 |
| 85 | rs7144011 | T | 0.05 | 0.01 | 0.00 | 0.00 |
| 86 | rs7177055 | A | 0.06 | 0.01 | 0.00 | 0.00 |
| 87 | rs7185735 | G | 0.11 | 0.01 | 0.00 | 0.00 |
| 88 | rs7240767 | C | 0.05 | 0.01 | 0.00 | 0.00 |
| 89 | rs72892910 | T | 0.06 | 0.01 | 0.00 | 0.00 |
| 90 | rs735949 | C | -0.07 | 0.01 | 0.00 | 0.00 |
| 91 | rs753270 | C | 0.05 | 0.01 | 0.00 | 0.00 |
| 92 | rs7561798 | G | 0.04 | 0.01 | 0.00 | 0.00 |
| 93 | rs7572970 | G | 0.06 | 0.01 | 0.00 | 0.00 |
| 94 | rs7619041 | A | -0.04 | 0.01 | 0.00 | 0.00 |
| 95 | rs7651090 | G | 0.12 | 0.01 | 0.00 | 0.00 |
| 96 | rs7674212 | T | -0.05 | 0.01 | 0.00 | 0.00 |
| 97 | rs7685296 | T | -0.05 | 0.01 | 0.00 | 0.00 |
| 98 | rs77258096 | A | -0.12 | 0.01 | 0.00 | 0.00 |
| 99 | rs7729395 | T | 0.14 | 0.02 | 0.00 | 0.00 |
| 100 | rs7756992 | G | 0.13 | 0.01 | 0.00 | 0.00 |
| 101 | rs7786095 | G | -0.07 | 0.01 | 0.00 | 0.00 |
| 102 | rs780094 | C | 0.07 | 0.01 | 0.00 | 0.00 |
| 103 | rs7845219 | C | -0.04 | 0.01 | 0.00 | 0.00 |
| 104 | rs7923866 | T | -0.10 | 0.01 | 0.00 | 0.00 |
| 105 | rs7929543 | C | 0.08 | 0.01 | 0.00 | 0.00 |
| 106 | rs8068804 | A | 0.06 | 0.01 | 0.00 | 0.00 |
| 107 | rs8108269 | G | 0.06 | 0.01 | 0.00 | 0.00 |
| 108 | rs825476 | T | 0.05 | 0.01 | 0.00 | 0.00 |
| 109 | rs840967 | A | -0.05 | 0.01 | 0.00 | 0.00 |
| 110 | rs849135 | A | -0.10 | 0.01 | 0.00 | 0.00 |
| 111 | rs853974 | C | -0.06 | 0.01 | 0.00 | 0.00 |
| 112 | rs9369425 | A | -0.05 | 0.01 | 0.00 | 0.00 |
| 113 | rs963740 | T | -0.05 | 0.01 | 0.00 | 0.00 |
| 114 | rs982077 | G | -0.05 | 0.01 | 0.00 | 0.00 |
| 115 | rs9844972 | C | 0.10 | 0.01 | 0.00 | 0.00 |
| 116 | rs9894220 | G | -0.06 | 0.01 | 0.00 | 0.00 |
| 117 | rs993380 | G | -0.05 | 0.01 | 0.00 | 0.00 |
| 118 | rs9940149 | A | -0.06 | 0.01 | 0.00 | 0.00 |

T2DM: Type-2 diabetes

Table 29: Genetic association estimates for the effect of T2DM on insulin sensitivity. ea=effect allele, gx=T2DM, gy=insulin sensitivity, se=standard error

|  | SNP | ea | gx | gx_se | gy | gy_se |
| --- | --- | --- | --- | --- | --- | --- |
| 1 | rs10077431 | A | -0.05 | 0.01 | -0.06 | 0.10 |
| 2 | rs10100265 | C | -0.05 | 0.01 | -0.01 | 0.08 |
| 3 | rs10114341 | C | -0.04 | 0.01 | 0.03 | 0.08 |
| 4 | rs10401969 | C | 0.09 | 0.01 | 0.18 | 0.18 |
| 5 | rs1050226 | G | -0.05 | 0.01 | -0.06 | 0.07 |
| 6 | rs1061813 | A | -0.04 | 0.01 | -0.05 | 0.08 |
| 7 | rs10740322 | A | 0.05 | 0.01 | -0.07 | 0.08 |
| 8 | rs10811661 | C | -0.16 | 0.01 | 0.04 | 0.11 |
| 9 | rs10830963 | G | 0.09 | 0.01 | -0.05 | 0.09 |
| 10 | rs10842994 | T | -0.08 | 0.01 | 0.05 | 0.10 |
| 11 | rs10974438 | C | 0.06 | 0.01 | -0.26 | 0.08 |
| 12 | rs11098676 | C | 0.05 | 0.01 | 0.08 | 0.10 |
| 13 | rs11107116 | T | 0.05 | 0.01 | 0.15 | 0.09 |
| 14 | rs11257655 | T | 0.07 | 0.01 | -0.01 | 0.09 |
| 15 | rs1127655 | T | -0.04 | 0.01 | 0.04 | 0.07 |
| 16 | rs11708067 | G | -0.10 | 0.01 | 0.01 | 0.10 |
| 17 | rs11925227 | A | -0.05 | 0.01 | -0.05 | 0.10 |
| 18 | rs11926707 | C | 0.05 | 0.01 | 0.00 | 0.08 |
| 19 | rs12088739 | G | -0.09 | 0.01 | -0.06 | 0.14 |
| 20 | rs12299509 | G | 0.05 | 0.01 | 0.09 | 0.08 |
| 21 | rs12617659 | T | -0.07 | 0.01 | 0.15 | 0.10 |
| 22 | rs12910825 | G | 0.05 | 0.01 | -0.05 | 0.08 |
| 23 | rs12945601 | C | -0.05 | 0.01 | 0.02 | 0.08 |
| 24 | rs12970134 | A | 0.06 | 0.01 | -0.11 | 0.09 |
| 25 | rs13234269 | A | -0.06 | 0.01 | -0.11 | 0.08 |
| 26 | rs13239186 | T | 0.05 | 0.01 | -0.01 | 0.08 |
| 27 | rs1333039 | C | 0.05 | 0.01 | -0.05 | 0.09 |
| 28 | rs13330951 | G | -0.05 | 0.01 | 0.09 | 0.07 |
| 29 | rs13389219 | T | -0.07 | 0.01 | 0.00 | 0.08 |
| 30 | rs1359790 | A | -0.08 | 0.01 | 0.07 | 0.08 |
| 31 | rs1496653 | G | -0.08 | 0.01 | 0.06 | 0.09 |
| 32 | rs1552224 | C | -0.10 | 0.01 | -0.11 | 0.10 |
| 33 | rs16988333 | G | -0.07 | 0.01 | -0.21 | 0.14 |
| 34 | rs17086692 | T | -0.05 | 0.01 | 0.17 | 0.08 |
| 35 | rs17168486 | T | 0.07 | 0.01 | 0.03 | 0.10 |
| 36 | rs17334919 | T | -0.14 | 0.01 | 0.07 | 0.14 |
| 37 | rs17405722 | A | 0.09 | 0.01 | 0.09 | 0.14 |
| 38 | rs17411031 | G | -0.05 | 0.01 | -0.10 | 0.09 |
| 39 | rs1758632 | G | 0.05 | 0.01 | -0.10 | 0.08 |
| 40 | rs17631783 | T | -0.05 | 0.01 | 0.03 | 0.09 |
| 41 | rs17791483 | G | -0.10 | 0.01 | -0.08 | 0.13 |
| 42 | rs1801214 | T | 0.09 | 0.01 | -0.16 | 0.08 |
| 43 | rs1899951 | T | -0.11 | 0.01 | -0.17 | 0.10 |
| 44 | rs2191348 | T | 0.07 | 0.01 | -0.14 | 0.07 |
| 45 | rs2237892 | T | -0.10 | 0.02 | 0.14 | 0.16 |
| 46 | rs2246618 | T | 0.05 | 0.01 | 0.08 | 0.08 |
| 47 | rs2261181 | T | 0.10 | 0.01 | -0.07 | 0.13 |
| 48 | rs2292662 | T | -0.06 | 0.01 | -0.05 | 0.10 |
| 49 | rs2294120 | G | -0.04 | 0.01 | -0.14 | 0.08 |
| 50 | rs2296173 | G | 0.07 | 0.01 | 0.06 | 0.10 |
| 51 | rs2299383 | T | 0.04 | 0.01 | -0.06 | 0.07 |
| 52 | rs243019 | C | 0.06 | 0.01 | 0.01 | 0.07 |
| 53 | rs2493394 | G | 0.07 | 0.01 | -0.47 | 0.11 |
| 54 | rs2796441 | A | -0.07 | 0.01 | 0.08 | 0.09 |
| 55 | rs2820426 | G | 0.05 | 0.01 | 0.04 | 0.08 |
| 56 | rs2867125 | C | 0.06 | 0.01 | 0.14 | 0.10 |
| 57 | rs2908282 | A | 0.06 | 0.01 | -0.04 | 0.11 |
| 58 | rs2925979 | C | -0.05 | 0.01 | -0.06 | 0.09 |
| 59 | rs2972144 | G | 0.09 | 0.01 | 0.14 | 0.07 |
| 60 | rs340874 | C | 0.06 | 0.01 | 0.04 | 0.08 |
| 61 | rs3756784 | G | 0.05 | 0.01 | -0.05 | 0.10 |
| 62 | rs3802177 | A | -0.12 | 0.01 | 0.01 | 0.09 |
| 63 | rs4506565 | T | 0.28 | 0.01 | -0.22 | 0.08 |
| 64 | rs459193 | G | 0.07 | 0.01 | 0.08 | 0.08 |
| 65 | rs4823182 | G | 0.05 | 0.01 | 0.05 | 0.08 |
| 66 | rs4865796 | A | 0.05 | 0.01 | 0.07 | 0.08 |
| 67 | rs516946 | C | 0.08 | 0.01 | -0.10 | 0.09 |
| 68 | rs5215 | T | -0.07 | 0.01 | 0.05 | 0.08 |
| 69 | rs576674 | A | -0.07 | 0.01 | 0.18 | 0.12 |
| 70 | rs6059662 | G | 0.04 | 0.01 | -0.08 | 0.09 |
| 71 | rs6066138 | A | -0.05 | 0.01 | -0.16 | 0.08 |
| 72 | rs622217 | C | -0.05 | 0.01 | 0.06 | 0.08 |
| 73 | rs6494307 | G | -0.04 | 0.01 | 0.08 | 0.08 |
| 74 | rs6515236 | C | -0.05 | 0.01 | 0.12 | 0.09 |
| 75 | rs6795735 | T | -0.06 | 0.01 | -0.02 | 0.07 |
| 76 | rs6878122 | A | -0.06 | 0.01 | 0.01 | 0.09 |
| 77 | rs7138300 | T | -0.04 | 0.01 | 0.08 | 0.08 |
| 78 | rs7144011 | T | 0.05 | 0.01 | -0.02 | 0.09 |
| 79 | rs7177055 | A | 0.06 | 0.01 | -0.13 | 0.08 |
| 80 | rs7185735 | G | 0.11 | 0.01 | -0.03 | 0.08 |
| 81 | rs7240767 | C | 0.05 | 0.01 | -0.01 | 0.08 |
| 82 | rs72892910 | T | 0.06 | 0.01 | 0.07 | 0.10 |
| 83 | rs735949 | C | -0.07 | 0.01 | 0.11 | 0.11 |
| 84 | rs753270 | C | 0.05 | 0.01 | 0.09 | 0.08 |
| 85 | rs7561798 | G | 0.04 | 0.01 | -0.06 | 0.07 |
| 86 | rs7572970 | G | 0.06 | 0.01 | 0.15 | 0.09 |
| 87 | rs7619041 | A | -0.04 | 0.01 | -0.02 | 0.08 |
| 88 | rs7651090 | G | 0.12 | 0.01 | -0.11 | 0.08 |
| 89 | rs7674212 | T | -0.05 | 0.01 | -0.03 | 0.08 |
| 90 | rs7685296 | T | -0.05 | 0.01 | -0.03 | 0.09 |
| 91 | rs77258096 | A | -0.12 | 0.01 | 0.07 | 0.11 |
| 92 | rs7729395 | T | 0.14 | 0.02 | -0.12 | 0.19 |
| 93 | rs7756992 | G | 0.13 | 0.01 | -0.04 | 0.08 |
| 94 | rs7786095 | G | -0.07 | 0.01 | -0.02 | 0.14 |
| 95 | rs780094 | C | 0.07 | 0.01 | -0.14 | 0.07 |
| 96 | rs7845219 | C | -0.04 | 0.01 | 0.03 | 0.08 |
| 97 | rs7923866 | T | -0.10 | 0.01 | -0.02 | 0.08 |
| 98 | rs7929543 | C | 0.08 | 0.01 | 0.07 | 0.16 |
| 99 | rs8068804 | A | 0.06 | 0.01 | -0.03 | 0.08 |
| 100 | rs8108269 | G | 0.06 | 0.01 | 0.01 | 0.09 |
| 101 | rs825476 | T | 0.05 | 0.01 | 0.02 | 0.08 |
| 102 | rs840967 | A | -0.05 | 0.01 | 0.07 | 0.08 |
| 103 | rs849135 | A | -0.10 | 0.01 | 0.06 | 0.08 |
| 104 | rs9369425 | A | -0.05 | 0.01 | 0.14 | 0.08 |
| 105 | rs963740 | T | -0.05 | 0.01 | 0.04 | 0.09 |
| 106 | rs982077 | G | -0.05 | 0.01 | -0.03 | 0.08 |
| 107 | rs9844972 | C | 0.10 | 0.01 | -0.05 | 0.16 |
| 108 | rs9894220 | G | -0.06 | 0.01 | -0.06 | 0.08 |
| 109 | rs993380 | G | -0.05 | 0.01 | -0.09 | 0.08 |
| 110 | rs9940149 | A | -0.06 | 0.01 | 0.11 | 0.10 |

T2DM: Type-2 diabetes

Table 30: MR estimates of T2DM on each risk factor.

|  | MR Method | OR | 95%CI | P-value |
| --- | --- | --- | --- | --- |
| SBP | Simple median | 2.01 | 1.65-2.45 | <0.001 |
|  | Weighted median | 2.02 | 1.68-2.44 | <0.001 |
|  | MR Egger | 2.01 | 1.04-3.86 | 0.039 |
|  | Inverse variance weighted | 2.17 | 1.64-2.86 | <0.001 |
| DBP | Simple median | 1.09 | 0.98-1.22 | 0.117 |
|  | Weighted median | 1.08 | 0.96-1.21 | 0.187 |
|  | MR Egger | 1.08 | 0.73-1.59 | 0.694 |
|  | Inverse variance weighted | 1.25 | 1.06-1.47 | 0.009 |
| TG | Simple median | 1.07 | 1.06-1.09 | <0.001 |
|  | Weighted median | 1.05 | 1.03-1.06 | <0.001 |
|  | MR Egger | 1 | 0.89-1.13 | 0.977 |
|  | Inverse variance weighted | 1.08 | 1.03-1.13 | 0.002 |
| HDL | Simple median | 0.83 | 0.76-0.90 | <0.001 |
|  | Weighted median | 0.89 | 0.82-0.96 | 0.005 |
|  | MR Egger | 1 | 0.88-1.13 | 0.940 |
|  | Inverse variance weighted | 0.86 | 0.82-0.91 | <0.001 |
| LDL | Simple median | 0.9 | 0.84-0.96 | 0.003 |
|  | Weighted median | 0.9 | 0.83-0.97 | 0.004 |
|  | MR Egger | 0.91 | 0.80-1.04 | 0.161 |
|  | Inverse variance weighted | 0.93 | 0.89-0.98 | 0.008 |
| TC | Simple median | 0.9 | 0.83-0.98 | 0.011 |
|  | Weighted median | 0.9 | 0.83-0.97 | 0.010 |
|  | MR Egger | 0.91 | 0.80-1.04 | 0.175 |
|  | Inverse variance weighted | 0.94 | 0.89-0.99 | 0.025 |
| WHR | Simple median | 1.04 | 1.02-1.06 | <0.001 |
|  | Weighted median | 1.03 | 1.01-1.05 | <0.001 |
|  | MR Egger | 1.02 | 0.97-1.08 | 0.402 |
|  | Inverse variance weighted | 1.05 | 1.03-0.08 | <0.001 |
| Insulin sensitivity | Simple median | 0.80 | 0.56-1.16 | 0.225 |
|  | Weighted median | 0.59 | 0.42-0.84 | 0.002 |
|  | MR Egger | 0.53 | 0.30-0.91 | 0.022 |
|  | Inverse variance weighted | 0.74 | 0.58-0.93 | 0.012 |
| BMI | Simple median | 1.02 | 0.98-1.05 | 0.404 |
|  | Weighted median | 1.00 | 0.96-1.03 | 0.780 |
|  | MR Egger | 0.96 | 0.92-1.01 | 0.087 |
|  | Inverse variance weighted | 1.01 | 0.99-1.02 | 0.615 |
| VLDL | Simple median | 1.04 | 0.99-1.09 | 0.105 |
|  | Weighted median | 1.02 | 0.97-1.08 | 0.381 |
|  | MR Egger | 1.00 | 0.92-1.08 | 0.998 |
|  | Inverse variance weighted | 1.02 | 0.99-1.06 | 0.168 |
| Hyperthyroidism | Simple median | 1.00 | 1.00-1.00 | 0.431 |
|  | Weighted median | 1.00 | 1.00-1.00 | 0.619 |
|  | MR Egger | 1.00 | 1.00-1.00 | 0.708 |
|  | Inverse variance weighted | 1.00 | 1.00-1.00 | 0.213 |
| Hypothyroidism | Simple median | 1.00 | 1.00-1.00 | 0.820 |
|  | Weighted median | 1.00 | 1.00-1.00 | 0.618 |
|  | MR Egger | 1.00 | 1.00-1.01 | 0.976 |
|  | Inverse variance weighted | 1.00 | 1.00-1.01 | 0.159 |

SBP: Systolic blood pressure; DBP: Diastolic blood pressure; TG: Triglycerides; HDL: High-density lipoprotein; LDL: Low-density lipoprotein; TC: Total cholesterol; WHR: Waist-hip-ratio; BMI: Body mass index; VLDL: Very low-density lipoprotein; OR: Odds ratio; CI: Confidence interval;

Table 31: Genetic association estimates for the effect of SBP on CHD, adjusted for T2DM. ea=effect allele, gx=SBP, gy=CHD, gz=T2DM, se=standard error

|  | SNP | ea | gx | gx_se | gy | gy_se | gz | gz_se |
| --- | --- | --- | --- | --- | --- | --- | --- | --- |
| 1 | rs1000423 | T | 0.41 | 0.03 | 0.04 | 0.01 | 0.00 | 0.01 |
| 2 | rs10008637 | C | -0.22 | 0.03 | 0.00 | 0.01 | 0.00 | 0.01 |
| 3 | rs10045307 | G | 0.20 | 0.04 | -0.01 | 0.01 | -0.01 | 0.01 |
| 4 | rs10048404 | T | -0.26 | 0.03 | -0.02 | 0.01 | 0.02 | 0.01 |
| 5 | rs1006545 | T | 0.68 | 0.05 | -0.02 | 0.02 | 0.03 | 0.01 |
| 6 | rs1010064 | C | -0.36 | 0.04 | -0.04 | 0.01 | 0.02 | 0.01 |
| 7 | rs1012089 | G | 0.19 | 0.03 | 0.00 | 0.01 | 0.01 | 0.01 |
| 8 | rs10188003 | T | 0.19 | 0.03 | -0.01 | 0.01 | 0.00 | 0.01 |
| 9 | rs10207726 | T | -0.21 | 0.03 | -0.02 | 0.01 | 0.02 | 0.01 |
| 10 | rs10224210 | C | 0.38 | 0.03 | 0.01 | 0.01 | 0.02 | 0.01 |
| 11 | rs1044822 | T | -0.25 | 0.04 | -0.02 | 0.01 | 0.02 | 0.01 |
| 12 | rs10460108 | G | -0.21 | 0.03 | 0.01 | 0.01 | 0.00 | 0.01 |
| 13 | rs1049212 | G | 0.30 | 0.03 | 0.01 | 0.01 | 0.01 | 0.01 |
| 14 | rs10501410 | A | 0.41 | 0.06 | -0.03 | 0.02 | -0.01 | 0.02 |
| 15 | rs1052501 | T | 0.23 | 0.04 | -0.01 | 0.01 | 0.01 | 0.01 |
| 16 | rs10749572 | T | -0.20 | 0.03 | -0.02 | 0.01 | 0.00 | 0.01 |
| 17 | rs10750441 | T | 0.18 | 0.03 | 0.00 | 0.01 | -0.01 | 0.01 |
| 18 | rs10777213 | A | -0.18 | 0.03 | 0.00 | 0.01 | 0.01 | 0.01 |
| 19 | rs10779795 | G | -0.22 | 0.03 | -0.01 | 0.01 | 0.01 | 0.01 |
| 20 | rs10782230 | A | 0.21 | 0.03 | 0.00 | 0.01 | -0.01 | 0.01 |
| 21 | rs10804330 | C | -0.24 | 0.03 | -0.02 | 0.01 | 0.07 | 0.01 |
| 22 | rs10914124 | C | -0.23 | 0.03 | -0.01 | 0.01 | 0.00 | 0.01 |
| 23 | rs10941043 | G | 0.26 | 0.03 | 0.00 | 0.01 | -0.01 | 0.01 |
| 24 | rs10980408 | C | 0.76 | 0.08 | 0.04 | 0.03 | -0.03 | 0.02 |
| 25 | rs11097909 | C | 0.36 | 0.04 | 0.01 | 0.01 | 0.00 | 0.01 |
| 26 | rs11120093 | T | -0.18 | 0.03 | -0.01 | 0.01 | -0.01 | 0.01 |
| 27 | rs11159091 | A | 0.20 | 0.03 | 0.00 | 0.01 | 0.01 | 0.01 |
| 28 | rs111866816 | T | 0.36 | 0.06 | 0.02 | 0.02 | -0.02 | 0.02 |
| 29 | rs11191580 | C | -1.10 | 0.06 | -0.07 | 0.01 | 0.00 | 0.01 |
| 30 | rs11210029 | G | 0.20 | 0.03 | 0.01 | 0.01 | 0.01 | 0.01 |
| 31 | rs11222084 | T | 0.34 | 0.03 | 0.00 | 0.01 | -0.02 | 0.01 |
| 32 | rs11241313 | T | -0.21 | 0.03 | -0.01 | 0.01 | 0.00 | 0.01 |
| 33 | rs112509803 | C | -0.26 | 0.05 | 0.00 | 0.02 | 0.01 | 0.01 |
| 34 | rs11252324 | T | -0.42 | 0.06 | -0.06 | 0.02 | -0.01 | 0.01 |
| 35 | rs115262049 | T | -0.59 | 0.06 | -0.03 | 0.02 | 0.02 | 0.01 |
| 36 | rs1154214 | G | 0.20 | 0.03 | 0.01 | 0.01 | 0.00 | 0.01 |
| 37 | rs11585169 | A | 0.18 | 0.03 | 0.03 | 0.01 | -0.02 | 0.01 |
| 38 | rs11592107 | A | 0.30 | 0.03 | 0.02 | 0.01 | -0.03 | 0.01 |
| 39 | rs11604357 | A | -0.28 | 0.04 | 0.01 | 0.01 | 0.00 | 0.01 |
| 40 | rs11636952 | C | -0.53 | 0.03 | -0.02 | 0.01 | 0.01 | 0.01 |
| 41 | rs1169078 | G | 0.20 | 0.03 | 0.01 | 0.01 | 0.00 | 0.01 |
| 42 | rs11694601 | G | 0.19 | 0.03 | 0.00 | 0.01 | -0.01 | 0.01 |
| 43 | rs11834380 | A | -0.28 | 0.05 | -0.01 | 0.02 | 0.05 | 0.01 |
| 44 | rs11874246 | T | 0.29 | 0.03 | 0.02 | 0.01 | -0.01 | 0.01 |
| 45 | rs11925504 | A | -0.29 | 0.03 | -0.04 | 0.01 | 0.03 | 0.01 |
| 46 | rs11960210 | C | -0.47 | 0.03 | -0.02 | 0.01 | 0.02 | 0.01 |
| 47 | rs11977526 | A | -0.32 | 0.03 | 0.03 | 0.01 | 0.01 | 0.01 |
| 48 | rs1199330 | G | 0.27 | 0.05 | 0.08 | 0.01 | -0.03 | 0.01 |
| 49 | rs12042924 | C | 0.18 | 0.03 | 0.00 | 0.01 | -0.02 | 0.01 |
| 50 | rs1209384 | G | -0.26 | 0.03 | -0.02 | 0.01 | -0.01 | 0.01 |
| 51 | rs12136922 | A | 0.20 | 0.03 | -0.02 | 0.01 | -0.01 | 0.01 |
| 52 | rs12255372 | T | 0.24 | 0.03 | 0.03 | 0.01 | -0.26 | 0.01 |
| 53 | rs12258967 | G | -0.63 | 0.03 | -0.03 | 0.01 | -0.02 | 0.01 |
| 54 | rs12264186 | T | 0.21 | 0.04 | 0.01 | 0.01 | -0.01 | 0.01 |
| 55 | rs12321 | C | -0.23 | 0.03 | -0.02 | 0.01 | -0.01 | 0.01 |
| 56 | rs12426261 | G | -0.38 | 0.03 | 0.00 | 0.01 | 0.00 | 0.01 |
| 57 | rs12464602 | A | -0.24 | 0.03 | -0.01 | 0.01 | 0.00 | 0.01 |
| 58 | rs12509595 | C | 0.84 | 0.03 | 0.05 | 0.01 | -0.02 | 0.01 |
| 59 | rs12511987 | G | 0.23 | 0.04 | 0.02 | 0.01 | -0.01 | 0.01 |
| 60 | rs12610654 | G | -0.23 | 0.03 | -0.01 | 0.01 | 0.00 | 0.01 |
| 61 | rs12637573 | G | 0.17 | 0.03 | 0.00 | 0.01 | -0.01 | 0.01 |
| 62 | rs12643599 | G | -0.31 | 0.03 | -0.05 | 0.01 | 0.02 | 0.01 |
| 63 | rs12656497 | C | 0.64 | 0.03 | 0.01 | 0.01 | 0.00 | 0.01 |
| 64 | rs12657950 | T | 0.46 | 0.06 | 0.02 | 0.02 | 0.01 | 0.02 |
| 65 | rs12661036 | C | 0.21 | 0.04 | 0.02 | 0.01 | -0.02 | 0.01 |
| 66 | rs12668436 | C | 0.22 | 0.04 | 0.01 | 0.01 | 0.00 | 0.01 |
| 67 | rs12693982 | T | 0.26 | 0.03 | 0.04 | 0.01 | 0.02 | 0.01 |
| 68 | rs12731646 | T | -0.19 | 0.03 | -0.02 | 0.01 | 0.01 | 0.01 |
| 69 | rs1275985 | T | -0.54 | 0.03 | 0.00 | 0.01 | -0.01 | 0.01 |
| 70 | rs12883810 | T | -0.24 | 0.04 | -0.02 | 0.01 | -0.02 | 0.01 |
| 71 | rs12906962 | C | 0.27 | 0.03 | 0.02 | 0.01 | -0.01 | 0.01 |
| 72 | rs1290784 | T | 0.41 | 0.03 | 0.01 | 0.01 | -0.01 | 0.01 |
| 73 | rs1290933 | A | -0.28 | 0.03 | 0.00 | 0.01 | 0.02 | 0.01 |
| 74 | rs12926550 | A | -0.25 | 0.03 | -0.03 | 0.01 | 0.02 | 0.01 |
| 75 | rs1293969 | C | 0.20 | 0.03 | -0.01 | 0.01 | 0.02 | 0.01 |
| 76 | rs13016772 | T | 0.25 | 0.04 | 0.01 | 0.01 | -0.02 | 0.01 |
| 77 | rs13091418 | G | 0.22 | 0.03 | 0.00 | 0.01 | -0.02 | 0.01 |
| 78 | rs13107261 | A | -0.18 | 0.03 | 0.00 | 0.01 | 0.01 | 0.01 |
| 79 | rs13204703 | C | -0.20 | 0.04 | -0.02 | 0.01 | 0.01 | 0.01 |
| 80 | rs13253358 | T | 0.21 | 0.03 | 0.01 | 0.01 | 0.00 | 0.01 |
| 81 | rs1332813 | C | -0.22 | 0.03 | 0.01 | 0.01 | 0.00 | 0.01 |
| 82 | rs13358657 | G | 0.39 | 0.04 | 0.01 | 0.01 | -0.01 | 0.01 |
| 83 | rs1340030 | C | -0.19 | 0.03 | 0.01 | 0.01 | 0.02 | 0.01 |
| 84 | rs13412750 | A | -0.29 | 0.03 | 0.00 | 0.01 | -0.01 | 0.01 |
| 85 | rs13420463 | G | -0.31 | 0.04 | -0.01 | 0.01 | 0.01 | 0.01 |
| 86 | rs1375564 | T | 0.26 | 0.03 | 0.00 | 0.01 | 0.01 | 0.01 |
| 87 | rs1382472 | A | -0.19 | 0.03 | 0.02 | 0.01 | 0.00 | 0.01 |
| 88 | rs1408945 | T | -0.32 | 0.03 | 0.02 | 0.01 | 0.00 | 0.01 |
| 89 | rs1410222 | T | 0.22 | 0.04 | -0.01 | 0.01 | 0.01 | 0.01 |
| 90 | rs1422279 | T | 0.33 | 0.03 | 0.01 | 0.01 | 0.00 | 0.01 |
| 91 | rs1433121 | T | -0.23 | 0.03 | -0.01 | 0.01 | -0.02 | 0.01 |
| 92 | rs1437649 | A | -0.22 | 0.04 | -0.01 | 0.01 | 0.01 | 0.01 |
| 93 | rs146550789 | C | 0.48 | 0.08 | 0.00 | 0.03 | 0.04 | 0.02 |
| 94 | rs148140538 | T | -0.33 | 0.06 | 0.01 | 0.02 | 0.00 | 0.01 |
| 95 | rs1493132 | C | 0.18 | 0.03 | 0.00 | 0.01 | -0.01 | 0.01 |
| 96 | rs1544861 | C | -0.20 | 0.03 | 0.01 | 0.01 | -0.02 | 0.01 |
| 97 | rs1551355 | T | 0.21 | 0.04 | 0.03 | 0.01 | 0.00 | 0.01 |
| 98 | rs1565440 | A | 0.17 | 0.03 | 0.01 | 0.01 | -0.01 | 0.01 |
| 99 | rs1575290 | T | 0.20 | 0.03 | -0.02 | 0.01 | 0.00 | 0.01 |
| 100 | rs1623474 | T | 0.38 | 0.03 | 0.01 | 0.01 | 0.00 | 0.01 |
| 101 | rs1630736 | T | -0.17 | 0.03 | 0.02 | 0.01 | 0.01 | 0.01 |
| 102 | rs1664781 | A | 0.26 | 0.03 | 0.01 | 0.01 | -0.06 | 0.01 |
| 103 | rs17010957 | C | 0.53 | 0.04 | 0.01 | 0.01 | 0.01 | 0.01 |
| 104 | rs17035181 | G | -0.31 | 0.04 | -0.02 | 0.01 | 0.04 | 0.01 |
| 105 | rs17080102 | C | -0.81 | 0.06 | -0.05 | 0.02 | 0.00 | 0.02 |
| 106 | rs17245822 | C | 0.19 | 0.03 | 0.00 | 0.01 | -0.02 | 0.01 |
| 107 | rs17249754 | A | -0.84 | 0.04 | 0.07 | 0.01 | -0.01 | 0.01 |
| 108 | rs17257081 | G | -0.23 | 0.04 | 0.01 | 0.01 | -0.02 | 0.01 |
| 109 | rs17608766 | C | 0.69 | 0.04 | 0.05 | 0.02 | 0.00 | 0.01 |
| 110 | rs177551 | A | 0.37 | 0.04 | 0.02 | 0.01 | 0.00 | 0.01 |
| 111 | rs17760259 | C | 0.27 | 0.03 | 0.02 | 0.01 | -0.01 | 0.01 |
| 112 | rs17762 | A | 0.41 | 0.06 | 0.01 | 0.01 | -0.02 | 0.01 |
| 113 | rs17807723 | A | -0.27 | 0.04 | -0.02 | 0.02 | -0.01 | 0.01 |
| 114 | rs17812022 | T | -0.36 | 0.05 | 0.02 | 0.02 | 0.00 | 0.01 |
| 115 | rs1786345 | C | -0.21 | 0.03 | -0.01 | 0.01 | 0.01 | 0.01 |
| 116 | rs1814951 | A | -0.32 | 0.05 | -0.01 | 0.01 | 0.00 | 0.01 |
| 117 | rs1821002 | G | -0.38 | 0.03 | -0.02 | 0.01 | 0.04 | 0.01 |
| 118 | rs1848994 | A | 0.20 | 0.03 | -0.01 | 0.01 | 0.02 | 0.01 |
| 119 | rs1870735 | G | -0.21 | 0.03 | 0.00 | 0.01 | 0.00 | 0.01 |
| 120 | rs1871190 | T | 0.20 | 0.03 | 0.03 | 0.01 | -0.01 | 0.01 |
| 121 | rs1882212 | G | -0.28 | 0.04 | -0.01 | 0.01 | 0.01 | 0.01 |
| 122 | rs1882961 | T | 0.24 | 0.03 | 0.04 | 0.01 | 0.01 | 0.01 |
| 123 | rs1889785 | A | 0.18 | 0.03 | 0.00 | 0.01 | -0.01 | 0.01 |
| 124 | rs1906672 | A | 0.30 | 0.04 | 0.00 | 0.01 | -0.01 | 0.01 |
| 125 | rs1957563 | T | 0.36 | 0.03 | 0.02 | 0.01 | -0.01 | 0.01 |
| 126 | rs1984195 | A | 0.24 | 0.03 | 0.03 | 0.01 | -0.01 | 0.01 |
| 127 | rs1994158 | G | -0.25 | 0.04 | 0.00 | 0.01 | -0.01 | 0.01 |
| 128 | rs2014408 | T | 0.52 | 0.04 | 0.01 | 0.01 | -0.02 | 0.01 |
| 129 | rs2024385 | A | -0.26 | 0.03 | 0.00 | 0.01 | -0.01 | 0.01 |
| 130 | rs2111557 | T | 0.18 | 0.03 | 0.00 | 0.01 | -0.01 | 0.01 |
| 131 | rs2126474 | T | -0.26 | 0.03 | 0.00 | 0.01 | 0.01 | 0.01 |
| 132 | rs2129869 | T | 0.26 | 0.04 | 0.00 | 0.01 | -0.05 | 0.01 |
| 133 | rs2161967 | G | -0.28 | 0.03 | -0.04 | 0.01 | 0.00 | 0.01 |
| 134 | rs2177843 | T | 0.44 | 0.04 | 0.00 | 0.01 | 0.03 | 0.01 |
| 135 | rs2236295 | T | -0.30 | 0.03 | 0.01 | 0.01 | 0.03 | 0.01 |
| 136 | rs2249105 | G | -0.29 | 0.03 | 0.00 | 0.01 | 0.05 | 0.01 |
| 137 | rs2276153 | G | -0.33 | 0.04 | 0.01 | 0.01 | 0.04 | 0.01 |
| 138 | rs2283500 | C | -0.31 | 0.05 | -0.03 | 0.02 | 0.01 | 0.01 |
| 139 | rs2291434 | T | -0.26 | 0.03 | 0.00 | 0.01 | 0.03 | 0.01 |
| 140 | rs2353940 | C | 0.21 | 0.04 | 0.01 | 0.01 | -0.01 | 0.01 |
| 141 | rs2354862 | C | -0.25 | 0.03 | 0.01 | 0.01 | 0.01 | 0.01 |
| 142 | rs2384063 | T | 0.33 | 0.04 | 0.00 | 0.01 | 0.00 | 0.01 |
| 143 | rs2392929 | G | 0.75 | 0.04 | 0.01 | 0.01 | 0.01 | 0.01 |
| 144 | rs2423514 | G | -0.30 | 0.03 | -0.02 | 0.01 | 0.01 | 0.01 |
| 145 | rs246973 | T | 0.25 | 0.03 | 0.01 | 0.01 | 0.00 | 0.01 |
| 146 | rs2470004 | T | -0.35 | 0.04 | 0.00 | 0.01 | 0.02 | 0.01 |
| 147 | rs2493134 | C | 0.37 | 0.03 | 0.02 | 0.01 | 0.00 | 0.01 |
| 148 | rs2498323 | A | 0.32 | 0.05 | 0.05 | 0.02 | -0.02 | 0.01 |
| 149 | rs2580350 | A | 0.18 | 0.03 | 0.00 | 0.01 | -0.02 | 0.01 |
| 150 | rs2589218 | C | 0.23 | 0.03 | 0.02 | 0.01 | -0.01 | 0.01 |
| 151 | rs2598 | G | -0.17 | 0.03 | 0.02 | 0.01 | -0.01 | 0.01 |
| 152 | rs2608029 | G | -0.18 | 0.03 | 0.00 | 0.01 | 0.01 | 0.01 |
| 153 | rs2610990 | G | 0.29 | 0.03 | 0.03 | 0.01 | -0.03 | 0.01 |
| 154 | rs2627313 | T | 0.32 | 0.03 | 0.01 | 0.01 | -0.02 | 0.01 |
| 155 | rs262986 | A | -0.24 | 0.03 | 0.00 | 0.01 | 0.01 | 0.01 |
| 156 | rs2643826 | T | 0.45 | 0.03 | 0.00 | 0.01 | -0.02 | 0.01 |
| 157 | rs2652812 | T | -0.25 | 0.04 | -0.02 | 0.01 | 0.02 | 0.01 |
| 158 | rs268263 | A | 0.59 | 0.04 | 0.04 | 0.01 | 0.01 | 0.01 |
| 159 | rs2689690 | T | -0.27 | 0.03 | 0.00 | 0.01 | 0.02 | 0.01 |
| 160 | rs2724377 | G | -0.19 | 0.03 | 0.00 | 0.01 | 0.00 | 0.01 |
| 161 | rs2744139 | C | -0.24 | 0.04 | -0.01 | 0.01 | 0.00 | 0.01 |
| 162 | rs2760748 | A | 0.36 | 0.05 | 0.03 | 0.02 | -0.01 | 0.01 |
| 163 | rs2776037 | C | 0.19 | 0.03 | 0.00 | 0.01 | 0.01 | 0.01 |
| 164 | rs2801008 | G | 0.19 | 0.03 | 0.00 | 0.01 | 0.00 | 0.01 |
| 165 | rs2833834 | A | 0.22 | 0.03 | 0.00 | 0.01 | -0.01 | 0.01 |
| 166 | rs2853736 | G | -0.24 | 0.03 | 0.01 | 0.01 | -0.02 | 0.01 |
| 167 | rs28572357 | C | 0.27 | 0.03 | 0.01 | 0.01 | 0.03 | 0.01 |
| 168 | rs28650790 | T | 0.23 | 0.04 | 0.05 | 0.01 | -0.08 | 0.01 |
| 169 | rs28688791 | C | 0.32 | 0.04 | 0.07 | 0.01 | -0.01 | 0.01 |
| 170 | rs28866311 | G | 0.28 | 0.03 | 0.01 | 0.01 | 0.00 | 0.01 |
| 171 | rs2904315 | G | 0.21 | 0.03 | 0.02 | 0.01 | 0.02 | 0.01 |
| 172 | rs2913920 | T | 0.24 | 0.04 | -0.02 | 0.01 | -0.01 | 0.01 |
| 173 | rs2957688 | A | 0.35 | 0.03 | 0.00 | 0.01 | -0.01 | 0.01 |
| 174 | rs3098186 | T | -0.24 | 0.03 | 0.00 | 0.01 | 0.01 | 0.01 |
| 175 | rs3104552 | C | -0.24 | 0.03 | 0.02 | 0.01 | 0.00 | 0.01 |
| 176 | rs34025993 | G | -0.22 | 0.03 | 0.00 | 0.01 | -0.01 | 0.01 |
| 177 | rs34072724 | A | -0.24 | 0.03 | -0.02 | 0.01 | 0.04 | 0.01 |
| 178 | rs34413141 | A | -0.35 | 0.04 | -0.03 | 0.01 | 0.01 | 0.01 |
| 179 | rs34727427 | C | 0.24 | 0.03 | -0.01 | 0.01 | 0.00 | 0.01 |
| 180 | rs34917849 | C | 0.31 | 0.05 | 0.05 | 0.01 | -0.03 | 0.01 |
| 181 | rs34941092 | A | -0.32 | 0.04 | 0.00 | 0.01 | 0.01 | 0.01 |
| 182 | rs35098810 | C | -0.20 | 0.04 | -0.01 | 0.01 | 0.00 | 0.01 |
| 183 | rs35413927 | G | 0.30 | 0.03 | 0.01 | 0.01 | 0.00 | 0.01 |
| 184 | rs35444 | G | -0.44 | 0.03 | 0.00 | 0.01 | 0.00 | 0.01 |
| 185 | rs35680304 | T | 0.27 | 0.03 | 0.02 | 0.01 | 0.00 | 0.01 |
| 186 | rs35783704 | A | -0.46 | 0.05 | -0.02 | 0.02 | 0.01 | 0.01 |
| 187 | rs3735533 | C | 0.91 | 0.06 | 0.00 | 0.02 | 0.03 | 0.01 |
| 188 | rs3764400 | C | -0.37 | 0.04 | 0.00 | 0.01 | 0.03 | 0.01 |
| 189 | rs3772219 | C | -0.27 | 0.03 | -0.02 | 0.01 | 0.00 | 0.01 |
| 190 | rs3802517 | A | 0.25 | 0.03 | 0.00 | 0.01 | -0.01 | 0.01 |
| 191 | rs3807925 | G | 0.19 | 0.03 | 0.00 | 0.01 | 0.00 | 0.01 |
| 192 | rs3815460 | G | 0.29 | 0.05 | -0.01 | 0.02 | -0.01 | 0.01 |
| 193 | rs3819532 | C | 0.19 | 0.03 | 0.02 | 0.01 | -0.01 | 0.01 |
| 194 | rs3845811 | G | 0.29 | 0.03 | -0.01 | 0.01 | 0.00 | 0.01 |
| 195 | rs3860770 | A | -0.27 | 0.03 | -0.01 | 0.01 | -0.01 | 0.01 |
| 196 | rs3980686 | T | -0.50 | 0.05 | -0.02 | 0.01 | 0.01 | 0.01 |
| 197 | rs404100 | T | 0.19 | 0.03 | 0.01 | 0.01 | 0.01 | 0.01 |
| 198 | rs4143175 | C | -0.22 | 0.04 | 0.00 | 0.01 | 0.00 | 0.01 |
| 199 | rs42032 | A | -0.32 | 0.03 | -0.03 | 0.01 | 0.01 | 0.01 |
| 200 | rs4245599 | G | 0.18 | 0.03 | 0.01 | 0.01 | 0.01 | 0.01 |
| 201 | rs4260863 | G | -0.19 | 0.03 | 0.00 | 0.01 | -0.02 | 0.01 |
| 202 | rs4274337 | G | 0.30 | 0.04 | 0.02 | 0.01 | -0.02 | 0.01 |
| 203 | rs4408839 | G | 0.23 | 0.03 | -0.04 | 0.01 | 0.02 | 0.01 |
| 204 | rs4440615 | A | -0.22 | 0.03 | -0.01 | 0.01 | 0.00 | 0.01 |
| 205 | rs4499560 | T | 0.22 | 0.03 | 0.01 | 0.01 | 0.00 | 0.01 |
| 206 | rs4511593 | T | -0.29 | 0.03 | 0.00 | 0.01 | -0.01 | 0.01 |
| 207 | rs4553000 | T | -0.20 | 0.03 | -0.02 | 0.01 | -0.01 | 0.01 |
| 208 | rs4577304 | C | 0.18 | 0.03 | 0.02 | 0.01 | -0.01 | 0.01 |
| 209 | rs4651224 | T | 0.20 | 0.03 | 0.00 | 0.01 | -0.01 | 0.01 |
| 210 | rs4734868 | G | 0.18 | 0.03 | -0.02 | 0.01 | 0.00 | 0.01 |
| 211 | rs4775769 | G | 0.42 | 0.05 | 0.02 | 0.02 | 0.00 | 0.01 |
| 212 | rs483071 | T | 0.27 | 0.03 | 0.00 | 0.01 | 0.01 | 0.01 |
| 213 | rs4834792 | A | 0.20 | 0.03 | 0.03 | 0.01 | 0.00 | 0.01 |
| 214 | rs4838021 | T | -0.30 | 0.05 | 0.00 | 0.01 | 0.05 | 0.01 |
| 215 | rs4873492 | T | 0.34 | 0.04 | 0.01 | 0.01 | -0.02 | 0.01 |
| 216 | rs4876133 | C | 0.22 | 0.03 | 0.01 | 0.01 | 0.01 | 0.01 |
| 217 | rs4888408 | A | 0.37 | 0.03 | 0.04 | 0.01 | 0.00 | 0.01 |
| 218 | rs4925159 | A | 0.22 | 0.03 | 0.00 | 0.01 | 0.01 | 0.01 |
| 219 | rs4952609 | G | -0.21 | 0.03 | -0.01 | 0.01 | 0.00 | 0.01 |
| 220 | rs4955575 | C | -0.22 | 0.03 | 0.00 | 0.01 | 0.00 | 0.01 |
| 221 | rs4961293 | T | 0.23 | 0.03 | -0.01 | 0.01 | 0.00 | 0.01 |
| 222 | rs509564 | T | 0.26 | 0.04 | 0.02 | 0.01 | 0.01 | 0.01 |
| 223 | rs509833 | G | -0.33 | 0.04 | -0.04 | 0.01 | -0.01 | 0.01 |
| 224 | rs55732192 | T | -0.34 | 0.05 | 0.00 | 0.02 | 0.00 | 0.01 |
| 225 | rs55944332 | G | 0.26 | 0.04 | -0.03 | 0.01 | 0.00 | 0.01 |
| 226 | rs56407827 | T | 0.36 | 0.03 | 0.00 | 0.01 | 0.01 | 0.01 |
| 227 | rs571689 | T | 0.23 | 0.03 | 0.01 | 0.01 | 0.01 | 0.01 |
| 228 | rs573455 | G | -0.20 | 0.03 | -0.03 | 0.01 | -0.01 | 0.01 |
| 229 | rs5742643 | C | 0.22 | 0.03 | 0.00 | 0.01 | -0.01 | 0.01 |
| 230 | rs57786342 | A | 0.23 | 0.04 | 0.02 | 0.01 | -0.02 | 0.01 |
| 231 | rs57866767 | C | -0.45 | 0.03 | 0.03 | 0.01 | 0.01 | 0.01 |
| 232 | rs57946343 | C | -0.72 | 0.04 | -0.01 | 0.01 | 0.00 | 0.01 |
| 233 | rs60138042 | G | -0.34 | 0.06 | 0.01 | 0.02 | 0.03 | 0.02 |
| 234 | rs60191654 | G | 0.24 | 0.04 | 0.00 | 0.01 | 0.01 | 0.01 |
| 235 | rs6026578 | G | 0.19 | 0.03 | -0.01 | 0.01 | 0.02 | 0.01 |
| 236 | rs6026744 | T | 0.71 | 0.05 | 0.05 | 0.02 | -0.01 | 0.01 |
| 237 | rs6029756 | A | -0.27 | 0.03 | -0.01 | 0.01 | 0.00 | 0.01 |
| 238 | rs6031431 | G | 0.26 | 0.03 | 0.00 | 0.01 | 0.00 | 0.01 |
| 239 | rs60444686 | A | 0.59 | 0.08 | 0.05 | 0.02 | 0.01 | 0.02 |
| 240 | rs604723 | C | 0.66 | 0.03 | 0.03 | 0.01 | -0.01 | 0.01 |
| 241 | rs6054139 | A | 0.21 | 0.03 | 0.01 | 0.01 | 0.01 | 0.01 |
| 242 | rs6058088 | G | -0.28 | 0.04 | -0.01 | 0.01 | 0.03 | 0.01 |
| 243 | rs6078093 | A | -0.18 | 0.03 | 0.00 | 0.01 | 0.01 | 0.01 |
| 244 | rs6090907 | A | -0.39 | 0.04 | -0.05 | 0.01 | 0.01 | 0.01 |
| 245 | rs60909079 | C | -0.21 | 0.04 | 0.00 | 0.01 | -0.01 | 0.01 |
| 246 | rs60991988 | G | -0.38 | 0.05 | -0.04 | 0.01 | -0.01 | 0.01 |
| 247 | rs6108787 | G | 0.43 | 0.03 | 0.02 | 0.01 | 0.01 | 0.01 |
| 248 | rs61772592 | G | 0.32 | 0.05 | 0.06 | 0.01 | -0.02 | 0.01 |
| 249 | rs62076622 | G | -0.24 | 0.04 | 0.00 | 0.01 | 0.00 | 0.01 |
| 250 | rs62082230 | A | -0.19 | 0.03 | 0.02 | 0.01 | -0.01 | 0.01 |
| 251 | rs62309747 | A | -0.22 | 0.03 | 0.00 | 0.01 | 0.02 | 0.01 |
| 252 | rs62512914 | G | -0.21 | 0.03 | -0.02 | 0.01 | 0.01 | 0.01 |
| 253 | rs641620 | C | 0.32 | 0.04 | 0.01 | 0.01 | -0.02 | 0.01 |
| 254 | rs6438857 | C | -0.27 | 0.03 | -0.01 | 0.01 | 0.00 | 0.01 |
| 255 | rs6445583 | A | 0.28 | 0.03 | 0.00 | 0.01 | -0.01 | 0.01 |
| 256 | rs6452769 | A | -0.31 | 0.04 | -0.01 | 0.01 | 0.00 | 0.01 |
| 257 | rs6490019 | G | 0.29 | 0.03 | 0.01 | 0.01 | 0.02 | 0.01 |
| 258 | rs6504213 | C | 0.30 | 0.03 | 0.04 | 0.01 | -0.02 | 0.01 |
| 259 | rs6540119 | T | -0.20 | 0.03 | -0.01 | 0.01 | -0.02 | 0.01 |
| 260 | rs6562778 | G | -0.18 | 0.03 | -0.01 | 0.01 | 0.01 | 0.01 |
| 261 | rs658780 | G | 0.20 | 0.03 | -0.01 | 0.01 | 0.00 | 0.01 |
| 262 | rs665445 | A | -0.19 | 0.03 | 0.01 | 0.01 | 0.00 | 0.01 |
| 263 | rs66864335 | A | -0.40 | 0.04 | -0.05 | 0.01 | 0.03 | 0.01 |
| 264 | rs6699618 | G | -0.91 | 0.04 | -0.02 | 0.01 | 0.02 | 0.01 |
| 265 | rs6731373 | A | 0.19 | 0.03 | -0.01 | 0.01 | 0.02 | 0.01 |
| 266 | rs6732123 | C | -0.17 | 0.03 | -0.01 | 0.01 | 0.00 | 0.01 |
| 267 | rs6737318 | G | -0.23 | 0.04 | -0.01 | 0.01 | 0.00 | 0.01 |
| 268 | rs67617547 | G | -0.18 | 0.03 | -0.01 | 0.01 | 0.02 | 0.01 |
| 269 | rs6788907 | A | 0.22 | 0.03 | 0.00 | 0.01 | -0.01 | 0.01 |
| 270 | rs6788984 | G | -0.30 | 0.04 | -0.01 | 0.01 | 0.00 | 0.01 |
| 271 | rs68085857 | T | 0.27 | 0.04 | 0.02 | 0.01 | 0.00 | 0.01 |
| 272 | rs6870654 | C | -0.21 | 0.03 | -0.01 | 0.01 | 0.00 | 0.01 |
| 273 | rs6892983 | A | 0.34 | 0.03 | 0.03 | 0.01 | 0.00 | 0.01 |
| 274 | rs6921291 | T | 0.36 | 0.04 | -0.05 | 0.01 | -0.01 | 0.01 |
| 275 | rs6957161 | G | -0.21 | 0.03 | -0.03 | 0.01 | -0.01 | 0.01 |
| 276 | rs6961048 | G | 0.53 | 0.05 | 0.01 | 0.02 | -0.02 | 0.01 |
| 277 | rs6986368 | T | 0.21 | 0.03 | 0.02 | 0.01 | -0.02 | 0.01 |
| 278 | rs7012866 | G | 0.23 | 0.03 | 0.01 | 0.01 | -0.01 | 0.01 |
| 279 | rs702395 | T | 0.23 | 0.03 | 0.00 | 0.01 | 0.00 | 0.01 |
| 280 | rs7026176 | T | -0.19 | 0.03 | 0.00 | 0.01 | 0.02 | 0.01 |
| 281 | rs7045409 | A | -0.19 | 0.03 | 0.00 | 0.01 | 0.02 | 0.01 |
| 282 | rs708117 | A | 0.29 | 0.03 | -0.01 | 0.01 | 0.01 | 0.01 |
| 283 | rs7093894 | A | 0.24 | 0.04 | 0.01 | 0.01 | -0.03 | 0.01 |
| 284 | rs7107356 | G | 0.46 | 0.03 | 0.01 | 0.01 | 0.02 | 0.01 |
| 285 | rs7134677 | T | -0.39 | 0.03 | -0.02 | 0.01 | 0.02 | 0.01 |
| 286 | rs7154723 | A | 0.25 | 0.03 | 0.02 | 0.01 | -0.02 | 0.01 |
| 287 | rs7186298 | T | -0.23 | 0.03 | -0.03 | 0.01 | 0.00 | 0.01 |
| 288 | rs7198817 | A | -0.18 | 0.03 | -0.01 | 0.01 | 0.00 | 0.01 |
| 289 | rs7211535 | G | 0.18 | 0.03 | 0.00 | 0.01 | 0.01 | 0.01 |
| 290 | rs7213273 | A | -0.40 | 0.03 | -0.01 | 0.01 | 0.01 | 0.01 |
| 291 | rs7236548 | A | 0.34 | 0.04 | 0.00 | 0.01 | -0.01 | 0.01 |
| 292 | rs7255933 | A | 0.23 | 0.03 | 0.04 | 0.01 | -0.03 | 0.01 |
| 293 | rs72719160 | T | 0.22 | 0.03 | 0.01 | 0.01 | -0.03 | 0.01 |
| 294 | rs72742507 | T | -0.21 | 0.03 | -0.01 | 0.01 | -0.02 | 0.01 |
| 295 | rs7278003 | C | 0.19 | 0.03 | 0.03 | 0.01 | -0.02 | 0.01 |
| 296 | rs72847885 | G | -0.24 | 0.03 | -0.02 | 0.01 | 0.02 | 0.01 |
| 297 | rs72931748 | G | -0.40 | 0.05 | -0.02 | 0.02 | 0.00 | 0.01 |
| 298 | rs7306710 | C | 0.24 | 0.03 | 0.02 | 0.01 | -0.05 | 0.01 |
| 299 | rs73075659 | G | -0.40 | 0.03 | -0.01 | 0.01 | -0.01 | 0.01 |
| 300 | rs73103937 | C | -0.21 | 0.03 | -0.02 | 0.01 | -0.02 | 0.01 |
| 301 | rs7310615 | G | -0.59 | 0.03 | -0.06 | 0.01 | 0.03 | 0.01 |
| 302 | rs7331680 | T | 0.41 | 0.04 | 0.01 | 0.01 | 0.01 | 0.01 |
| 303 | rs73855810 | A | 0.27 | 0.04 | 0.06 | 0.01 | -0.02 | 0.01 |
| 304 | rs7395791 | A | -0.22 | 0.03 | -0.01 | 0.01 | 0.02 | 0.01 |
| 305 | rs740746 | A | 0.46 | 0.03 | 0.01 | 0.01 | 0.02 | 0.01 |
| 306 | rs7439567 | T | 0.25 | 0.03 | 0.02 | 0.01 | 0.00 | 0.01 |
| 307 | rs7463212 | A | -0.28 | 0.03 | 0.00 | 0.01 | 0.00 | 0.01 |
| 308 | rs7491248 | A | 0.22 | 0.04 | 0.02 | 0.01 | 0.00 | 0.01 |
| 309 | rs7493678 | T | 0.19 | 0.03 | 0.01 | 0.01 | -0.01 | 0.01 |
| 310 | rs75016974 | T | -0.25 | 0.04 | 0.00 | 0.01 | -0.01 | 0.01 |
| 311 | rs7514579 | C | -0.22 | 0.04 | 0.01 | 0.01 | -0.01 | 0.01 |
| 312 | rs75461554 | T | -0.30 | 0.04 | -0.02 | 0.01 | -0.01 | 0.01 |
| 313 | rs7555285 | C | 0.23 | 0.04 | 0.01 | 0.01 | -0.02 | 0.01 |
| 314 | rs75961402 | A | 0.27 | 0.04 | -0.01 | 0.01 | 0.01 | 0.01 |
| 315 | rs7615099 | G | -0.19 | 0.03 | -0.04 | 0.01 | 0.03 | 0.01 |
| 316 | rs76443575 | C | -0.52 | 0.08 | 0.02 | 0.02 | -0.05 | 0.02 |
| 317 | rs7683728 | T | -0.37 | 0.03 | -0.03 | 0.01 | 0.00 | 0.01 |
| 318 | rs7703560 | G | 0.22 | 0.03 | 0.01 | 0.01 | 0.00 | 0.01 |
| 319 | rs7722243 | A | -0.20 | 0.03 | -0.01 | 0.01 | -0.01 | 0.01 |
| 320 | rs7725413 | T | -0.20 | 0.04 | -0.01 | 0.01 | 0.00 | 0.01 |
| 321 | rs77375686 | G | 0.35 | 0.05 | 0.03 | 0.02 | 0.00 | 0.01 |
| 322 | rs7744902 | A | -0.41 | 0.06 | -0.02 | 0.02 | 0.00 | 0.02 |
| 323 | rs7763558 | A | 0.34 | 0.03 | 0.02 | 0.01 | -0.03 | 0.01 |
| 324 | rs7765526 | G | -0.20 | 0.03 | 0.00 | 0.01 | 0.01 | 0.01 |
| 325 | rs778124 | A | 0.30 | 0.03 | 0.02 | 0.01 | 0.00 | 0.01 |
| 326 | rs7796 | G | -0.34 | 0.03 | -0.01 | 0.01 | 0.00 | 0.01 |
| 327 | rs7821832 | G | -0.42 | 0.03 | -0.02 | 0.01 | 0.00 | 0.01 |
| 328 | rs7844887 | A | 0.27 | 0.04 | 0.00 | 0.01 | 0.01 | 0.01 |
| 329 | rs78474310 | G | 0.47 | 0.07 | 0.03 | 0.03 | 0.01 | 0.02 |
| 330 | rs786923 | T | -0.31 | 0.03 | -0.01 | 0.01 | 0.01 | 0.01 |
| 331 | rs78998485 | G | 0.24 | 0.03 | -0.03 | 0.01 | -0.03 | 0.01 |
| 332 | rs7912283 | A | -0.21 | 0.03 | -0.01 | 0.01 | 0.00 | 0.01 |
| 333 | rs7926110 | G | -0.26 | 0.03 | -0.02 | 0.01 | 0.00 | 0.01 |
| 334 | rs79384779 | T | 0.32 | 0.04 | 0.02 | 0.01 | -0.02 | 0.01 |
| 335 | rs79539362 | C | -0.40 | 0.05 | 0.03 | 0.02 | 0.00 | 0.01 |
| 336 | rs7963801 | C | 0.24 | 0.03 | 0.02 | 0.01 | 0.00 | 0.01 |
| 337 | rs79782817 | T | 0.53 | 0.05 | 0.01 | 0.02 | -0.01 | 0.01 |
| 338 | rs7980644 | G | -0.26 | 0.04 | 0.02 | 0.01 | 0.02 | 0.01 |
| 339 | rs8003103 | A | -0.18 | 0.03 | 0.00 | 0.01 | 0.01 | 0.01 |
| 340 | rs8030856 | G | 0.18 | 0.03 | 0.01 | 0.01 | -0.01 | 0.01 |
| 341 | rs8044992 | C | -0.21 | 0.03 | -0.01 | 0.01 | 0.01 | 0.01 |
| 342 | rs8054587 | C | -0.17 | 0.03 | -0.01 | 0.01 | 0.00 | 0.01 |
| 343 | rs8125763 | A | 0.18 | 0.03 | -0.01 | 0.01 | -0.01 | 0.01 |
| 344 | rs8142376 | T | 0.17 | 0.03 | 0.02 | 0.01 | 0.01 | 0.01 |
| 345 | rs8180684 | T | 0.21 | 0.03 | 0.02 | 0.01 | -0.01 | 0.01 |
| 346 | rs848445 | C | 0.20 | 0.03 | 0.00 | 0.01 | -0.03 | 0.01 |
| 347 | rs869396 | A | -0.21 | 0.03 | -0.04 | 0.01 | 0.00 | 0.01 |
| 348 | rs871004 | A | 0.23 | 0.03 | 0.01 | 0.01 | -0.03 | 0.01 |
| 349 | rs8904 | A | 0.31 | 0.03 | 0.00 | 0.01 | 0.00 | 0.01 |
| 350 | rs927315 | T | 0.17 | 0.03 | 0.00 | 0.01 | 0.00 | 0.01 |
| 351 | rs9285476 | G | -0.18 | 0.03 | -0.07 | 0.01 | 0.01 | 0.01 |
| 352 | rs9302885 | G | -0.22 | 0.03 | -0.01 | 0.01 | 0.02 | 0.01 |
| 353 | rs9327297 | G | -0.27 | 0.03 | -0.03 | 0.01 | 0.02 | 0.01 |
| 354 | rs9349379 | G | -0.27 | 0.03 | 0.13 | 0.01 | 0.01 | 0.01 |
| 355 | rs9361836 | T | 0.22 | 0.03 | 0.00 | 0.01 | 0.01 | 0.01 |
| 356 | rs9368222 | A | 0.23 | 0.03 | 0.02 | 0.01 | -0.13 | 0.01 |
| 357 | rs9401913 | A | 0.52 | 0.03 | 0.01 | 0.01 | 0.02 | 0.01 |
| 358 | rs9486916 | T | 0.27 | 0.04 | 0.01 | 0.01 | 0.02 | 0.01 |
| 359 | rs9526707 | A | -0.20 | 0.03 | -0.01 | 0.01 | -0.01 | 0.01 |
| 360 | rs961764 | G | 0.19 | 0.03 | -0.01 | 0.01 | -0.02 | 0.01 |
| 361 | rs9848170 | C | 0.32 | 0.03 | 0.01 | 0.01 | -0.01 | 0.01 |
| 362 | rs9857362 | C | -0.17 | 0.03 | 0.00 | 0.01 | -0.01 | 0.01 |
| 363 | rs9869437 | A | -0.20 | 0.03 | 0.00 | 0.01 | -0.02 | 0.01 |
| 364 | rs9876694 | T | 0.47 | 0.07 | -0.01 | 0.02 | 0.05 | 0.02 |
| 365 | rs9880098 | A | 0.31 | 0.03 | 0.02 | 0.01 | 0.00 | 0.01 |
| 366 | rs9886665 | C | -0.20 | 0.03 | -0.01 | 0.01 | 0.01 | 0.01 |
| 367 | rs9899540 | T | -0.20 | 0.03 | 0.00 | 0.01 | -0.01 | 0.01 |
| 368 | rs9918876 | A | -0.30 | 0.05 | -0.01 | 0.01 | 0.00 | 0.01 |

SBP: Systolic blood pressure; CHD: Coronary heart disease; T2DM: Type-2 diabetes

Table 32: Genetic association estimates for the effect of DBP on CHD, adjusted for T2DM. ea=effect allele, gx=DBP, gy=CHD, gz=T2DM, se=standard error

|  | SNP | ea | gx | gx_se | gy | gy_se | gz | gz_se |
| --- | --- | --- | --- | --- | --- | --- | --- | --- |
| 1 | rs10048404 | T | -0.11 | 0.02 | -0.02 | 0.01 | 0.02 | 0.01 |
| 2 | rs10062049 | T | 0.22 | 0.03 | 0.01 | 0.01 | 0.02 | 0.01 |
| 3 | rs1006545 | T | 0.36 | 0.03 | -0.02 | 0.02 | 0.03 | 0.01 |
| 4 | rs10087280 | G | -0.14 | 0.02 | 0.00 | 0.01 | 0.02 | 0.01 |
| 5 | rs10164193 | G | 0.22 | 0.03 | 0.01 | 0.02 | 0.01 | 0.01 |
| 6 | rs10279432 | A | 0.12 | 0.02 | 0.02 | 0.01 | -0.02 | 0.01 |
| 7 | rs1035673 | C | -0.16 | 0.02 | -0.04 | 0.01 | 0.01 | 0.01 |
| 8 | rs1044822 | T | -0.13 | 0.02 | -0.02 | 0.01 | 0.02 | 0.01 |
| 9 | rs10490923 | A | 0.15 | 0.03 | 0.01 | 0.01 | -0.02 | 0.01 |
| 10 | rs10491713 | T | -0.12 | 0.02 | 0.00 | 0.01 | 0.00 | 0.01 |
| 11 | rs1049212 | G | 0.18 | 0.02 | 0.01 | 0.01 | 0.01 | 0.01 |
| 12 | rs10493408 | A | 0.16 | 0.03 | 0.00 | 0.01 | -0.03 | 0.01 |
| 13 | rs10500932 | A | 0.28 | 0.03 | 0.02 | 0.01 | -0.02 | 0.01 |
| 14 | rs10759697 | A | 0.13 | 0.02 | 0.01 | 0.01 | 0.02 | 0.01 |
| 15 | rs10804330 | C | -0.13 | 0.02 | -0.02 | 0.01 | 0.07 | 0.01 |
| 16 | rs10832586 | C | 0.31 | 0.02 | 0.01 | 0.01 | -0.02 | 0.01 |
| 17 | rs10838702 | T | 0.24 | 0.02 | 0.00 | 0.01 | 0.01 | 0.01 |
| 18 | rs10873612 | T | -0.11 | 0.02 | -0.02 | 0.01 | 0.00 | 0.01 |
| 19 | rs10941043 | G | 0.13 | 0.02 | 0.00 | 0.01 | -0.01 | 0.01 |
| 20 | rs10980408 | C | 0.37 | 0.05 | 0.04 | 0.03 | -0.03 | 0.02 |
| 21 | rs11021221 | A | -0.19 | 0.02 | -0.02 | 0.01 | 0.00 | 0.01 |
| 22 | rs11070245 | G | 0.13 | 0.02 | -0.01 | 0.01 | -0.01 | 0.01 |
| 23 | rs11108209 | C | 0.19 | 0.03 | -0.04 | 0.02 | -0.01 | 0.01 |
| 24 | rs11112548 | T | -0.27 | 0.04 | 0.04 | 0.03 | 0.02 | 0.02 |
| 25 | rs11130602 | A | 0.15 | 0.02 | 0.01 | 0.01 | 0.02 | 0.01 |
| 26 | rs11141731 | T | -0.13 | 0.02 | 0.00 | 0.01 | 0.00 | 0.01 |
| 27 | rs1114347 | G | 0.18 | 0.02 | 0.03 | 0.01 | 0.01 | 0.01 |
| 28 | rs11153590 | A | -0.11 | 0.02 | 0.02 | 0.01 | 0.00 | 0.01 |
| 29 | rs11153730 | C | -0.16 | 0.02 | 0.02 | 0.01 | -0.01 | 0.01 |
| 30 | rs11187838 | A | -0.28 | 0.02 | 0.02 | 0.01 | 0.01 | 0.01 |
| 31 | rs11191580 | C | -0.51 | 0.03 | -0.07 | 0.01 | 0.00 | 0.01 |
| 32 | rs11231693 | A | 0.22 | 0.04 | 0.02 | 0.02 | -0.06 | 0.02 |
| 33 | rs112393817 | G | -0.12 | 0.02 | 0.02 | 0.01 | -0.01 | 0.01 |
| 34 | rs11245631 | T | -0.15 | 0.02 | 0.00 | 0.01 | 0.02 | 0.01 |
| 35 | rs11252324 | T | -0.23 | 0.03 | -0.06 | 0.02 | -0.01 | 0.01 |
| 36 | rs114714860 | C | 0.33 | 0.02 | 0.00 | 0.01 | -0.01 | 0.01 |
| 37 | rs11556924 | T | -0.18 | 0.02 | -0.07 | 0.01 | 0.01 | 0.01 |
| 38 | rs11578696 | G | -0.15 | 0.03 | -0.02 | 0.02 | -0.01 | 0.01 |
| 39 | rs11592107 | A | 0.12 | 0.02 | 0.02 | 0.01 | -0.03 | 0.01 |
| 40 | rs11636952 | C | -0.40 | 0.02 | -0.02 | 0.01 | 0.01 | 0.01 |
| 41 | rs11661473 | A | 0.20 | 0.02 | 0.00 | 0.01 | 0.01 | 0.01 |
| 42 | rs11664194 | A | -0.11 | 0.02 | 0.03 | 0.01 | -0.01 | 0.01 |
| 43 | rs11665020 | C | -0.14 | 0.02 | -0.01 | 0.01 | 0.00 | 0.01 |
| 44 | rs11721984 | T | -0.14 | 0.02 | -0.03 | 0.01 | 0.01 | 0.01 |
| 45 | rs11745207 | G | -0.11 | 0.02 | -0.02 | 0.01 | 0.01 | 0.01 |
| 46 | rs11778153 | C | -0.12 | 0.02 | 0.01 | 0.01 | 0.00 | 0.01 |
| 47 | rs1178979 | C | -0.15 | 0.02 | -0.01 | 0.01 | -0.02 | 0.01 |
| 48 | rs11923343 | G | 0.11 | 0.02 | 0.00 | 0.01 | 0.01 | 0.01 |
| 49 | rs11923667 | A | 0.12 | 0.02 | 0.00 | 0.01 | 0.01 | 0.01 |
| 50 | rs11945489 | T | -0.14 | 0.02 | -0.02 | 0.01 | 0.02 | 0.01 |
| 51 | rs11960210 | C | -0.25 | 0.02 | -0.02 | 0.01 | 0.02 | 0.01 |
| 52 | rs11961593 | T | -0.32 | 0.03 | -0.02 | 0.02 | 0.00 | 0.01 |
| 53 | rs12088448 | C | 0.15 | 0.02 | -0.01 | 0.01 | 0.00 | 0.01 |
| 54 | rs1212061 | C | 0.13 | 0.02 | -0.02 | 0.01 | 0.00 | 0.01 |
| 55 | rs12149254 | A | -0.13 | 0.02 | 0.01 | 0.01 | -0.01 | 0.01 |
| 56 | rs12152463 | T | 0.10 | 0.02 | -0.01 | 0.01 | -0.01 | 0.01 |
| 57 | rs12258967 | G | -0.35 | 0.02 | -0.03 | 0.01 | -0.02 | 0.01 |
| 58 | rs12321 | C | -0.15 | 0.02 | -0.02 | 0.01 | -0.01 | 0.01 |
| 59 | rs12337056 | T | 0.14 | 0.02 | 0.01 | 0.01 | 0.02 | 0.01 |
| 60 | rs12363520 | A | 0.17 | 0.02 | -0.02 | 0.01 | 0.01 | 0.01 |
| 61 | rs12405515 | T | -0.17 | 0.02 | -0.02 | 0.01 | 0.04 | 0.01 |
| 62 | rs1243876 | T | -0.11 | 0.02 | -0.03 | 0.01 | -0.03 | 0.01 |
| 63 | rs12503341 | A | -0.30 | 0.05 | 0.02 | 0.02 | 0.02 | 0.02 |
| 64 | rs12509595 | C | 0.50 | 0.02 | 0.05 | 0.01 | -0.02 | 0.01 |
| 65 | rs12515541 | T | 0.12 | 0.02 | 0.02 | 0.01 | -0.02 | 0.01 |
| 66 | rs12574332 | T | 0.21 | 0.03 | 0.03 | 0.01 | -0.01 | 0.01 |
| 67 | rs12609484 | T | -0.14 | 0.02 | -0.02 | 0.01 | 0.01 | 0.01 |
| 68 | rs1263671 | C | 0.14 | 0.02 | 0.03 | 0.01 | -0.01 | 0.01 |
| 69 | rs12656497 | C | 0.31 | 0.02 | 0.01 | 0.01 | 0.00 | 0.01 |
| 70 | rs1265842 | C | -0.11 | 0.02 | 0.00 | 0.01 | -0.01 | 0.01 |
| 71 | rs12693302 | A | -0.24 | 0.02 | -0.03 | 0.01 | -0.02 | 0.01 |
| 72 | rs12728150 | G | 0.20 | 0.03 | 0.02 | 0.02 | -0.01 | 0.01 |
| 73 | rs1275985 | T | -0.29 | 0.02 | 0.00 | 0.01 | -0.01 | 0.01 |
| 74 | rs12790943 | T | -0.10 | 0.02 | -0.03 | 0.01 | 0.00 | 0.01 |
| 75 | rs12906962 | C | 0.24 | 0.02 | 0.02 | 0.01 | -0.01 | 0.01 |
| 76 | rs12919839 | T | -0.11 | 0.02 | -0.01 | 0.01 | 0.00 | 0.01 |
| 77 | rs12929303 | A | 0.16 | 0.02 | 0.02 | 0.01 | -0.02 | 0.01 |
| 78 | rs12938803 | C | 0.16 | 0.02 | -0.03 | 0.01 | -0.01 | 0.01 |
| 79 | rs12990959 | C | 0.13 | 0.02 | 0.04 | 0.01 | -0.01 | 0.01 |
| 80 | rs13004222 | G | -0.29 | 0.04 | 0.02 | 0.02 | -0.02 | 0.02 |
| 81 | rs13118687 | A | -0.15 | 0.02 | -0.01 | 0.01 | 0.00 | 0.01 |
| 82 | rs13124515 | C | 0.11 | 0.02 | 0.00 | 0.01 | -0.01 | 0.01 |
| 83 | rs13139571 | A | -0.24 | 0.02 | -0.05 | 0.01 | 0.02 | 0.01 |
| 84 | rs13152154 | T | -0.12 | 0.02 | -0.02 | 0.01 | -0.01 | 0.01 |
| 85 | rs13215166 | G | 0.31 | 0.02 | 0.01 | 0.01 | 0.02 | 0.01 |
| 86 | rs1322639 | A | -0.16 | 0.02 | -0.01 | 0.01 | -0.01 | 0.01 |
| 87 | rs13237249 | T | 0.14 | 0.02 | 0.01 | 0.01 | -0.01 | 0.01 |
| 88 | rs1327235 | G | 0.30 | 0.02 | 0.02 | 0.01 | 0.00 | 0.01 |
| 89 | rs1332812 | A | -0.11 | 0.02 | 0.01 | 0.01 | 0.00 | 0.01 |
| 90 | rs13355146 | T | 0.12 | 0.02 | 0.01 | 0.01 | 0.00 | 0.01 |
| 91 | rs13358657 | G | 0.22 | 0.03 | 0.01 | 0.01 | -0.01 | 0.01 |
| 92 | rs135023 | G | 0.10 | 0.02 | 0.01 | 0.01 | -0.01 | 0.01 |
| 93 | rs1373780 | C | 0.12 | 0.02 | 0.01 | 0.01 | 0.01 | 0.01 |
| 94 | rs1390754 | T | 0.13 | 0.02 | 0.01 | 0.01 | 0.03 | 0.01 |
| 95 | rs142449193 | T | -0.26 | 0.04 | -0.02 | 0.03 | -0.03 | 0.02 |
| 96 | rs1425486 | T | -0.13 | 0.02 | -0.01 | 0.01 | 0.04 | 0.01 |
| 97 | rs1433121 | T | -0.14 | 0.02 | -0.01 | 0.01 | -0.02 | 0.01 |
| 98 | rs1446468 | C | 0.25 | 0.02 | 0.02 | 0.01 | 0.01 | 0.01 |
| 99 | rs1449596 | G | 0.11 | 0.02 | 0.00 | 0.01 | 0.00 | 0.01 |
| 100 | rs1467049 | G | -0.12 | 0.02 | -0.01 | 0.01 | -0.01 | 0.01 |
| 101 | rs15009 | G | 0.12 | 0.02 | 0.00 | 0.01 | 0.02 | 0.01 |
| 102 | rs1502358 | A | -0.11 | 0.02 | -0.01 | 0.01 | 0.00 | 0.01 |
| 103 | rs1523871 | G | 0.12 | 0.02 | 0.00 | 0.01 | 0.01 | 0.01 |
| 104 | rs1527797 | T | -0.14 | 0.02 | 0.03 | 0.01 | -0.02 | 0.01 |
| 105 | rs1528293 | T | -0.28 | 0.02 | -0.01 | 0.01 | 0.01 | 0.01 |
| 106 | rs1534338 | A | -0.11 | 0.02 | 0.00 | 0.01 | 0.00 | 0.01 |
| 107 | rs1582931 | A | 0.22 | 0.02 | -0.02 | 0.01 | 0.01 | 0.01 |
| 108 | rs1623474 | T | 0.22 | 0.02 | 0.01 | 0.01 | 0.00 | 0.01 |
| 109 | rs1669907 | G | -0.12 | 0.02 | -0.01 | 0.01 | 0.00 | 0.01 |
| 110 | rs16853198 | G | -0.34 | 0.03 | -0.04 | 0.02 | 0.01 | 0.01 |
| 111 | rs1687295 | C | -0.21 | 0.02 | -0.01 | 0.01 | 0.03 | 0.01 |
| 112 | rs16875357 | G | 0.12 | 0.02 | 0.00 | 0.01 | 0.00 | 0.01 |
| 113 | rs16896276 | A | -0.13 | 0.02 | -0.03 | 0.01 | 0.03 | 0.01 |
| 114 | rs1693560 | G | -0.15 | 0.02 | 0.00 | 0.01 | 0.00 | 0.01 |
| 115 | rs17321041 | T | 0.23 | 0.04 | -0.01 | 0.02 | 0.01 | 0.02 |
| 116 | rs173396 | A | 0.21 | 0.02 | 0.05 | 0.01 | 0.00 | 0.01 |
| 117 | rs17396055 | A | -0.12 | 0.02 | 0.01 | 0.01 | 0.00 | 0.01 |
| 118 | rs17432462 | C | 0.10 | 0.02 | 0.01 | 0.01 | 0.00 | 0.01 |
| 119 | rs17454517 | G | -0.12 | 0.02 | 0.00 | 0.01 | 0.00 | 0.01 |
| 120 | rs17677603 | G | 0.20 | 0.02 | 0.03 | 0.01 | 0.00 | 0.01 |
| 121 | rs17696749 | G | 0.13 | 0.02 | 0.04 | 0.01 | 0.00 | 0.01 |
| 122 | rs17807723 | A | -0.18 | 0.03 | -0.02 | 0.02 | -0.01 | 0.01 |
| 123 | rs1790123 | T | 0.20 | 0.02 | 0.02 | 0.01 | -0.04 | 0.01 |
| 124 | rs1799945 | G | 0.39 | 0.02 | 0.01 | 0.01 | -0.02 | 0.01 |
| 125 | rs1819663 | G | -0.11 | 0.02 | 0.01 | 0.01 | 0.00 | 0.01 |
| 126 | rs1867624 | T | 0.14 | 0.02 | 0.04 | 0.01 | -0.02 | 0.01 |
| 127 | rs1871190 | T | 0.11 | 0.02 | 0.03 | 0.01 | -0.01 | 0.01 |
| 128 | rs1876490 | A | 0.14 | 0.02 | 0.02 | 0.01 | 0.00 | 0.01 |
| 129 | rs1882961 | T | 0.13 | 0.02 | 0.04 | 0.01 | 0.01 | 0.01 |
| 130 | rs1889785 | A | 0.13 | 0.02 | 0.00 | 0.01 | -0.01 | 0.01 |
| 131 | rs1903752 | T | -0.10 | 0.02 | -0.02 | 0.01 | 0.01 | 0.01 |
| 132 | rs1906672 | A | 0.14 | 0.02 | 0.00 | 0.01 | -0.01 | 0.01 |
| 133 | rs194742 | C | -0.13 | 0.02 | -0.03 | 0.01 | 0.02 | 0.01 |
| 134 | rs1948151 | A | -0.14 | 0.02 | -0.02 | 0.01 | 0.02 | 0.01 |
| 135 | rs1950500 | C | -0.14 | 0.02 | 0.00 | 0.01 | 0.00 | 0.01 |
| 136 | rs1984195 | A | 0.17 | 0.02 | 0.03 | 0.01 | -0.01 | 0.01 |
| 137 | rs1999996 | G | 0.11 | 0.02 | 0.00 | 0.01 | -0.01 | 0.01 |
| 138 | rs2067831 | C | -0.13 | 0.02 | 0.04 | 0.01 | 0.01 | 0.01 |
| 139 | rs2070527 | C | 0.15 | 0.02 | 0.03 | 0.01 | -0.02 | 0.01 |
| 140 | rs2133386 | A | -0.13 | 0.02 | 0.03 | 0.01 | 0.00 | 0.01 |
| 141 | rs2146315 | T | -0.12 | 0.02 | -0.01 | 0.01 | 0.01 | 0.01 |
| 142 | rs2160236 | C | -0.14 | 0.02 | 0.00 | 0.01 | -0.01 | 0.01 |
| 143 | rs2169137 | C | 0.16 | 0.02 | 0.01 | 0.01 | 0.02 | 0.01 |
| 144 | rs2191046 | G | -0.12 | 0.02 | -0.02 | 0.01 | 0.01 | 0.01 |
| 145 | rs2236295 | T | -0.21 | 0.02 | 0.01 | 0.01 | 0.03 | 0.01 |
| 146 | rs2239268 | A | 0.11 | 0.02 | 0.00 | 0.01 | 0.00 | 0.01 |
| 147 | rs2239917 | C | -0.17 | 0.02 | 0.00 | 0.01 | 0.01 | 0.01 |
| 148 | rs2256187 | A | -0.14 | 0.02 | -0.03 | 0.01 | 0.02 | 0.01 |
| 149 | rs2273654 | C | -0.12 | 0.02 | 0.00 | 0.01 | -0.01 | 0.01 |
| 150 | rs227426 | T | 0.11 | 0.02 | 0.01 | 0.01 | 0.00 | 0.01 |
| 151 | rs2305654 | A | 0.17 | 0.02 | -0.01 | 0.01 | -0.03 | 0.01 |
| 152 | rs2307111 | C | 0.17 | 0.02 | -0.01 | 0.01 | 0.04 | 0.01 |
| 153 | rs234616 | A | -0.12 | 0.02 | 0.00 | 0.01 | 0.00 | 0.01 |
| 154 | rs2384061 | A | -0.17 | 0.02 | 0.01 | 0.01 | -0.01 | 0.01 |
| 155 | rs2397060 | C | 0.16 | 0.03 | 0.00 | 0.01 | -0.04 | 0.01 |
| 156 | rs2421200 | T | -0.11 | 0.02 | -0.01 | 0.01 | 0.00 | 0.01 |
| 157 | rs2444769 | A | 0.16 | 0.02 | -0.01 | 0.01 | 0.00 | 0.01 |
| 158 | rs2484294 | A | 0.32 | 0.02 | 0.01 | 0.01 | 0.02 | 0.01 |
| 159 | rs2487926 | G | -0.10 | 0.02 | 0.06 | 0.01 | 0.00 | 0.01 |
| 160 | rs2493136 | T | 0.23 | 0.02 | 0.02 | 0.01 | -0.01 | 0.01 |
| 161 | rs2515424 | T | 0.13 | 0.02 | 0.02 | 0.01 | -0.01 | 0.01 |
| 162 | rs2548459 | C | 0.13 | 0.02 | 0.01 | 0.01 | 0.01 | 0.01 |
| 163 | rs2586970 | G | 0.15 | 0.02 | 0.00 | 0.01 | -0.02 | 0.01 |
| 164 | rs2589218 | C | 0.12 | 0.02 | 0.02 | 0.01 | -0.01 | 0.01 |
| 165 | rs2598 | G | -0.14 | 0.02 | 0.02 | 0.01 | -0.01 | 0.01 |
| 166 | rs2627313 | T | 0.15 | 0.02 | 0.01 | 0.01 | -0.02 | 0.01 |
| 167 | rs2643826 | T | 0.19 | 0.02 | 0.00 | 0.01 | -0.02 | 0.01 |
| 168 | rs2681485 | A | 0.29 | 0.02 | -0.04 | 0.01 | 0.00 | 0.01 |
| 169 | rs2744133 | G | -0.14 | 0.02 | 0.00 | 0.01 | 0.01 | 0.01 |
| 170 | rs28377357 | A | -0.12 | 0.02 | -0.02 | 0.01 | 0.02 | 0.01 |
| 171 | rs28544928 | G | -0.15 | 0.02 | 0.00 | 0.01 | 0.02 | 0.01 |
| 172 | rs2854746 | C | 0.11 | 0.02 | 0.02 | 0.01 | 0.02 | 0.01 |
| 173 | rs28675079 | A | -0.14 | 0.02 | -0.01 | 0.01 | -0.01 | 0.01 |
| 174 | rs2921604 | C | 0.10 | 0.02 | 0.00 | 0.01 | 0.01 | 0.01 |
| 175 | rs2925345 | C | -0.19 | 0.02 | -0.01 | 0.01 | 0.00 | 0.01 |
| 176 | rs2957468 | G | -0.14 | 0.02 | 0.01 | 0.01 | -0.01 | 0.01 |
| 177 | rs3006583 | C | 0.13 | 0.02 | -0.02 | 0.01 | -0.02 | 0.01 |
| 178 | rs310597 | G | 0.12 | 0.02 | 0.00 | 0.01 | 0.02 | 0.01 |
| 179 | rs311564 | A | -0.13 | 0.02 | -0.02 | 0.01 | 0.00 | 0.01 |
| 180 | rs3117736 | T | 0.24 | 0.02 | 0.02 | 0.01 | -0.01 | 0.01 |
| 181 | rs318712 | C | 0.24 | 0.03 | 0.03 | 0.02 | 0.01 | 0.02 |
| 182 | rs335170 | C | -0.11 | 0.02 | -0.03 | 0.01 | 0.01 | 0.01 |
| 183 | rs342977 | A | -0.16 | 0.02 | -0.02 | 0.01 | 0.00 | 0.01 |
| 184 | rs34413141 | A | -0.18 | 0.02 | -0.03 | 0.01 | 0.01 | 0.01 |
| 185 | rs34587839 | A | -0.17 | 0.02 | 0.01 | 0.01 | -0.04 | 0.01 |
| 186 | rs34645159 | A | -0.13 | 0.02 | -0.01 | 0.01 | 0.00 | 0.01 |
| 187 | rs347585 | T | 0.15 | 0.02 | 0.01 | 0.01 | -0.01 | 0.01 |
| 188 | rs35091929 | C | -0.18 | 0.02 | -0.02 | 0.01 | 0.04 | 0.01 |
| 189 | rs35261542 | A | 0.12 | 0.02 | 0.02 | 0.01 | -0.12 | 0.01 |
| 190 | rs35413927 | G | 0.13 | 0.02 | 0.01 | 0.01 | 0.00 | 0.01 |
| 191 | rs35443 | C | -0.27 | 0.02 | 0.00 | 0.01 | 0.00 | 0.01 |
| 192 | rs35506078 | C | 0.13 | 0.02 | 0.00 | 0.01 | -0.02 | 0.01 |
| 193 | rs3735533 | C | 0.49 | 0.03 | 0.00 | 0.02 | 0.03 | 0.01 |
| 194 | rs3743111 | A | 0.15 | 0.02 | -0.02 | 0.01 | -0.01 | 0.01 |
| 195 | rs3743369 | A | 0.10 | 0.02 | 0.00 | 0.01 | -0.01 | 0.01 |
| 196 | rs3761077 | T | 0.17 | 0.03 | 0.00 | 0.02 | -0.06 | 0.01 |
| 197 | rs3772219 | C | -0.18 | 0.02 | -0.02 | 0.01 | 0.00 | 0.01 |
| 198 | rs3774702 | A | 0.15 | 0.02 | -0.02 | 0.01 | -0.01 | 0.01 |
| 199 | rs3776299 | A | 0.13 | 0.02 | 0.03 | 0.01 | -0.02 | 0.01 |
| 200 | rs3785837 | A | 0.15 | 0.02 | 0.03 | 0.01 | -0.01 | 0.01 |
| 201 | rs3798293 | G | 0.13 | 0.02 | -0.04 | 0.01 | -0.01 | 0.01 |
| 202 | rs3802230 | A | -0.16 | 0.02 | 0.01 | 0.01 | 0.00 | 0.01 |
| 203 | rs3802517 | A | 0.13 | 0.02 | 0.00 | 0.01 | -0.01 | 0.01 |
| 204 | rs3807101 | T | -0.17 | 0.03 | 0.01 | 0.01 | -0.01 | 0.01 |
| 205 | rs3861113 | A | 0.21 | 0.03 | 0.01 | 0.01 | 0.00 | 0.01 |
| 206 | rs3864004 | A | 0.10 | 0.02 | 0.01 | 0.01 | -0.01 | 0.01 |
| 207 | rs3943093 | T | 0.25 | 0.02 | 0.00 | 0.01 | 0.00 | 0.01 |
| 208 | rs4074812 | A | -0.13 | 0.02 | 0.00 | 0.01 | -0.01 | 0.01 |
| 209 | rs4077158 | C | 0.18 | 0.02 | 0.01 | 0.01 | 0.01 | 0.01 |
| 210 | rs4141663 | T | -0.15 | 0.02 | -0.01 | 0.01 | 0.00 | 0.01 |
| 211 | rs4244200 | C | -0.12 | 0.02 | 0.01 | 0.01 | -0.02 | 0.01 |
| 212 | rs4245930 | A | -0.12 | 0.02 | 0.00 | 0.01 | -0.01 | 0.01 |
| 213 | rs4306343 | T | 0.32 | 0.02 | 0.03 | 0.01 | 0.00 | 0.01 |
| 214 | rs4362428 | A | -0.11 | 0.02 | 0.00 | 0.01 | 0.00 | 0.01 |
| 215 | rs440454 | G | 0.26 | 0.02 | -0.03 | 0.01 | -0.03 | 0.01 |
| 216 | rs4424827 | T | -0.10 | 0.02 | 0.00 | 0.01 | -0.01 | 0.01 |
| 217 | rs4507125 | C | 0.12 | 0.02 | 0.00 | 0.01 | 0.02 | 0.01 |
| 218 | rs45474499 | T | 0.36 | 0.04 | 0.00 | 0.02 | 0.04 | 0.02 |
| 219 | rs4556017 | T | -0.16 | 0.02 | -0.01 | 0.01 | -0.02 | 0.01 |
| 220 | rs4615669 | G | 0.11 | 0.02 | -0.04 | 0.01 | 0.02 | 0.01 |
| 221 | rs4645335 | G | -0.11 | 0.02 | -0.01 | 0.01 | 0.00 | 0.01 |
| 222 | rs4675682 | C | 0.14 | 0.02 | 0.01 | 0.01 | 0.00 | 0.01 |
| 223 | rs4704514 | T | 0.11 | 0.02 | 0.02 | 0.01 | 0.00 | 0.01 |
| 224 | rs4739832 | C | -0.13 | 0.02 | -0.02 | 0.01 | 0.01 | 0.01 |
| 225 | rs4756779 | G | 0.15 | 0.02 | 0.00 | 0.01 | -0.02 | 0.01 |
| 226 | rs4814837 | T | -0.10 | 0.02 | -0.02 | 0.01 | 0.01 | 0.01 |
| 227 | rs4873492 | T | 0.14 | 0.02 | 0.01 | 0.01 | -0.02 | 0.01 |
| 228 | rs4890499 | A | 0.11 | 0.02 | 0.01 | 0.01 | 0.00 | 0.01 |
| 229 | rs4891258 | G | 0.12 | 0.02 | 0.00 | 0.01 | -0.01 | 0.01 |
| 230 | rs4903064 | C | -0.15 | 0.02 | 0.02 | 0.01 | -0.02 | 0.01 |
| 231 | rs4909314 | A | 0.13 | 0.02 | 0.01 | 0.01 | -0.01 | 0.01 |
| 232 | rs4926901 | A | 0.10 | 0.02 | 0.01 | 0.01 | 0.01 | 0.01 |
| 233 | rs4926923 | C | -0.19 | 0.03 | -0.02 | 0.02 | 0.01 | 0.01 |
| 234 | rs4930295 | G | -0.24 | 0.02 | -0.05 | 0.01 | 0.03 | 0.01 |
| 235 | rs4936099 | A | 0.17 | 0.02 | 0.00 | 0.01 | 0.02 | 0.01 |
| 236 | rs4952668 | A | -0.19 | 0.02 | -0.02 | 0.01 | 0.00 | 0.01 |
| 237 | rs4954192 | T | -0.12 | 0.02 | 0.01 | 0.01 | -0.02 | 0.01 |
| 238 | rs5012479 | G | -0.10 | 0.02 | 0.00 | 0.01 | 0.00 | 0.01 |
| 239 | rs504217 | T | 0.27 | 0.03 | -0.02 | 0.02 | -0.02 | 0.02 |
| 240 | rs504691 | A | -0.12 | 0.02 | -0.03 | 0.01 | 0.00 | 0.01 |
| 241 | rs509067 | C | 0.14 | 0.02 | -0.01 | 0.01 | -0.02 | 0.01 |
| 242 | rs520592 | G | 0.17 | 0.02 | 0.04 | 0.01 | -0.01 | 0.01 |
| 243 | rs55684003 | G | -0.12 | 0.02 | -0.01 | 0.01 | 0.00 | 0.01 |
| 244 | rs55770741 | T | -0.13 | 0.02 | -0.03 | 0.01 | 0.02 | 0.01 |
| 245 | rs55857306 | A | -0.52 | 0.02 | -0.02 | 0.01 | 0.02 | 0.01 |
| 246 | rs55944332 | G | 0.24 | 0.02 | -0.03 | 0.01 | 0.00 | 0.01 |
| 247 | rs55993676 | T | -0.21 | 0.02 | -0.01 | 0.01 | -0.01 | 0.01 |
| 248 | rs56809883 | T | 0.11 | 0.02 | 0.01 | 0.01 | 0.00 | 0.01 |
| 249 | rs58693787 | G | -0.16 | 0.02 | -0.01 | 0.01 | 0.01 | 0.01 |
| 250 | rs5992929 | T | 0.17 | 0.02 | 0.02 | 0.01 | 0.02 | 0.01 |
| 251 | rs602521 | A | 0.14 | 0.02 | 0.01 | 0.01 | 0.01 | 0.01 |
| 252 | rs6026739 | T | 0.50 | 0.03 | 0.05 | 0.01 | -0.01 | 0.01 |
| 253 | rs6031431 | G | 0.12 | 0.02 | 0.00 | 0.01 | 0.00 | 0.01 |
| 254 | rs604723 | C | 0.38 | 0.02 | 0.03 | 0.01 | -0.01 | 0.01 |
| 255 | rs6058261 | A | -0.12 | 0.02 | 0.00 | 0.01 | 0.01 | 0.01 |
| 256 | rs6078393 | G | -0.12 | 0.02 | 0.00 | 0.01 | 0.01 | 0.01 |
| 257 | rs6108168 | A | -0.19 | 0.02 | 0.00 | 0.01 | 0.00 | 0.01 |
| 258 | rs61789369 | G | 0.30 | 0.04 | 0.00 | 0.02 | -0.01 | 0.02 |
| 259 | rs61912333 | G | -0.12 | 0.02 | 0.00 | 0.01 | -0.01 | 0.01 |
| 260 | rs61948065 | C | 0.17 | 0.03 | 0.03 | 0.01 | 0.00 | 0.01 |
| 261 | rs62155750 | G | 0.22 | 0.02 | 0.01 | 0.01 | 0.00 | 0.01 |
| 262 | rs62158170 | G | -0.16 | 0.02 | -0.01 | 0.01 | -0.01 | 0.01 |
| 263 | rs62234672 | A | 0.12 | 0.02 | 0.02 | 0.01 | 0.00 | 0.01 |
| 264 | rs62301873 | G | 0.17 | 0.03 | 0.01 | 0.01 | 0.00 | 0.01 |
| 265 | rs62434124 | T | -0.49 | 0.03 | -0.05 | 0.02 | 0.00 | 0.02 |
| 266 | rs62503324 | T | 0.20 | 0.02 | -0.01 | 0.01 | 0.01 | 0.01 |
| 267 | rs636202 | C | -0.10 | 0.02 | -0.01 | 0.01 | 0.01 | 0.01 |
| 268 | rs6464165 | C | 0.22 | 0.02 | 0.01 | 0.01 | 0.02 | 0.01 |
| 269 | rs6487076 | G | -0.17 | 0.02 | 0.00 | 0.01 | 0.00 | 0.01 |
| 270 | rs6546810 | C | 0.12 | 0.02 | 0.02 | 0.01 | -0.01 | 0.01 |
| 271 | rs6556384 | A | -0.15 | 0.02 | -0.03 | 0.01 | 0.02 | 0.01 |
| 272 | rs6580970 | T | -0.17 | 0.02 | -0.02 | 0.01 | 0.02 | 0.01 |
| 273 | rs66682451 | G | -0.13 | 0.02 | -0.02 | 0.01 | 0.00 | 0.01 |
| 274 | rs6686889 | T | 0.19 | 0.02 | 0.03 | 0.01 | 0.00 | 0.01 |
| 275 | rs66887589 | C | 0.16 | 0.02 | 0.03 | 0.01 | 0.00 | 0.01 |
| 276 | rs6715901 | A | -0.14 | 0.02 | 0.00 | 0.01 | 0.02 | 0.01 |
| 277 | rs672272 | T | -0.19 | 0.02 | -0.01 | 0.01 | -0.02 | 0.01 |
| 278 | rs6735275 | C | -0.12 | 0.02 | -0.01 | 0.01 | -0.02 | 0.01 |
| 279 | rs6763931 | A | 0.14 | 0.02 | -0.02 | 0.01 | 0.03 | 0.01 |
| 280 | rs6777317 | A | 0.12 | 0.02 | 0.00 | 0.01 | 0.00 | 0.01 |
| 281 | rs6779368 | G | 0.18 | 0.02 | 0.00 | 0.01 | -0.02 | 0.01 |
| 282 | rs6795735 | T | -0.14 | 0.02 | 0.01 | 0.01 | 0.06 | 0.01 |
| 283 | rs6800730 | G | 0.25 | 0.02 | 0.02 | 0.01 | -0.02 | 0.01 |
| 284 | rs68085857 | T | 0.19 | 0.02 | 0.02 | 0.01 | 0.00 | 0.01 |
| 285 | rs682681 | C | 0.15 | 0.02 | -0.01 | 0.01 | 0.00 | 0.01 |
| 286 | rs6875967 | G | -0.13 | 0.02 | -0.02 | 0.01 | 0.00 | 0.01 |
| 287 | rs6934891 | A | 0.13 | 0.02 | 0.00 | 0.01 | 0.00 | 0.01 |
| 288 | rs6961048 | G | 0.27 | 0.03 | 0.01 | 0.02 | -0.02 | 0.01 |
| 289 | rs6983239 | T | 0.12 | 0.02 | 0.01 | 0.01 | -0.03 | 0.01 |
| 290 | rs7012891 | C | 0.14 | 0.02 | 0.03 | 0.01 | -0.02 | 0.01 |
| 291 | rs710249 | C | 0.15 | 0.02 | 0.02 | 0.01 | 0.02 | 0.01 |
| 292 | rs7106104 | C | 0.12 | 0.02 | 0.03 | 0.01 | -0.01 | 0.01 |
| 293 | rs710698 | G | -0.11 | 0.02 | -0.01 | 0.01 | 0.00 | 0.01 |
| 294 | rs7115331 | G | 0.13 | 0.02 | -0.02 | 0.01 | 0.02 | 0.01 |
| 295 | rs7132012 | G | -0.16 | 0.02 | -0.01 | 0.01 | 0.02 | 0.01 |
| 296 | rs7137828 | T | -0.50 | 0.02 | -0.06 | 0.01 | 0.02 | 0.01 |
| 297 | rs7192407 | C | -0.10 | 0.02 | 0.00 | 0.01 | 0.00 | 0.01 |
| 298 | rs7217916 | G | -0.11 | 0.02 | -0.01 | 0.01 | 0.02 | 0.01 |
| 299 | rs722783 | A | -0.21 | 0.02 | 0.01 | 0.01 | -0.01 | 0.01 |
| 300 | rs7235890 | T | -0.17 | 0.03 | 0.00 | 0.02 | -0.01 | 0.01 |
| 301 | rs7257694 | T | 0.18 | 0.02 | -0.02 | 0.01 | -0.01 | 0.01 |
| 302 | rs7259285 | A | 0.11 | 0.02 | 0.01 | 0.01 | -0.02 | 0.01 |
| 303 | rs7265695 | C | -0.20 | 0.02 | -0.02 | 0.01 | 0.00 | 0.01 |
| 304 | rs72719149 | C | 0.13 | 0.02 | 0.01 | 0.01 | -0.03 | 0.01 |
| 305 | rs7278003 | C | 0.13 | 0.02 | 0.03 | 0.01 | -0.02 | 0.01 |
| 306 | rs72831343 | G | -0.49 | 0.02 | -0.02 | 0.02 | 0.00 | 0.01 |
| 307 | rs72842207 | T | -0.21 | 0.02 | -0.01 | 0.01 | 0.02 | 0.01 |
| 308 | rs72976750 | C | 0.17 | 0.03 | 0.01 | 0.01 | 0.00 | 0.01 |
| 309 | rs7299936 | A | 0.18 | 0.02 | 0.01 | 0.01 | 0.01 | 0.01 |
| 310 | rs73036520 | C | 0.16 | 0.02 | 0.04 | 0.01 | -0.03 | 0.01 |
| 311 | rs7321688 | A | 0.15 | 0.02 | 0.01 | 0.01 | 0.01 | 0.01 |
| 312 | rs7324697 | A | 0.10 | 0.02 | -0.01 | 0.01 | 0.03 | 0.01 |
| 313 | rs73276406 | C | 0.16 | 0.02 | 0.03 | 0.01 | -0.03 | 0.01 |
| 314 | rs7350752 | A | -0.15 | 0.03 | 0.04 | 0.02 | -0.01 | 0.01 |
| 315 | rs7427249 | A | -0.11 | 0.02 | -0.01 | 0.01 | 0.00 | 0.01 |
| 316 | rs751984 | C | -0.39 | 0.03 | -0.02 | 0.01 | 0.04 | 0.01 |
| 317 | rs7524019 | T | 0.10 | 0.02 | 0.00 | 0.01 | 0.01 | 0.01 |
| 318 | rs75507123 | T | -0.14 | 0.03 | 0.01 | 0.01 | -0.02 | 0.01 |
| 319 | rs7569128 | A | 0.20 | 0.02 | 0.02 | 0.01 | 0.01 | 0.01 |
| 320 | rs7572130 | G | 0.18 | 0.03 | 0.04 | 0.01 | 0.01 | 0.01 |
| 321 | rs7576060 | T | -0.10 | 0.02 | -0.03 | 0.01 | 0.00 | 0.01 |
| 322 | rs7611674 | G | -0.16 | 0.02 | 0.01 | 0.01 | -0.01 | 0.01 |
| 323 | rs7623706 | G | -0.10 | 0.02 | -0.01 | 0.01 | -0.01 | 0.01 |
| 324 | rs76326501 | C | -0.36 | 0.03 | -0.02 | 0.02 | 0.01 | 0.01 |
| 325 | rs7694000 | T | 0.10 | 0.02 | -0.01 | 0.01 | -0.01 | 0.01 |
| 326 | rs76954792 | T | 0.12 | 0.02 | 0.03 | 0.01 | 0.00 | 0.01 |
| 327 | rs7788746 | T | -0.16 | 0.02 | -0.03 | 0.01 | -0.02 | 0.01 |
| 328 | rs7800558 | C | -0.10 | 0.02 | 0.00 | 0.01 | 0.01 | 0.01 |
| 329 | rs7805035 | A | 0.13 | 0.02 | 0.01 | 0.01 | 0.00 | 0.01 |
| 330 | rs78151625 | C | 0.19 | 0.02 | 0.01 | 0.01 | -0.01 | 0.01 |
| 331 | rs786921 | A | -0.11 | 0.02 | -0.01 | 0.01 | 0.01 | 0.01 |
| 332 | rs78809139 | A | -0.23 | 0.03 | 0.03 | 0.02 | 0.00 | 0.01 |
| 333 | rs78909293 | C | -0.32 | 0.04 | -0.06 | 0.02 | 0.03 | 0.02 |
| 334 | rs79044887 | G | -0.24 | 0.02 | -0.05 | 0.01 | 0.01 | 0.01 |
| 335 | rs7926335 | T | 0.18 | 0.02 | 0.02 | 0.01 | -0.01 | 0.01 |
| 336 | rs7933758 | T | -0.11 | 0.02 | -0.01 | 0.01 | 0.00 | 0.01 |
| 337 | rs7959649 | C | -0.12 | 0.02 | -0.01 | 0.01 | 0.01 | 0.01 |
| 338 | rs7967705 | C | -0.27 | 0.02 | 0.00 | 0.01 | 0.00 | 0.01 |
| 339 | rs79724577 | C | -0.14 | 0.02 | 0.04 | 0.01 | -0.01 | 0.01 |
| 340 | rs7992292 | A | 0.14 | 0.02 | 0.02 | 0.01 | -0.02 | 0.01 |
| 341 | rs8014182 | T | -0.19 | 0.03 | -0.02 | 0.01 | 0.03 | 0.01 |
| 342 | rs8078510 | A | -0.13 | 0.02 | -0.05 | 0.01 | 0.06 | 0.01 |
| 343 | rs824523 | A | 0.12 | 0.02 | -0.01 | 0.01 | -0.01 | 0.01 |
| 344 | rs881858 | A | 0.16 | 0.02 | 0.01 | 0.01 | 0.05 | 0.01 |
| 345 | rs882624 | T | -0.16 | 0.02 | 0.02 | 0.01 | 0.01 | 0.01 |
| 346 | rs917522 | T | 0.17 | 0.03 | 0.01 | 0.01 | 0.01 | 0.01 |
| 347 | rs9286351 | G | 0.14 | 0.02 | 0.02 | 0.01 | 0.00 | 0.01 |
| 348 | rs9326869 | C | -0.11 | 0.02 | 0.00 | 0.01 | 0.02 | 0.01 |
| 349 | rs9368 | A | 0.11 | 0.02 | 0.00 | 0.01 | 0.01 | 0.01 |
| 350 | rs9399137 | C | -0.11 | 0.02 | -0.02 | 0.01 | 0.00 | 0.01 |
| 351 | rs9406076 | T | 0.10 | 0.02 | 0.02 | 0.01 | 0.02 | 0.01 |
| 352 | rs9419374 | G | -0.12 | 0.02 | -0.01 | 0.01 | 0.00 | 0.01 |
| 353 | rs9467545 | T | 0.25 | 0.02 | 0.00 | 0.01 | -0.01 | 0.01 |
| 354 | rs9478282 | T | -0.20 | 0.03 | -0.01 | 0.01 | -0.02 | 0.01 |
| 355 | rs951914 | C | 0.19 | 0.02 | 0.01 | 0.01 | -0.01 | 0.01 |
| 356 | rs9526707 | A | -0.12 | 0.02 | -0.01 | 0.01 | -0.01 | 0.01 |
| 357 | rs962369 | C | -0.17 | 0.02 | 0.02 | 0.01 | -0.03 | 0.01 |
| 358 | rs964941 | A | 0.17 | 0.02 | -0.01 | 0.01 | 0.01 | 0.01 |
| 359 | rs9791312 | C | 0.12 | 0.02 | 0.00 | 0.01 | -0.01 | 0.01 |
| 360 | rs9841978 | A | 0.18 | 0.02 | 0.00 | 0.01 | -0.02 | 0.01 |
| 361 | rs9889262 | A | 0.23 | 0.02 | 0.04 | 0.01 | -0.01 | 0.01 |
| 362 | rs9893005 | G | 0.12 | 0.02 | 0.00 | 0.01 | 0.01 | 0.01 |
| 363 | rs9900637 | A | 0.10 | 0.02 | 0.01 | 0.01 | -0.03 | 0.01 |
| 364 | rs990619 | G | 0.16 | 0.02 | 0.04 | 0.01 | 0.00 | 0.01 |
| 365 | rs9918907 | G | 0.12 | 0.02 | 0.02 | 0.01 | 0.01 | 0.01 |
| 366 | rs9932220 | A | -0.16 | 0.02 | -0.01 | 0.01 | -0.01 | 0.01 |
| 367 | rs9937801 | C | -0.16 | 0.02 | -0.03 | 0.01 | 0.00 | 0.01 |

DBP: Diastolic blood pressure; CHD: Coronary heart disease; T2DM: Type-2 diabetes

Table 33: Genetic association estimates for the effect of TG on CHD, adjusted for T2DM. ea=effect allele, gx=TG, gy=CHD, gz=T2DM, se=standard error

|  | SNP | ea | gx | gx_se | gy | gy_se | gz | gz_se |
| --- | --- | --- | --- | --- | --- | --- | --- | --- |
| 1 | rs10040328 | A | 0.02 | 0.00 | 0.02 | 0.01 | -0.02 | 0.01 |
| 2 | rs1009590 | C | 0.02 | 0.00 | 0.00 | 0.02 | -0.03 | 0.01 |
| 3 | rs1044808 | C | -0.03 | 0.00 | -0.03 | 0.02 | 0.05 | 0.01 |
| 4 | rs1045241 | T | -0.02 | 0.00 | -0.01 | 0.01 | 0.02 | 0.01 |
| 5 | rs10750766 | A | 0.02 | 0.00 | 0.03 | 0.01 | -0.03 | 0.01 |
| 6 | rs10772947 | G | -0.01 | 0.00 | 0.00 | 0.01 | 0.01 | 0.01 |
| 7 | rs10797119 | C | 0.02 | 0.00 | 0.02 | 0.01 | -0.01 | 0.01 |
| 8 | rs10838681 | A | -0.03 | 0.00 | 0.00 | 0.01 | 0.00 | 0.01 |
| 9 | rs10851698 | T | 0.02 | 0.00 | 0.02 | 0.01 | -0.02 | 0.01 |
| 10 | rs10872003 | A | -0.01 | 0.00 | 0.00 | 0.01 | 0.00 | 0.01 |
| 11 | rs10883026 | T | -0.02 | 0.00 | -0.02 | 0.01 | 0.02 | 0.01 |
| 12 | rs10957299 | G | -0.01 | 0.00 | -0.01 | 0.01 | 0.02 | 0.01 |
| 13 | rs11030107 | G | 0.02 | 0.00 | 0.03 | 0.01 | -0.03 | 0.01 |
| 14 | rs11045171 | G | -0.03 | 0.00 | 0.00 | 0.01 | 0.02 | 0.01 |
| 15 | rs11122450 | G | -0.05 | 0.00 | -0.03 | 0.01 | 0.01 | 0.01 |
| 16 | rs11187019 | G | -0.01 | 0.00 | -0.01 | 0.01 | 0.08 | 0.01 |
| 17 | rs11206374 | A | 0.03 | 0.00 | 0.02 | 0.01 | -0.06 | 0.01 |
| 18 | rs112424890 | T | 0.02 | 0.00 | 0.03 | 0.01 | -0.01 | 0.01 |
| 19 | rs1128249 | T | -0.04 | 0.00 | -0.02 | 0.01 | 0.07 | 0.01 |
| 20 | rs115271198 | T | -0.03 | 0.00 | -0.02 | 0.02 | 0.05 | 0.02 |
| 21 | rs11631625 | G | 0.01 | 0.00 | 0.02 | 0.01 | -0.02 | 0.01 |
| 22 | rs11705483 | A | 0.02 | 0.00 | 0.04 | 0.02 | -0.05 | 0.01 |
| 23 | rs11722924 | C | 0.01 | 0.00 | -0.01 | 0.01 | 0.00 | 0.01 |
| 24 | rs11752394 | G | 0.02 | 0.00 | 0.00 | 0.01 | -0.02 | 0.01 |
| 25 | rs12088739 | G | -0.03 | 0.00 | -0.05 | 0.02 | 0.09 | 0.01 |
| 26 | rs12119979 | G | 0.02 | 0.00 | 0.01 | 0.01 | -0.04 | 0.01 |
| 27 | rs12424054 | A | 0.02 | 0.00 | 0.00 | 0.01 | -0.02 | 0.01 |
| 28 | rs12446515 | T | -0.04 | 0.00 | -0.03 | 0.01 | 0.01 | 0.01 |
| 29 | rs12480662 | T | -0.02 | 0.00 | -0.01 | 0.01 | -0.01 | 0.01 |
| 30 | rs12513202 | T | 0.01 | 0.00 | -0.01 | 0.01 | -0.02 | 0.01 |
| 31 | rs12541912 | C | -0.10 | 0.00 | -0.03 | 0.01 | 0.03 | 0.01 |
| 32 | rs12686780 | T | 0.02 | 0.00 | 0.00 | 0.01 | -0.03 | 0.01 |
| 33 | rs12749691 | T | -0.02 | 0.00 | -0.01 | 0.01 | -0.01 | 0.01 |
| 34 | rs1279840 | C | 0.03 | 0.00 | 0.03 | 0.01 | -0.03 | 0.01 |
| 35 | rs12868517 | G | -0.01 | 0.00 | 0.00 | 0.01 | 0.03 | 0.01 |
| 36 | rs12880341 | C | 0.02 | 0.00 | 0.01 | 0.01 | -0.02 | 0.01 |
| 37 | rs1292065 | G | -0.02 | 0.00 | -0.02 | 0.01 | 0.00 | 0.01 |
| 38 | rs12928099 | A | -0.03 | 0.00 | -0.02 | 0.01 | 0.02 | 0.01 |
| 39 | rs13234131 | G | -0.13 | 0.00 | 0.01 | 0.02 | -0.03 | 0.01 |
| 40 | rs13269725 | G | 0.04 | 0.00 | 0.02 | 0.02 | -0.05 | 0.01 |
| 41 | rs13273454 | T | -0.06 | 0.00 | -0.02 | 0.01 | 0.02 | 0.01 |
| 42 | rs1340819 | C | -0.01 | 0.00 | 0.00 | 0.01 | 0.01 | 0.01 |
| 43 | rs1420384 | T | -0.01 | 0.00 | 0.00 | 0.01 | 0.03 | 0.01 |
| 44 | rs142047875 | T | -0.01 | 0.00 | -0.02 | 0.01 | 0.02 | 0.01 |
| 45 | rs1471251 | T | 0.04 | 0.00 | 0.01 | 0.01 | -0.01 | 0.01 |
| 46 | rs1473886 | T | -0.02 | 0.00 | 0.02 | 0.01 | 0.00 | 0.01 |
| 47 | rs149142833 | T | 0.02 | 0.00 | 0.00 | 0.01 | -0.04 | 0.01 |
| 48 | rs154735 | A | 0.03 | 0.00 | 0.00 | 0.02 | -0.01 | 0.02 |
| 49 | rs1549293 | T | -0.02 | 0.00 | -0.01 | 0.01 | 0.01 | 0.01 |
| 50 | rs1644005 | C | -0.01 | 0.00 | 0.00 | 0.01 | 0.04 | 0.01 |
| 51 | rs1684608 | A | 0.02 | 0.00 | -0.01 | 0.01 | 0.00 | 0.01 |
| 52 | rs1688043 | T | 0.03 | 0.00 | -0.02 | 0.02 | 0.01 | 0.01 |
| 53 | rs17052058 | G | -0.03 | 0.00 | 0.00 | 0.01 | 0.02 | 0.01 |
| 54 | rs17138358 | C | 0.02 | 0.00 | -0.02 | 0.01 | 0.00 | 0.01 |
| 55 | rs17311740 | T | -0.03 | 0.00 | 0.02 | 0.02 | 0.00 | 0.02 |
| 56 | rs17326656 | T | 0.02 | 0.00 | 0.01 | 0.01 | -0.01 | 0.01 |
| 57 | rs174574 | C | -0.05 | 0.00 | 0.02 | 0.01 | -0.02 | 0.01 |
| 58 | rs17496249 | G | -0.01 | 0.00 | -0.02 | 0.01 | 0.03 | 0.01 |
| 59 | rs17699425 | A | -0.03 | 0.00 | 0.05 | 0.02 | -0.03 | 0.02 |
| 60 | rs1790099 | T | 0.02 | 0.00 | 0.01 | 0.01 | -0.04 | 0.01 |
| 61 | rs1800978 | G | -0.03 | 0.00 | -0.03 | 0.01 | 0.02 | 0.01 |
| 62 | rs1853413 | G | -0.01 | 0.00 | -0.01 | 0.01 | 0.01 | 0.01 |
| 63 | rs1924485 | T | -0.02 | 0.00 | 0.00 | 0.01 | -0.01 | 0.01 |
| 64 | rs1928496 | T | 0.02 | 0.00 | -0.01 | 0.01 | 0.02 | 0.01 |
| 65 | rs199607859 | T | -0.03 | 0.00 | -0.01 | 0.01 | 0.03 | 0.01 |
| 66 | rs199795230 | T | 0.03 | 0.00 | -0.03 | 0.01 | 0.01 | 0.01 |
| 67 | rs2068888 | A | -0.03 | 0.00 | -0.04 | 0.01 | 0.00 | 0.01 |
| 68 | rs2070971 | T | 0.02 | 0.00 | 0.02 | 0.01 | -0.02 | 0.01 |
| 69 | rs2071887 | A | 0.02 | 0.00 | -0.02 | 0.01 | 0.00 | 0.01 |
| 70 | rs2081687 | C | -0.03 | 0.00 | 0.00 | 0.01 | -0.01 | 0.01 |
| 71 | rs2114273 | C | 0.02 | 0.00 | 0.00 | 0.01 | 0.00 | 0.01 |
| 72 | rs213479 | T | -0.02 | 0.00 | 0.00 | 0.01 | -0.01 | 0.01 |
| 73 | rs2139980 | A | -0.01 | 0.00 | -0.01 | 0.01 | 0.03 | 0.01 |
| 74 | rs2267373 | T | 0.02 | 0.00 | 0.00 | 0.01 | -0.01 | 0.01 |
| 75 | rs2510344 | C | -0.02 | 0.00 | -0.01 | 0.01 | 0.03 | 0.01 |
| 76 | rs261342 | C | -0.05 | 0.00 | -0.03 | 0.01 | 0.00 | 0.01 |
| 77 | rs2652812 | T | -0.02 | 0.00 | -0.02 | 0.01 | 0.02 | 0.01 |
| 78 | rs2694913 | C | 0.01 | 0.00 | -0.01 | 0.01 | 0.00 | 0.01 |
| 79 | rs2699805 | A | -0.02 | 0.00 | -0.03 | 0.01 | 0.03 | 0.01 |
| 80 | rs2723067 | G | -0.02 | 0.00 | 0.01 | 0.01 | 0.05 | 0.01 |
| 81 | rs2773469 | G | -0.02 | 0.00 | 0.01 | 0.01 | 0.02 | 0.01 |
| 82 | rs2925979 | C | -0.03 | 0.00 | -0.02 | 0.01 | 0.05 | 0.01 |
| 83 | rs2943645 | T | 0.04 | 0.00 | 0.03 | 0.01 | -0.09 | 0.01 |
| 84 | rs2954017 | C | -0.09 | 0.00 | -0.04 | 0.01 | 0.00 | 0.01 |
| 85 | rs296884 | T | -0.02 | 0.00 | -0.01 | 0.01 | 0.01 | 0.01 |
| 86 | rs2971669 | T | 0.02 | 0.00 | 0.01 | 0.01 | -0.05 | 0.01 |
| 87 | rs34682685 | A | 0.03 | 0.00 | 0.02 | 0.02 | -0.01 | 0.01 |
| 88 | rs35859536 | T | -0.02 | 0.00 | -0.01 | 0.01 | 0.12 | 0.01 |
| 89 | rs3731696 | G | 0.02 | 0.00 | -0.01 | 0.01 | -0.04 | 0.01 |
| 90 | rs38205 | C | -0.01 | 0.00 | -0.02 | 0.01 | 0.04 | 0.01 |
| 91 | rs3826043 | T | -0.01 | 0.00 | -0.02 | 0.01 | 0.03 | 0.01 |
| 92 | rs41749 | A | -0.01 | 0.00 | -0.01 | 0.01 | 0.01 | 0.01 |
| 93 | rs4410790 | C | 0.02 | 0.00 | 0.03 | 0.01 | -0.02 | 0.01 |
| 94 | rs45487899 | T | -0.04 | 0.01 | -0.06 | 0.03 | 0.01 | 0.02 |
| 95 | rs4646246 | G | 0.03 | 0.00 | 0.04 | 0.01 | -0.03 | 0.01 |
| 96 | rs4675812 | A | -0.01 | 0.00 | 0.01 | 0.01 | 0.01 | 0.01 |
| 97 | rs4710938 | G | -0.01 | 0.00 | 0.00 | 0.01 | 0.07 | 0.01 |
| 98 | rs4731701 | T | -0.03 | 0.00 | -0.02 | 0.01 | 0.06 | 0.01 |
| 99 | rs4760254 | C | -0.03 | 0.00 | -0.01 | 0.01 | 0.01 | 0.01 |
| 100 | rs4761234 | C | -0.02 | 0.00 | 0.00 | 0.01 | 0.01 | 0.01 |
| 101 | rs4789182 | A | 0.02 | 0.00 | 0.00 | 0.01 | -0.01 | 0.01 |
| 102 | rs4841580 | C | -0.02 | 0.00 | 0.01 | 0.01 | -0.03 | 0.01 |
| 103 | rs4843754 | G | 0.01 | 0.00 | 0.00 | 0.01 | -0.02 | 0.01 |
| 104 | rs4930724 | C | -0.03 | 0.00 | -0.02 | 0.01 | 0.03 | 0.01 |
| 105 | rs4969179 | G | -0.02 | 0.00 | -0.01 | 0.01 | 0.02 | 0.01 |
| 106 | rs5005705 | A | -0.02 | 0.00 | -0.02 | 0.01 | 0.04 | 0.01 |
| 107 | rs56030759 | C | 0.04 | 0.00 | 0.01 | 0.02 | -0.01 | 0.02 |
| 108 | rs57232565 | T | 0.19 | 0.01 | 0.05 | 0.02 | 0.00 | 0.01 |
| 109 | rs5755799 | G | 0.01 | 0.00 | 0.01 | 0.01 | -0.02 | 0.01 |
| 110 | rs58284370 | A | 0.03 | 0.00 | -0.01 | 0.02 | 0.01 | 0.01 |
| 111 | rs58542926 | T | -0.11 | 0.00 | -0.05 | 0.02 | -0.09 | 0.01 |
| 112 | rs591939 | G | 0.02 | 0.00 | 0.03 | 0.01 | -0.05 | 0.01 |
| 113 | rs593979 | C | -0.02 | 0.00 | -0.02 | 0.01 | 0.03 | 0.01 |
| 114 | rs6066138 | A | -0.02 | 0.00 | -0.01 | 0.01 | 0.05 | 0.01 |
| 115 | rs6073958 | C | 0.06 | 0.00 | -0.03 | 0.01 | 0.02 | 0.01 |
| 116 | rs60856912 | T | 0.03 | 0.00 | 0.02 | 0.01 | -0.04 | 0.01 |
| 117 | rs6093446 | A | 0.01 | 0.00 | 0.04 | 0.01 | -0.01 | 0.01 |
| 118 | rs62102718 | T | 0.02 | 0.00 | 0.03 | 0.01 | -0.04 | 0.01 |
| 119 | rs62112763 | G | 0.02 | 0.00 | 0.00 | 0.01 | -0.02 | 0.01 |
| 120 | rs631106 | A | -0.08 | 0.00 | -0.01 | 0.01 | 0.00 | 0.01 |
| 121 | rs6486122 | T | 0.02 | 0.00 | 0.03 | 0.01 | -0.03 | 0.01 |
| 122 | rs6492721 | C | -0.01 | 0.00 | 0.00 | 0.01 | -0.03 | 0.01 |
| 123 | rs6506033 | T | -0.03 | 0.00 | 0.00 | 0.02 | 0.02 | 0.02 |
| 124 | rs6547692 | A | -0.09 | 0.00 | 0.00 | 0.01 | -0.05 | 0.01 |
| 125 | rs6554198 | A | -0.01 | 0.00 | -0.01 | 0.01 | 0.00 | 0.01 |
| 126 | rs6708784 | G | -0.01 | 0.00 | 0.01 | 0.01 | 0.04 | 0.01 |
| 127 | rs676210 | A | -0.08 | 0.00 | 0.00 | 0.01 | -0.01 | 0.01 |
| 128 | rs6882076 | C | 0.04 | 0.00 | 0.01 | 0.01 | 0.02 | 0.01 |
| 129 | rs6916318 | T | 0.03 | 0.00 | 0.01 | 0.01 | 0.01 | 0.01 |
| 130 | rs7117238 | A | -0.02 | 0.00 | -0.03 | 0.01 | 0.01 | 0.01 |
| 131 | rs7123454 | A | -0.13 | 0.00 | 0.00 | 0.01 | 0.01 | 0.01 |
| 132 | rs71603401 | G | 0.03 | 0.00 | 0.03 | 0.01 | -0.02 | 0.01 |
| 133 | rs7167078 | G | -0.02 | 0.00 | 0.02 | 0.01 | 0.03 | 0.01 |
| 134 | rs7274718 | A | 0.02 | 0.00 | -0.01 | 0.01 | 0.01 | 0.01 |
| 135 | rs729761 | G | 0.02 | 0.00 | 0.01 | 0.01 | 0.05 | 0.01 |
| 136 | rs7298844 | G | 0.02 | 0.00 | -0.02 | 0.01 | -0.03 | 0.01 |
| 137 | rs73025562 | A | 0.02 | 0.00 | -0.02 | 0.01 | 0.00 | 0.01 |
| 138 | rs73243877 | G | 0.03 | 0.00 | 0.01 | 0.01 | -0.02 | 0.01 |
| 139 | rs7529073 | C | -0.01 | 0.00 | 0.00 | 0.01 | 0.05 | 0.01 |
| 140 | rs76669111 | T | -0.02 | 0.00 | 0.00 | 0.01 | -0.02 | 0.01 |
| 141 | rs7735249 | G | 0.03 | 0.00 | 0.06 | 0.02 | -0.04 | 0.01 |
| 142 | rs7826687 | G | 0.03 | 0.00 | 0.01 | 0.01 | -0.02 | 0.01 |
| 143 | rs7896783 | A | -0.03 | 0.00 | 0.01 | 0.01 | 0.01 | 0.01 |
| 144 | rs79291519 | T | -0.06 | 0.01 | -0.01 | 0.02 | 0.02 | 0.02 |
| 145 | rs863750 | T | 0.03 | 0.00 | 0.03 | 0.01 | -0.05 | 0.01 |
| 146 | rs867939 | A | -0.02 | 0.00 | -0.01 | 0.01 | 0.02 | 0.01 |
| 147 | rs917195 | T | -0.02 | 0.00 | -0.01 | 0.01 | 0.05 | 0.01 |
| 148 | rs9425589 | A | -0.01 | 0.00 | -0.02 | 0.01 | 0.04 | 0.01 |
| 149 | rs9692598 | G | -0.01 | 0.00 | 0.00 | 0.01 | 0.01 | 0.01 |
| 150 | rs9836434 | T | 0.01 | 0.00 | 0.00 | 0.01 | -0.02 | 0.01 |
| 151 | rs9844972 | C | 0.04 | 0.00 | 0.04 | 0.02 | -0.10 | 0.01 |

TG: Triglycerides; CHD: Coronary heart disease; T2DM: Type-2 diabetes

Table 34: Genetic association estimates for the effect of HDL on CHD, adjusted for T2DM. ea=effect allele, gx=HDL, gy=CHD, gz=T2DM, se=standard error

|  | SNP | ea | gx | gx_se | gy | gy_se | gz | gz_se |
| --- | --- | --- | --- | --- | --- | --- | --- | --- |
| 1 | rs117199990 | T | 0.19 | 0.02 | -0.05 | 0.02 | 0.06 | 0.01 |
| 2 | rs1532085 | G | -0.10 | 0.02 | -0.02 | 0.01 | -0.02 | 0.01 |
| 3 | rs183130 | T | 0.24 | 0.02 | -0.03 | 0.01 | 0.01 | 0.01 |
| 4 | rs964184 | C | 0.14 | 0.02 | -0.05 | 0.01 | 0.03 | 0.01 |

HDL: High-density lipoprotein; CHD: Coronary heart disease; T2DM: Type-2 diabetes

Table 35: Genetic association estimates for the effect of WHR on CHD, adjusted for T2DM. ea=effect allele, gx=WHR, gy=CHD, gz=T2DM, se=standard error

|  | SNP | ea | gx | gx_se | gy | gy_se | gz | gz_se |
| --- | --- | --- | --- | --- | --- | --- | --- | --- |
| 1 | rs10245353 | A | 0.04 | 0.00 | 0.01 | 0.01 | -0.02 | 0.01 |
| 2 | rs10804591 | A | 0.02 | 0.00 | 0.03 | 0.01 | -0.03 | 0.01 |
| 3 | rs10842707 | T | 0.03 | 0.00 | 0.00 | 0.01 | -0.05 | 0.01 |
| 4 | rs10991437 | A | 0.03 | 0.01 | 0.01 | 0.01 | -0.02 | 0.01 |
| 5 | rs11231693 | A | 0.04 | 0.01 | 0.02 | 0.02 | -0.06 | 0.02 |
| 6 | rs1128249 | T | -0.03 | 0.00 | -0.02 | 0.01 | 0.07 | 0.01 |
| 7 | rs12143789 | C | 0.02 | 0.00 | -0.01 | 0.01 | 0.01 | 0.01 |
| 8 | rs12679556 | G | 0.03 | 0.00 | 0.01 | 0.01 | -0.03 | 0.01 |
| 9 | rs1294410 | C | 0.03 | 0.00 | -0.02 | 0.01 | 0.01 | 0.01 |
| 10 | rs1385167 | G | 0.03 | 0.00 | 0.02 | 0.01 | -0.02 | 0.01 |
| 11 | rs1440372 | C | 0.02 | 0.00 | 0.01 | 0.01 | -0.02 | 0.01 |
| 12 | rs1569135 | G | -0.02 | 0.00 | -0.02 | 0.01 | 0.01 | 0.01 |
| 13 | rs17451107 | C | -0.03 | 0.00 | 0.01 | 0.01 | -0.02 | 0.01 |
| 14 | rs17819328 | G | 0.02 | 0.00 | 0.03 | 0.01 | -0.03 | 0.01 |
| 15 | rs1936805 | T | 0.04 | 0.00 | 0.01 | 0.01 | 0.01 | 0.01 |
| 16 | rs2071449 | A | 0.03 | 0.00 | -0.02 | 0.01 | 0.02 | 0.01 |
| 17 | rs2276824 | G | -0.02 | 0.00 | 0.00 | 0.01 | 0.00 | 0.01 |
| 18 | rs2294239 | G | -0.03 | 0.00 | -0.02 | 0.01 | -0.01 | 0.01 |
| 19 | rs2645294 | T | 0.03 | 0.00 | -0.01 | 0.01 | 0.00 | 0.01 |
| 20 | rs2820443 | C | -0.04 | 0.00 | -0.01 | 0.01 | 0.05 | 0.01 |
| 21 | rs303084 | A | 0.02 | 0.00 | -0.01 | 0.01 | -0.04 | 0.01 |
| 22 | rs4081724 | A | -0.04 | 0.01 | -0.02 | 0.02 | -0.02 | 0.01 |
| 23 | rs459193 | G | -0.03 | 0.00 | 0.03 | 0.01 | -0.07 | 0.01 |
| 24 | rs4765219 | A | -0.03 | 0.00 | -0.03 | 0.01 | 0.03 | 0.01 |
| 25 | rs6772129 | G | -0.04 | 0.00 | 0.00 | 0.01 | 0.06 | 0.01 |
| 26 | rs714515 | A | -0.03 | 0.00 | -0.02 | 0.01 | 0.03 | 0.01 |
| 27 | rs7705502 | A | 0.03 | 0.00 | -0.01 | 0.01 | -0.01 | 0.01 |
| 28 | rs8030605 | A | 0.03 | 0.01 | -0.01 | 0.01 | -0.01 | 0.01 |
| 29 | rs878639 | G | -0.02 | 0.00 | -0.01 | 0.01 | -0.02 | 0.01 |
| 30 | rs979012 | C | -0.03 | 0.00 | 0.00 | 0.01 | 0.00 | 0.01 |

WHR: Waist-hip-ratio; CHD: Coronary heart disease; T2DM: Type-2 diabetes

Table 36: Genetic association estimates for the effect of insulin sensitivity on CHD, adjusted for T2DM. ea=effect allele, gx=insulin sensitivity, gy=CHD, gz=T2DM, se=standard error

|  | SNP | ea | gx | gx_se | gy | gy_se | gz | gz_se |
| --- | --- | --- | --- | --- | --- | --- | --- | --- |
| 1 | rs10483182 | A | -2 | 0.26 | 0.02 | 0.04 | -0.01 | 0.03 |
| 2 | rs10495667 | A | -0.9 | 0.18 | 0.00 | 0.03 | -0.02 | 0.02 |
| 3 | rs10506418 | A | -0.8 | 0.25 | 0.00 | 0.02 | 0.03 | 0.02 |
| 4 | rs11594101 | G | -1.6 | 0.25 | 0.01 | 0.04 | 0.04 | 0.03 |
| 5 | rs11790816 | T | -1.4 | 0.26 | -0.03 | 0.03 | 0.00 | 0.02 |
| 6 | rs12583553 | T | -1 | 0.19 | -0.01 | 0.02 | 0.02 | 0.03 |
| 7 | rs1857095 | T | 0.88 | 0.25 | 0.03 | 0.02 | 0.03 | 0.03 |
| 8 | rs2828537 | T | -1.1 | 0.17 | 0.03 | 0.02 | -0.01 | 0.02 |
| 9 | rs2972146 | T | 0.14 | 0.07 | 0.04 | 0.01 | -0.09 | 0.01 |
| 10 | rs4078023 | T | 1.3 | 0.23 | -0.01 | 0.03 | -0.06 | 0.04 |
| 11 | rs6027072 | A | -0.48 | 0.18 | 0.00 | 0.02 | 0.03 | 0.02 |

CHD: Coronary heart disease; T2DM: Type-2 diabetes

Table 37: Genetic association estimates for the effect of SBP on MI, adjusted for T2DM. ea=effect allele, gx=SBP, gy=MI, gz=T2DM, se=standard error

|  | SNP | ea | gx | gx_se | gy | gy_se | gz | gz_se |
| --- | --- | --- | --- | --- | --- | --- | --- | --- |
| 1 | rs1000423 | T | 0.41 | 0.03 | 0.02 | 0.01 | 0.00 | 0.01 |
| 2 | rs10008637 | C | -0.22 | 0.03 | 0.00 | 0.01 | 0.00 | 0.01 |
| 3 | rs10045307 | G | 0.20 | 0.04 | 0.00 | 0.01 | -0.01 | 0.01 |
| 4 | rs10048404 | T | -0.26 | 0.03 | -0.01 | 0.01 | 0.02 | 0.01 |
| 5 | rs1006545 | T | 0.68 | 0.05 | -0.02 | 0.02 | 0.03 | 0.01 |
| 6 | rs1010064 | C | -0.36 | 0.04 | -0.04 | 0.01 | 0.02 | 0.01 |
| 7 | rs1012089 | G | 0.19 | 0.03 | 0.00 | 0.01 | 0.01 | 0.01 |
| 8 | rs10188003 | T | 0.19 | 0.03 | -0.01 | 0.01 | 0.00 | 0.01 |
| 9 | rs10207726 | T | -0.21 | 0.03 | -0.01 | 0.01 | 0.02 | 0.01 |
| 10 | rs10224210 | C | 0.38 | 0.03 | 0.01 | 0.01 | 0.02 | 0.01 |
| 11 | rs1044822 | T | -0.25 | 0.04 | -0.01 | 0.02 | 0.02 | 0.01 |
| 12 | rs10460108 | G | -0.21 | 0.03 | 0.01 | 0.01 | 0.00 | 0.01 |
| 13 | rs1049212 | G | 0.30 | 0.03 | 0.01 | 0.01 | 0.01 | 0.01 |
| 14 | rs10501410 | A | 0.41 | 0.06 | -0.03 | 0.02 | -0.01 | 0.02 |
| 15 | rs1052501 | T | 0.23 | 0.04 | -0.02 | 0.01 | 0.01 | 0.01 |
| 16 | rs10749572 | T | -0.20 | 0.03 | -0.02 | 0.01 | 0.00 | 0.01 |
| 17 | rs10750441 | T | 0.18 | 0.03 | 0.00 | 0.01 | -0.01 | 0.01 |
| 18 | rs10777213 | A | -0.18 | 0.03 | -0.01 | 0.01 | 0.01 | 0.01 |
| 19 | rs10779795 | G | -0.22 | 0.03 | 0.00 | 0.01 | 0.01 | 0.01 |
| 20 | rs10782230 | A | 0.21 | 0.03 | -0.01 | 0.01 | -0.01 | 0.01 |
| 21 | rs10804330 | C | -0.24 | 0.03 | -0.01 | 0.01 | 0.07 | 0.01 |
| 22 | rs10914124 | C | -0.23 | 0.03 | 0.00 | 0.01 | 0.00 | 0.01 |
| 23 | rs10941043 | G | 0.26 | 0.03 | 0.00 | 0.01 | -0.01 | 0.01 |
| 24 | rs10980408 | C | 0.76 | 0.08 | 0.06 | 0.03 | -0.03 | 0.02 |
| 25 | rs11097909 | C | 0.36 | 0.04 | 0.01 | 0.02 | 0.00 | 0.01 |
| 26 | rs11120093 | T | -0.18 | 0.03 | -0.01 | 0.01 | -0.01 | 0.01 |
| 27 | rs11159091 | A | 0.20 | 0.03 | 0.00 | 0.01 | 0.01 | 0.01 |
| 28 | rs111866816 | T | 0.36 | 0.06 | 0.04 | 0.02 | -0.02 | 0.02 |
| 29 | rs11191580 | C | -1.10 | 0.06 | -0.07 | 0.02 | 0.00 | 0.01 |
| 30 | rs11210029 | G | 0.20 | 0.03 | 0.00 | 0.01 | 0.01 | 0.01 |
| 31 | rs11222084 | T | 0.34 | 0.03 | -0.01 | 0.01 | -0.02 | 0.01 |
| 32 | rs11241313 | T | -0.21 | 0.03 | -0.01 | 0.01 | 0.00 | 0.01 |
| 33 | rs112509803 | C | -0.26 | 0.05 | -0.01 | 0.02 | 0.01 | 0.01 |
| 34 | rs11252324 | T | -0.42 | 0.06 | -0.07 | 0.02 | -0.01 | 0.01 |
| 35 | rs115262049 | T | -0.59 | 0.06 | -0.03 | 0.02 | 0.02 | 0.01 |
| 36 | rs1154214 | G | 0.20 | 0.03 | 0.01 | 0.01 | 0.00 | 0.01 |
| 37 | rs11585169 | A | 0.18 | 0.03 | 0.02 | 0.01 | -0.02 | 0.01 |
| 38 | rs11592107 | A | 0.30 | 0.03 | 0.02 | 0.01 | -0.03 | 0.01 |
| 39 | rs11604357 | A | -0.28 | 0.04 | 0.00 | 0.01 | 0.00 | 0.01 |
| 40 | rs11636952 | C | -0.53 | 0.03 | -0.03 | 0.01 | 0.01 | 0.01 |
| 41 | rs1169078 | G | 0.20 | 0.03 | 0.01 | 0.01 | 0.00 | 0.01 |
| 42 | rs11694601 | G | 0.19 | 0.03 | -0.01 | 0.01 | -0.01 | 0.01 |
| 43 | rs11834380 | A | -0.28 | 0.05 | -0.01 | 0.02 | 0.05 | 0.01 |
| 44 | rs11874246 | T | 0.29 | 0.03 | 0.02 | 0.01 | -0.01 | 0.01 |
| 45 | rs11925504 | A | -0.29 | 0.03 | -0.04 | 0.01 | 0.03 | 0.01 |
| 46 | rs11960210 | C | -0.47 | 0.03 | -0.03 | 0.01 | 0.02 | 0.01 |
| 47 | rs11977526 | A | -0.32 | 0.03 | 0.02 | 0.01 | 0.01 | 0.01 |
| 48 | rs1199330 | G | 0.27 | 0.05 | 0.06 | 0.02 | -0.03 | 0.01 |
| 49 | rs12042924 | C | 0.18 | 0.03 | -0.01 | 0.01 | -0.02 | 0.01 |
| 50 | rs1209384 | G | -0.26 | 0.03 | -0.01 | 0.01 | -0.01 | 0.01 |
| 51 | rs12136922 | A | 0.20 | 0.03 | -0.01 | 0.01 | -0.01 | 0.01 |
| 52 | rs12255372 | T | 0.24 | 0.03 | 0.02 | 0.01 | -0.26 | 0.01 |
| 53 | rs12258967 | G | -0.63 | 0.03 | -0.03 | 0.01 | -0.02 | 0.01 |
| 54 | rs12264186 | T | 0.21 | 0.04 | 0.01 | 0.01 | -0.01 | 0.01 |
| 55 | rs12321 | C | -0.23 | 0.03 | -0.02 | 0.01 | -0.01 | 0.01 |
| 56 | rs12426261 | G | -0.38 | 0.03 | 0.00 | 0.01 | 0.00 | 0.01 |
| 57 | rs12464602 | A | -0.24 | 0.03 | -0.01 | 0.01 | 0.00 | 0.01 |
| 58 | rs12509595 | C | 0.84 | 0.03 | 0.05 | 0.01 | -0.02 | 0.01 |
| 59 | rs12511987 | G | 0.23 | 0.04 | 0.02 | 0.01 | -0.01 | 0.01 |
| 60 | rs12610654 | G | -0.23 | 0.03 | -0.01 | 0.01 | 0.00 | 0.01 |
| 61 | rs12637573 | G | 0.17 | 0.03 | 0.01 | 0.01 | -0.01 | 0.01 |
| 62 | rs12643599 | G | -0.31 | 0.03 | -0.05 | 0.01 | 0.02 | 0.01 |
| 63 | rs12656497 | C | 0.64 | 0.03 | 0.01 | 0.01 | 0.00 | 0.01 |
| 64 | rs12657950 | T | 0.46 | 0.06 | 0.02 | 0.02 | 0.01 | 0.02 |
| 65 | rs12661036 | C | 0.21 | 0.04 | 0.02 | 0.01 | -0.02 | 0.01 |
| 66 | rs12668436 | C | 0.22 | 0.04 | 0.01 | 0.01 | 0.00 | 0.01 |
| 67 | rs12693982 | T | 0.26 | 0.03 | 0.03 | 0.01 | 0.02 | 0.01 |
| 68 | rs12731646 | T | -0.19 | 0.03 | -0.02 | 0.01 | 0.01 | 0.01 |
| 69 | rs1275985 | T | -0.54 | 0.03 | 0.01 | 0.01 | -0.01 | 0.01 |
| 70 | rs12883810 | T | -0.24 | 0.04 | -0.02 | 0.01 | -0.02 | 0.01 |
| 71 | rs12906962 | C | 0.27 | 0.03 | 0.02 | 0.01 | -0.01 | 0.01 |
| 72 | rs1290784 | T | 0.41 | 0.03 | 0.01 | 0.01 | -0.01 | 0.01 |
| 73 | rs1290933 | A | -0.28 | 0.03 | -0.01 | 0.01 | 0.02 | 0.01 |
| 74 | rs12926550 | A | -0.25 | 0.03 | -0.03 | 0.01 | 0.02 | 0.01 |
| 75 | rs1293969 | C | 0.20 | 0.03 | -0.02 | 0.01 | 0.02 | 0.01 |
| 76 | rs13016772 | T | 0.25 | 0.04 | 0.01 | 0.01 | -0.02 | 0.01 |
| 77 | rs13091418 | G | 0.22 | 0.03 | -0.01 | 0.01 | -0.02 | 0.01 |
| 78 | rs13107261 | A | -0.18 | 0.03 | 0.00 | 0.01 | 0.01 | 0.01 |
| 79 | rs13204703 | C | -0.20 | 0.04 | 0.00 | 0.01 | 0.01 | 0.01 |
| 80 | rs13253358 | T | 0.21 | 0.03 | 0.01 | 0.01 | 0.00 | 0.01 |
| 81 | rs1332813 | C | -0.22 | 0.03 | 0.00 | 0.01 | 0.00 | 0.01 |
| 82 | rs13358657 | G | 0.39 | 0.04 | 0.01 | 0.02 | -0.01 | 0.01 |
| 83 | rs1340030 | C | -0.19 | 0.03 | 0.01 | 0.01 | 0.02 | 0.01 |
| 84 | rs13412750 | A | -0.29 | 0.03 | 0.00 | 0.01 | -0.01 | 0.01 |
| 85 | rs13420463 | G | -0.31 | 0.04 | -0.02 | 0.01 | 0.01 | 0.01 |
| 86 | rs1375564 | T | 0.26 | 0.03 | 0.00 | 0.01 | 0.01 | 0.01 |
| 87 | rs1382472 | A | -0.19 | 0.03 | 0.01 | 0.01 | 0.00 | 0.01 |
| 88 | rs1408945 | T | -0.32 | 0.03 | 0.01 | 0.01 | 0.00 | 0.01 |
| 89 | rs1410222 | T | 0.22 | 0.04 | 0.00 | 0.01 | 0.01 | 0.01 |
| 90 | rs1422279 | T | 0.33 | 0.03 | 0.02 | 0.01 | 0.00 | 0.01 |
| 91 | rs1433121 | T | -0.23 | 0.03 | -0.01 | 0.01 | -0.02 | 0.01 |
| 92 | rs1437649 | A | -0.22 | 0.04 | -0.01 | 0.01 | 0.01 | 0.01 |
| 93 | rs146550789 | C | 0.48 | 0.08 | -0.01 | 0.03 | 0.04 | 0.02 |
| 94 | rs148140538 | T | -0.33 | 0.06 | 0.02 | 0.02 | 0.00 | 0.01 |
| 95 | rs1493132 | C | 0.18 | 0.03 | 0.01 | 0.01 | -0.01 | 0.01 |
| 96 | rs1544861 | C | -0.20 | 0.03 | 0.02 | 0.01 | -0.02 | 0.01 |
| 97 | rs1551355 | T | 0.21 | 0.04 | 0.02 | 0.01 | 0.00 | 0.01 |
| 98 | rs1565440 | A | 0.17 | 0.03 | 0.02 | 0.01 | -0.01 | 0.01 |
| 99 | rs1575290 | T | 0.20 | 0.03 | -0.01 | 0.01 | 0.00 | 0.01 |
| 100 | rs1623474 | T | 0.38 | 0.03 | 0.01 | 0.01 | 0.00 | 0.01 |
| 101 | rs1630736 | T | -0.17 | 0.03 | 0.02 | 0.01 | 0.01 | 0.01 |
| 102 | rs1664781 | A | 0.26 | 0.03 | 0.01 | 0.01 | -0.06 | 0.01 |
| 103 | rs17010957 | C | 0.53 | 0.04 | 0.00 | 0.01 | 0.01 | 0.01 |
| 104 | rs17035181 | G | -0.31 | 0.04 | -0.02 | 0.01 | 0.04 | 0.01 |
| 105 | rs17080102 | C | -0.81 | 0.06 | -0.06 | 0.02 | 0.00 | 0.02 |
| 106 | rs17245822 | C | 0.19 | 0.03 | 0.00 | 0.01 | -0.02 | 0.01 |
| 107 | rs17249754 | A | -0.84 | 0.04 | 0.07 | 0.01 | -0.01 | 0.01 |
| 108 | rs17257081 | G | -0.23 | 0.04 | 0.01 | 0.01 | -0.02 | 0.01 |
| 109 | rs17608766 | C | 0.69 | 0.04 | 0.05 | 0.02 | 0.00 | 0.01 |
| 110 | rs177551 | A | 0.37 | 0.04 | 0.03 | 0.02 | 0.00 | 0.01 |
| 111 | rs17760259 | C | 0.27 | 0.03 | 0.01 | 0.01 | -0.01 | 0.01 |
| 112 | rs17762 | A | 0.41 | 0.06 | 0.02 | 0.02 | -0.02 | 0.01 |
| 113 | rs17807723 | A | -0.27 | 0.04 | -0.02 | 0.02 | -0.01 | 0.01 |
| 114 | rs17812022 | T | -0.36 | 0.05 | 0.03 | 0.02 | 0.00 | 0.01 |
| 115 | rs1786345 | C | -0.21 | 0.03 | 0.00 | 0.01 | 0.01 | 0.01 |
| 116 | rs1814951 | A | -0.32 | 0.05 | -0.02 | 0.01 | 0.00 | 0.01 |
| 117 | rs1821002 | G | -0.38 | 0.03 | -0.01 | 0.01 | 0.04 | 0.01 |
| 118 | rs1848994 | A | 0.20 | 0.03 | -0.02 | 0.01 | 0.02 | 0.01 |
| 119 | rs1870735 | G | -0.21 | 0.03 | 0.01 | 0.01 | 0.00 | 0.01 |
| 120 | rs1871190 | T | 0.20 | 0.03 | 0.02 | 0.01 | -0.01 | 0.01 |
| 121 | rs1882212 | G | -0.28 | 0.04 | 0.01 | 0.01 | 0.01 | 0.01 |
| 122 | rs1882961 | T | 0.24 | 0.03 | 0.02 | 0.01 | 0.01 | 0.01 |
| 123 | rs1889785 | A | 0.18 | 0.03 | -0.01 | 0.01 | -0.01 | 0.01 |
| 124 | rs1906672 | A | 0.30 | 0.04 | -0.01 | 0.01 | -0.01 | 0.01 |
| 125 | rs1957563 | T | 0.36 | 0.03 | 0.01 | 0.01 | -0.01 | 0.01 |
| 126 | rs1984195 | A | 0.24 | 0.03 | 0.03 | 0.01 | -0.01 | 0.01 |
| 127 | rs1994158 | G | -0.25 | 0.04 | 0.00 | 0.01 | -0.01 | 0.01 |
| 128 | rs2014408 | T | 0.52 | 0.04 | 0.02 | 0.01 | -0.02 | 0.01 |
| 129 | rs2024385 | A | -0.26 | 0.03 | 0.01 | 0.01 | -0.01 | 0.01 |
| 130 | rs2111557 | T | 0.18 | 0.03 | 0.00 | 0.01 | -0.01 | 0.01 |
| 131 | rs2126474 | T | -0.26 | 0.03 | 0.00 | 0.01 | 0.01 | 0.01 |
| 132 | rs2129869 | T | 0.26 | 0.04 | 0.00 | 0.01 | -0.05 | 0.01 |
| 133 | rs2161967 | G | -0.28 | 0.03 | -0.04 | 0.01 | 0.00 | 0.01 |
| 134 | rs2177843 | T | 0.44 | 0.04 | 0.01 | 0.01 | 0.03 | 0.01 |
| 135 | rs2236295 | T | -0.30 | 0.03 | 0.01 | 0.01 | 0.03 | 0.01 |
| 136 | rs2249105 | G | -0.29 | 0.03 | 0.00 | 0.01 | 0.05 | 0.01 |
| 137 | rs2276153 | G | -0.33 | 0.04 | 0.01 | 0.01 | 0.04 | 0.01 |
| 138 | rs2283500 | C | -0.31 | 0.05 | -0.02 | 0.02 | 0.01 | 0.01 |
| 139 | rs2291434 | T | -0.26 | 0.03 | 0.00 | 0.01 | 0.03 | 0.01 |
| 140 | rs2353940 | C | 0.21 | 0.04 | 0.01 | 0.01 | -0.01 | 0.01 |
| 141 | rs2354862 | C | -0.25 | 0.03 | 0.02 | 0.01 | 0.01 | 0.01 |
| 142 | rs2384063 | T | 0.33 | 0.04 | -0.02 | 0.01 | 0.00 | 0.01 |
| 143 | rs2392929 | G | 0.75 | 0.04 | 0.00 | 0.01 | 0.01 | 0.01 |
| 144 | rs2423514 | G | -0.30 | 0.03 | -0.03 | 0.01 | 0.01 | 0.01 |
| 145 | rs246973 | T | 0.25 | 0.03 | 0.01 | 0.01 | 0.00 | 0.01 |
| 146 | rs2470004 | T | -0.35 | 0.04 | 0.00 | 0.01 | 0.02 | 0.01 |
| 147 | rs2493134 | C | 0.37 | 0.03 | 0.03 | 0.01 | 0.00 | 0.01 |
| 148 | rs2498323 | A | 0.32 | 0.05 | 0.02 | 0.02 | -0.02 | 0.01 |
| 149 | rs2580350 | A | 0.18 | 0.03 | 0.01 | 0.01 | -0.02 | 0.01 |
| 150 | rs2589218 | C | 0.23 | 0.03 | 0.01 | 0.01 | -0.01 | 0.01 |
| 151 | rs2598 | G | -0.17 | 0.03 | 0.01 | 0.01 | -0.01 | 0.01 |
| 152 | rs2608029 | G | -0.18 | 0.03 | 0.00 | 0.01 | 0.01 | 0.01 |
| 153 | rs2610990 | G | 0.29 | 0.03 | 0.04 | 0.01 | -0.03 | 0.01 |
| 154 | rs2627313 | T | 0.32 | 0.03 | 0.01 | 0.01 | -0.02 | 0.01 |
| 155 | rs262986 | A | -0.24 | 0.03 | -0.01 | 0.01 | 0.01 | 0.01 |
| 156 | rs2643826 | T | 0.45 | 0.03 | 0.00 | 0.01 | -0.02 | 0.01 |
| 157 | rs2652812 | T | -0.25 | 0.04 | -0.03 | 0.01 | 0.02 | 0.01 |
| 158 | rs268263 | A | 0.59 | 0.04 | 0.04 | 0.01 | 0.01 | 0.01 |
| 159 | rs2689690 | T | -0.27 | 0.03 | 0.00 | 0.01 | 0.02 | 0.01 |
| 160 | rs2724377 | G | -0.19 | 0.03 | 0.00 | 0.01 | 0.00 | 0.01 |
| 161 | rs2744139 | C | -0.24 | 0.04 | 0.00 | 0.01 | 0.00 | 0.01 |
| 162 | rs2760748 | A | 0.36 | 0.05 | 0.03 | 0.02 | -0.01 | 0.01 |
| 163 | rs2776037 | C | 0.19 | 0.03 | 0.00 | 0.01 | 0.01 | 0.01 |
| 164 | rs2801008 | G | 0.19 | 0.03 | 0.00 | 0.01 | 0.00 | 0.01 |
| 165 | rs2833834 | A | 0.22 | 0.03 | 0.01 | 0.01 | -0.01 | 0.01 |
| 166 | rs2853736 | G | -0.24 | 0.03 | 0.01 | 0.01 | -0.02 | 0.01 |
| 167 | rs28572357 | C | 0.27 | 0.03 | 0.02 | 0.01 | 0.03 | 0.01 |
| 168 | rs28650790 | T | 0.23 | 0.04 | 0.03 | 0.01 | -0.08 | 0.01 |
| 169 | rs28688791 | C | 0.32 | 0.04 | 0.04 | 0.01 | -0.01 | 0.01 |
| 170 | rs28866311 | G | 0.28 | 0.03 | 0.02 | 0.01 | 0.00 | 0.01 |
| 171 | rs2904315 | G | 0.21 | 0.03 | 0.02 | 0.01 | 0.02 | 0.01 |
| 172 | rs2913920 | T | 0.24 | 0.04 | 0.00 | 0.01 | -0.01 | 0.01 |
| 173 | rs2957688 | A | 0.35 | 0.03 | 0.00 | 0.01 | -0.01 | 0.01 |
| 174 | rs3098186 | T | -0.24 | 0.03 | 0.00 | 0.01 | 0.01 | 0.01 |
| 175 | rs3104552 | C | -0.24 | 0.03 | 0.03 | 0.01 | 0.00 | 0.01 |
| 176 | rs34025993 | G | -0.22 | 0.03 | -0.01 | 0.01 | -0.01 | 0.01 |
| 177 | rs34072724 | A | -0.24 | 0.03 | -0.01 | 0.01 | 0.04 | 0.01 |
| 178 | rs34413141 | A | -0.35 | 0.04 | -0.03 | 0.01 | 0.01 | 0.01 |
| 179 | rs34727427 | C | 0.24 | 0.03 | -0.01 | 0.01 | 0.00 | 0.01 |
| 180 | rs34917849 | C | 0.31 | 0.05 | 0.04 | 0.02 | -0.03 | 0.01 |
| 181 | rs34941092 | A | -0.32 | 0.04 | 0.01 | 0.01 | 0.01 | 0.01 |
| 182 | rs35098810 | C | -0.20 | 0.04 | -0.02 | 0.01 | 0.00 | 0.01 |
| 183 | rs35413927 | G | 0.30 | 0.03 | 0.01 | 0.01 | 0.00 | 0.01 |
| 184 | rs35444 | G | -0.44 | 0.03 | 0.00 | 0.01 | 0.00 | 0.01 |
| 185 | rs35680304 | T | 0.27 | 0.03 | 0.02 | 0.01 | 0.00 | 0.01 |
| 186 | rs35783704 | A | -0.46 | 0.05 | -0.01 | 0.02 | 0.01 | 0.01 |
| 187 | rs3735533 | C | 0.91 | 0.06 | -0.01 | 0.02 | 0.03 | 0.01 |
| 188 | rs3764400 | C | -0.37 | 0.04 | 0.00 | 0.01 | 0.03 | 0.01 |
| 189 | rs3772219 | C | -0.27 | 0.03 | -0.02 | 0.01 | 0.00 | 0.01 |
| 190 | rs3802517 | A | 0.25 | 0.03 | -0.01 | 0.01 | -0.01 | 0.01 |
| 191 | rs3807925 | G | 0.19 | 0.03 | 0.00 | 0.01 | 0.00 | 0.01 |
| 192 | rs3815460 | G | 0.29 | 0.05 | 0.01 | 0.02 | -0.01 | 0.01 |
| 193 | rs3819532 | C | 0.19 | 0.03 | 0.01 | 0.01 | -0.01 | 0.01 |
| 194 | rs3845811 | G | 0.29 | 0.03 | -0.01 | 0.01 | 0.00 | 0.01 |
| 195 | rs3860770 | A | -0.27 | 0.03 | -0.01 | 0.01 | -0.01 | 0.01 |
| 196 | rs3980686 | T | -0.50 | 0.05 | -0.03 | 0.01 | 0.01 | 0.01 |
| 197 | rs404100 | T | 0.19 | 0.03 | 0.00 | 0.01 | 0.01 | 0.01 |
| 198 | rs4143175 | C | -0.22 | 0.04 | -0.01 | 0.01 | 0.00 | 0.01 |
| 199 | rs42032 | A | -0.32 | 0.03 | -0.02 | 0.01 | 0.01 | 0.01 |
| 200 | rs4245599 | G | 0.18 | 0.03 | 0.01 | 0.01 | 0.01 | 0.01 |
| 201 | rs4260863 | G | -0.19 | 0.03 | 0.00 | 0.01 | -0.02 | 0.01 |
| 202 | rs4274337 | G | 0.30 | 0.04 | 0.02 | 0.01 | -0.02 | 0.01 |
| 203 | rs4408839 | G | 0.23 | 0.03 | -0.03 | 0.01 | 0.02 | 0.01 |
| 204 | rs4440615 | A | -0.22 | 0.03 | -0.01 | 0.01 | 0.00 | 0.01 |
| 205 | rs4499560 | T | 0.22 | 0.03 | 0.01 | 0.01 | 0.00 | 0.01 |
| 206 | rs4511593 | T | -0.29 | 0.03 | 0.00 | 0.01 | -0.01 | 0.01 |
| 207 | rs4553000 | T | -0.20 | 0.03 | -0.01 | 0.01 | -0.01 | 0.01 |
| 208 | rs4577304 | C | 0.18 | 0.03 | 0.02 | 0.01 | -0.01 | 0.01 |
| 209 | rs4651224 | T | 0.20 | 0.03 | -0.01 | 0.01 | -0.01 | 0.01 |
| 210 | rs4734868 | G | 0.18 | 0.03 | -0.03 | 0.01 | 0.00 | 0.01 |
| 211 | rs4775769 | G | 0.42 | 0.05 | 0.01 | 0.02 | 0.00 | 0.01 |
| 212 | rs483071 | T | 0.27 | 0.03 | -0.01 | 0.01 | 0.01 | 0.01 |
| 213 | rs4834792 | A | 0.20 | 0.03 | 0.03 | 0.01 | 0.00 | 0.01 |
| 214 | rs4838021 | T | -0.30 | 0.05 | -0.01 | 0.02 | 0.05 | 0.01 |
| 215 | rs4873492 | T | 0.34 | 0.04 | 0.02 | 0.01 | -0.02 | 0.01 |
| 216 | rs4876133 | C | 0.22 | 0.03 | 0.02 | 0.01 | 0.01 | 0.01 |
| 217 | rs4888408 | A | 0.37 | 0.03 | 0.02 | 0.01 | 0.00 | 0.01 |
| 218 | rs4925159 | A | 0.22 | 0.03 | 0.00 | 0.01 | 0.01 | 0.01 |
| 219 | rs4952609 | G | -0.21 | 0.03 | -0.01 | 0.01 | 0.00 | 0.01 |
| 220 | rs4955575 | C | -0.22 | 0.03 | 0.00 | 0.01 | 0.00 | 0.01 |
| 221 | rs4961293 | T | 0.23 | 0.03 | -0.01 | 0.01 | 0.00 | 0.01 |
| 222 | rs509564 | T | 0.26 | 0.04 | 0.02 | 0.01 | 0.01 | 0.01 |
| 223 | rs509833 | G | -0.33 | 0.04 | -0.03 | 0.02 | -0.01 | 0.01 |
| 224 | rs55732192 | T | -0.34 | 0.05 | -0.01 | 0.02 | 0.00 | 0.01 |
| 225 | rs55944332 | G | 0.26 | 0.04 | -0.02 | 0.01 | 0.00 | 0.01 |
| 226 | rs56407827 | T | 0.36 | 0.03 | -0.01 | 0.01 | 0.01 | 0.01 |
| 227 | rs571689 | T | 0.23 | 0.03 | 0.01 | 0.01 | 0.01 | 0.01 |
| 228 | rs573455 | G | -0.20 | 0.03 | -0.02 | 0.01 | -0.01 | 0.01 |
| 229 | rs5742643 | C | 0.22 | 0.03 | 0.00 | 0.01 | -0.01 | 0.01 |
| 230 | rs57786342 | A | 0.23 | 0.04 | 0.02 | 0.01 | -0.02 | 0.01 |
| 231 | rs57866767 | C | -0.45 | 0.03 | 0.02 | 0.01 | 0.01 | 0.01 |
| 232 | rs57946343 | C | -0.72 | 0.04 | -0.02 | 0.02 | 0.00 | 0.01 |
| 233 | rs60138042 | G | -0.34 | 0.06 | -0.01 | 0.02 | 0.03 | 0.02 |
| 234 | rs60191654 | G | 0.24 | 0.04 | 0.01 | 0.01 | 0.01 | 0.01 |
| 235 | rs6026578 | G | 0.19 | 0.03 | -0.01 | 0.01 | 0.02 | 0.01 |
| 236 | rs6026744 | T | 0.71 | 0.05 | 0.05 | 0.02 | -0.01 | 0.01 |
| 237 | rs6029756 | A | -0.27 | 0.03 | -0.01 | 0.01 | 0.00 | 0.01 |
| 238 | rs6031431 | G | 0.26 | 0.03 | 0.00 | 0.01 | 0.00 | 0.01 |
| 239 | rs60444686 | A | 0.59 | 0.08 | 0.05 | 0.03 | 0.01 | 0.02 |
| 240 | rs604723 | C | 0.66 | 0.03 | 0.03 | 0.01 | -0.01 | 0.01 |
| 241 | rs6054139 | A | 0.21 | 0.03 | 0.01 | 0.01 | 0.01 | 0.01 |
| 242 | rs6058088 | G | -0.28 | 0.04 | -0.01 | 0.02 | 0.03 | 0.01 |
| 243 | rs6078093 | A | -0.18 | 0.03 | 0.00 | 0.01 | 0.01 | 0.01 |
| 244 | rs6090907 | A | -0.39 | 0.04 | -0.03 | 0.01 | 0.01 | 0.01 |
| 245 | rs60909079 | C | -0.21 | 0.04 | 0.00 | 0.01 | -0.01 | 0.01 |
| 246 | rs60991988 | G | -0.38 | 0.05 | -0.04 | 0.01 | -0.01 | 0.01 |
| 247 | rs6108787 | G | 0.43 | 0.03 | 0.01 | 0.01 | 0.01 | 0.01 |
| 248 | rs61772592 | G | 0.32 | 0.05 | 0.06 | 0.02 | -0.02 | 0.01 |
| 249 | rs62076622 | G | -0.24 | 0.04 | 0.00 | 0.01 | 0.00 | 0.01 |
| 250 | rs62082230 | A | -0.19 | 0.03 | 0.00 | 0.01 | -0.01 | 0.01 |
| 251 | rs62309747 | A | -0.22 | 0.03 | 0.00 | 0.01 | 0.02 | 0.01 |
| 252 | rs62512914 | G | -0.21 | 0.03 | -0.02 | 0.01 | 0.01 | 0.01 |
| 253 | rs641620 | C | 0.32 | 0.04 | 0.01 | 0.01 | -0.02 | 0.01 |
| 254 | rs6438857 | C | -0.27 | 0.03 | -0.01 | 0.01 | 0.00 | 0.01 |
| 255 | rs6445583 | A | 0.28 | 0.03 | -0.01 | 0.01 | -0.01 | 0.01 |
| 256 | rs6452769 | A | -0.31 | 0.04 | -0.01 | 0.01 | 0.00 | 0.01 |
| 257 | rs6490019 | G | 0.29 | 0.03 | 0.01 | 0.01 | 0.02 | 0.01 |
| 258 | rs6504213 | C | 0.30 | 0.03 | 0.04 | 0.01 | -0.02 | 0.01 |
| 259 | rs6540119 | T | -0.20 | 0.03 | 0.00 | 0.01 | -0.02 | 0.01 |
| 260 | rs6562778 | G | -0.18 | 0.03 | -0.02 | 0.01 | 0.01 | 0.01 |
| 261 | rs658780 | G | 0.20 | 0.03 | 0.00 | 0.01 | 0.00 | 0.01 |
| 262 | rs665445 | A | -0.19 | 0.03 | 0.02 | 0.01 | 0.00 | 0.01 |
| 263 | rs66864335 | A | -0.40 | 0.04 | -0.04 | 0.01 | 0.03 | 0.01 |
| 264 | rs6699618 | G | -0.91 | 0.04 | -0.02 | 0.01 | 0.02 | 0.01 |
| 265 | rs6731373 | A | 0.19 | 0.03 | -0.03 | 0.01 | 0.02 | 0.01 |
| 266 | rs6732123 | C | -0.17 | 0.03 | -0.02 | 0.01 | 0.00 | 0.01 |
| 267 | rs6737318 | G | -0.23 | 0.04 | 0.00 | 0.01 | 0.00 | 0.01 |
| 268 | rs67617547 | G | -0.18 | 0.03 | -0.01 | 0.01 | 0.02 | 0.01 |
| 269 | rs6788907 | A | 0.22 | 0.03 | 0.00 | 0.01 | -0.01 | 0.01 |
| 270 | rs6788984 | G | -0.30 | 0.04 | 0.00 | 0.01 | 0.00 | 0.01 |
| 271 | rs68085857 | T | 0.27 | 0.04 | 0.03 | 0.01 | 0.00 | 0.01 |
| 272 | rs6870654 | C | -0.21 | 0.03 | -0.01 | 0.01 | 0.00 | 0.01 |
| 273 | rs6892983 | A | 0.34 | 0.03 | 0.02 | 0.01 | 0.00 | 0.01 |
| 274 | rs6921291 | T | 0.36 | 0.04 | -0.05 | 0.01 | -0.01 | 0.01 |
| 275 | rs6957161 | G | -0.21 | 0.03 | -0.02 | 0.01 | -0.01 | 0.01 |
| 276 | rs6961048 | G | 0.53 | 0.05 | 0.01 | 0.02 | -0.02 | 0.01 |
| 277 | rs6986368 | T | 0.21 | 0.03 | 0.02 | 0.01 | -0.02 | 0.01 |
| 278 | rs7012866 | G | 0.23 | 0.03 | 0.01 | 0.01 | -0.01 | 0.01 |
| 279 | rs702395 | T | 0.23 | 0.03 | 0.00 | 0.01 | 0.00 | 0.01 |
| 280 | rs7026176 | T | -0.19 | 0.03 | 0.02 | 0.01 | 0.02 | 0.01 |
| 281 | rs7045409 | A | -0.19 | 0.03 | 0.01 | 0.01 | 0.02 | 0.01 |
| 282 | rs708117 | A | 0.29 | 0.03 | 0.00 | 0.01 | 0.01 | 0.01 |
| 283 | rs7093894 | A | 0.24 | 0.04 | -0.01 | 0.01 | -0.03 | 0.01 |
| 284 | rs7107356 | G | 0.46 | 0.03 | 0.01 | 0.01 | 0.02 | 0.01 |
| 285 | rs7134677 | T | -0.39 | 0.03 | -0.01 | 0.01 | 0.02 | 0.01 |
| 286 | rs7154723 | A | 0.25 | 0.03 | 0.01 | 0.01 | -0.02 | 0.01 |
| 287 | rs7186298 | T | -0.23 | 0.03 | -0.02 | 0.01 | 0.00 | 0.01 |
| 288 | rs7198817 | A | -0.18 | 0.03 | -0.02 | 0.01 | 0.00 | 0.01 |
| 289 | rs7211535 | G | 0.18 | 0.03 | -0.01 | 0.01 | 0.01 | 0.01 |
| 290 | rs7213273 | A | -0.40 | 0.03 | -0.01 | 0.01 | 0.01 | 0.01 |
| 291 | rs7236548 | A | 0.34 | 0.04 | 0.00 | 0.01 | -0.01 | 0.01 |
| 292 | rs7255933 | A | 0.23 | 0.03 | 0.03 | 0.01 | -0.03 | 0.01 |
| 293 | rs72719160 | T | 0.22 | 0.03 | 0.02 | 0.01 | -0.03 | 0.01 |
| 294 | rs72742507 | T | -0.21 | 0.03 | -0.01 | 0.01 | -0.02 | 0.01 |
| 295 | rs7278003 | C | 0.19 | 0.03 | 0.04 | 0.01 | -0.02 | 0.01 |
| 296 | rs72847885 | G | -0.24 | 0.03 | -0.02 | 0.01 | 0.02 | 0.01 |
| 297 | rs72931748 | G | -0.40 | 0.05 | -0.01 | 0.02 | 0.00 | 0.01 |
| 298 | rs7306710 | C | 0.24 | 0.03 | 0.02 | 0.01 | -0.05 | 0.01 |
| 299 | rs73075659 | G | -0.40 | 0.03 | -0.01 | 0.01 | -0.01 | 0.01 |
| 300 | rs73103937 | C | -0.21 | 0.03 | -0.02 | 0.01 | -0.02 | 0.01 |
| 301 | rs7310615 | G | -0.59 | 0.03 | -0.07 | 0.01 | 0.03 | 0.01 |
| 302 | rs7331680 | T | 0.41 | 0.04 | 0.00 | 0.01 | 0.01 | 0.01 |
| 303 | rs73855810 | A | 0.27 | 0.04 | 0.06 | 0.01 | -0.02 | 0.01 |
| 304 | rs7395791 | A | -0.22 | 0.03 | 0.00 | 0.01 | 0.02 | 0.01 |
| 305 | rs740746 | A | 0.46 | 0.03 | 0.02 | 0.01 | 0.02 | 0.01 |
| 306 | rs7439567 | T | 0.25 | 0.03 | 0.03 | 0.01 | 0.00 | 0.01 |
| 307 | rs7463212 | A | -0.28 | 0.03 | 0.01 | 0.01 | 0.00 | 0.01 |
| 308 | rs7491248 | A | 0.22 | 0.04 | 0.01 | 0.01 | 0.00 | 0.01 |
| 309 | rs7493678 | T | 0.19 | 0.03 | 0.00 | 0.01 | -0.01 | 0.01 |
| 310 | rs75016974 | T | -0.25 | 0.04 | 0.00 | 0.02 | -0.01 | 0.01 |
| 311 | rs7514579 | C | -0.22 | 0.04 | 0.00 | 0.01 | -0.01 | 0.01 |
| 312 | rs75461554 | T | -0.30 | 0.04 | -0.02 | 0.01 | -0.01 | 0.01 |
| 313 | rs7555285 | C | 0.23 | 0.04 | 0.02 | 0.01 | -0.02 | 0.01 |
| 314 | rs75961402 | A | 0.27 | 0.04 | -0.01 | 0.01 | 0.01 | 0.01 |
| 315 | rs7615099 | G | -0.19 | 0.03 | -0.03 | 0.01 | 0.03 | 0.01 |
| 316 | rs76443575 | C | -0.52 | 0.08 | 0.04 | 0.03 | -0.05 | 0.02 |
| 317 | rs7683728 | T | -0.37 | 0.03 | -0.03 | 0.01 | 0.00 | 0.01 |
| 318 | rs7703560 | G | 0.22 | 0.03 | 0.00 | 0.01 | 0.00 | 0.01 |
| 319 | rs7722243 | A | -0.20 | 0.03 | 0.00 | 0.01 | -0.01 | 0.01 |
| 320 | rs7725413 | T | -0.20 | 0.04 | -0.01 | 0.01 | 0.00 | 0.01 |
| 321 | rs77375686 | G | 0.35 | 0.05 | 0.04 | 0.02 | 0.00 | 0.01 |
| 322 | rs7744902 | A | -0.41 | 0.06 | -0.01 | 0.02 | 0.00 | 0.02 |
| 323 | rs7763558 | A | 0.34 | 0.03 | 0.02 | 0.01 | -0.03 | 0.01 |
| 324 | rs7765526 | G | -0.20 | 0.03 | 0.01 | 0.01 | 0.01 | 0.01 |
| 325 | rs778124 | A | 0.30 | 0.03 | 0.02 | 0.01 | 0.00 | 0.01 |
| 326 | rs7796 | G | -0.34 | 0.03 | 0.00 | 0.01 | 0.00 | 0.01 |
| 327 | rs7821832 | G | -0.42 | 0.03 | -0.01 | 0.01 | 0.00 | 0.01 |
| 328 | rs7844887 | A | 0.27 | 0.04 | -0.01 | 0.01 | 0.01 | 0.01 |
| 329 | rs78474310 | G | 0.47 | 0.07 | 0.03 | 0.03 | 0.01 | 0.02 |
| 330 | rs786923 | T | -0.31 | 0.03 | 0.00 | 0.01 | 0.01 | 0.01 |
| 331 | rs78998485 | G | 0.24 | 0.03 | -0.04 | 0.01 | -0.03 | 0.01 |
| 332 | rs7912283 | A | -0.21 | 0.03 | -0.01 | 0.01 | 0.00 | 0.01 |
| 333 | rs7926110 | G | -0.26 | 0.03 | -0.02 | 0.01 | 0.00 | 0.01 |
| 334 | rs79384779 | T | 0.32 | 0.04 | 0.00 | 0.01 | -0.02 | 0.01 |
| 335 | rs79539362 | C | -0.40 | 0.05 | 0.04 | 0.02 | 0.00 | 0.01 |
| 336 | rs7963801 | C | 0.24 | 0.03 | 0.03 | 0.01 | 0.00 | 0.01 |
| 337 | rs79782817 | T | 0.53 | 0.05 | 0.03 | 0.02 | -0.01 | 0.01 |
| 338 | rs7980644 | G | -0.26 | 0.04 | 0.02 | 0.01 | 0.02 | 0.01 |
| 339 | rs8003103 | A | -0.18 | 0.03 | 0.01 | 0.01 | 0.01 | 0.01 |
| 340 | rs8030856 | G | 0.18 | 0.03 | 0.01 | 0.01 | -0.01 | 0.01 |
| 341 | rs8044992 | C | -0.21 | 0.03 | -0.01 | 0.01 | 0.01 | 0.01 |
| 342 | rs8054587 | C | -0.17 | 0.03 | -0.01 | 0.01 | 0.00 | 0.01 |
| 343 | rs8125763 | A | 0.18 | 0.03 | -0.01 | 0.01 | -0.01 | 0.01 |
| 344 | rs8142376 | T | 0.17 | 0.03 | 0.02 | 0.01 | 0.01 | 0.01 |
| 345 | rs8180684 | T | 0.21 | 0.03 | 0.02 | 0.01 | -0.01 | 0.01 |
| 346 | rs848445 | C | 0.20 | 0.03 | 0.00 | 0.01 | -0.03 | 0.01 |
| 347 | rs869396 | A | -0.21 | 0.03 | -0.03 | 0.01 | 0.00 | 0.01 |
| 348 | rs871004 | A | 0.23 | 0.03 | 0.01 | 0.01 | -0.03 | 0.01 |
| 349 | rs8904 | A | 0.31 | 0.03 | 0.00 | 0.01 | 0.00 | 0.01 |
| 350 | rs927315 | T | 0.17 | 0.03 | 0.00 | 0.01 | 0.00 | 0.01 |
| 351 | rs9285476 | G | -0.18 | 0.03 | -0.06 | 0.01 | 0.01 | 0.01 |
| 352 | rs9302885 | G | -0.22 | 0.03 | -0.01 | 0.01 | 0.02 | 0.01 |
| 353 | rs9327297 | G | -0.27 | 0.03 | -0.04 | 0.01 | 0.02 | 0.01 |
| 354 | rs9349379 | G | -0.27 | 0.03 | 0.13 | 0.01 | 0.01 | 0.01 |
| 355 | rs9361836 | T | 0.22 | 0.03 | 0.01 | 0.01 | 0.01 | 0.01 |
| 356 | rs9368222 | A | 0.23 | 0.03 | 0.01 | 0.01 | -0.13 | 0.01 |
| 357 | rs9401913 | A | 0.52 | 0.03 | 0.01 | 0.01 | 0.02 | 0.01 |
| 358 | rs9486916 | T | 0.27 | 0.04 | -0.01 | 0.01 | 0.02 | 0.01 |
| 359 | rs9526707 | A | -0.20 | 0.03 | -0.01 | 0.01 | -0.01 | 0.01 |
| 360 | rs961764 | G | 0.19 | 0.03 | 0.00 | 0.01 | -0.02 | 0.01 |
| 361 | rs9848170 | C | 0.32 | 0.03 | 0.01 | 0.01 | -0.01 | 0.01 |
| 362 | rs9857362 | C | -0.17 | 0.03 | 0.00 | 0.01 | -0.01 | 0.01 |
| 363 | rs9869437 | A | -0.20 | 0.03 | 0.01 | 0.01 | -0.02 | 0.01 |
| 364 | rs9876694 | T | 0.47 | 0.07 | -0.01 | 0.02 | 0.05 | 0.02 |
| 365 | rs9880098 | A | 0.31 | 0.03 | 0.01 | 0.01 | 0.00 | 0.01 |
| 366 | rs9886665 | C | -0.20 | 0.03 | -0.02 | 0.01 | 0.01 | 0.01 |
| 367 | rs9899540 | T | -0.20 | 0.03 | 0.00 | 0.01 | -0.01 | 0.01 |
| 368 | rs9918876 | A | -0.30 | 0.05 | 0.00 | 0.02 | 0.00 | 0.01 |

SBP: Systolic blood pressure; MI: Myocardial infarction; T2DM: Type-2 diabetes

Table 38: Genetic association estimates for the effect of DBP on MI, adjusted for T2DM. ea=effect allele, gx=DBP, gy=MI, gz=T2DM, se=standard error

|  | SNP | ea | gx | gx_se | gy | gy_se | gz | gz_se |
| --- | --- | --- | --- | --- | --- | --- | --- | --- |
| 1 | rs10048404 | T | -0.11 | 0.02 | -0.01 | 0.01 | 0.02 | 0.01 |
| 2 | rs10062049 | T | 0.22 | 0.03 | 0.01 | 0.01 | 0.02 | 0.01 |
| 3 | rs1006545 | T | 0.36 | 0.03 | -0.02 | 0.02 | 0.03 | 0.01 |
| 4 | rs10087280 | G | -0.14 | 0.02 | 0.00 | 0.01 | 0.02 | 0.01 |
| 5 | rs10164193 | G | 0.22 | 0.03 | 0.00 | 0.02 | 0.01 | 0.01 |
| 6 | rs10279432 | A | 0.12 | 0.02 | 0.02 | 0.01 | -0.02 | 0.01 |
| 7 | rs1035673 | C | -0.16 | 0.02 | -0.04 | 0.01 | 0.01 | 0.01 |
| 8 | rs1044822 | T | -0.13 | 0.02 | -0.01 | 0.02 | 0.02 | 0.01 |
| 9 | rs10490923 | A | 0.15 | 0.03 | -0.01 | 0.02 | -0.02 | 0.01 |
| 10 | rs10491713 | T | -0.12 | 0.02 | 0.00 | 0.01 | 0.00 | 0.01 |
| 11 | rs1049212 | G | 0.18 | 0.02 | 0.01 | 0.01 | 0.01 | 0.01 |
| 12 | rs10493408 | A | 0.16 | 0.03 | -0.01 | 0.01 | -0.03 | 0.01 |
| 13 | rs10500932 | A | 0.28 | 0.03 | 0.03 | 0.02 | -0.02 | 0.01 |
| 14 | rs10759697 | A | 0.13 | 0.02 | 0.02 | 0.01 | 0.02 | 0.01 |
| 15 | rs10804330 | C | -0.13 | 0.02 | -0.01 | 0.01 | 0.07 | 0.01 |
| 16 | rs10832586 | C | 0.31 | 0.02 | 0.02 | 0.01 | -0.02 | 0.01 |
| 17 | rs10838702 | T | 0.24 | 0.02 | 0.01 | 0.01 | 0.01 | 0.01 |
| 18 | rs10873612 | T | -0.11 | 0.02 | -0.02 | 0.01 | 0.00 | 0.01 |
| 19 | rs10941043 | G | 0.13 | 0.02 | 0.00 | 0.01 | -0.01 | 0.01 |
| 20 | rs10980408 | C | 0.37 | 0.05 | 0.06 | 0.03 | -0.03 | 0.02 |
| 21 | rs11021221 | A | -0.19 | 0.02 | -0.02 | 0.01 | 0.00 | 0.01 |
| 22 | rs11070245 | G | 0.13 | 0.02 | -0.01 | 0.01 | -0.01 | 0.01 |
| 23 | rs11108209 | C | 0.19 | 0.03 | -0.04 | 0.02 | -0.01 | 0.01 |
| 24 | rs11112548 | T | -0.27 | 0.04 | 0.05 | 0.03 | 0.02 | 0.02 |
| 25 | rs11130602 | A | 0.15 | 0.02 | 0.02 | 0.01 | 0.02 | 0.01 |
| 26 | rs11141731 | T | -0.13 | 0.02 | 0.00 | 0.01 | 0.00 | 0.01 |
| 27 | rs1114347 | G | 0.18 | 0.02 | 0.01 | 0.01 | 0.01 | 0.01 |
| 28 | rs11153590 | A | -0.11 | 0.02 | 0.02 | 0.01 | 0.00 | 0.01 |
| 29 | rs11153730 | C | -0.16 | 0.02 | 0.03 | 0.01 | -0.01 | 0.01 |
| 30 | rs11187838 | A | -0.28 | 0.02 | 0.02 | 0.01 | 0.01 | 0.01 |
| 31 | rs11191580 | C | -0.51 | 0.03 | -0.07 | 0.02 | 0.00 | 0.01 |
| 32 | rs11231693 | A | 0.22 | 0.04 | 0.02 | 0.02 | -0.06 | 0.02 |
| 33 | rs112393817 | G | -0.12 | 0.02 | 0.01 | 0.01 | -0.01 | 0.01 |
| 34 | rs11245631 | T | -0.15 | 0.02 | 0.01 | 0.01 | 0.02 | 0.01 |
| 35 | rs11252324 | T | -0.23 | 0.03 | -0.07 | 0.02 | -0.01 | 0.01 |
| 36 | rs114714860 | C | 0.33 | 0.02 | 0.01 | 0.01 | -0.01 | 0.01 |
| 37 | rs11556924 | T | -0.18 | 0.02 | -0.07 | 0.01 | 0.01 | 0.01 |
| 38 | rs11578696 | G | -0.15 | 0.03 | -0.01 | 0.02 | -0.01 | 0.01 |
| 39 | rs11592107 | A | 0.12 | 0.02 | 0.02 | 0.01 | -0.03 | 0.01 |
| 40 | rs11636952 | C | -0.40 | 0.02 | -0.03 | 0.01 | 0.01 | 0.01 |
| 41 | rs11661473 | A | 0.20 | 0.02 | -0.01 | 0.01 | 0.01 | 0.01 |
| 42 | rs11664194 | A | -0.11 | 0.02 | 0.03 | 0.01 | -0.01 | 0.01 |
| 43 | rs11665020 | C | -0.14 | 0.02 | -0.01 | 0.01 | 0.00 | 0.01 |
| 44 | rs11721984 | T | -0.14 | 0.02 | -0.02 | 0.01 | 0.01 | 0.01 |
| 45 | rs11745207 | G | -0.11 | 0.02 | -0.01 | 0.01 | 0.01 | 0.01 |
| 46 | rs11778153 | C | -0.12 | 0.02 | 0.01 | 0.01 | 0.00 | 0.01 |
| 47 | rs1178979 | C | -0.15 | 0.02 | -0.01 | 0.01 | -0.02 | 0.01 |
| 48 | rs11923343 | G | 0.11 | 0.02 | 0.00 | 0.01 | 0.01 | 0.01 |
| 49 | rs11923667 | A | 0.12 | 0.02 | 0.00 | 0.01 | 0.01 | 0.01 |
| 50 | rs11945489 | T | -0.14 | 0.02 | -0.02 | 0.01 | 0.02 | 0.01 |
| 51 | rs11960210 | C | -0.25 | 0.02 | -0.03 | 0.01 | 0.02 | 0.01 |
| 52 | rs11961593 | T | -0.32 | 0.03 | -0.01 | 0.02 | 0.00 | 0.01 |
| 53 | rs12088448 | C | 0.15 | 0.02 | 0.00 | 0.01 | 0.00 | 0.01 |
| 54 | rs1212061 | C | 0.13 | 0.02 | -0.01 | 0.01 | 0.00 | 0.01 |
| 55 | rs12149254 | A | -0.13 | 0.02 | 0.00 | 0.02 | -0.01 | 0.01 |
| 56 | rs12152463 | T | 0.10 | 0.02 | 0.01 | 0.01 | -0.01 | 0.01 |
| 57 | rs12258967 | G | -0.35 | 0.02 | -0.03 | 0.01 | -0.02 | 0.01 |
| 58 | rs12321 | C | -0.15 | 0.02 | -0.02 | 0.01 | -0.01 | 0.01 |
| 59 | rs12337056 | T | 0.14 | 0.02 | 0.02 | 0.02 | 0.02 | 0.01 |
| 60 | rs12363520 | A | 0.17 | 0.02 | -0.02 | 0.01 | 0.01 | 0.01 |
| 61 | rs12405515 | T | -0.17 | 0.02 | -0.02 | 0.01 | 0.04 | 0.01 |
| 62 | rs1243876 | T | -0.11 | 0.02 | -0.03 | 0.01 | -0.03 | 0.01 |
| 63 | rs12503341 | A | -0.30 | 0.05 | 0.02 | 0.02 | 0.02 | 0.02 |
| 64 | rs12509595 | C | 0.50 | 0.02 | 0.05 | 0.01 | -0.02 | 0.01 |
| 65 | rs12515541 | T | 0.12 | 0.02 | 0.02 | 0.01 | -0.02 | 0.01 |
| 66 | rs12574332 | T | 0.21 | 0.03 | 0.02 | 0.02 | -0.01 | 0.01 |
| 67 | rs12609484 | T | -0.14 | 0.02 | -0.02 | 0.01 | 0.01 | 0.01 |
| 68 | rs1263671 | C | 0.14 | 0.02 | 0.02 | 0.01 | -0.01 | 0.01 |
| 69 | rs12656497 | C | 0.31 | 0.02 | 0.01 | 0.01 | 0.00 | 0.01 |
| 70 | rs1265842 | C | -0.11 | 0.02 | 0.01 | 0.01 | -0.01 | 0.01 |
| 71 | rs12693302 | A | -0.24 | 0.02 | -0.04 | 0.01 | -0.02 | 0.01 |
| 72 | rs12728150 | G | 0.20 | 0.03 | 0.02 | 0.02 | -0.01 | 0.01 |
| 73 | rs1275985 | T | -0.29 | 0.02 | 0.01 | 0.01 | -0.01 | 0.01 |
| 74 | rs12790943 | T | -0.10 | 0.02 | -0.04 | 0.01 | 0.00 | 0.01 |
| 75 | rs12906962 | C | 0.24 | 0.02 | 0.02 | 0.01 | -0.01 | 0.01 |
| 76 | rs12919839 | T | -0.11 | 0.02 | -0.01 | 0.01 | 0.00 | 0.01 |
| 77 | rs12929303 | A | 0.16 | 0.02 | 0.01 | 0.01 | -0.02 | 0.01 |
| 78 | rs12938803 | C | 0.16 | 0.02 | -0.03 | 0.01 | -0.01 | 0.01 |
| 79 | rs12990959 | C | 0.13 | 0.02 | 0.03 | 0.01 | -0.01 | 0.01 |
| 80 | rs13004222 | G | -0.29 | 0.04 | 0.00 | 0.02 | -0.02 | 0.02 |
| 81 | rs13118687 | A | -0.15 | 0.02 | 0.00 | 0.01 | 0.00 | 0.01 |
| 82 | rs13124515 | C | 0.11 | 0.02 | -0.01 | 0.01 | -0.01 | 0.01 |
| 83 | rs13139571 | A | -0.24 | 0.02 | -0.05 | 0.01 | 0.02 | 0.01 |
| 84 | rs13152154 | T | -0.12 | 0.02 | -0.02 | 0.01 | -0.01 | 0.01 |
| 85 | rs13215166 | G | 0.31 | 0.02 | 0.00 | 0.01 | 0.02 | 0.01 |
| 86 | rs1322639 | A | -0.16 | 0.02 | -0.01 | 0.01 | -0.01 | 0.01 |
| 87 | rs13237249 | T | 0.14 | 0.02 | 0.01 | 0.01 | -0.01 | 0.01 |
| 88 | rs1327235 | G | 0.30 | 0.02 | 0.01 | 0.01 | 0.00 | 0.01 |
| 89 | rs1332812 | A | -0.11 | 0.02 | 0.00 | 0.01 | 0.00 | 0.01 |
| 90 | rs13355146 | T | 0.12 | 0.02 | 0.00 | 0.01 | 0.00 | 0.01 |
| 91 | rs13358657 | G | 0.22 | 0.03 | 0.01 | 0.02 | -0.01 | 0.01 |
| 92 | rs135023 | G | 0.10 | 0.02 | 0.01 | 0.01 | -0.01 | 0.01 |
| 93 | rs1373780 | C | 0.12 | 0.02 | 0.00 | 0.01 | 0.01 | 0.01 |
| 94 | rs1390754 | T | 0.13 | 0.02 | 0.02 | 0.01 | 0.03 | 0.01 |
| 95 | rs142449193 | T | -0.26 | 0.04 | -0.03 | 0.03 | -0.03 | 0.02 |
| 96 | rs1425486 | T | -0.13 | 0.02 | -0.01 | 0.01 | 0.04 | 0.01 |
| 97 | rs1433121 | T | -0.14 | 0.02 | -0.01 | 0.01 | -0.02 | 0.01 |
| 98 | rs1446468 | C | 0.25 | 0.02 | 0.01 | 0.01 | 0.01 | 0.01 |
| 99 | rs1449596 | G | 0.11 | 0.02 | -0.01 | 0.01 | 0.00 | 0.01 |
| 100 | rs1467049 | G | -0.12 | 0.02 | -0.01 | 0.01 | -0.01 | 0.01 |
| 101 | rs15009 | G | 0.12 | 0.02 | 0.00 | 0.01 | 0.02 | 0.01 |
| 102 | rs1502358 | A | -0.11 | 0.02 | 0.00 | 0.01 | 0.00 | 0.01 |
| 103 | rs1523871 | G | 0.12 | 0.02 | -0.01 | 0.01 | 0.01 | 0.01 |
| 104 | rs1527797 | T | -0.14 | 0.02 | 0.03 | 0.01 | -0.02 | 0.01 |
| 105 | rs1528293 | T | -0.28 | 0.02 | -0.02 | 0.01 | 0.01 | 0.01 |
| 106 | rs1534338 | A | -0.11 | 0.02 | -0.01 | 0.01 | 0.00 | 0.01 |
| 107 | rs1582931 | A | 0.22 | 0.02 | -0.02 | 0.01 | 0.01 | 0.01 |
| 108 | rs1623474 | T | 0.22 | 0.02 | 0.01 | 0.01 | 0.00 | 0.01 |
| 109 | rs1669907 | G | -0.12 | 0.02 | -0.02 | 0.01 | 0.00 | 0.01 |
| 110 | rs16853198 | G | -0.34 | 0.03 | -0.04 | 0.02 | 0.01 | 0.01 |
| 111 | rs1687295 | C | -0.21 | 0.02 | -0.01 | 0.01 | 0.03 | 0.01 |
| 112 | rs16875357 | G | 0.12 | 0.02 | 0.00 | 0.01 | 0.00 | 0.01 |
| 113 | rs16896276 | A | -0.13 | 0.02 | -0.04 | 0.01 | 0.03 | 0.01 |
| 114 | rs1693560 | G | -0.15 | 0.02 | 0.00 | 0.01 | 0.00 | 0.01 |
| 115 | rs17321041 | T | 0.23 | 0.04 | -0.01 | 0.02 | 0.01 | 0.02 |
| 116 | rs173396 | A | 0.21 | 0.02 | 0.04 | 0.01 | 0.00 | 0.01 |
| 117 | rs17396055 | A | -0.12 | 0.02 | -0.01 | 0.01 | 0.00 | 0.01 |
| 118 | rs17432462 | C | 0.10 | 0.02 | 0.00 | 0.01 | 0.00 | 0.01 |
| 119 | rs17454517 | G | -0.12 | 0.02 | -0.01 | 0.01 | 0.00 | 0.01 |
| 120 | rs17677603 | G | 0.20 | 0.02 | 0.02 | 0.01 | 0.00 | 0.01 |
| 121 | rs17696749 | G | 0.13 | 0.02 | 0.02 | 0.01 | 0.00 | 0.01 |
| 122 | rs17807723 | A | -0.18 | 0.03 | -0.02 | 0.02 | -0.01 | 0.01 |
| 123 | rs1790123 | T | 0.20 | 0.02 | 0.02 | 0.01 | -0.04 | 0.01 |
| 124 | rs1799945 | G | 0.39 | 0.02 | 0.00 | 0.02 | -0.02 | 0.01 |
| 125 | rs1819663 | G | -0.11 | 0.02 | 0.02 | 0.01 | 0.00 | 0.01 |
| 126 | rs1867624 | T | 0.14 | 0.02 | 0.04 | 0.01 | -0.02 | 0.01 |
| 127 | rs1871190 | T | 0.11 | 0.02 | 0.02 | 0.01 | -0.01 | 0.01 |
| 128 | rs1876490 | A | 0.14 | 0.02 | 0.02 | 0.01 | 0.00 | 0.01 |
| 129 | rs1882961 | T | 0.13 | 0.02 | 0.02 | 0.01 | 0.01 | 0.01 |
| 130 | rs1889785 | A | 0.13 | 0.02 | -0.01 | 0.01 | -0.01 | 0.01 |
| 131 | rs1903752 | T | -0.10 | 0.02 | -0.01 | 0.01 | 0.01 | 0.01 |
| 132 | rs1906672 | A | 0.14 | 0.02 | -0.01 | 0.01 | -0.01 | 0.01 |
| 133 | rs194742 | C | -0.13 | 0.02 | -0.03 | 0.01 | 0.02 | 0.01 |
| 134 | rs1948151 | A | -0.14 | 0.02 | 0.00 | 0.01 | 0.02 | 0.01 |
| 135 | rs1950500 | C | -0.14 | 0.02 | 0.00 | 0.01 | 0.00 | 0.01 |
| 136 | rs1984195 | A | 0.17 | 0.02 | 0.03 | 0.01 | -0.01 | 0.01 |
| 137 | rs1999996 | G | 0.11 | 0.02 | 0.00 | 0.01 | -0.01 | 0.01 |
| 138 | rs2067831 | C | -0.13 | 0.02 | 0.04 | 0.01 | 0.01 | 0.01 |
| 139 | rs2070527 | C | 0.15 | 0.02 | 0.04 | 0.01 | -0.02 | 0.01 |
| 140 | rs2133386 | A | -0.13 | 0.02 | 0.03 | 0.01 | 0.00 | 0.01 |
| 141 | rs2146315 | T | -0.12 | 0.02 | 0.00 | 0.01 | 0.01 | 0.01 |
| 142 | rs2160236 | C | -0.14 | 0.02 | 0.00 | 0.01 | -0.01 | 0.01 |
| 143 | rs2169137 | C | 0.16 | 0.02 | 0.00 | 0.01 | 0.02 | 0.01 |
| 144 | rs2191046 | G | -0.12 | 0.02 | -0.02 | 0.01 | 0.01 | 0.01 |
| 145 | rs2236295 | T | -0.21 | 0.02 | 0.01 | 0.01 | 0.03 | 0.01 |
| 146 | rs2239268 | A | 0.11 | 0.02 | 0.00 | 0.01 | 0.00 | 0.01 |
| 147 | rs2239917 | C | -0.17 | 0.02 | 0.00 | 0.01 | 0.01 | 0.01 |
| 148 | rs2256187 | A | -0.14 | 0.02 | -0.04 | 0.01 | 0.02 | 0.01 |
| 149 | rs2273654 | C | -0.12 | 0.02 | 0.01 | 0.01 | -0.01 | 0.01 |
| 150 | rs227426 | T | 0.11 | 0.02 | 0.01 | 0.01 | 0.00 | 0.01 |
| 151 | rs2305654 | A | 0.17 | 0.02 | -0.01 | 0.01 | -0.03 | 0.01 |
| 152 | rs2307111 | C | 0.17 | 0.02 | -0.01 | 0.01 | 0.04 | 0.01 |
| 153 | rs234616 | A | -0.12 | 0.02 | 0.01 | 0.01 | 0.00 | 0.01 |
| 154 | rs2384061 | A | -0.17 | 0.02 | 0.02 | 0.01 | -0.01 | 0.01 |
| 155 | rs2397060 | C | 0.16 | 0.03 | 0.00 | 0.01 | -0.04 | 0.01 |
| 156 | rs2421200 | T | -0.11 | 0.02 | -0.01 | 0.01 | 0.00 | 0.01 |
| 157 | rs2444769 | A | 0.16 | 0.02 | -0.02 | 0.01 | 0.00 | 0.01 |
| 158 | rs2484294 | A | 0.32 | 0.02 | 0.01 | 0.01 | 0.02 | 0.01 |
| 159 | rs2487926 | G | -0.10 | 0.02 | 0.05 | 0.01 | 0.00 | 0.01 |
| 160 | rs2493136 | T | 0.23 | 0.02 | 0.03 | 0.01 | -0.01 | 0.01 |
| 161 | rs2515424 | T | 0.13 | 0.02 | 0.03 | 0.01 | -0.01 | 0.01 |
| 162 | rs2548459 | C | 0.13 | 0.02 | 0.01 | 0.01 | 0.01 | 0.01 |
| 163 | rs2586970 | G | 0.15 | 0.02 | 0.00 | 0.01 | -0.02 | 0.01 |
| 164 | rs2589218 | C | 0.12 | 0.02 | 0.01 | 0.01 | -0.01 | 0.01 |
| 165 | rs2598 | G | -0.14 | 0.02 | 0.01 | 0.01 | -0.01 | 0.01 |
| 166 | rs2627313 | T | 0.15 | 0.02 | 0.01 | 0.01 | -0.02 | 0.01 |
| 167 | rs2643826 | T | 0.19 | 0.02 | 0.00 | 0.01 | -0.02 | 0.01 |
| 168 | rs2681485 | A | 0.29 | 0.02 | -0.04 | 0.01 | 0.00 | 0.01 |
| 169 | rs2744133 | G | -0.14 | 0.02 | 0.01 | 0.01 | 0.01 | 0.01 |
| 170 | rs28377357 | A | -0.12 | 0.02 | -0.01 | 0.01 | 0.02 | 0.01 |
| 171 | rs28544928 | G | -0.15 | 0.02 | 0.00 | 0.01 | 0.02 | 0.01 |
| 172 | rs2854746 | C | 0.11 | 0.02 | 0.02 | 0.01 | 0.02 | 0.01 |
| 173 | rs28675079 | A | -0.14 | 0.02 | 0.00 | 0.01 | -0.01 | 0.01 |
| 174 | rs2921604 | C | 0.10 | 0.02 | -0.01 | 0.01 | 0.01 | 0.01 |
| 175 | rs2925345 | C | -0.19 | 0.02 | -0.02 | 0.01 | 0.00 | 0.01 |
| 176 | rs2957468 | G | -0.14 | 0.02 | 0.01 | 0.01 | -0.01 | 0.01 |
| 177 | rs3006583 | C | 0.13 | 0.02 | -0.02 | 0.01 | -0.02 | 0.01 |
| 178 | rs310597 | G | 0.12 | 0.02 | 0.00 | 0.01 | 0.02 | 0.01 |
| 179 | rs311564 | A | -0.13 | 0.02 | -0.02 | 0.01 | 0.00 | 0.01 |
| 180 | rs3117736 | T | 0.24 | 0.02 | 0.02 | 0.01 | -0.01 | 0.01 |
| 181 | rs318712 | C | 0.24 | 0.03 | 0.03 | 0.02 | 0.01 | 0.02 |
| 182 | rs335170 | C | -0.11 | 0.02 | -0.02 | 0.01 | 0.01 | 0.01 |
| 183 | rs342977 | A | -0.16 | 0.02 | -0.02 | 0.01 | 0.00 | 0.01 |
| 184 | rs34413141 | A | -0.18 | 0.02 | -0.03 | 0.01 | 0.01 | 0.01 |
| 185 | rs34587839 | A | -0.17 | 0.02 | 0.01 | 0.02 | -0.04 | 0.01 |
| 186 | rs34645159 | A | -0.13 | 0.02 | 0.00 | 0.01 | 0.00 | 0.01 |
| 187 | rs347585 | T | 0.15 | 0.02 | 0.00 | 0.01 | -0.01 | 0.01 |
| 188 | rs35091929 | C | -0.18 | 0.02 | -0.01 | 0.01 | 0.04 | 0.01 |
| 189 | rs35261542 | A | 0.12 | 0.02 | 0.02 | 0.01 | -0.12 | 0.01 |
| 190 | rs35413927 | G | 0.13 | 0.02 | 0.01 | 0.01 | 0.00 | 0.01 |
| 191 | rs35443 | C | -0.27 | 0.02 | 0.00 | 0.01 | 0.00 | 0.01 |
| 192 | rs35506078 | C | 0.13 | 0.02 | -0.01 | 0.01 | -0.02 | 0.01 |
| 193 | rs3735533 | C | 0.49 | 0.03 | -0.01 | 0.02 | 0.03 | 0.01 |
| 194 | rs3743111 | A | 0.15 | 0.02 | -0.02 | 0.01 | -0.01 | 0.01 |
| 195 | rs3743369 | A | 0.10 | 0.02 | 0.01 | 0.01 | -0.01 | 0.01 |
| 196 | rs3761077 | T | 0.17 | 0.03 | 0.01 | 0.02 | -0.06 | 0.01 |
| 197 | rs3772219 | C | -0.18 | 0.02 | -0.02 | 0.01 | 0.00 | 0.01 |
| 198 | rs3774702 | A | 0.15 | 0.02 | -0.02 | 0.01 | -0.01 | 0.01 |
| 199 | rs3776299 | A | 0.13 | 0.02 | 0.03 | 0.01 | -0.02 | 0.01 |
| 200 | rs3785837 | A | 0.15 | 0.02 | 0.02 | 0.01 | -0.01 | 0.01 |
| 201 | rs3798293 | G | 0.13 | 0.02 | -0.05 | 0.01 | -0.01 | 0.01 |
| 202 | rs3802230 | A | -0.16 | 0.02 | 0.01 | 0.01 | 0.00 | 0.01 |
| 203 | rs3802517 | A | 0.13 | 0.02 | -0.01 | 0.01 | -0.01 | 0.01 |
| 204 | rs3807101 | T | -0.17 | 0.03 | 0.01 | 0.02 | -0.01 | 0.01 |
| 205 | rs3861113 | A | 0.21 | 0.03 | 0.00 | 0.01 | 0.00 | 0.01 |
| 206 | rs3864004 | A | 0.10 | 0.02 | 0.01 | 0.01 | -0.01 | 0.01 |
| 207 | rs3943093 | T | 0.25 | 0.02 | 0.01 | 0.01 | 0.00 | 0.01 |
| 208 | rs4074812 | A | -0.13 | 0.02 | 0.01 | 0.01 | -0.01 | 0.01 |
| 209 | rs4077158 | C | 0.18 | 0.02 | 0.01 | 0.01 | 0.01 | 0.01 |
| 210 | rs4141663 | T | -0.15 | 0.02 | -0.01 | 0.01 | 0.00 | 0.01 |
| 211 | rs4244200 | C | -0.12 | 0.02 | 0.01 | 0.01 | -0.02 | 0.01 |
| 212 | rs4245930 | A | -0.12 | 0.02 | 0.00 | 0.01 | -0.01 | 0.01 |
| 213 | rs4306343 | T | 0.32 | 0.02 | 0.03 | 0.01 | 0.00 | 0.01 |
| 214 | rs4362428 | A | -0.11 | 0.02 | 0.00 | 0.01 | 0.00 | 0.01 |
| 215 | rs440454 | G | 0.26 | 0.02 | -0.03 | 0.01 | -0.03 | 0.01 |
| 216 | rs4424827 | T | -0.10 | 0.02 | 0.01 | 0.01 | -0.01 | 0.01 |
| 217 | rs4507125 | C | 0.12 | 0.02 | 0.00 | 0.01 | 0.02 | 0.01 |
| 218 | rs45474499 | T | 0.36 | 0.04 | 0.00 | 0.03 | 0.04 | 0.02 |
| 219 | rs4556017 | T | -0.16 | 0.02 | -0.01 | 0.02 | -0.02 | 0.01 |
| 220 | rs4615669 | G | 0.11 | 0.02 | -0.04 | 0.01 | 0.02 | 0.01 |
| 221 | rs4645335 | G | -0.11 | 0.02 | 0.00 | 0.01 | 0.00 | 0.01 |
| 222 | rs4675682 | C | 0.14 | 0.02 | 0.00 | 0.01 | 0.00 | 0.01 |
| 223 | rs4704514 | T | 0.11 | 0.02 | 0.01 | 0.01 | 0.00 | 0.01 |
| 224 | rs4739832 | C | -0.13 | 0.02 | -0.02 | 0.01 | 0.01 | 0.01 |
| 225 | rs4756779 | G | 0.15 | 0.02 | 0.01 | 0.01 | -0.02 | 0.01 |
| 226 | rs4814837 | T | -0.10 | 0.02 | -0.02 | 0.01 | 0.01 | 0.01 |
| 227 | rs4873492 | T | 0.14 | 0.02 | 0.02 | 0.01 | -0.02 | 0.01 |
| 228 | rs4890499 | A | 0.11 | 0.02 | 0.00 | 0.01 | 0.00 | 0.01 |
| 229 | rs4891258 | G | 0.12 | 0.02 | 0.00 | 0.01 | -0.01 | 0.01 |
| 230 | rs4903064 | C | -0.15 | 0.02 | 0.02 | 0.01 | -0.02 | 0.01 |
| 231 | rs4909314 | A | 0.13 | 0.02 | 0.01 | 0.01 | -0.01 | 0.01 |
| 232 | rs4926901 | A | 0.10 | 0.02 | 0.00 | 0.01 | 0.01 | 0.01 |
| 233 | rs4926923 | C | -0.19 | 0.03 | -0.01 | 0.02 | 0.01 | 0.01 |
| 234 | rs4930295 | G | -0.24 | 0.02 | -0.04 | 0.01 | 0.03 | 0.01 |
| 235 | rs4936099 | A | 0.17 | 0.02 | 0.01 | 0.01 | 0.02 | 0.01 |
| 236 | rs4952668 | A | -0.19 | 0.02 | -0.01 | 0.01 | 0.00 | 0.01 |
| 237 | rs4954192 | T | -0.12 | 0.02 | 0.01 | 0.01 | -0.02 | 0.01 |
| 238 | rs5012479 | G | -0.10 | 0.02 | 0.00 | 0.01 | 0.00 | 0.01 |
| 239 | rs504217 | T | 0.27 | 0.03 | -0.01 | 0.02 | -0.02 | 0.02 |
| 240 | rs504691 | A | -0.12 | 0.02 | -0.04 | 0.01 | 0.00 | 0.01 |
| 241 | rs509067 | C | 0.14 | 0.02 | 0.00 | 0.01 | -0.02 | 0.01 |
| 242 | rs520592 | G | 0.17 | 0.02 | 0.03 | 0.02 | -0.01 | 0.01 |
| 243 | rs55684003 | G | -0.12 | 0.02 | -0.01 | 0.01 | 0.00 | 0.01 |
| 244 | rs55770741 | T | -0.13 | 0.02 | -0.03 | 0.01 | 0.02 | 0.01 |
| 245 | rs55857306 | A | -0.52 | 0.02 | -0.02 | 0.01 | 0.02 | 0.01 |
| 246 | rs55944332 | G | 0.24 | 0.02 | -0.02 | 0.01 | 0.00 | 0.01 |
| 247 | rs55993676 | T | -0.21 | 0.02 | -0.01 | 0.01 | -0.01 | 0.01 |
| 248 | rs56809883 | T | 0.11 | 0.02 | 0.00 | 0.01 | 0.00 | 0.01 |
| 249 | rs58693787 | G | -0.16 | 0.02 | -0.01 | 0.01 | 0.01 | 0.01 |
| 250 | rs5992929 | T | 0.17 | 0.02 | 0.02 | 0.01 | 0.02 | 0.01 |
| 251 | rs602521 | A | 0.14 | 0.02 | -0.01 | 0.01 | 0.01 | 0.01 |
| 252 | rs6026739 | T | 0.50 | 0.03 | 0.05 | 0.02 | -0.01 | 0.01 |
| 253 | rs6031431 | G | 0.12 | 0.02 | 0.00 | 0.01 | 0.00 | 0.01 |
| 254 | rs604723 | C | 0.38 | 0.02 | 0.03 | 0.01 | -0.01 | 0.01 |
| 255 | rs6058261 | A | -0.12 | 0.02 | 0.00 | 0.01 | 0.01 | 0.01 |
| 256 | rs6078393 | G | -0.12 | 0.02 | 0.00 | 0.01 | 0.01 | 0.01 |
| 257 | rs6108168 | A | -0.19 | 0.02 | -0.02 | 0.01 | 0.00 | 0.01 |
| 258 | rs61789369 | G | 0.30 | 0.04 | 0.00 | 0.03 | -0.01 | 0.02 |
| 259 | rs61912333 | G | -0.12 | 0.02 | 0.01 | 0.01 | -0.01 | 0.01 |
| 260 | rs61948065 | C | 0.17 | 0.03 | 0.03 | 0.02 | 0.00 | 0.01 |
| 261 | rs62155750 | G | 0.22 | 0.02 | 0.02 | 0.01 | 0.00 | 0.01 |
| 262 | rs62158170 | G | -0.16 | 0.02 | 0.01 | 0.01 | -0.01 | 0.01 |
| 263 | rs62234672 | A | 0.12 | 0.02 | 0.02 | 0.01 | 0.00 | 0.01 |
| 264 | rs62301873 | G | 0.17 | 0.03 | 0.02 | 0.02 | 0.00 | 0.01 |
| 265 | rs62434124 | T | -0.49 | 0.03 | -0.05 | 0.02 | 0.00 | 0.02 |
| 266 | rs62503324 | T | 0.20 | 0.02 | -0.01 | 0.01 | 0.01 | 0.01 |
| 267 | rs636202 | C | -0.10 | 0.02 | 0.00 | 0.01 | 0.01 | 0.01 |
| 268 | rs6464165 | C | 0.22 | 0.02 | 0.01 | 0.01 | 0.02 | 0.01 |
| 269 | rs6487076 | G | -0.17 | 0.02 | 0.00 | 0.01 | 0.00 | 0.01 |
| 270 | rs6546810 | C | 0.12 | 0.02 | 0.02 | 0.01 | -0.01 | 0.01 |
| 271 | rs6556384 | A | -0.15 | 0.02 | -0.03 | 0.01 | 0.02 | 0.01 |
| 272 | rs6580970 | T | -0.17 | 0.02 | -0.01 | 0.01 | 0.02 | 0.01 |
| 273 | rs66682451 | G | -0.13 | 0.02 | -0.02 | 0.01 | 0.00 | 0.01 |
| 274 | rs6686889 | T | 0.19 | 0.02 | 0.02 | 0.01 | 0.00 | 0.01 |
| 275 | rs66887589 | C | 0.16 | 0.02 | 0.03 | 0.01 | 0.00 | 0.01 |
| 276 | rs6715901 | A | -0.14 | 0.02 | 0.00 | 0.01 | 0.02 | 0.01 |
| 277 | rs672272 | T | -0.19 | 0.02 | -0.01 | 0.01 | -0.02 | 0.01 |
| 278 | rs6735275 | C | -0.12 | 0.02 | -0.01 | 0.01 | -0.02 | 0.01 |
| 279 | rs6763931 | A | 0.14 | 0.02 | -0.02 | 0.01 | 0.03 | 0.01 |
| 280 | rs6777317 | A | 0.12 | 0.02 | -0.01 | 0.01 | 0.00 | 0.01 |
| 281 | rs6779368 | G | 0.18 | 0.02 | 0.00 | 0.01 | -0.02 | 0.01 |
| 282 | rs6795735 | T | -0.14 | 0.02 | 0.01 | 0.01 | 0.06 | 0.01 |
| 283 | rs6800730 | G | 0.25 | 0.02 | 0.02 | 0.01 | -0.02 | 0.01 |
| 284 | rs68085857 | T | 0.19 | 0.02 | 0.03 | 0.01 | 0.00 | 0.01 |
| 285 | rs682681 | C | 0.15 | 0.02 | -0.01 | 0.01 | 0.00 | 0.01 |
| 286 | rs6875967 | G | -0.13 | 0.02 | -0.01 | 0.01 | 0.00 | 0.01 |
| 287 | rs6934891 | A | 0.13 | 0.02 | 0.01 | 0.01 | 0.00 | 0.01 |
| 288 | rs6961048 | G | 0.27 | 0.03 | 0.01 | 0.02 | -0.02 | 0.01 |
| 289 | rs6983239 | T | 0.12 | 0.02 | 0.01 | 0.01 | -0.03 | 0.01 |
| 290 | rs7012891 | C | 0.14 | 0.02 | 0.03 | 0.01 | -0.02 | 0.01 |
| 291 | rs710249 | C | 0.15 | 0.02 | 0.01 | 0.01 | 0.02 | 0.01 |
| 292 | rs7106104 | C | 0.12 | 0.02 | 0.03 | 0.01 | -0.01 | 0.01 |
| 293 | rs710698 | G | -0.11 | 0.02 | 0.00 | 0.01 | 0.00 | 0.01 |
| 294 | rs7115331 | G | 0.13 | 0.02 | -0.02 | 0.01 | 0.02 | 0.01 |
| 295 | rs7132012 | G | -0.16 | 0.02 | -0.01 | 0.01 | 0.02 | 0.01 |
| 296 | rs7137828 | T | -0.50 | 0.02 | -0.07 | 0.01 | 0.02 | 0.01 |
| 297 | rs7192407 | C | -0.10 | 0.02 | 0.00 | 0.01 | 0.00 | 0.01 |
| 298 | rs7217916 | G | -0.11 | 0.02 | -0.02 | 0.01 | 0.02 | 0.01 |
| 299 | rs722783 | A | -0.21 | 0.02 | 0.00 | 0.01 | -0.01 | 0.01 |
| 300 | rs7235890 | T | -0.17 | 0.03 | 0.00 | 0.02 | -0.01 | 0.01 |
| 301 | rs7257694 | T | 0.18 | 0.02 | -0.01 | 0.01 | -0.01 | 0.01 |
| 302 | rs7259285 | A | 0.11 | 0.02 | 0.01 | 0.01 | -0.02 | 0.01 |
| 303 | rs7265695 | C | -0.20 | 0.02 | -0.02 | 0.01 | 0.00 | 0.01 |
| 304 | rs72719149 | C | 0.13 | 0.02 | 0.02 | 0.01 | -0.03 | 0.01 |
| 305 | rs7278003 | C | 0.13 | 0.02 | 0.04 | 0.01 | -0.02 | 0.01 |
| 306 | rs72831343 | G | -0.49 | 0.02 | -0.02 | 0.02 | 0.00 | 0.01 |
| 307 | rs72842207 | T | -0.21 | 0.02 | -0.02 | 0.01 | 0.02 | 0.01 |
| 308 | rs72976750 | C | 0.17 | 0.03 | 0.00 | 0.01 | 0.00 | 0.01 |
| 309 | rs7299936 | A | 0.18 | 0.02 | 0.01 | 0.01 | 0.01 | 0.01 |
| 310 | rs73036520 | C | 0.16 | 0.02 | 0.03 | 0.01 | -0.03 | 0.01 |
| 311 | rs7321688 | A | 0.15 | 0.02 | 0.01 | 0.01 | 0.01 | 0.01 |
| 312 | rs7324697 | A | 0.10 | 0.02 | 0.00 | 0.01 | 0.03 | 0.01 |
| 313 | rs73276406 | C | 0.16 | 0.02 | 0.04 | 0.01 | -0.03 | 0.01 |
| 314 | rs7350752 | A | -0.15 | 0.03 | 0.03 | 0.02 | -0.01 | 0.01 |
| 315 | rs7427249 | A | -0.11 | 0.02 | -0.01 | 0.01 | 0.00 | 0.01 |
| 316 | rs751984 | C | -0.39 | 0.03 | -0.03 | 0.01 | 0.04 | 0.01 |
| 317 | rs7524019 | T | 0.10 | 0.02 | 0.00 | 0.01 | 0.01 | 0.01 |
| 318 | rs75507123 | T | -0.14 | 0.03 | 0.01 | 0.02 | -0.02 | 0.01 |
| 319 | rs7569128 | A | 0.20 | 0.02 | 0.02 | 0.01 | 0.01 | 0.01 |
| 320 | rs7572130 | G | 0.18 | 0.03 | 0.05 | 0.02 | 0.01 | 0.01 |
| 321 | rs7576060 | T | -0.10 | 0.02 | -0.03 | 0.01 | 0.00 | 0.01 |
| 322 | rs7611674 | G | -0.16 | 0.02 | 0.01 | 0.01 | -0.01 | 0.01 |
| 323 | rs7623706 | G | -0.10 | 0.02 | -0.01 | 0.01 | -0.01 | 0.01 |
| 324 | rs76326501 | C | -0.36 | 0.03 | -0.02 | 0.02 | 0.01 | 0.01 |
| 325 | rs7694000 | T | 0.10 | 0.02 | -0.01 | 0.01 | -0.01 | 0.01 |
| 326 | rs76954792 | T | 0.12 | 0.02 | 0.02 | 0.01 | 0.00 | 0.01 |
| 327 | rs7788746 | T | -0.16 | 0.02 | -0.04 | 0.01 | -0.02 | 0.01 |
| 328 | rs7800558 | C | -0.10 | 0.02 | -0.01 | 0.01 | 0.01 | 0.01 |
| 329 | rs7805035 | A | 0.13 | 0.02 | -0.01 | 0.01 | 0.00 | 0.01 |
| 330 | rs78151625 | C | 0.19 | 0.02 | 0.01 | 0.01 | -0.01 | 0.01 |
| 331 | rs786921 | A | -0.11 | 0.02 | 0.00 | 0.01 | 0.01 | 0.01 |
| 332 | rs78809139 | A | -0.23 | 0.03 | 0.04 | 0.02 | 0.00 | 0.01 |
| 333 | rs78909293 | C | -0.32 | 0.04 | -0.07 | 0.02 | 0.03 | 0.02 |
| 334 | rs79044887 | G | -0.24 | 0.02 | -0.04 | 0.01 | 0.01 | 0.01 |
| 335 | rs7926335 | T | 0.18 | 0.02 | 0.02 | 0.01 | -0.01 | 0.01 |
| 336 | rs7933758 | T | -0.11 | 0.02 | -0.01 | 0.01 | 0.00 | 0.01 |
| 337 | rs7959649 | C | -0.12 | 0.02 | -0.01 | 0.01 | 0.01 | 0.01 |
| 338 | rs7967705 | C | -0.27 | 0.02 | 0.00 | 0.01 | 0.00 | 0.01 |
| 339 | rs79724577 | C | -0.14 | 0.02 | 0.04 | 0.02 | -0.01 | 0.01 |
| 340 | rs7992292 | A | 0.14 | 0.02 | 0.02 | 0.01 | -0.02 | 0.01 |
| 341 | rs8014182 | T | -0.19 | 0.03 | -0.01 | 0.01 | 0.03 | 0.01 |
| 342 | rs8078510 | A | -0.13 | 0.02 | -0.05 | 0.01 | 0.06 | 0.01 |
| 343 | rs824523 | A | 0.12 | 0.02 | -0.01 | 0.01 | -0.01 | 0.01 |
| 344 | rs881858 | A | 0.16 | 0.02 | 0.01 | 0.01 | 0.05 | 0.01 |
| 345 | rs882624 | T | -0.16 | 0.02 | 0.01 | 0.01 | 0.01 | 0.01 |
| 346 | rs917522 | T | 0.17 | 0.03 | 0.02 | 0.02 | 0.01 | 0.01 |
| 347 | rs9286351 | G | 0.14 | 0.02 | 0.02 | 0.01 | 0.00 | 0.01 |
| 348 | rs9326869 | C | -0.11 | 0.02 | 0.00 | 0.01 | 0.02 | 0.01 |
| 349 | rs9368 | A | 0.11 | 0.02 | 0.00 | 0.01 | 0.01 | 0.01 |
| 350 | rs9399137 | C | -0.11 | 0.02 | -0.02 | 0.01 | 0.00 | 0.01 |
| 351 | rs9406076 | T | 0.10 | 0.02 | 0.02 | 0.01 | 0.02 | 0.01 |
| 352 | rs9419374 | G | -0.12 | 0.02 | -0.02 | 0.01 | 0.00 | 0.01 |
| 353 | rs9467545 | T | 0.25 | 0.02 | 0.00 | 0.01 | -0.01 | 0.01 |
| 354 | rs9478282 | T | -0.20 | 0.03 | 0.00 | 0.02 | -0.02 | 0.01 |
| 355 | rs951914 | C | 0.19 | 0.02 | 0.01 | 0.01 | -0.01 | 0.01 |
| 356 | rs9526707 | A | -0.12 | 0.02 | -0.01 | 0.01 | -0.01 | 0.01 |
| 357 | rs962369 | C | -0.17 | 0.02 | 0.03 | 0.01 | -0.03 | 0.01 |
| 358 | rs964941 | A | 0.17 | 0.02 | 0.00 | 0.01 | 0.01 | 0.01 |
| 359 | rs9791312 | C | 0.12 | 0.02 | 0.01 | 0.01 | -0.01 | 0.01 |
| 360 | rs9841978 | A | 0.18 | 0.02 | 0.01 | 0.01 | -0.02 | 0.01 |
| 361 | rs9889262 | A | 0.23 | 0.02 | 0.04 | 0.01 | -0.01 | 0.01 |
| 362 | rs9893005 | G | 0.12 | 0.02 | 0.00 | 0.01 | 0.01 | 0.01 |
| 363 | rs9900637 | A | 0.10 | 0.02 | 0.00 | 0.01 | -0.03 | 0.01 |
| 364 | rs990619 | G | 0.16 | 0.02 | 0.03 | 0.01 | 0.00 | 0.01 |
| 365 | rs9918907 | G | 0.12 | 0.02 | 0.02 | 0.01 | 0.01 | 0.01 |
| 366 | rs9932220 | A | -0.16 | 0.02 | -0.02 | 0.01 | -0.01 | 0.01 |
| 367 | rs9937801 | C | -0.16 | 0.02 | -0.02 | 0.01 | 0.00 | 0.01 |

DBP: Diastolic blood pressure; MI: Myocardial infarction; T2DM: Type-2 diabetes

Table 39: Genetic association estimates for the effect of TG on MI, adjusted for T2DM. ea=effect allele, gx=TG, gy=MI, gz=T2DM, se=standard error

|  | SNP | ea | gx | gx_se | gy | gy_se | gz | gz_se |
| --- | --- | --- | --- | --- | --- | --- | --- | --- |
| 1 | rs10040328 | A | 0.02 | 0.00 | 0.01 | 0.01 | -0.02 | 0.01 |
| 2 | rs1009590 | C | 0.02 | 0.00 | -0.01 | 0.02 | -0.03 | 0.01 |
| 3 | rs1044808 | C | -0.03 | 0.00 | -0.01 | 0.02 | 0.05 | 0.01 |
| 4 | rs1045241 | T | -0.02 | 0.00 | -0.02 | 0.01 | 0.02 | 0.01 |
| 5 | rs10750766 | A | 0.02 | 0.00 | 0.03 | 0.01 | -0.03 | 0.01 |
| 6 | rs10772947 | G | -0.01 | 0.00 | 0.00 | 0.01 | 0.01 | 0.01 |
| 7 | rs10797119 | C | 0.02 | 0.00 | 0.02 | 0.01 | -0.01 | 0.01 |
| 8 | rs10838681 | A | -0.03 | 0.00 | 0.02 | 0.01 | 0.00 | 0.01 |
| 9 | rs10851698 | T | 0.02 | 0.00 | 0.01 | 0.01 | -0.02 | 0.01 |
| 10 | rs10872003 | A | -0.01 | 0.00 | 0.00 | 0.01 | 0.00 | 0.01 |
| 11 | rs10883026 | T | -0.02 | 0.00 | -0.02 | 0.01 | 0.02 | 0.01 |
| 12 | rs10957299 | G | -0.01 | 0.00 | -0.01 | 0.01 | 0.02 | 0.01 |
| 13 | rs11030107 | G | 0.02 | 0.00 | 0.02 | 0.01 | -0.03 | 0.01 |
| 14 | rs11045171 | G | -0.03 | 0.00 | 0.00 | 0.01 | 0.02 | 0.01 |
| 15 | rs11122450 | G | -0.05 | 0.00 | -0.02 | 0.01 | 0.01 | 0.01 |
| 16 | rs11187019 | G | -0.01 | 0.00 | -0.01 | 0.01 | 0.08 | 0.01 |
| 17 | rs11206374 | A | 0.03 | 0.00 | 0.02 | 0.01 | -0.06 | 0.01 |
| 18 | rs112424890 | T | 0.02 | 0.00 | 0.04 | 0.02 | -0.01 | 0.01 |
| 19 | rs1128249 | T | -0.04 | 0.00 | -0.01 | 0.01 | 0.07 | 0.01 |
| 20 | rs115271198 | T | -0.03 | 0.00 | -0.02 | 0.02 | 0.05 | 0.02 |
| 21 | rs11631625 | G | 0.01 | 0.00 | 0.01 | 0.01 | -0.02 | 0.01 |
| 22 | rs11705483 | A | 0.02 | 0.00 | 0.04 | 0.02 | -0.05 | 0.01 |
| 23 | rs11722924 | C | 0.01 | 0.00 | -0.01 | 0.01 | 0.00 | 0.01 |
| 24 | rs11752394 | G | 0.02 | 0.00 | 0.00 | 0.01 | -0.02 | 0.01 |
| 25 | rs12088739 | G | -0.03 | 0.00 | -0.05 | 0.02 | 0.09 | 0.01 |
| 26 | rs12119979 | G | 0.02 | 0.00 | 0.01 | 0.01 | -0.04 | 0.01 |
| 27 | rs12424054 | A | 0.02 | 0.00 | 0.00 | 0.01 | -0.02 | 0.01 |
| 28 | rs12446515 | T | -0.04 | 0.00 | -0.03 | 0.01 | 0.01 | 0.01 |
| 29 | rs12480662 | T | -0.02 | 0.00 | -0.02 | 0.01 | -0.01 | 0.01 |
| 30 | rs12513202 | T | 0.01 | 0.00 | -0.01 | 0.01 | -0.02 | 0.01 |
| 31 | rs12541912 | C | -0.10 | 0.00 | -0.03 | 0.01 | 0.03 | 0.01 |
| 32 | rs12686780 | T | 0.02 | 0.00 | 0.00 | 0.01 | -0.03 | 0.01 |
| 33 | rs12749691 | T | -0.02 | 0.00 | -0.02 | 0.01 | -0.01 | 0.01 |
| 34 | rs1279840 | C | 0.03 | 0.00 | 0.03 | 0.01 | -0.03 | 0.01 |
| 35 | rs12868517 | G | -0.01 | 0.00 | 0.01 | 0.01 | 0.03 | 0.01 |
| 36 | rs12880341 | C | 0.02 | 0.00 | 0.01 | 0.02 | -0.02 | 0.01 |
| 37 | rs1292065 | G | -0.02 | 0.00 | -0.02 | 0.01 | 0.00 | 0.01 |
| 38 | rs12928099 | A | -0.03 | 0.00 | -0.01 | 0.01 | 0.02 | 0.01 |
| 39 | rs13234131 | G | -0.13 | 0.00 | 0.01 | 0.02 | -0.03 | 0.01 |
| 40 | rs13269725 | G | 0.04 | 0.00 | 0.01 | 0.02 | -0.05 | 0.01 |
| 41 | rs13273454 | T | -0.06 | 0.00 | -0.03 | 0.01 | 0.02 | 0.01 |
| 42 | rs1340819 | C | -0.01 | 0.00 | 0.00 | 0.01 | 0.01 | 0.01 |
| 43 | rs1420384 | T | -0.01 | 0.00 | 0.00 | 0.01 | 0.03 | 0.01 |
| 44 | rs142047875 | T | -0.01 | 0.00 | -0.02 | 0.01 | 0.02 | 0.01 |
| 45 | rs1471251 | T | 0.04 | 0.00 | 0.01 | 0.01 | -0.01 | 0.01 |
| 46 | rs1473886 | T | -0.02 | 0.00 | 0.02 | 0.01 | 0.00 | 0.01 |
| 47 | rs149142833 | T | 0.02 | 0.00 | 0.01 | 0.02 | -0.04 | 0.01 |
| 48 | rs154735 | A | 0.03 | 0.00 | -0.01 | 0.02 | -0.01 | 0.02 |
| 49 | rs1549293 | T | -0.02 | 0.00 | -0.01 | 0.01 | 0.01 | 0.01 |
| 50 | rs1644005 | C | -0.01 | 0.00 | -0.01 | 0.01 | 0.04 | 0.01 |
| 51 | rs1684608 | A | 0.02 | 0.00 | 0.00 | 0.01 | 0.00 | 0.01 |
| 52 | rs1688043 | T | 0.03 | 0.00 | -0.03 | 0.02 | 0.01 | 0.01 |
| 53 | rs17052058 | G | -0.03 | 0.00 | -0.01 | 0.01 | 0.02 | 0.01 |
| 54 | rs17138358 | C | 0.02 | 0.00 | -0.02 | 0.01 | 0.00 | 0.01 |
| 55 | rs17311740 | T | -0.03 | 0.00 | 0.01 | 0.02 | 0.00 | 0.02 |
| 56 | rs17326656 | T | 0.02 | 0.00 | 0.01 | 0.01 | -0.01 | 0.01 |
| 57 | rs174574 | C | -0.05 | 0.00 | 0.02 | 0.01 | -0.02 | 0.01 |
| 58 | rs17496249 | G | -0.01 | 0.00 | -0.03 | 0.01 | 0.03 | 0.01 |
| 59 | rs17699425 | A | -0.03 | 0.00 | 0.05 | 0.03 | -0.03 | 0.02 |
| 60 | rs1790099 | T | 0.02 | 0.00 | 0.01 | 0.01 | -0.04 | 0.01 |
| 61 | rs1800978 | G | -0.03 | 0.00 | -0.04 | 0.01 | 0.02 | 0.01 |
| 62 | rs1853413 | G | -0.01 | 0.00 | 0.00 | 0.01 | 0.01 | 0.01 |
| 63 | rs1924485 | T | -0.02 | 0.00 | 0.00 | 0.01 | -0.01 | 0.01 |
| 64 | rs1928496 | T | 0.02 | 0.00 | -0.01 | 0.01 | 0.02 | 0.01 |
| 65 | rs199607859 | T | -0.03 | 0.00 | 0.00 | 0.01 | 0.03 | 0.01 |
| 66 | rs199795230 | T | 0.03 | 0.00 | -0.03 | 0.01 | 0.01 | 0.01 |
| 67 | rs2068888 | A | -0.03 | 0.00 | -0.04 | 0.01 | 0.00 | 0.01 |
| 68 | rs2070971 | T | 0.02 | 0.00 | 0.00 | 0.02 | -0.02 | 0.01 |
| 69 | rs2071887 | A | 0.02 | 0.00 | -0.01 | 0.01 | 0.00 | 0.01 |
| 70 | rs2081687 | C | -0.03 | 0.00 | -0.01 | 0.01 | -0.01 | 0.01 |
| 71 | rs2114273 | C | 0.02 | 0.00 | 0.00 | 0.01 | 0.00 | 0.01 |
| 72 | rs213479 | T | -0.02 | 0.00 | 0.00 | 0.01 | -0.01 | 0.01 |
| 73 | rs2139980 | A | -0.01 | 0.00 | -0.01 | 0.01 | 0.03 | 0.01 |
| 74 | rs2267373 | T | 0.02 | 0.00 | 0.01 | 0.01 | -0.01 | 0.01 |
| 75 | rs2510344 | C | -0.02 | 0.00 | -0.01 | 0.01 | 0.03 | 0.01 |
| 76 | rs261342 | C | -0.05 | 0.00 | -0.03 | 0.01 | 0.00 | 0.01 |
| 77 | rs2652812 | T | -0.02 | 0.00 | -0.03 | 0.01 | 0.02 | 0.01 |
| 78 | rs2694913 | C | 0.01 | 0.00 | 0.00 | 0.01 | 0.00 | 0.01 |
| 79 | rs2699805 | A | -0.02 | 0.00 | -0.03 | 0.01 | 0.03 | 0.01 |
| 80 | rs2723067 | G | -0.02 | 0.00 | 0.01 | 0.01 | 0.05 | 0.01 |
| 81 | rs2773469 | G | -0.02 | 0.00 | 0.02 | 0.01 | 0.02 | 0.01 |
| 82 | rs2925979 | C | -0.03 | 0.00 | -0.01 | 0.01 | 0.05 | 0.01 |
| 83 | rs2943645 | T | 0.04 | 0.00 | 0.03 | 0.01 | -0.09 | 0.01 |
| 84 | rs2954017 | C | -0.09 | 0.00 | -0.05 | 0.01 | 0.00 | 0.01 |
| 85 | rs296884 | T | -0.02 | 0.00 | -0.01 | 0.01 | 0.01 | 0.01 |
| 86 | rs2971669 | T | 0.02 | 0.00 | 0.01 | 0.01 | -0.05 | 0.01 |
| 87 | rs34682685 | A | 0.03 | 0.00 | 0.02 | 0.02 | -0.01 | 0.01 |
| 88 | rs35859536 | T | -0.02 | 0.00 | 0.00 | 0.01 | 0.12 | 0.01 |
| 89 | rs3731696 | G | 0.02 | 0.00 | -0.02 | 0.01 | -0.04 | 0.01 |
| 90 | rs38205 | C | -0.01 | 0.00 | -0.02 | 0.01 | 0.04 | 0.01 |
| 91 | rs3826043 | T | -0.01 | 0.00 | -0.02 | 0.01 | 0.03 | 0.01 |
| 92 | rs41749 | A | -0.01 | 0.00 | 0.00 | 0.01 | 0.01 | 0.01 |
| 93 | rs4410790 | C | 0.02 | 0.00 | 0.03 | 0.01 | -0.02 | 0.01 |
| 94 | rs45487899 | T | -0.04 | 0.01 | -0.06 | 0.03 | 0.01 | 0.02 |
| 95 | rs4646246 | G | 0.03 | 0.00 | 0.04 | 0.01 | -0.03 | 0.01 |
| 96 | rs4675812 | A | -0.01 | 0.00 | 0.02 | 0.01 | 0.01 | 0.01 |
| 97 | rs4710938 | G | -0.01 | 0.00 | 0.00 | 0.01 | 0.07 | 0.01 |
| 98 | rs4731701 | T | -0.03 | 0.00 | -0.01 | 0.01 | 0.06 | 0.01 |
| 99 | rs4760254 | C | -0.03 | 0.00 | 0.00 | 0.01 | 0.01 | 0.01 |
| 100 | rs4761234 | C | -0.02 | 0.00 | -0.02 | 0.01 | 0.01 | 0.01 |
| 101 | rs4789182 | A | 0.02 | 0.00 | -0.02 | 0.01 | -0.01 | 0.01 |
| 102 | rs4841580 | C | -0.02 | 0.00 | 0.01 | 0.01 | -0.03 | 0.01 |
| 103 | rs4843754 | G | 0.01 | 0.00 | 0.00 | 0.01 | -0.02 | 0.01 |
| 104 | rs4930724 | C | -0.03 | 0.00 | -0.03 | 0.01 | 0.03 | 0.01 |
| 105 | rs4969179 | G | -0.02 | 0.00 | -0.01 | 0.01 | 0.02 | 0.01 |
| 106 | rs5005705 | A | -0.02 | 0.00 | -0.02 | 0.01 | 0.04 | 0.01 |
| 107 | rs56030759 | C | 0.04 | 0.00 | 0.00 | 0.02 | -0.01 | 0.02 |
| 108 | rs57232565 | T | 0.19 | 0.01 | 0.05 | 0.02 | 0.00 | 0.01 |
| 109 | rs5755799 | G | 0.01 | 0.00 | 0.01 | 0.01 | -0.02 | 0.01 |
| 110 | rs58284370 | A | 0.03 | 0.00 | -0.03 | 0.02 | 0.01 | 0.01 |
| 111 | rs58542926 | T | -0.11 | 0.00 | -0.05 | 0.02 | -0.09 | 0.01 |
| 112 | rs591939 | G | 0.02 | 0.00 | 0.04 | 0.01 | -0.05 | 0.01 |
| 113 | rs593979 | C | -0.02 | 0.00 | -0.02 | 0.01 | 0.03 | 0.01 |
| 114 | rs6066138 | A | -0.02 | 0.00 | -0.01 | 0.01 | 0.05 | 0.01 |
| 115 | rs6073958 | C | 0.06 | 0.00 | -0.03 | 0.01 | 0.02 | 0.01 |
| 116 | rs60856912 | T | 0.03 | 0.00 | 0.03 | 0.01 | -0.04 | 0.01 |
| 117 | rs6093446 | A | 0.01 | 0.00 | 0.04 | 0.01 | -0.01 | 0.01 |
| 118 | rs62102718 | T | 0.02 | 0.00 | 0.03 | 0.01 | -0.04 | 0.01 |
| 119 | rs62112763 | G | 0.02 | 0.00 | 0.00 | 0.01 | -0.02 | 0.01 |
| 120 | rs631106 | A | -0.08 | 0.00 | 0.00 | 0.01 | 0.00 | 0.01 |
| 121 | rs6486122 | T | 0.02 | 0.00 | 0.03 | 0.01 | -0.03 | 0.01 |
| 122 | rs6492721 | C | -0.01 | 0.00 | -0.01 | 0.01 | -0.03 | 0.01 |
| 123 | rs6506033 | T | -0.03 | 0.00 | -0.01 | 0.02 | 0.02 | 0.02 |
| 124 | rs6547692 | A | -0.09 | 0.00 | 0.00 | 0.01 | -0.05 | 0.01 |
| 125 | rs6554198 | A | -0.01 | 0.00 | -0.02 | 0.01 | 0.00 | 0.01 |
| 126 | rs6708784 | G | -0.01 | 0.00 | 0.01 | 0.01 | 0.04 | 0.01 |
| 127 | rs676210 | A | -0.08 | 0.00 | -0.01 | 0.01 | -0.01 | 0.01 |
| 128 | rs6882076 | C | 0.04 | 0.00 | 0.01 | 0.01 | 0.02 | 0.01 |
| 129 | rs6916318 | T | 0.03 | 0.00 | 0.01 | 0.01 | 0.01 | 0.01 |
| 130 | rs7117238 | A | -0.02 | 0.00 | -0.04 | 0.01 | 0.01 | 0.01 |
| 131 | rs7123454 | A | -0.13 | 0.00 | 0.00 | 0.01 | 0.01 | 0.01 |
| 132 | rs71603401 | G | 0.03 | 0.00 | 0.03 | 0.01 | -0.02 | 0.01 |
| 133 | rs7167078 | G | -0.02 | 0.00 | 0.02 | 0.01 | 0.03 | 0.01 |
| 134 | rs7274718 | A | 0.02 | 0.00 | -0.01 | 0.01 | 0.01 | 0.01 |
| 135 | rs729761 | G | 0.02 | 0.00 | 0.02 | 0.01 | 0.05 | 0.01 |
| 136 | rs7298844 | G | 0.02 | 0.00 | -0.03 | 0.01 | -0.03 | 0.01 |
| 137 | rs73025562 | A | 0.02 | 0.00 | -0.02 | 0.01 | 0.00 | 0.01 |
| 138 | rs73243877 | G | 0.03 | 0.00 | 0.02 | 0.01 | -0.02 | 0.01 |
| 139 | rs7529073 | C | -0.01 | 0.00 | -0.01 | 0.01 | 0.05 | 0.01 |
| 140 | rs76669111 | T | -0.02 | 0.00 | 0.01 | 0.02 | -0.02 | 0.01 |
| 141 | rs7735249 | G | 0.03 | 0.00 | 0.06 | 0.02 | -0.04 | 0.01 |
| 142 | rs7826687 | G | 0.03 | 0.00 | 0.00 | 0.01 | -0.02 | 0.01 |
| 143 | rs7896783 | A | -0.03 | 0.00 | 0.01 | 0.01 | 0.01 | 0.01 |
| 144 | rs79291519 | T | -0.06 | 0.01 | -0.02 | 0.02 | 0.02 | 0.02 |
| 145 | rs863750 | T | 0.03 | 0.00 | 0.02 | 0.01 | -0.05 | 0.01 |
| 146 | rs867939 | A | -0.02 | 0.00 | -0.01 | 0.01 | 0.02 | 0.01 |
| 147 | rs917195 | T | -0.02 | 0.00 | 0.01 | 0.01 | 0.05 | 0.01 |
| 148 | rs9425589 | A | -0.01 | 0.00 | -0.02 | 0.01 | 0.04 | 0.01 |
| 149 | rs9692598 | G | -0.01 | 0.00 | 0.00 | 0.01 | 0.01 | 0.01 |
| 150 | rs9836434 | T | 0.01 | 0.00 | 0.01 | 0.01 | -0.02 | 0.01 |
| 151 | rs9844972 | C | 0.04 | 0.00 | 0.04 | 0.02 | -0.10 | 0.01 |

TG: Triglycerides; MI: Myocardial infarction; T2DM: Type-2 diabetes

Table 40: Genetic association estimates for the effect of HDL on MI, adjusted for T2DM. ea=effect allele, gx=HDL, gy=MI, gz=T2DM, se=standard error

|  | SNP | ea | gx | gx_se | gy | gy_se | gz | gz_se |
| --- | --- | --- | --- | --- | --- | --- | --- | --- |
| 1 | rs117199990 | T | 0.19 | 0.02 | -0.06 | 0.02 | 0.06 | 0.01 |
| 2 | rs1532085 | G | -0.10 | 0.02 | -0.01 | 0.01 | -0.02 | 0.01 |
| 3 | rs183130 | T | 0.24 | 0.02 | -0.03 | 0.01 | 0.01 | 0.01 |
| 4 | rs964184 | C | 0.14 | 0.02 | -0.05 | 0.01 | 0.03 | 0.01 |

HDL: High-density lipoprotein; MI: Myocardial infarction; T2DM: Type-2 diabetes

Table 41: Genetic association estimates for the effect of WHR on MI, adjusted for T2DM. ea=effect allele, gx=WHR, gy=MI, gz=T2DM, se=standard error

|  | SNP | ea | gx | gx_se | gy | gy_se | gz | gz_se |
| --- | --- | --- | --- | --- | --- | --- | --- | --- |
| 1 | rs10245353 | A | 0.04 | 0.00 | 0.00 | 0.01 | -0.02 | 0.01 |
| 2 | rs10804591 | A | 0.02 | 0.00 | 0.02 | 0.01 | -0.03 | 0.01 |
| 3 | rs10842707 | T | 0.03 | 0.00 | 0.00 | 0.01 | -0.05 | 0.01 |
| 4 | rs10991437 | A | 0.03 | 0.01 | 0.02 | 0.02 | -0.02 | 0.01 |
| 5 | rs11231693 | A | 0.04 | 0.01 | 0.02 | 0.02 | -0.06 | 0.02 |
| 6 | rs1128249 | T | -0.03 | 0.00 | -0.01 | 0.01 | 0.07 | 0.01 |
| 7 | rs12143789 | C | 0.02 | 0.00 | -0.02 | 0.01 | 0.01 | 0.01 |
| 8 | rs12679556 | G | 0.03 | 0.00 | 0.02 | 0.01 | -0.03 | 0.01 |
| 9 | rs1294410 | C | 0.03 | 0.00 | -0.02 | 0.01 | 0.01 | 0.01 |
| 10 | rs1385167 | G | 0.03 | 0.00 | 0.01 | 0.01 | -0.02 | 0.01 |
| 11 | rs1440372 | C | 0.02 | 0.00 | 0.01 | 0.01 | -0.02 | 0.01 |
| 12 | rs1569135 | G | -0.02 | 0.00 | -0.02 | 0.01 | 0.01 | 0.01 |
| 13 | rs17451107 | C | -0.03 | 0.00 | 0.02 | 0.01 | -0.02 | 0.01 |
| 14 | rs17819328 | G | 0.02 | 0.00 | 0.04 | 0.01 | -0.03 | 0.01 |
| 15 | rs1936805 | T | 0.04 | 0.00 | 0.00 | 0.01 | 0.01 | 0.01 |
| 16 | rs2071449 | A | 0.03 | 0.00 | -0.02 | 0.01 | 0.02 | 0.01 |
| 17 | rs2276824 | G | -0.02 | 0.00 | 0.00 | 0.01 | 0.00 | 0.01 |
| 18 | rs2294239 | G | -0.03 | 0.00 | -0.01 | 0.01 | -0.01 | 0.01 |
| 19 | rs2645294 | T | 0.03 | 0.00 | -0.01 | 0.01 | 0.00 | 0.01 |
| 20 | rs2820443 | C | -0.04 | 0.00 | 0.00 | 0.01 | 0.05 | 0.01 |
| 21 | rs303084 | A | 0.02 | 0.00 | -0.01 | 0.01 | -0.04 | 0.01 |
| 22 | rs4081724 | A | -0.04 | 0.01 | -0.03 | 0.02 | -0.02 | 0.01 |
| 23 | rs459193 | G | -0.03 | 0.00 | 0.03 | 0.01 | -0.07 | 0.01 |
| 24 | rs4765219 | A | -0.03 | 0.00 | -0.03 | 0.01 | 0.03 | 0.01 |
| 25 | rs6772129 | G | -0.04 | 0.00 | 0.00 | 0.01 | 0.06 | 0.01 |
| 26 | rs714515 | A | -0.03 | 0.00 | -0.02 | 0.01 | 0.03 | 0.01 |
| 27 | rs7705502 | A | 0.03 | 0.00 | -0.01 | 0.01 | -0.01 | 0.01 |
| 28 | rs8030605 | A | 0.03 | 0.01 | -0.01 | 0.01 | -0.01 | 0.01 |
| 29 | rs878639 | G | -0.02 | 0.00 | -0.02 | 0.01 | -0.02 | 0.01 |
| 30 | rs979012 | C | -0.03 | 0.00 | 0.01 | 0.01 | 0.00 | 0.01 |

WHR: Waist-hip-ratio; MI: Myocardial infarction; T2DM: Type-2 diabetes

Table 42: Genetic association estimates for the effect of insulin sensitivity on MI, adjusted for T2DM. ea=effect allele, gx=insulin sensitivity, gy=MI, gz=T2DM, se=standard error

|  | SNP | ea | gx | gx_se | gy | gy_se | gz | gz_se |
| --- | --- | --- | --- | --- | --- | --- | --- | --- |
| 1 | rs10483182 | A | -2.00 | 0.26 | 0.04 | 0.04 | -0.01 | 0.03 |
| 2 | rs10495667 | A | -0.90 | 0.18 | 0.00 | 0.03 | -0.02 | 0.02 |
| 3 | rs10506418 | A | -0.80 | 0.25 | 0.03 | 0.03 | 0.03 | 0.02 |
| 4 | rs11594101 | G | -1.60 | 0.25 | 0.03 | 0.05 | 0.04 | 0.03 |
| 5 | rs11790816 | T | -1.40 | 0.26 | -0.05 | 0.03 | 0.00 | 0.02 |
| 6 | rs12583553 | T | -1.00 | 0.19 | 0.00 | 0.03 | 0.02 | 0.03 |
| 7 | rs1857095 | T | 0.88 | 0.25 | 0.05 | 0.02 | 0.03 | 0.03 |
| 8 | rs2828537 | T | -1.10 | 0.17 | 0.04 | 0.02 | -0.01 | 0.02 |
| 9 | rs2972146 | T | 0.14 | 0.07 | 0.04 | 0.01 | -0.09 | 0.01 |
| 10 | rs4078023 | T | 1.30 | 0.23 | -0.06 | 0.03 | -0.06 | 0.04 |
| 11 | rs6027072 | A | -0.48 | 0.18 | -0.01 | 0.03 | 0.03 | 0.02 |

MI: Myocardial infarction; T2DM: Type-2 diabetes

Table 43: Genetic association estimates for the effect of SBP on stroke, adjusted for T2DM. ea=effect allele, gx=SBP, gy=stroke, gz=T2DM, se=standard error

|  | SNP | ea | gx | gx_se | gy | gy_se | gz | gz_se |
| --- | --- | --- | --- | --- | --- | --- | --- | --- |
| 1 | rs1000423 | T | 0.41 | 0.03 | 0.02 | 0.01 | 0.00 | 0.01 |
| 2 | rs10008637 | C | -0.22 | 0.03 | -0.01 | 0.01 | 0.00 | 0.01 |
| 3 | rs10045307 | G | 0.20 | 0.04 | 0.01 | 0.01 | -0.01 | 0.01 |
| 4 | rs10048404 | T | -0.26 | 0.03 | -0.01 | 0.01 | 0.02 | 0.01 |
| 5 | rs1006545 | T | 0.68 | 0.05 | -0.03 | 0.01 | 0.03 | 0.01 |
| 6 | rs1010064 | C | -0.36 | 0.04 | 0.01 | 0.01 | 0.02 | 0.01 |
| 7 | rs1012089 | G | 0.19 | 0.03 | -0.02 | 0.01 | 0.01 | 0.01 |
| 8 | rs10188003 | T | 0.19 | 0.03 | 0.00 | 0.01 | 0.00 | 0.01 |
| 9 | rs10207726 | T | -0.21 | 0.03 | -0.01 | 0.01 | 0.02 | 0.01 |
| 10 | rs10224210 | C | 0.38 | 0.03 | 0.01 | 0.01 | 0.02 | 0.01 |
| 11 | rs1044822 | T | -0.25 | 0.04 | -0.02 | 0.01 | 0.02 | 0.01 |
| 12 | rs10460108 | G | -0.21 | 0.03 | -0.01 | 0.01 | 0.00 | 0.01 |
| 13 | rs1049212 | G | 0.30 | 0.03 | 0.01 | 0.01 | 0.01 | 0.01 |
| 14 | rs10501410 | A | 0.41 | 0.06 | -0.02 | 0.01 | -0.01 | 0.02 |
| 15 | rs1052501 | T | 0.23 | 0.04 | 0.04 | 0.01 | 0.01 | 0.01 |
| 16 | rs10749572 | T | -0.20 | 0.03 | -0.01 | 0.01 | 0.00 | 0.01 |
| 17 | rs10750441 | T | 0.18 | 0.03 | 0.00 | 0.01 | -0.01 | 0.01 |
| 18 | rs10777213 | A | -0.18 | 0.03 | -0.03 | 0.01 | 0.01 | 0.01 |
| 19 | rs10779795 | G | -0.22 | 0.03 | 0.01 | 0.01 | 0.01 | 0.01 |
| 20 | rs10782230 | A | 0.21 | 0.03 | 0.01 | 0.01 | -0.01 | 0.01 |
| 21 | rs10804330 | C | -0.24 | 0.03 | -0.02 | 0.01 | 0.07 | 0.01 |
| 22 | rs10914124 | C | -0.23 | 0.03 | 0.00 | 0.01 | 0.00 | 0.01 |
| 23 | rs10941043 | G | 0.26 | 0.03 | 0.00 | 0.01 | -0.01 | 0.01 |
| 24 | rs10980408 | C | 0.76 | 0.08 | -0.01 | 0.02 | -0.03 | 0.02 |
| 25 | rs11097909 | C | 0.36 | 0.04 | 0.03 | 0.01 | 0.00 | 0.01 |
| 26 | rs11120093 | T | -0.18 | 0.03 | 0.00 | 0.01 | -0.01 | 0.01 |
| 27 | rs11159091 | A | 0.20 | 0.03 | 0.01 | 0.01 | 0.01 | 0.01 |
| 28 | rs111866816 | T | 0.36 | 0.06 | -0.01 | 0.02 | -0.02 | 0.02 |
| 29 | rs11191580 | C | -1.10 | 0.06 | 0.00 | 0.01 | 0.00 | 0.01 |
| 30 | rs11210029 | G | 0.20 | 0.03 | 0.01 | 0.01 | 0.01 | 0.01 |
| 31 | rs11222084 | T | 0.34 | 0.03 | -0.01 | 0.01 | -0.02 | 0.01 |
| 32 | rs11241313 | T | -0.21 | 0.03 | -0.02 | 0.01 | 0.00 | 0.01 |
| 33 | rs112509803 | C | -0.26 | 0.05 | -0.04 | 0.01 | 0.01 | 0.01 |
| 34 | rs11252324 | T | -0.42 | 0.06 | -0.03 | 0.02 | -0.01 | 0.01 |
| 35 | rs115262049 | T | -0.59 | 0.06 | 0.00 | 0.02 | 0.02 | 0.01 |
| 36 | rs1154214 | G | 0.20 | 0.03 | 0.00 | 0.01 | 0.00 | 0.01 |
| 37 | rs11585169 | A | 0.18 | 0.03 | 0.00 | 0.01 | -0.02 | 0.01 |
| 38 | rs11592107 | A | 0.30 | 0.03 | 0.01 | 0.01 | -0.03 | 0.01 |
| 39 | rs11604357 | A | -0.28 | 0.04 | -0.02 | 0.01 | 0.00 | 0.01 |
| 40 | rs11636952 | C | -0.53 | 0.03 | -0.02 | 0.01 | 0.01 | 0.01 |
| 41 | rs1169078 | G | 0.20 | 0.03 | 0.01 | 0.01 | 0.00 | 0.01 |
| 42 | rs11694601 | G | 0.19 | 0.03 | 0.01 | 0.01 | -0.01 | 0.01 |
| 43 | rs11834380 | A | -0.28 | 0.05 | -0.01 | 0.01 | 0.05 | 0.01 |
| 44 | rs11874246 | T | 0.29 | 0.03 | 0.00 | 0.01 | -0.01 | 0.01 |
| 45 | rs11925504 | A | -0.29 | 0.03 | -0.01 | 0.01 | 0.03 | 0.01 |
| 46 | rs11960210 | C | -0.47 | 0.03 | -0.02 | 0.01 | 0.02 | 0.01 |
| 47 | rs11977526 | A | -0.32 | 0.03 | -0.02 | 0.01 | 0.01 | 0.01 |
| 48 | rs1199330 | G | 0.27 | 0.05 | 0.00 | 0.01 | -0.03 | 0.01 |
| 49 | rs12042924 | C | 0.18 | 0.03 | -0.01 | 0.01 | -0.02 | 0.01 |
| 50 | rs1209384 | G | -0.26 | 0.03 | -0.01 | 0.01 | -0.01 | 0.01 |
| 51 | rs12136922 | A | 0.20 | 0.03 | 0.00 | 0.01 | -0.01 | 0.01 |
| 52 | rs12255372 | T | 0.24 | 0.03 | 0.02 | 0.01 | -0.26 | 0.01 |
| 53 | rs12258967 | G | -0.63 | 0.03 | -0.03 | 0.01 | -0.02 | 0.01 |
| 54 | rs12264186 | T | 0.21 | 0.04 | -0.02 | 0.01 | -0.01 | 0.01 |
| 55 | rs12321 | C | -0.23 | 0.03 | 0.00 | 0.01 | -0.01 | 0.01 |
| 56 | rs12426261 | G | -0.38 | 0.03 | 0.00 | 0.01 | 0.00 | 0.01 |
| 57 | rs12464602 | A | -0.24 | 0.03 | -0.02 | 0.01 | 0.00 | 0.01 |
| 58 | rs12509595 | C | 0.84 | 0.03 | 0.03 | 0.01 | -0.02 | 0.01 |
| 59 | rs12511987 | G | 0.23 | 0.04 | 0.01 | 0.01 | -0.01 | 0.01 |
| 60 | rs12610654 | G | -0.23 | 0.03 | -0.01 | 0.01 | 0.00 | 0.01 |
| 61 | rs12637573 | G | 0.17 | 0.03 | 0.00 | 0.01 | -0.01 | 0.01 |
| 62 | rs12643599 | G | -0.31 | 0.03 | -0.02 | 0.01 | 0.02 | 0.01 |
| 63 | rs12656497 | C | 0.64 | 0.03 | 0.03 | 0.01 | 0.00 | 0.01 |
| 64 | rs12657950 | T | 0.46 | 0.06 | 0.01 | 0.01 | 0.01 | 0.02 |
| 65 | rs12661036 | C | 0.21 | 0.04 | 0.01 | 0.01 | -0.02 | 0.01 |
| 66 | rs12668436 | C | 0.22 | 0.04 | 0.01 | 0.01 | 0.00 | 0.01 |
| 67 | rs12693982 | T | 0.26 | 0.03 | 0.00 | 0.01 | 0.02 | 0.01 |
| 68 | rs12731646 | T | -0.19 | 0.03 | -0.02 | 0.01 | 0.01 | 0.01 |
| 69 | rs1275985 | T | -0.54 | 0.03 | -0.05 | 0.01 | -0.01 | 0.01 |
| 70 | rs12883810 | T | -0.24 | 0.04 | -0.02 | 0.01 | -0.02 | 0.01 |
| 71 | rs12906962 | C | 0.27 | 0.03 | 0.00 | 0.01 | -0.01 | 0.01 |
| 72 | rs1290784 | T | 0.41 | 0.03 | 0.02 | 0.01 | -0.01 | 0.01 |
| 73 | rs1290933 | A | -0.28 | 0.03 | -0.02 | 0.01 | 0.02 | 0.01 |
| 74 | rs12926550 | A | -0.25 | 0.03 | -0.03 | 0.01 | 0.02 | 0.01 |
| 75 | rs1293969 | C | 0.20 | 0.03 | 0.02 | 0.01 | 0.02 | 0.01 |
| 76 | rs13016772 | T | 0.25 | 0.04 | 0.02 | 0.01 | -0.02 | 0.01 |
| 77 | rs13091418 | G | 0.22 | 0.03 | 0.01 | 0.01 | -0.02 | 0.01 |
| 78 | rs13107261 | A | -0.18 | 0.03 | 0.00 | 0.01 | 0.01 | 0.01 |
| 79 | rs13204703 | C | -0.20 | 0.04 | 0.01 | 0.01 | 0.01 | 0.01 |
| 80 | rs13253358 | T | 0.21 | 0.03 | -0.01 | 0.01 | 0.00 | 0.01 |
| 81 | rs1332813 | C | -0.22 | 0.03 | 0.00 | 0.01 | 0.00 | 0.01 |
| 82 | rs13358657 | G | 0.39 | 0.04 | 0.02 | 0.01 | -0.01 | 0.01 |
| 83 | rs1340030 | C | -0.19 | 0.03 | 0.00 | 0.01 | 0.02 | 0.01 |
| 84 | rs13412750 | A | -0.29 | 0.03 | -0.01 | 0.01 | -0.01 | 0.01 |
| 85 | rs13420463 | G | -0.31 | 0.04 | 0.01 | 0.01 | 0.01 | 0.01 |
| 86 | rs1375564 | T | 0.26 | 0.03 | -0.01 | 0.01 | 0.01 | 0.01 |
| 87 | rs1382472 | A | -0.19 | 0.03 | 0.01 | 0.01 | 0.00 | 0.01 |
| 88 | rs1408945 | T | -0.32 | 0.03 | -0.02 | 0.01 | 0.00 | 0.01 |
| 89 | rs1410222 | T | 0.22 | 0.04 | 0.00 | 0.01 | 0.01 | 0.01 |
| 90 | rs1422279 | T | 0.33 | 0.03 | 0.02 | 0.01 | 0.00 | 0.01 |
| 91 | rs1433121 | T | -0.23 | 0.03 | 0.00 | 0.01 | -0.02 | 0.01 |
| 92 | rs1437649 | A | -0.22 | 0.04 | -0.01 | 0.01 | 0.01 | 0.01 |
| 93 | rs146550789 | C | 0.48 | 0.08 | -0.01 | 0.02 | 0.04 | 0.02 |
| 94 | rs148140538 | T | -0.33 | 0.06 | 0.00 | 0.02 | 0.00 | 0.01 |
| 95 | rs1493132 | C | 0.18 | 0.03 | 0.00 | 0.01 | -0.01 | 0.01 |
| 96 | rs1544861 | C | -0.20 | 0.03 | 0.02 | 0.01 | -0.02 | 0.01 |
| 97 | rs1551355 | T | 0.21 | 0.04 | -0.02 | 0.01 | 0.00 | 0.01 |
| 98 | rs1565440 | A | 0.17 | 0.03 | 0.00 | 0.01 | -0.01 | 0.01 |
| 99 | rs1575290 | T | 0.20 | 0.03 | 0.00 | 0.01 | 0.00 | 0.01 |
| 100 | rs1623474 | T | 0.38 | 0.03 | 0.01 | 0.01 | 0.00 | 0.01 |
| 101 | rs1630736 | T | -0.17 | 0.03 | 0.00 | 0.01 | 0.01 | 0.01 |
| 102 | rs1664781 | A | 0.26 | 0.03 | 0.01 | 0.01 | -0.06 | 0.01 |
| 103 | rs17010957 | C | 0.53 | 0.04 | 0.01 | 0.01 | 0.01 | 0.01 |
| 104 | rs17035181 | G | -0.31 | 0.04 | 0.00 | 0.01 | 0.04 | 0.01 |
| 105 | rs17080102 | C | -0.81 | 0.06 | -0.03 | 0.02 | 0.00 | 0.02 |
| 106 | rs17245822 | C | 0.19 | 0.03 | 0.00 | 0.01 | -0.02 | 0.01 |
| 107 | rs17249754 | A | -0.84 | 0.04 | -0.04 | 0.01 | -0.01 | 0.01 |
| 108 | rs17257081 | G | -0.23 | 0.04 | 0.02 | 0.01 | -0.02 | 0.01 |
| 109 | rs17608766 | C | 0.69 | 0.04 | 0.05 | 0.01 | 0.00 | 0.01 |
| 110 | rs177551 | A | 0.37 | 0.04 | 0.02 | 0.01 | 0.00 | 0.01 |
| 111 | rs17760259 | C | 0.27 | 0.03 | 0.00 | 0.01 | -0.01 | 0.01 |
| 112 | rs17762 | A | 0.41 | 0.06 | 0.01 | 0.01 | -0.02 | 0.01 |
| 113 | rs17807723 | A | -0.27 | 0.04 | -0.02 | 0.01 | -0.01 | 0.01 |
| 114 | rs17812022 | T | -0.36 | 0.05 | -0.01 | 0.02 | 0.00 | 0.01 |
| 115 | rs1786345 | C | -0.21 | 0.03 | -0.03 | 0.01 | 0.01 | 0.01 |
| 116 | rs1814951 | A | -0.32 | 0.05 | -0.01 | 0.01 | 0.00 | 0.01 |
| 117 | rs1821002 | G | -0.38 | 0.03 | -0.02 | 0.01 | 0.04 | 0.01 |
| 118 | rs1848994 | A | 0.20 | 0.03 | -0.02 | 0.01 | 0.02 | 0.01 |
| 119 | rs1870735 | G | -0.21 | 0.03 | -0.01 | 0.01 | 0.00 | 0.01 |
| 120 | rs1871190 | T | 0.20 | 0.03 | -0.01 | 0.01 | -0.01 | 0.01 |
| 121 | rs1882212 | G | -0.28 | 0.04 | 0.01 | 0.01 | 0.01 | 0.01 |
| 122 | rs1882961 | T | 0.24 | 0.03 | 0.02 | 0.01 | 0.01 | 0.01 |
| 123 | rs1889785 | A | 0.18 | 0.03 | 0.00 | 0.01 | -0.01 | 0.01 |
| 124 | rs1906672 | A | 0.30 | 0.04 | 0.02 | 0.01 | -0.01 | 0.01 |
| 125 | rs1957563 | T | 0.36 | 0.03 | 0.02 | 0.01 | -0.01 | 0.01 |
| 126 | rs1984195 | A | 0.24 | 0.03 | 0.00 | 0.01 | -0.01 | 0.01 |
| 127 | rs1994158 | G | -0.25 | 0.04 | 0.00 | 0.01 | -0.01 | 0.01 |
| 128 | rs2014408 | T | 0.52 | 0.04 | 0.00 | 0.01 | -0.02 | 0.01 |
| 129 | rs2024385 | A | -0.26 | 0.03 | -0.01 | 0.01 | -0.01 | 0.01 |
| 130 | rs2111557 | T | 0.18 | 0.03 | 0.01 | 0.01 | -0.01 | 0.01 |
| 131 | rs2126474 | T | -0.26 | 0.03 | 0.01 | 0.01 | 0.01 | 0.01 |
| 132 | rs2129869 | T | 0.26 | 0.04 | -0.01 | 0.01 | -0.05 | 0.01 |
| 133 | rs2161967 | G | -0.28 | 0.03 | 0.00 | 0.01 | 0.00 | 0.01 |
| 134 | rs2177843 | T | 0.44 | 0.04 | 0.00 | 0.01 | 0.03 | 0.01 |
| 135 | rs2236295 | T | -0.30 | 0.03 | 0.00 | 0.01 | 0.03 | 0.01 |
| 136 | rs2249105 | G | -0.29 | 0.03 | -0.02 | 0.01 | 0.05 | 0.01 |
| 137 | rs2276153 | G | -0.33 | 0.04 | -0.02 | 0.01 | 0.04 | 0.01 |
| 138 | rs2283500 | C | -0.31 | 0.05 | -0.01 | 0.01 | 0.01 | 0.01 |
| 139 | rs2291434 | T | -0.26 | 0.03 | -0.01 | 0.01 | 0.03 | 0.01 |
| 140 | rs2353940 | C | 0.21 | 0.04 | 0.00 | 0.01 | -0.01 | 0.01 |
| 141 | rs2354862 | C | -0.25 | 0.03 | 0.01 | 0.01 | 0.01 | 0.01 |
| 142 | rs2384063 | T | 0.33 | 0.04 | -0.01 | 0.01 | 0.00 | 0.01 |
| 143 | rs2392929 | G | 0.75 | 0.04 | 0.03 | 0.01 | 0.01 | 0.01 |
| 144 | rs2423514 | G | -0.30 | 0.03 | 0.00 | 0.01 | 0.01 | 0.01 |
| 145 | rs246973 | T | 0.25 | 0.03 | 0.01 | 0.01 | 0.00 | 0.01 |
| 146 | rs2470004 | T | -0.35 | 0.04 | 0.01 | 0.01 | 0.02 | 0.01 |
| 147 | rs2493134 | C | 0.37 | 0.03 | 0.01 | 0.01 | 0.00 | 0.01 |
| 148 | rs2498323 | A | 0.32 | 0.05 | 0.02 | 0.01 | -0.02 | 0.01 |
| 149 | rs2580350 | A | 0.18 | 0.03 | 0.01 | 0.01 | -0.02 | 0.01 |
| 150 | rs2589218 | C | 0.23 | 0.03 | 0.00 | 0.01 | -0.01 | 0.01 |
| 151 | rs2598 | G | -0.17 | 0.03 | 0.00 | 0.01 | -0.01 | 0.01 |
| 152 | rs2608029 | G | -0.18 | 0.03 | -0.02 | 0.01 | 0.01 | 0.01 |
| 153 | rs2610990 | G | 0.29 | 0.03 | 0.03 | 0.01 | -0.03 | 0.01 |
| 154 | rs2627313 | T | 0.32 | 0.03 | 0.03 | 0.01 | -0.02 | 0.01 |
| 155 | rs262986 | A | -0.24 | 0.03 | 0.01 | 0.01 | 0.01 | 0.01 |
| 156 | rs2643826 | T | 0.45 | 0.03 | 0.03 | 0.01 | -0.02 | 0.01 |
| 157 | rs2652812 | T | -0.25 | 0.04 | -0.01 | 0.01 | 0.02 | 0.01 |
| 158 | rs268263 | A | 0.59 | 0.04 | 0.00 | 0.01 | 0.01 | 0.01 |
| 159 | rs2689690 | T | -0.27 | 0.03 | -0.01 | 0.01 | 0.02 | 0.01 |
| 160 | rs2724377 | G | -0.19 | 0.03 | 0.00 | 0.01 | 0.00 | 0.01 |
| 161 | rs2744139 | C | -0.24 | 0.04 | -0.03 | 0.01 | 0.00 | 0.01 |
| 162 | rs2760748 | A | 0.36 | 0.05 | 0.02 | 0.02 | -0.01 | 0.01 |
| 163 | rs2776037 | C | 0.19 | 0.03 | -0.01 | 0.01 | 0.01 | 0.01 |
| 164 | rs2801008 | G | 0.19 | 0.03 | -0.01 | 0.01 | 0.00 | 0.01 |
| 165 | rs2833834 | A | 0.22 | 0.03 | 0.00 | 0.01 | -0.01 | 0.01 |
| 166 | rs2853736 | G | -0.24 | 0.03 | -0.01 | 0.01 | -0.02 | 0.01 |
| 167 | rs28572357 | C | 0.27 | 0.03 | 0.01 | 0.01 | 0.03 | 0.01 |
| 168 | rs28650790 | T | 0.23 | 0.04 | 0.02 | 0.01 | -0.08 | 0.01 |
| 169 | rs28688791 | C | 0.32 | 0.04 | 0.05 | 0.01 | -0.01 | 0.01 |
| 170 | rs28866311 | G | 0.28 | 0.03 | 0.01 | 0.01 | 0.00 | 0.01 |
| 171 | rs2904315 | G | 0.21 | 0.03 | -0.01 | 0.01 | 0.02 | 0.01 |
| 172 | rs2913920 | T | 0.24 | 0.04 | 0.01 | 0.01 | -0.01 | 0.01 |
| 173 | rs2957688 | A | 0.35 | 0.03 | 0.00 | 0.01 | -0.01 | 0.01 |
| 174 | rs3098186 | T | -0.24 | 0.03 | 0.00 | 0.01 | 0.01 | 0.01 |
| 175 | rs3104552 | C | -0.24 | 0.03 | 0.00 | 0.01 | 0.00 | 0.01 |
| 176 | rs34025993 | G | -0.22 | 0.03 | -0.03 | 0.01 | -0.01 | 0.01 |
| 177 | rs34072724 | A | -0.24 | 0.03 | -0.01 | 0.01 | 0.04 | 0.01 |
| 178 | rs34413141 | A | -0.35 | 0.04 | 0.00 | 0.01 | 0.01 | 0.01 |
| 179 | rs34727427 | C | 0.24 | 0.03 | 0.00 | 0.01 | 0.00 | 0.01 |
| 180 | rs34917849 | C | 0.31 | 0.05 | 0.02 | 0.01 | -0.03 | 0.01 |
| 181 | rs34941092 | A | -0.32 | 0.04 | 0.02 | 0.01 | 0.01 | 0.01 |
| 182 | rs35098810 | C | -0.20 | 0.04 | -0.02 | 0.01 | 0.00 | 0.01 |
| 183 | rs35413927 | G | 0.30 | 0.03 | 0.00 | 0.01 | 0.00 | 0.01 |
| 184 | rs35444 | G | -0.44 | 0.03 | -0.04 | 0.01 | 0.00 | 0.01 |
| 185 | rs35680304 | T | 0.27 | 0.03 | 0.02 | 0.01 | 0.00 | 0.01 |
| 186 | rs35783704 | A | -0.46 | 0.05 | -0.04 | 0.02 | 0.01 | 0.01 |
| 187 | rs3735533 | C | 0.91 | 0.06 | 0.04 | 0.01 | 0.03 | 0.01 |
| 188 | rs3764400 | C | -0.37 | 0.04 | -0.02 | 0.01 | 0.03 | 0.01 |
| 189 | rs3772219 | C | -0.27 | 0.03 | -0.01 | 0.01 | 0.00 | 0.01 |
| 190 | rs3802517 | A | 0.25 | 0.03 | 0.00 | 0.01 | -0.01 | 0.01 |
| 191 | rs3807925 | G | 0.19 | 0.03 | 0.00 | 0.01 | 0.00 | 0.01 |
| 192 | rs3815460 | G | 0.29 | 0.05 | -0.02 | 0.01 | -0.01 | 0.01 |
| 193 | rs3819532 | C | 0.19 | 0.03 | 0.01 | 0.01 | -0.01 | 0.01 |
| 194 | rs3845811 | G | 0.29 | 0.03 | -0.01 | 0.01 | 0.00 | 0.01 |
| 195 | rs3860770 | A | -0.27 | 0.03 | -0.02 | 0.01 | -0.01 | 0.01 |
| 196 | rs3980686 | T | -0.50 | 0.05 | -0.02 | 0.01 | 0.01 | 0.01 |
| 197 | rs404100 | T | 0.19 | 0.03 | 0.01 | 0.01 | 0.01 | 0.01 |
| 198 | rs4143175 | C | -0.22 | 0.04 | 0.00 | 0.01 | 0.00 | 0.01 |
| 199 | rs42032 | A | -0.32 | 0.03 | -0.05 | 0.01 | 0.01 | 0.01 |
| 200 | rs4245599 | G | 0.18 | 0.03 | 0.00 | 0.01 | 0.01 | 0.01 |
| 201 | rs4260863 | G | -0.19 | 0.03 | 0.00 | 0.01 | -0.02 | 0.01 |
| 202 | rs4274337 | G | 0.30 | 0.04 | 0.01 | 0.01 | -0.02 | 0.01 |
| 203 | rs4408839 | G | 0.23 | 0.03 | 0.00 | 0.01 | 0.02 | 0.01 |
| 204 | rs4440615 | A | -0.22 | 0.03 | -0.01 | 0.01 | 0.00 | 0.01 |
| 205 | rs4499560 | T | 0.22 | 0.03 | 0.00 | 0.01 | 0.00 | 0.01 |
| 206 | rs4511593 | T | -0.29 | 0.03 | 0.00 | 0.01 | -0.01 | 0.01 |
| 207 | rs4553000 | T | -0.20 | 0.03 | -0.01 | 0.01 | -0.01 | 0.01 |
| 208 | rs4577304 | C | 0.18 | 0.03 | 0.01 | 0.01 | -0.01 | 0.01 |
| 209 | rs4651224 | T | 0.20 | 0.03 | -0.01 | 0.01 | -0.01 | 0.01 |
| 210 | rs4734868 | G | 0.18 | 0.03 | -0.01 | 0.01 | 0.00 | 0.01 |
| 211 | rs4775769 | G | 0.42 | 0.05 | 0.00 | 0.02 | 0.00 | 0.01 |
| 212 | rs483071 | T | 0.27 | 0.03 | 0.02 | 0.01 | 0.01 | 0.01 |
| 213 | rs4834792 | A | 0.20 | 0.03 | 0.00 | 0.01 | 0.00 | 0.01 |
| 214 | rs4838021 | T | -0.30 | 0.05 | -0.02 | 0.01 | 0.05 | 0.01 |
| 215 | rs4873492 | T | 0.34 | 0.04 | 0.00 | 0.01 | -0.02 | 0.01 |
| 216 | rs4876133 | C | 0.22 | 0.03 | 0.03 | 0.01 | 0.01 | 0.01 |
| 217 | rs4888408 | A | 0.37 | 0.03 | 0.01 | 0.01 | 0.00 | 0.01 |
| 218 | rs4925159 | A | 0.22 | 0.03 | 0.01 | 0.01 | 0.01 | 0.01 |
| 219 | rs4952609 | G | -0.21 | 0.03 | -0.01 | 0.01 | 0.00 | 0.01 |
| 220 | rs4955575 | C | -0.22 | 0.03 | -0.01 | 0.01 | 0.00 | 0.01 |
| 221 | rs4961293 | T | 0.23 | 0.03 | 0.02 | 0.01 | 0.00 | 0.01 |
| 222 | rs509564 | T | 0.26 | 0.04 | 0.00 | 0.01 | 0.01 | 0.01 |
| 223 | rs509833 | G | -0.33 | 0.04 | -0.02 | 0.01 | -0.01 | 0.01 |
| 224 | rs55732192 | T | -0.34 | 0.05 | -0.02 | 0.01 | 0.00 | 0.01 |
| 225 | rs55944332 | G | 0.26 | 0.04 | 0.01 | 0.01 | 0.00 | 0.01 |
| 226 | rs56407827 | T | 0.36 | 0.03 | 0.01 | 0.01 | 0.01 | 0.01 |
| 227 | rs571689 | T | 0.23 | 0.03 | 0.02 | 0.01 | 0.01 | 0.01 |
| 228 | rs573455 | G | -0.20 | 0.03 | -0.01 | 0.01 | -0.01 | 0.01 |
| 229 | rs5742643 | C | 0.22 | 0.03 | 0.01 | 0.01 | -0.01 | 0.01 |
| 230 | rs57786342 | A | 0.23 | 0.04 | 0.00 | 0.01 | -0.02 | 0.01 |
| 231 | rs57866767 | C | -0.45 | 0.03 | -0.02 | 0.01 | 0.01 | 0.01 |
| 232 | rs57946343 | C | -0.72 | 0.04 | -0.03 | 0.01 | 0.00 | 0.01 |
| 233 | rs60138042 | G | -0.34 | 0.06 | 0.01 | 0.01 | 0.03 | 0.02 |
| 234 | rs60191654 | G | 0.24 | 0.04 | 0.00 | 0.01 | 0.01 | 0.01 |
| 235 | rs6026578 | G | 0.19 | 0.03 | 0.00 | 0.01 | 0.02 | 0.01 |
| 236 | rs6026744 | T | 0.71 | 0.05 | 0.02 | 0.01 | -0.01 | 0.01 |
| 237 | rs6029756 | A | -0.27 | 0.03 | -0.02 | 0.01 | 0.00 | 0.01 |
| 238 | rs6031431 | G | 0.26 | 0.03 | 0.03 | 0.01 | 0.00 | 0.01 |
| 239 | rs60444686 | A | 0.59 | 0.08 | 0.02 | 0.02 | 0.01 | 0.02 |
| 240 | rs604723 | C | 0.66 | 0.03 | 0.02 | 0.01 | -0.01 | 0.01 |
| 241 | rs6054139 | A | 0.21 | 0.03 | 0.00 | 0.01 | 0.01 | 0.01 |
| 242 | rs6058088 | G | -0.28 | 0.04 | 0.00 | 0.01 | 0.03 | 0.01 |
| 243 | rs6078093 | A | -0.18 | 0.03 | 0.00 | 0.01 | 0.01 | 0.01 |
| 244 | rs6090907 | A | -0.39 | 0.04 | -0.02 | 0.01 | 0.01 | 0.01 |
| 245 | rs60909079 | C | -0.21 | 0.04 | 0.01 | 0.01 | -0.01 | 0.01 |
| 246 | rs60991988 | G | -0.38 | 0.05 | -0.01 | 0.01 | -0.01 | 0.01 |
| 247 | rs6108787 | G | 0.43 | 0.03 | 0.03 | 0.01 | 0.01 | 0.01 |
| 248 | rs61772592 | G | 0.32 | 0.05 | 0.03 | 0.01 | -0.02 | 0.01 |
| 249 | rs62076622 | G | -0.24 | 0.04 | -0.01 | 0.01 | 0.00 | 0.01 |
| 250 | rs62082230 | A | -0.19 | 0.03 | 0.01 | 0.01 | -0.01 | 0.01 |
| 251 | rs62309747 | A | -0.22 | 0.03 | -0.02 | 0.01 | 0.02 | 0.01 |
| 252 | rs62512914 | G | -0.21 | 0.03 | -0.01 | 0.01 | 0.01 | 0.01 |
| 253 | rs641620 | C | 0.32 | 0.04 | 0.01 | 0.01 | -0.02 | 0.01 |
| 254 | rs6438857 | C | -0.27 | 0.03 | -0.02 | 0.01 | 0.00 | 0.01 |
| 255 | rs6445583 | A | 0.28 | 0.03 | 0.04 | 0.01 | -0.01 | 0.01 |
| 256 | rs6452769 | A | -0.31 | 0.04 | 0.00 | 0.01 | 0.00 | 0.01 |
| 257 | rs6490019 | G | 0.29 | 0.03 | 0.00 | 0.01 | 0.02 | 0.01 |
| 258 | rs6504213 | C | 0.30 | 0.03 | 0.00 | 0.01 | -0.02 | 0.01 |
| 259 | rs6540119 | T | -0.20 | 0.03 | 0.01 | 0.01 | -0.02 | 0.01 |
| 260 | rs6562778 | G | -0.18 | 0.03 | 0.00 | 0.01 | 0.01 | 0.01 |
| 261 | rs658780 | G | 0.20 | 0.03 | -0.02 | 0.01 | 0.00 | 0.01 |
| 262 | rs665445 | A | -0.19 | 0.03 | 0.00 | 0.01 | 0.00 | 0.01 |
| 263 | rs66864335 | A | -0.40 | 0.04 | -0.03 | 0.01 | 0.03 | 0.01 |
| 264 | rs6699618 | G | -0.91 | 0.04 | -0.02 | 0.01 | 0.02 | 0.01 |
| 265 | rs6731373 | A | 0.19 | 0.03 | 0.00 | 0.01 | 0.02 | 0.01 |
| 266 | rs6732123 | C | -0.17 | 0.03 | -0.01 | 0.01 | 0.00 | 0.01 |
| 267 | rs6737318 | G | -0.23 | 0.04 | -0.01 | 0.01 | 0.00 | 0.01 |
| 268 | rs67617547 | G | -0.18 | 0.03 | 0.00 | 0.01 | 0.02 | 0.01 |
| 269 | rs6788907 | A | 0.22 | 0.03 | 0.02 | 0.01 | -0.01 | 0.01 |
| 270 | rs6788984 | G | -0.30 | 0.04 | -0.03 | 0.01 | 0.00 | 0.01 |
| 271 | rs68085857 | T | 0.27 | 0.04 | 0.01 | 0.01 | 0.00 | 0.01 |
| 272 | rs6870654 | C | -0.21 | 0.03 | -0.03 | 0.01 | 0.00 | 0.01 |
| 273 | rs6892983 | A | 0.34 | 0.03 | 0.03 | 0.01 | 0.00 | 0.01 |
| 274 | rs6921291 | T | 0.36 | 0.04 | 0.01 | 0.01 | -0.01 | 0.01 |
| 275 | rs6957161 | G | -0.21 | 0.03 | -0.02 | 0.01 | -0.01 | 0.01 |
| 276 | rs6961048 | G | 0.53 | 0.05 | 0.05 | 0.01 | -0.02 | 0.01 |
| 277 | rs6986368 | T | 0.21 | 0.03 | 0.01 | 0.01 | -0.02 | 0.01 |
| 278 | rs7012866 | G | 0.23 | 0.03 | 0.01 | 0.01 | -0.01 | 0.01 |
| 279 | rs702395 | T | 0.23 | 0.03 | 0.00 | 0.01 | 0.00 | 0.01 |
| 280 | rs7026176 | T | -0.19 | 0.03 | 0.00 | 0.01 | 0.02 | 0.01 |
| 281 | rs7045409 | A | -0.19 | 0.03 | -0.01 | 0.01 | 0.02 | 0.01 |
| 282 | rs708117 | A | 0.29 | 0.03 | -0.01 | 0.01 | 0.01 | 0.01 |
| 283 | rs7093894 | A | 0.24 | 0.04 | 0.01 | 0.01 | -0.03 | 0.01 |
[truncated: 106,570 more chars]
